# Novel Antagonist of the Type 2 Lysophosphatidic Acid Receptor (LPA_2_), UCM-14216, Ameliorates Spinal Cord Injury in Mice

**DOI:** 10.1021/acs.jmedchem.2c00046

**Published:** 2022-08-10

**Authors:** Nora Khiar-Fernández, Debora Zian, Henar Vázquez-Villa, R. Fernando Martínez, Andrea Escobar-Peña, Román Foronda-Sainz, Manisha Ray, Maria Puigdomenech-Poch, Giovanni Cincilla, Melchor Sánchez-Martínez, Yasuyuki Kihara, Jerold Chun, Rubèn López-Vales, María L. López-Rodríguez, Silvia Ortega-Gutiérrez

**Affiliations:** ‡ Departamento de Química Orgánica I, Facultad de Ciencias Químicas, Universidad Complutense de Madrid, Madrid E-28040, Spain; § Translational Neuroscience Initiative, Sanford Burnham Prebys Medical Discovery Institute, 10901 North Torrey Pines Road, La Jolla, California 92037, United States; ⊥ Departament de Biologia Cel·lular, Fisiologia i Immunologia, Institut de Neurociències, Centro de Investigación Biomédica en Red sobre Enfermedades Neurodegenerativas (CIBERNED), Universitat Autònoma de Barcelona, Bellaterra, BarcelonaE-08193, Spain; ∥ Molomics, Barcelona Science Park, Baldiri i Reixac 4-8, Barcelona E-08028, Spain; # Burua Scientific, Sant Pere de Ribes E-08810, Spain

## Abstract

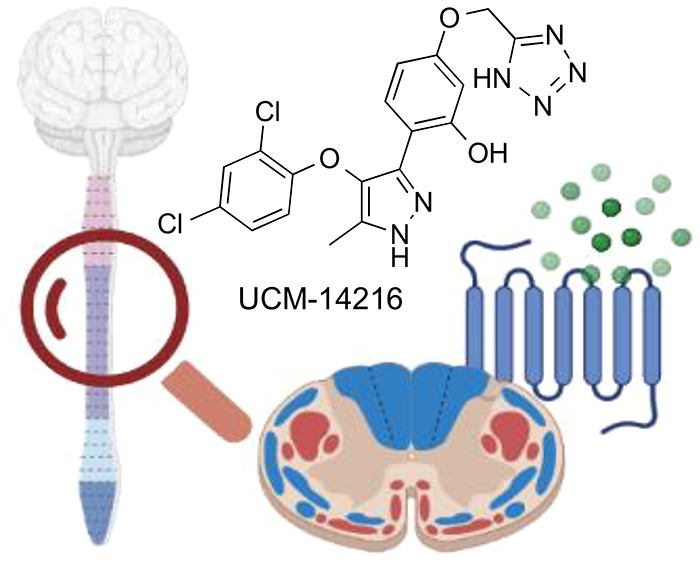

Spinal cord injuries (SCIs) irreversibly disrupt spinal connectivity, leading to permanent neurological disabilities. Current medical treatments for reducing the secondary damage that follows the initial injury are limited to surgical decompression and anti-inflammatory drugs, so there is a pressing need for new therapeutic strategies. Inhibition of the type 2 lysophosphatidic acid receptor (LPA_2_) has recently emerged as a new potential pharmacological approach to decrease SCI-associated damage. Toward validating this receptor as a target in SCI, we have developed a new series of LPA_2_ antagonists, among which compound **54** (UCM-14216) stands out as a potent and selective LPA_2_ receptor antagonist (*E*_max_ = 90%, IC_50_ = 1.9 μM, *K*_D_ = 1.3 nM; inactive at LPA_1,3–6_ receptors). This compound shows efficacy in an in vivo mouse model of SCI in an LPA_2_-dependent manner, confirming the potential of LPA_2_ inhibition for providing a new alternative for treating SCI.

## Introduction

**Figure 1:**
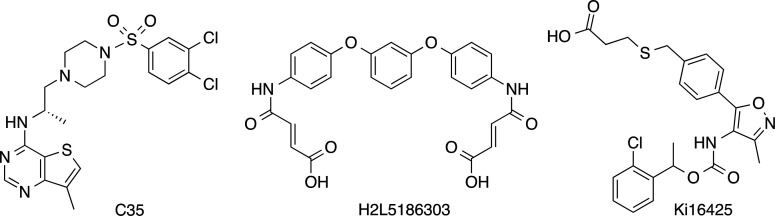
Structure of the LPA_2_ antagonists C35, H2L5186303, and Ki16425.

A spinal cord injury (SCI) is defined as damage to the spinal cord that provokes a temporary or permanent impairment of its function. It has negative consequences for the physical and social well-being of patients and imposes an important economic burden to the individual and the health care system. SCI can have traumatic or nontraumatic origins. The former happens when an external physical impact acutely harms the spinal cord, whereas the latter is associated with disease development, such as a tumor, an infection, or a neurodegenerative process. Regardless of the etiology, the primary injury damages cells and initiates a complex secondary cascade of secondary degeneration characterized by ischemia, excitotoxicity, and inflammatory processes that lead to the death of neurons and glial cells. This process is followed by a reorganization of the structural architecture of the spinal cord and by the formation of glial scars that, together with the poor capacity of the central nervous system (CNS) to promote remyelination and axonal growth, causes irreversible neurological deficits. Considering the negative impact of SCI, it is clear that prevention of the primary injury is desirable, as would an efficacious treatment to minimize secondary injury events to prevent functional impairments. The last several years have witnessed an important advancement of the field, with the development of different experimental neuroprotective and neuroregenerative therapies that have been translated from preclinical studies into clinical trials.^1−3^ However, the current medical reality is that there is no treatment for acute SCI because methylprednisolone, which was the standard treatment for acute SCI, is no longer used for the management of spinal cord trauma in many countries based on several reports demonstrating its lack of therapeutic efficacy and its undesirable side effects related to immunosuppression and gastrointestinal bleeding.^4^ Hence, it is evident there is a crucial need to develop new treatments for SCI. In this regard, there is a consensus in that primary injury cannot be therapeutically addressed, but secondary cell damage events that occur after SCI could be susceptible to therapeutic intervention. Hence, much research effort has focused on delineation of the receptor pathways responsible for the irreversible cellular damage that occurs after SCI, because they could represent new therapeutic targets for novel drug treatments. In this context, bioactive lipids have recently emerged as major players in the initiation and maintenance of the pro-inflammatory environment that prevent tissue repair and recovery of homeostasis.^5^ Among them, lysophosphatidic acid (LPA, 1-acyl-*sn*-glycerol-3-phosphate) has received an increasing attention.^6,7^ Although LPA can refer to multiple different species of lysophospholipids with saturated (16:0, 18:0) and unsaturated (16:1, 18:1, 18:2, 20:4) acyl chains, in the context of SCI, LPA 18:1 (1-oleoyl-*sn*-glycerol-3-phosphate) appears to be the most important form.^8^ The increase in LPA levels in the CNS after traumatic injury has detrimental effects, as it has been confirmed by experiments showing that intraspinal injection of LPA leads to inflammation and demyelination.^8^ However, taking into account that LPA can activate at least six different receptors (LPA_1–6_) that belong to the G protein-coupled receptor (GPCR) superfamily,^9−11^ the next important step is to determine which specific receptor subtype(s) is responsible for the deleterious effect of pathological LPA exposure. In this regard, the importance of LPA_1_ as a target for the treatment of SCI has been well established,^8,12^ but this receptor does not account for all the effects observed with LPA. Very recently, LPA_2_ has been postulated as a key receptor in mediating the effects of LPA in SCI.^13^ However, its validation has been hampered by the lack of selective antagonists. Currently, only two compounds (C35 and H2L5186303, Figure 1) have been characterized as potent (IC_50_ values at LPA_2_ of 0.017 and 0.0089 μM, respectively) and selective LPA_2_ antagonists (IC_50_ values >50 μM at LPA_1_ and LPA_3_ for C35 and 1.23 and 27.3 μM at LPA_1_ and LPA_3_ for H2L5186303).^14,15^ However, their selectivity profile versus the other LPA receptors (LPA_4–6_), pharmacokinetic properties, and in vivo efficacy have not been studied. Another tool compound widely used to study the effect of blocking LPA receptor signaling is Ki16425 (Figure 1), but although it has good in vitro potency, this derivative is a nonselective antagonist with submicromolar activity at LPA_1_ and LPA_3_ and lower affinity at LPA_2_ (IC_50_ values of 0.34, 0.93, and 6.5 μM, respectively)^16^ and with limited in vivo activity that may reflect its short half-life.^17^

New potent and selective LPA_2_ antagonists could enable the validation of this receptor as a target for the treatment of SCI and might represent a new therapeutic avenue. Here we report the development of the most potent and selective LPA_2_ antagonist described so far, compound UCM-14216 (**54**), which has an IC_50_ value of 1.9 μM as an LPA_2_ antagonist, a *K*_D_ value of 1.3 nM, and a selectivity over other LPA receptor subtypes (LPA_1_ and LPA_3–6_, with 10-fold selectivity in terms of IC_50_ value with respect to LPA_1_ and LPA_3_ and 10-fold selectivity versus LPA_6_ and >50-fold selectivity versus LPA_4_ and LPA_5_ in terms of *K*_D_). In addition, this compound significantly improves motor recovery in an in vivo model of SCI, supporting the importance of LPA_2_ for the treatment of SCI.

## Results and Discussion

**Figure 2:**
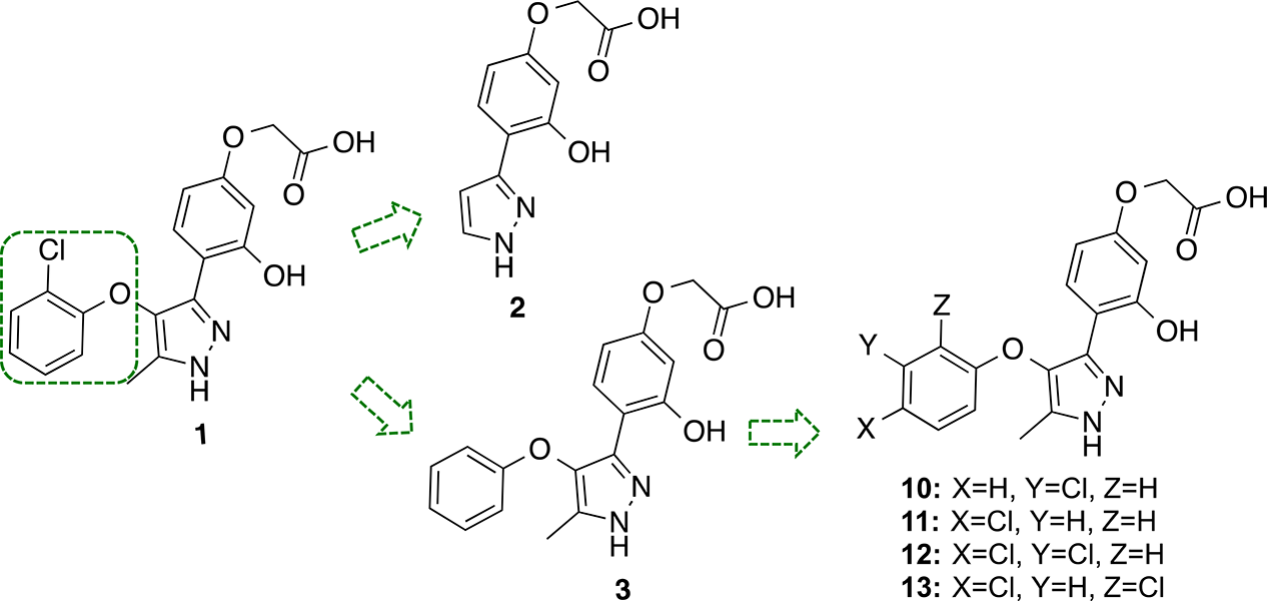
Design of new LPA_2_ antagonists **2**, **3**, and **10**–**13**.

Within a broad project focused on the discovery of new ligands for LPA receptors,^18^ we started our search of potent and selective LPA_2_ antagonists through an in-house screen using a functional assay to detect calcium mobilization in cells stably transfected with the LPA_2_ receptor in which the compounds under study were added at a fixed dose of 10 μM and the cells were subsequently stimulated with LPA at the same concentration. We considered active those compounds able to reduce the LPA-mediated calcium response by at least 30%. Among all tested molecules, compound **1** (Figure 2) showed a consistent antagonist signal at LPA_2_ receptor, absence of significant agonist activity at this receptor (Figure S1), and selectivity versus LPA_1_ and LPA_3_ receptors, so it was selected as our initial hit. However, its moderate antagonism at LPA_2_ at 10 μM (*E*_max_ = 48 ± 9%) led us to carry out a systematic structural exploration of this compound with the aim of improving its biological activity.

### Structure–Activity Relationship (SAR) Study of Hit 1

First, we tried to establish the relative importance of the different parts of the molecule for the LPA_2_ antagonist activity. We started by studying the influence of the chlorophenoxy group by removing the whole moiety or just the halogen atom with the synthesis of compounds **2** and **3** (Figure 2).

**Scheme 1:**
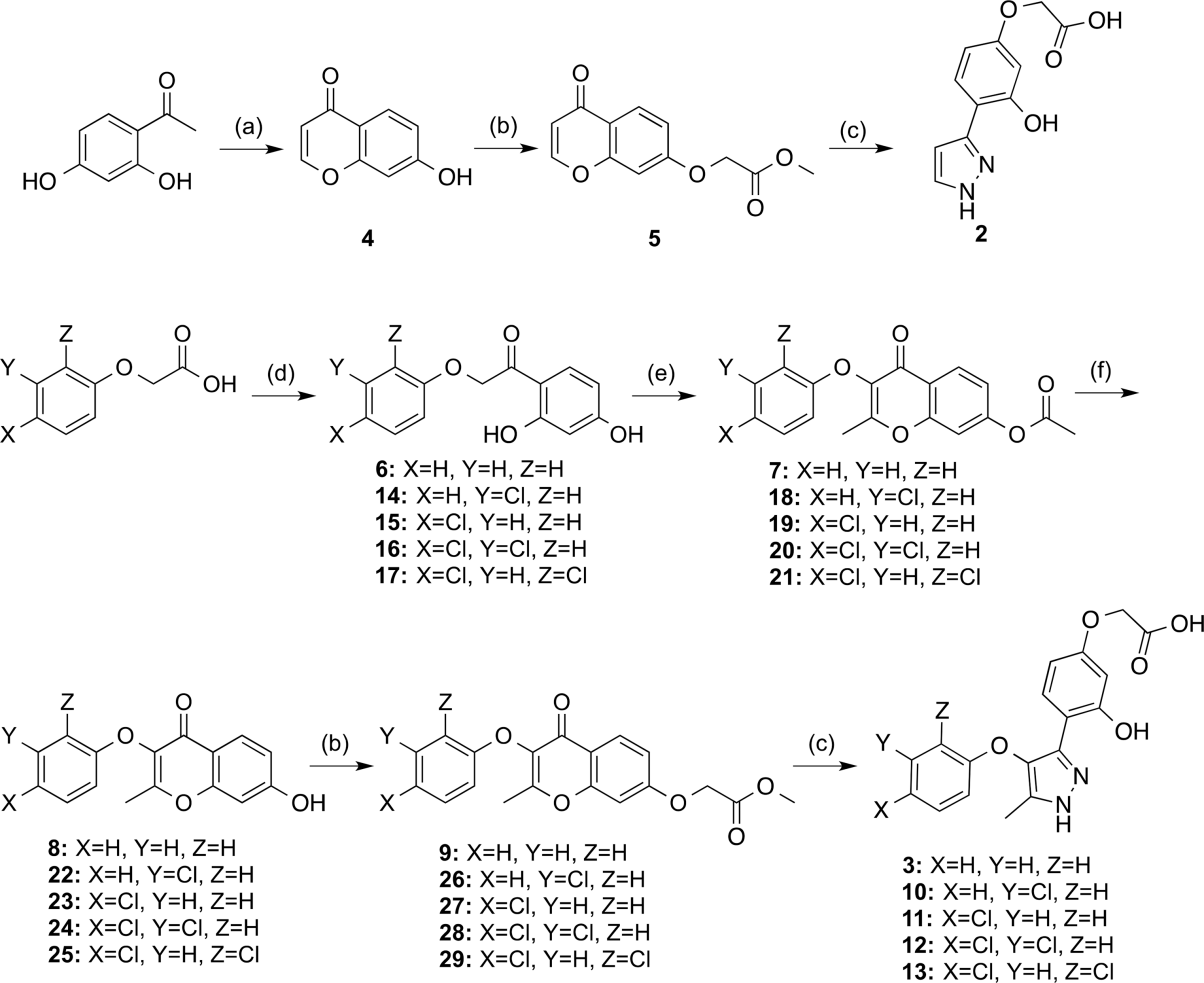
Synthesis of Compounds **2**, **3**, and **10**–**13** Reagents and conditions: (a) CH(OEt)_3_, 70% HClO_4_, H_2_O, rt, 13 h, 47%; (b) methyl bromoacetate, K_2_CO_3_, acetone, reflux, 3 h, 24–98%; (c) (i) 65% N_2_H_4_·H_2_O, EtOH, reflux, 30 min, 77–99%; (ii) 2 M NaOH, EtOH, reflux, 12 h, 67–99%; (d) (i) SOCl_2_, toluene, 110 °C, 16 h, 99%; (ii) resorcinol, BF_3_·Et_2_O, DCM, reflux, 4–5 h, 19–25%; (e) acetic anhydride, Et_3_N, NaOAc, 140 °C, 2–3 h, 83–99%; (f) conc. HCl, EtOH, reflux, 2 h, 75–99%.

Compound **2** was prepared from commercially available 1-(2,4-dihydroxyphenyl)ethanone by treatment with triethyl orthoformate and perchloric acid. Then, resulting hydroxychromone **4** was alkylated with methyl bromoacetate and treated with an excess of hydrazine to obtain desired derivative **2** (Scheme 1), through opening of the pyrone ring and subsequent formation of pyrazole ring. The reaction with hydrazine promoted the simultaneous transformation of the ester group to the corresponding hydrazide, which was hydrolyzed to obtain the target carboxylic acid. With respect to compound **3**, its synthesis started with a Friedel–Crafts acylation between 2-phenoxyacetyl chloride and resorcinol. Next, the Kostanecki–Robinson reaction between the resulting ketone **6** and acetic anhydride afforded chromone **7** in a good yield, which was, after hydrolysis of the acetyl group in acid media, alkylated with methyl bromoacetate to obtain intermediate **9**. Finally, treatment with hydrazine gave target compound **3** (Scheme 1). Antagonist activity assays revealed that compound **2** was inactive at LPA_2_ (*E*_max_ = 7 ± 3%) whereas derivative **3** showed a low activity at LPA_2_ (*E*_max_ = 20 ± 7%, Table 1), highlighting the need not only of the chlorine atom but also of the whole phenoxy system for the LPA_2_ antagonist activity. Hence, we studied the influence of the position of the chloro substituent with the synthesis of compounds **10** and **11**, where the chlorine atom was located in a meta or para position, respectively (Figure 2). These syntheses were accomplished following a synthetic route similar to the one previously followed for compound **3** starting from the corresponding chlorophenoxyacetic acid and resorcinol (Scheme 1).

**Table 1:**
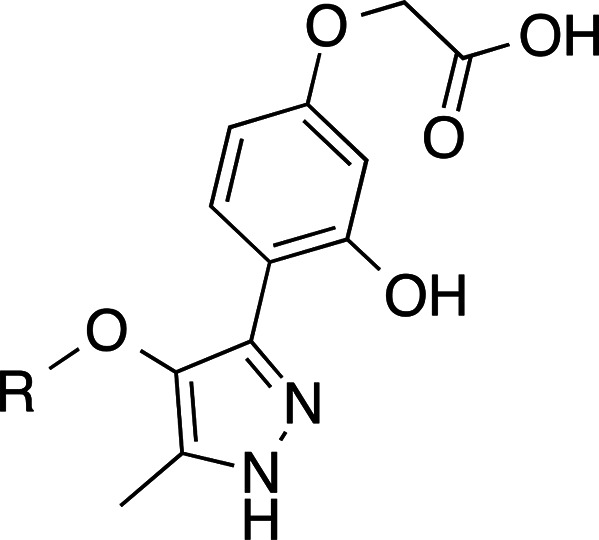

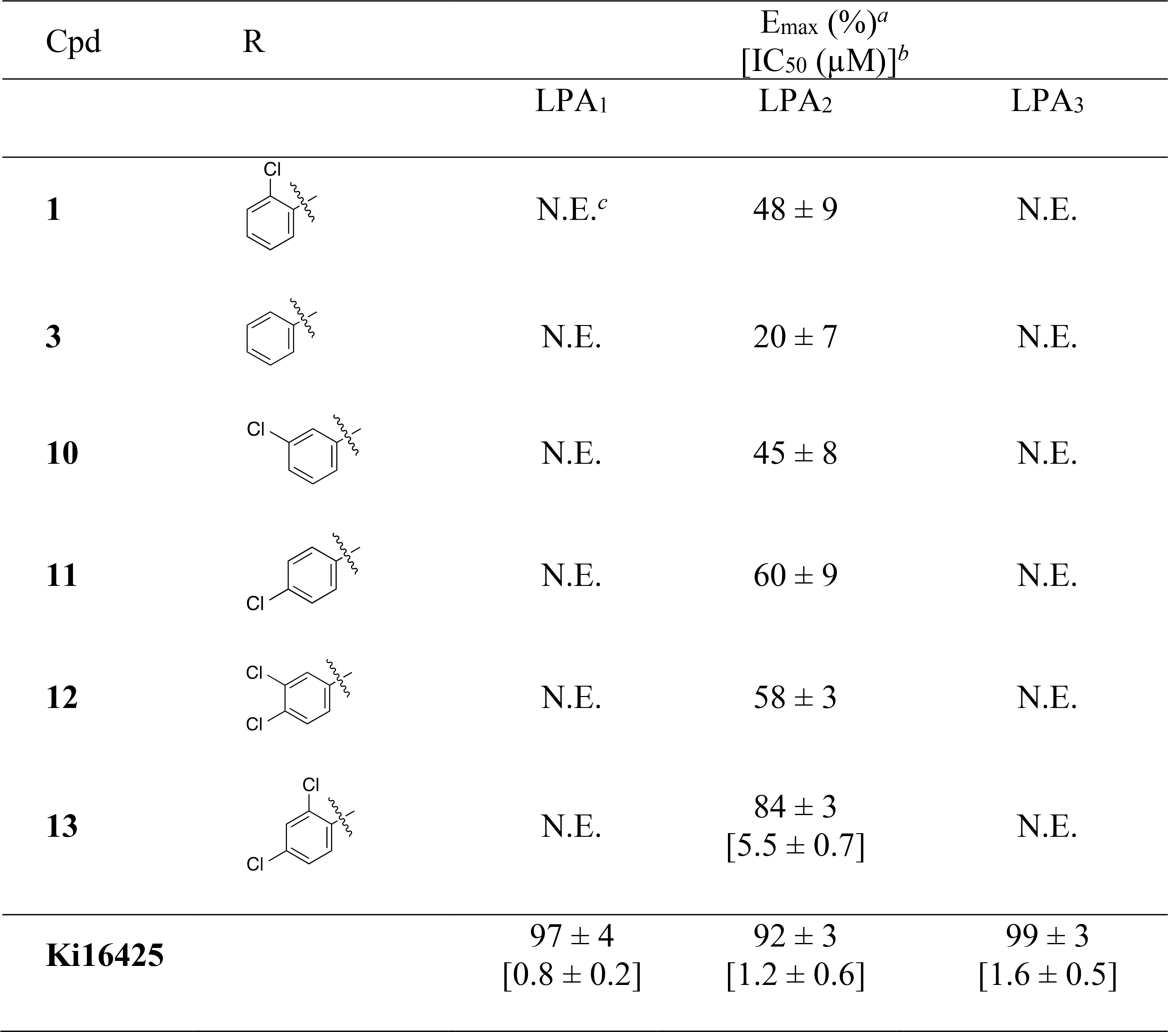
Antagonist Activities of Compounds **1**, **3**, **10**–**13**, and Ki16425 at LPA_1-3_

a *E*_max_ = maximum blockade effect of the activation induced by 10 μM of LPA (18:1, 1-oleoyl-*sn*-glycerol-3-phosphate) at a concentration of the compound under study of 10 μM.

b For *E*_max_ > 70%, IC_50_ values are expressed as mean ± s.e.m, from a minimum of two independent experiments, performed in triplicate.

c N.E., no effect was observed at the highest concentration of compound tested (10 μM).

Determination of the LPA_2_ antagonist character of compounds **10** and **11** revealed that whereas the former did not improve the antagonist activity of the initial hit **1** [*E*_max_ (**1**) = 48%; *E*_max_ (**10**) = 45%], the latter increased the LPA_2_ antagonist activity at the maximum concentration [*E*_max_ (**11**) = 60%] (Table 1). These results suggested that the chlorine atom was tolerated at the three positions, with the best result obtained for the para derivative, so it may be possible that the introduction of a second chlorine atom allowed further improvement of activity. Accordingly, compounds **12** and **13** were synthesized (Scheme 1) and tested for LPA_2_ activity (Table 1). Determination of their antagonist character revealed that introduction of the 2,4-dichlorophenoxy moiety yielded an excellent LPA_2_ antagonist [*E*_max_ (**13**) = 84%; IC_50_ (**13**) = 5.5 μM; Figure S2], with similar LPA_2_ antagonist activity to Ki16425, used as the reference ligand (Table 1). Also, to rule out the existence of partial agonism, we measured the agonist activity of compounds **3** and **10**–**13** at LPA_2_ receptors, and none of them was able to induce any significant activation of the receptor at 10 μM concentration (see Figure S1 for the result obtained for compound **13**, which is representative of the rest of the compounds).

**Figure 3:**
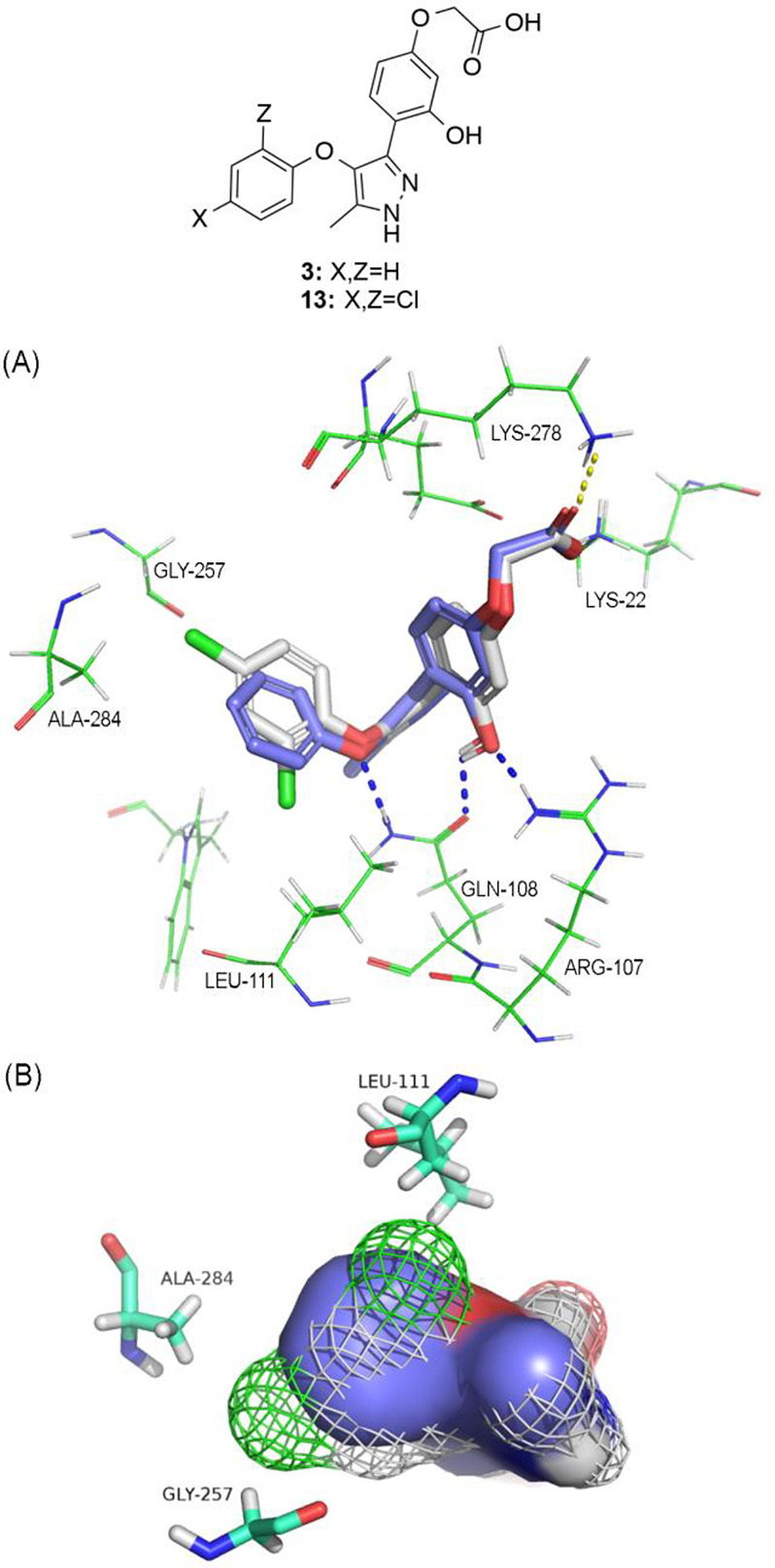
(A) LPA_1_ (PDB ID 4Z35)-derived homology model of LPA_2_R in complex with compounds **3** (in purple) and **13** (in white). The phenolic hydroxy group of both compounds is engaged in two hydrogen bonds with arginine 107 and glutamine 108 and the carboxylic acid group is engaged in two salt bridges with lysines 22 and 278. Also, the oxygen atom of the phenoxy moiety forms a hydrogen bond with glutamine 108. (B) Phenoxy moiety of the two compounds lies in the same hydrophobic pocket but compound **3**, represented here with a C-purple surface representation, cannot reach simultaneously residues glycine 257, alanine 284, and leucine 111 as compound **13** does, represented here as C-white mesh representation.

At this point, we considered that a detailed study of the molecular interactions involved in the affinity of compound **13** for LPA_2_ could help us to rationalize the activity results and also shed some light on the binding site of the compound. Hence, we built a homology model of LPA_2_ using the disclosed crystal structure of the LPA_1_ as a template.^19^ The best docking pose of compound **13** in the LPA_2_ receptor model (Figure 3A) suggests that the phenolic hydroxy group interacted with two hydrogen bonds with arginine 107 and glutamine 108 and the carboxylic acid group is engaged in two salt bridges with lysines 22 and 278 (Figure 3A). The dichlorophenoxy moiety lies in a hydrophobic pocket surrounded by leucine 261, leucine 111, glutamine 108, glycine 257, tryptophan 254, alanine 284, tyrosine 85 and phenylalanine 280. The chlorine atom in the 2-position points to residues leucine 111 and glutamine 108, while the one in the 4-position points to residues glycine 257 and alanine 284 (Figure 3A). Also, the oxygen atom of the phenoxy moiety forms a hydrogen bond with glutamine 108. Compound **3** adopts a similar pose to compound **13**, but its phenoxy moiety does not completely fill the hydrophobic pocket since it cannot simultaneously reach glycine 257, alanine 284, and leucine 111 as compound **13** does through its two chlorine atoms (Figure 3B).

**Scheme 2:**
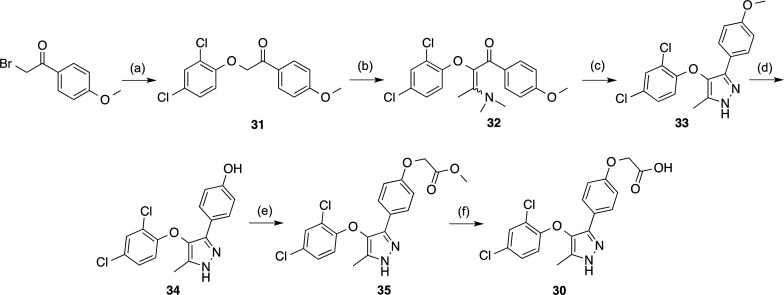
Synthesis of Compound **30** Reagents and conditions: (a) 2,4-dichlorophenol, DBU, DMF, MW, 140 °C, 30 min, 80%; (b) 1,1-dimethoxy-*N*,*N*-dimethylethanamine, 90 °C, 4 h, 52%; (c) 65% N_2_H_4_·H_2_O, EtOH, reflux, 1 h, 44%; (d) BBr_3_, DCM, −78 °C to rt, 21 h, 70%; (e) methyl bromoacetate, K_2_CO_3_, DMF, −20 °C to rt, 16 h, 32%; (f) 1 M NaOH, 1,4-dioxane, 60 °C, 2 h, 99%.

The importance of the phenolic hydroxy group was confirmed through the synthesis of compound **30** (Scheme 2), which was obtained starting with a Williamson reaction between 2-bromo-1-(4-methoxyphenyl)ethanone and 2,4-dichlorophenol under microwave (MW) irradiation, using 1,8-diazabicyclo[5.4.0]undec-7-ene (DBU) as a base. Then, treatment of the intermediate **31** with 1,1-dimethoxy-*N*,*N*-dimethylethanamine yielded enaminone **32**, which was reacted with hydrazine to obtain pyrazole **33**. Finally, removal of the methoxy group followed by *O*-alkylation with methyl bromoacetate and hydrolysis of the ester gave the target pyrazole **30** (Scheme 2), which was basically inactive at the LPA_2_ antagonist assay, with an *E*_max_ value of only 11%.

**Scheme 3:**
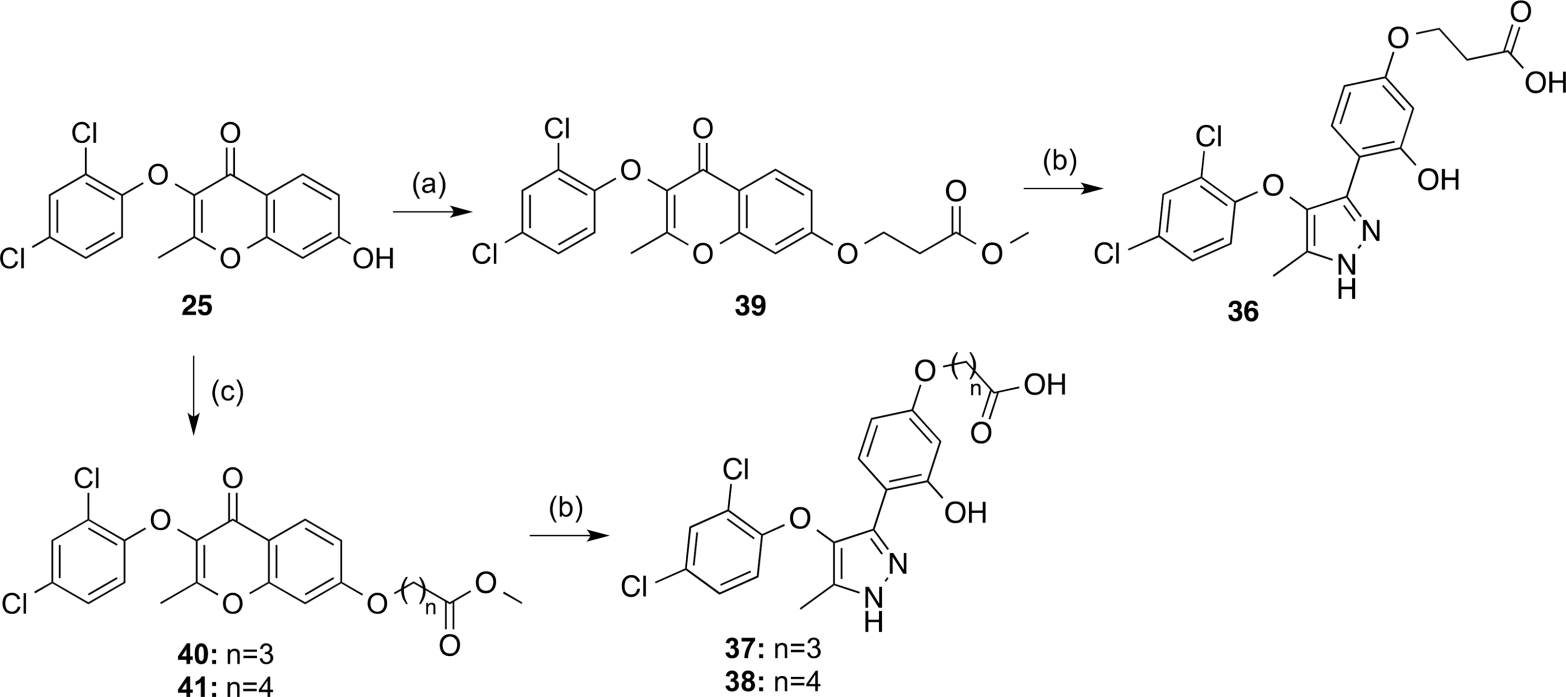
Synthesis of Compounds **36–38** Reagents and conditions: (a) methyl acrylate, DMAP, MW, 150 °C, 2.5 h, 10%; (b) (i) 65% N_2_H_4_·H_2_O, EtOH, reflux, 30 min, 24–86%; (ii) 2 M NaOH, EtOH, reflux, 12 h, 86–99%; (c) methyl 4-bromobutanoate or methyl 5-bromopentanoate, K_2_CO_3_, acetone, reflux, 3–5 h, 80–82%.

Next, we focused our attention on the influence of the distance between the oxygen atom and the carboxylic acid group in compound **13**. To determine the optimum length of the methylenic chain that separates these two moieties, we synthesized compounds **36**–**38**, which have 2–4 methylenes in the linker (Scheme 3).

**Figure 4:**
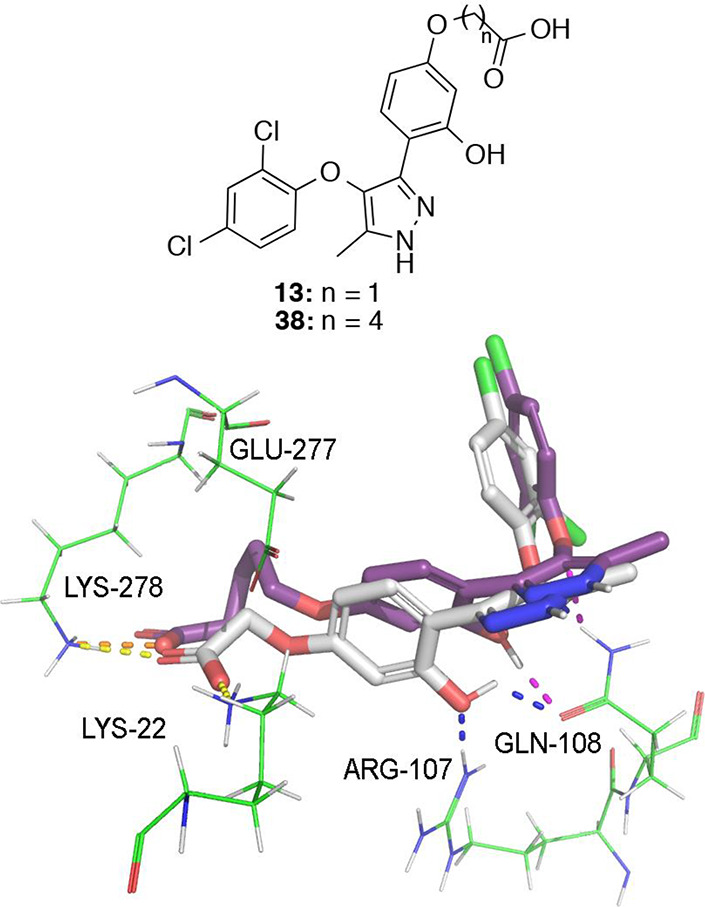
LPA_1_ (PDB ID 4Z35)-derived homology model of LPA_2_ in complex with compound **13** (in white) and compound **38** (in purple). Docking of compound **13** in the model suggests that the carboxylic acid of the compound is involved in two salt bridge interactions with lysine 22 and lysine 278; however, derivative **38** cannot form the salt bridge with lysine 22. Docking results also suggest that the phenolic hydroxy group of compound **13** establishes a hydrogen bond with arginine 107 and another one with glutamine 108, whereas derivative **38** can only establish hydrogen bonds with glutamine 108. For compound **13**, salt bridges are colored in yellow and hydrogen bonds in blue, whereas for derivative **38**, they are colored in orange and pink, respectively.

None of the synthesized compounds showed any activity as LPA_2_ agonists at 10 μM concentration and, in all cases, increasing the distance between the carboxylic acid group and the rest of the molecule resulted in decreased LPA_2_ antagonism activity (Table 2). The worst result was obtained for compound **38**, bearing the longest chain (*n* = 4) with an *E*_max_ value of 21% compared to the 84% of derivative **13**. This decrease in activity can be rationalized by the docking model between compound **38** and LPA_2_ (Figure 4), which shows that a key salt bridge interaction between the carboxylic acid group and lysine 22 can take place in compound **13** but not in derivative **38** due to the binding conformation induced by the four-unit spacer. In addition, a key hydrogen bond established between the phenolic hydroxy group of compound **13** and arginine 107 is missing in the binding of compound **38** to LPA_2_ (Figure 4).

**Table 2:**
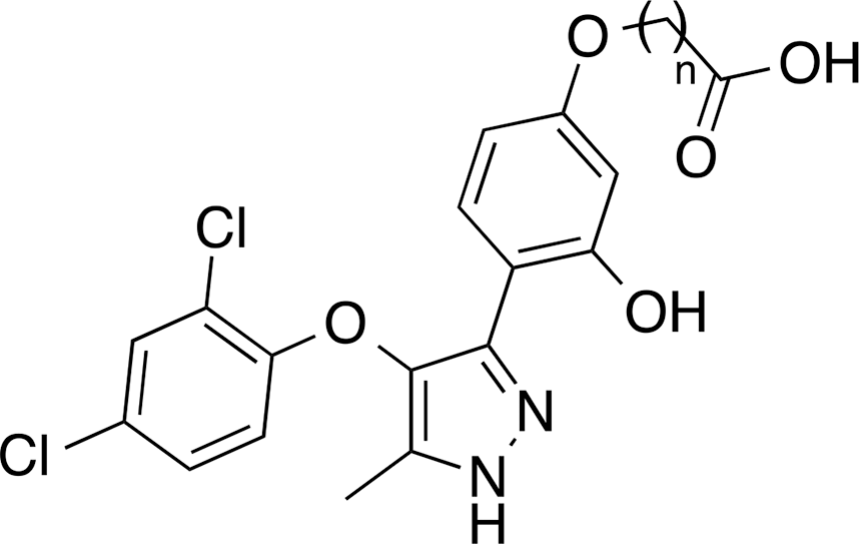
Antagonist activities of compounds **13**, **36**–**38**, and Ki16425 at LPA_1-3_

		*E*_max_ (%)^a^ [IC_50_ (μM)]^b^		
compd	*n*	LPA_1_	LPA_2_	LPA_3_
**13**	1	N.E.^c^	84 ± 3 [5.5 ± 0.7]	N.E.
**36**	2	N.E.	67 ± 7	N.E.
**37**	3	N.E.	43 ± 12	N.E.
**38**	4	N.E.	21 ± 8	N.E.
**Ki16425**		97 ± 4 [0.8 ± 0.2]	92 ± 3 [1.2 ± 0.6]	99 ± 3 [1.6 ± 0.5]

a *E*_max_ = maximum blockade effect of the activation induced by 10 μM of LPA (18:1, 1-oleoyl-*sn*-glycerol-3-phosphate) at a concentration of the compound under study of 10 μM.

b For *E*_max_ > 70%, IC_50_ values are expressed as mean ± s.e.m, from a minimum of two independent experiments, performed in triplicate.

c N.E., no effect was observed at the highest concentration of compound tested (10 μM).

**Scheme 4:**
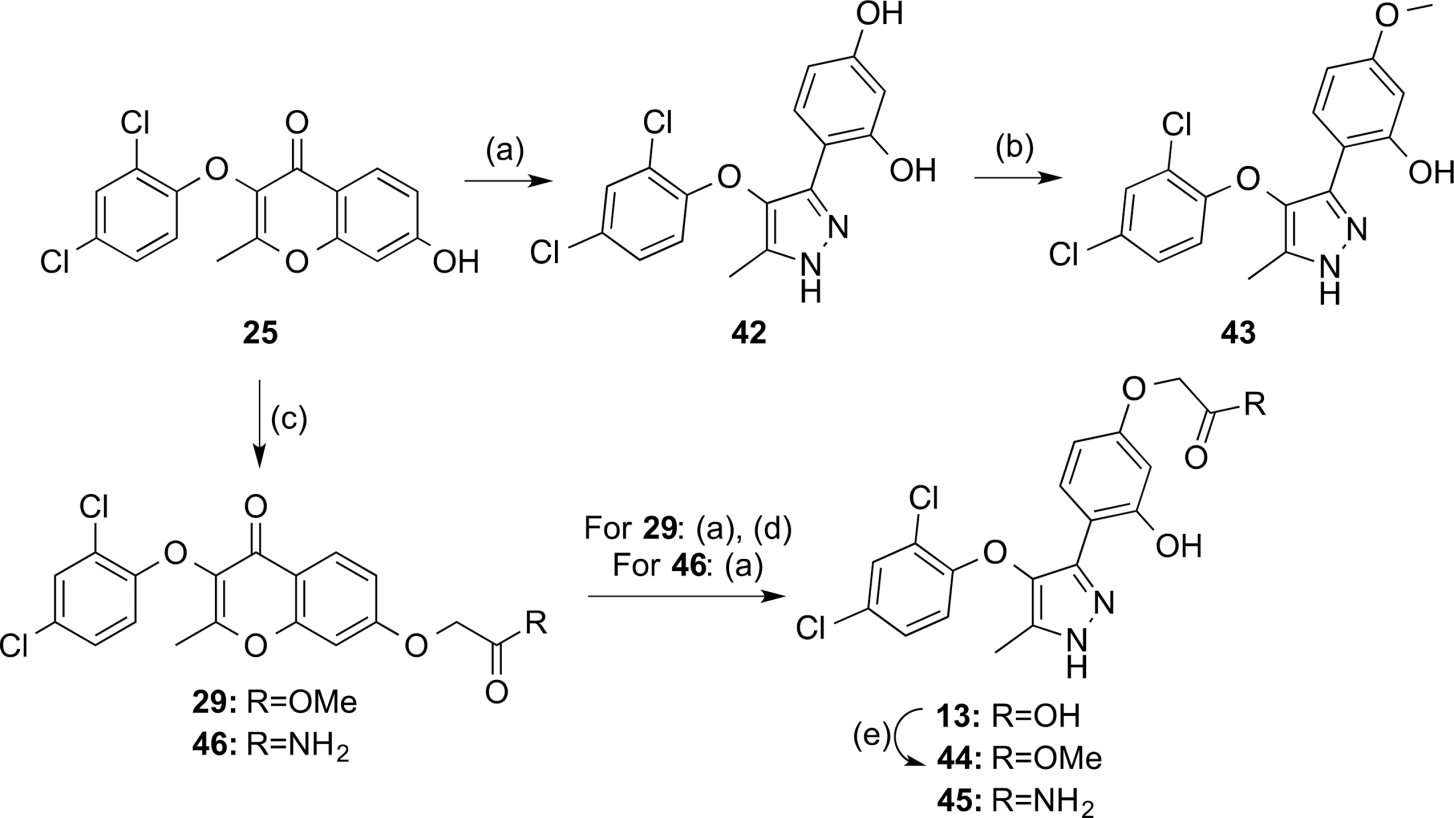
Synthesis of Compounds **42**–**45** Reagents and conditions: (a) 65% N_2_H_4_·H_2_O, EtOH, reflux, 30 min, 65–80%; (b) CH_3_I, K_2_CO_3_, acetone, 65 °C, 6 h, 20%; (c) methyl bromoacetate or bromoacetamide, K_2_CO_3_, acetone, reflux, 3 h, 51–67%; (d) 2 M NaOH, EtOH, reflux, 12 h, 90%; (e) CH_3_OH, cat. H_2_SO_4_, reflux, 16 h, 85%.

Further confirmation of the importance of the carboxylic acid interactions was obtained with the synthesis of compounds **42**–**45**, where the carboxylic acid moiety was replaced by hydroxy, methoxy, methyl ester or carboxamide groups, respectively. The synthesis of these compounds started from chromone **25**, which by reaction with hydrazine yielded pyrazole **42** that was further methylated to give **43**. Alternatively, *O*-alkylation of chromone **25** with methyl bromoacetate or bromoacetamide followed by pyrazole ring formation yielded compounds **44** and **45** (Scheme 4). Biological evaluation of all these compounds (Table 3) revealed that only the methyl ester derivative **44** showed a good activity value (*E*_max_ = 74 ± 7%; IC_50_ = 11.6 ± 0.4 μM). To further discard the existence of partial agonism, derivatives **42**–**45** were tested for their capacity to activate the LPA_2_ receptor and none of them induced any appreciable effect at a concentration of 10 μM.

**Table 3:**
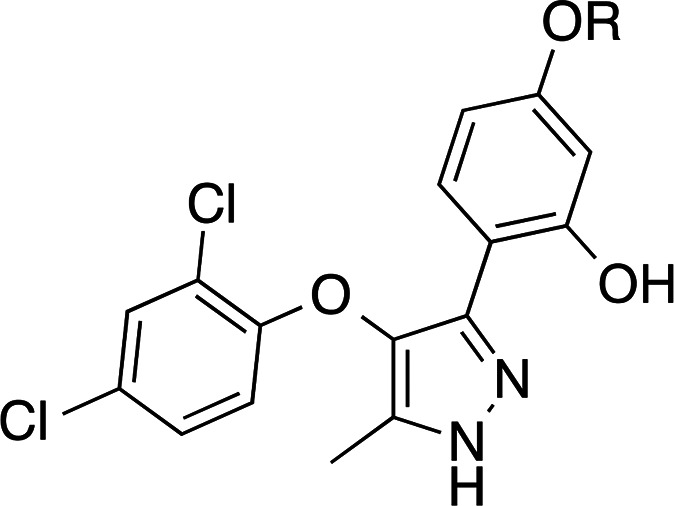

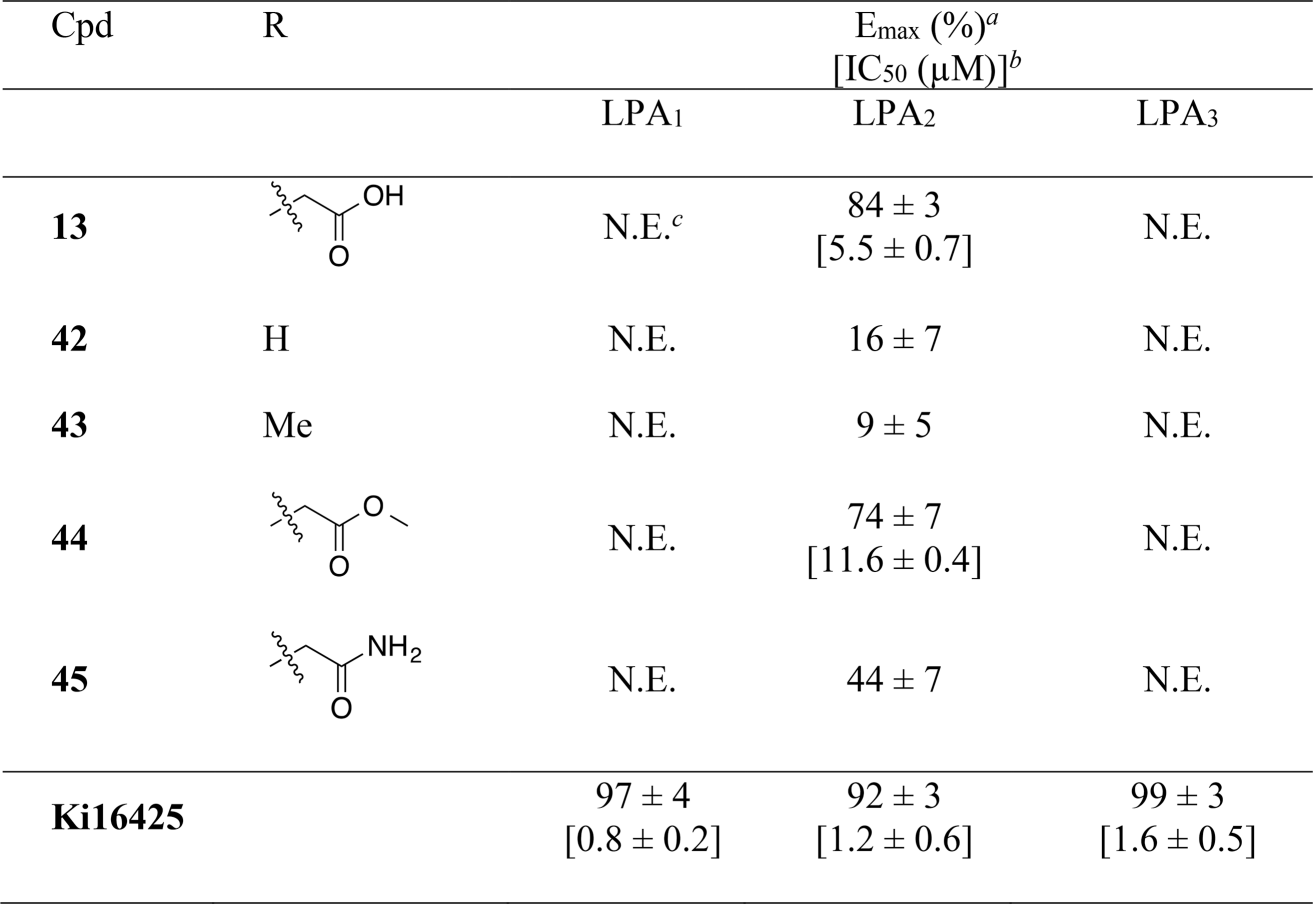
Antagonist Activities of Compounds **13**, **42–45**, and Ki16425 at LPA_1-3_

a *E*_max_ = maximum blockade effect of the activation induced by 10 μM of LPA (18:1, 1-oleoyl-*sn*-glycerol-3-phosphate) at a concentration of the compound under study of 10 μM.

b For *E*_max_ > 70%, IC_50_ values are expressed as mean ± s.e.m, from a minimum of two independent experiments, performed in triplicate.

c N.E., no effect was observed at the highest concentration of compound tested (10 μM).

**Scheme 5:**
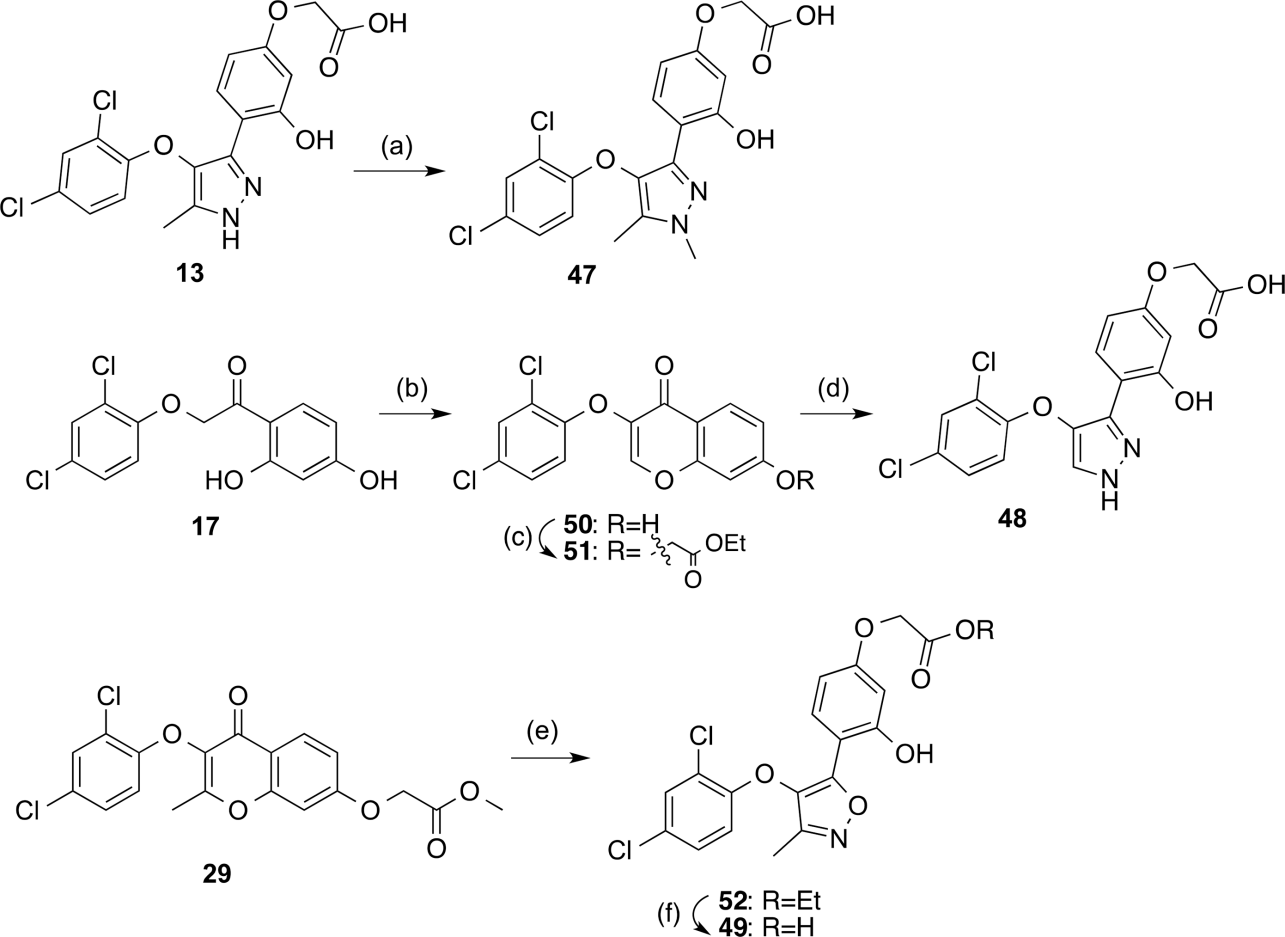
Synthesis of Compounds **47-49** Reagents and conditions: (a) CH_3_I, NaH, THF, rt, 16 h, 31%; (b) CH_3_SO_2_Cl, BF_3_·Et_2_O, DMF, 100 °C, 1.5 h, 94%; (c) ethyl bromoacetate, K_2_CO_3_, acetone, reflux, 3 h, 91%; (d) (i) 65% N_2_H_4_·H_2_O, EtOH, reflux, 30 min, 99%; (ii) 2 M NaOH, EtOH, reflux, 12 h, 58%; (e) (i) NH_2_OH·HCl, pyridine, EtOH, 85 °C, 12 h; (ii) *p-*toluenesulfonic acid, EtOH, 78 °C, 5 h, 40%; (f) 1 M NaOH, 1,4-dioxane, 60 °C, 16 h, 91%.

We next studied the effect of changes in the pyrazole ring. Specifically, we introduced an *N*-methyl group (compound **47**), removed the methyl group located at position 5 of the heterocycle (compound **48**), and replaced the pyrazole by an isoxazole ring (derivative **49**). Direct methylation reaction of **13** provided *N*-methylated analogue **47** (Scheme 5), whereas the preparation of compound **48** started with the reaction of intermediate **17** with methanesulfonyl chloride, in the presence of the boron trifluoride diethyl etherate complex to obtain the corresponding hydroxychromone **50**. Alkylation of this intermediate with ethyl bromoacetate and reaction with hydrazine, afforded desired pyrazole **48** (Scheme 5). Finally, isoxazole analogue **49** was obtained from chromone **29** using hydroxylamine (Scheme 5). Biological evaluation of the compounds (Table 4) revealed the importance of the methyl group in position 5 of the pyrazole ring for the antagonist activity, since derivative **48** exhibited a moderate *E*_max_ value of 35%. With respect to derivatives **47** and **49**, they showed good activity at LPA_2_ but also a decrease in selectivity, since they display some antagonist character at LPA_3_ (Table 4). None of them showed any activity as LPA_2_ agonists at a concentration of 10 μM.

**Table 4:**
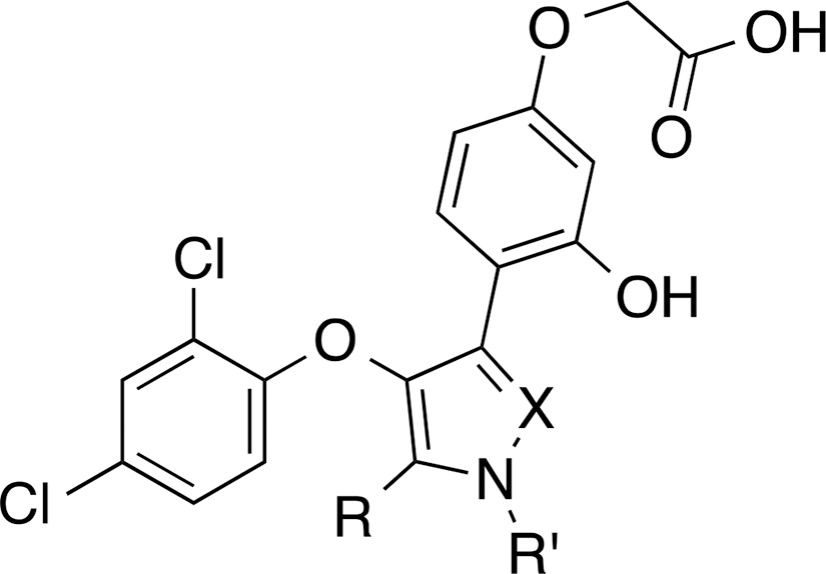
Antagonist Activities of Compounds **13, 47–49**, and Ki16425 at LPA_1-3_

				*E*_max_ (%)^a^ [IC_50_ (μM)]^b^		
compd	R	R′	X	LPA_1_	LPA_2_	LPA_3_
**13**	Me	H	N	N.E.^c^	84 ± 3 [5.5 ± 0.7]	N.E.
**47**	Me	Me	N	N.E.	73 ± 10	47 ± 7
**48**	H	H	N	N.E.	35 ± 8	N.E.
**49**	Me	H	O	N.E.	58 ± 3	71 ± 4
**Ki16425**				97 ± 4 [0.8 ± 0.2]	92 ± 3 [1.2 ± 0.6]	99 ± 3 [1.6 ± 0.5]

a *E*_max_ = maximum blockade effect of the activation induced by 10 μM LPA (18:1, 1-oleoyl-*sn*-glycerol-3-phosphate) at a concentration of the compound under study of 10 μM.

b For *E*_max_ > 70% and selectivity at LPA_2_ receptor, IC_50_ values are expressed as mean ± s.e.m, from a minimum of two independent experiments, performed in triplicate.

c N.E., no effect was observed at the highest concentration of compound tested (10 μM).

In sum, these results indicated that derivative **13** was the best compound identified so far. Hence, we studied its pharmacokinetic profile. First, we estimated the membrane permeability using the parallel artificial membrane permeability assay (PAMPA) and its metabolic stability in mouse and human liver microsomes (MLMs and HLMs, respectively). In these assays, compound **13** showed a moderate permeability value (P) of 0.11 × 10^–6^ cm/s, considering as reference values *P* < 1 × 10^–7^ cm/s for low permeable compounds and *P* > 1 × 10^–5^ cm/s for highly permeable molecules. The metabolic stability was also moderate, with a half-life (*t*_1/2_) of about 60 min in HLMs and 16 min in MLMs. Hence, it would be desirable to improve these parameters to obtain an optimized compound suitable for in vivo efficacy experiments.

### Optimization of Compound **13**

**Scheme 6:**
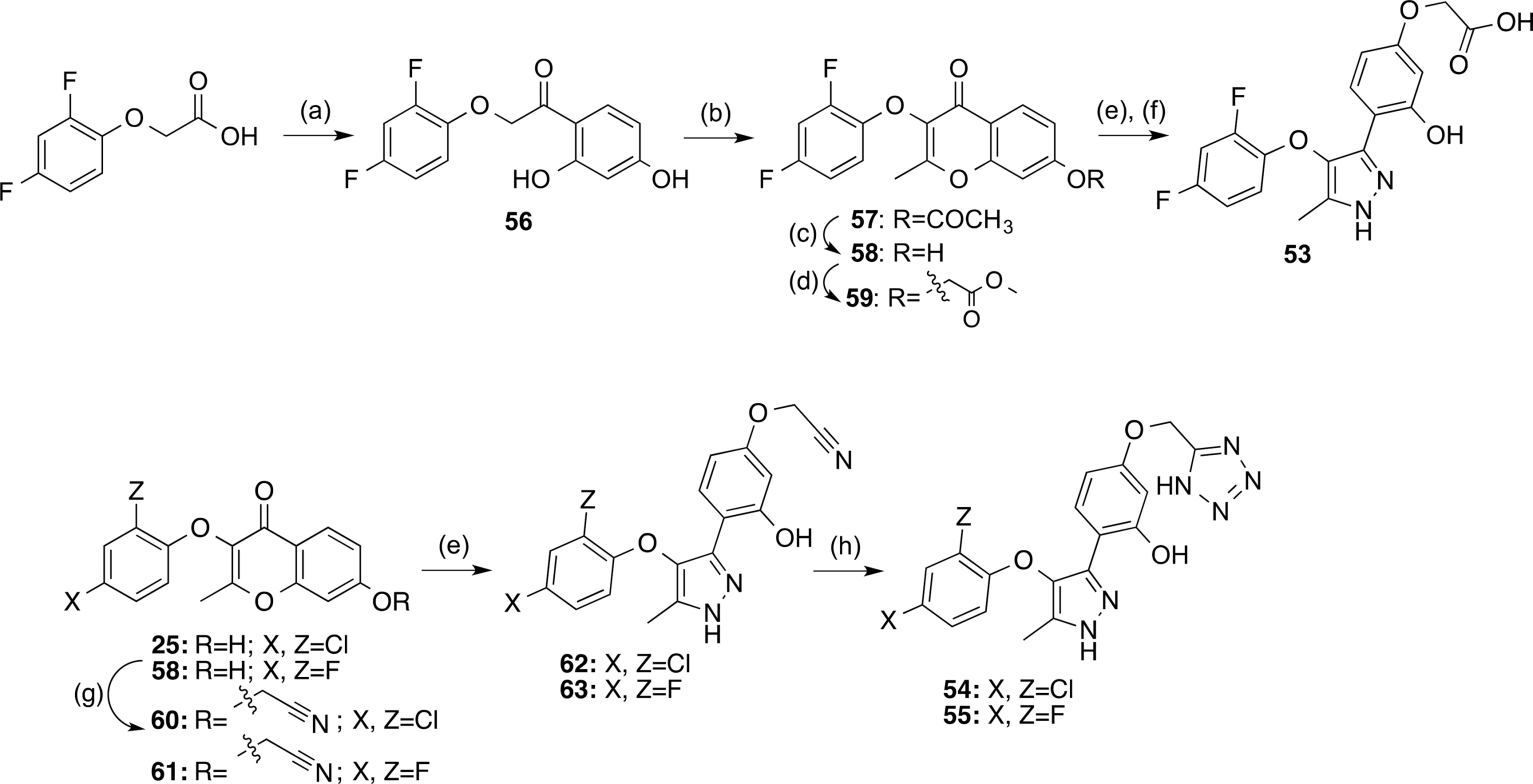
Synthesis of Compounds **53**–**55** Reagents and conditions: (a) (i) SOCl_2_, toluene, 110 °C, 16 h, 99%; (ii) resorcinol, BF_3_·Et_2_O, DCM, reflux, 4 h, 10%; (b) acetic anhydride, Et_3_N, NaOAc, 140 °C, 2.5 h, 71%; (c) conc. HCl, EtOH, reflux, 2 h, 99%; (d) methyl bromoacetate, K_2_CO_3_, acetone, reflux, 3 h, 99%; (e) 65% N_2_H_4_·H_2_O, EtOH, reflux, 30 min, 21–99%; (f) 2 M NaOH, EtOH, reflux, 12 h, 61%; (g) bromoacetonitrile, K_2_CO_3_, acetone, reflux, 3–5 h, 77–93%; (h) NaN_3_, NH_4_Cl, DMF, reflux, 16 h, 52–72%.

We initially addressed the optimization of derivative **13** with the replacement of chlorine atoms by fluorine in compound **53** (Scheme 6), as this change usually involves an improvement of the pharmacokinetic parameters.^20,21^ Also, considering that the free carboxylic acid could be responsible for the moderate permeability, it was replaced by the (bio)isostere tetrazole (compounds **54** and **55**, Scheme 6). Tetrazole is among the most commonly employed carboxylic acid isosteres^22^ because its planarity and acidity closely resemble those of carboxylic acids (p*K*_a_ = 4.5–4.9). In addition, tetrazolate anions are more lipophilic than the corresponding carboxylates and they exhibit slightly different electrostatic potential and charge distribution due to the delocalization of the negative charge over the five-membered ring system. Then, synthesis of difluorinated derivative **53** was carried out following a similar route to the one described for compound **13** but starting with 2,4-difluorophenoxyacetic acid (Scheme 6). With respect to the tetrazole derivatives **54** and **55**, they were prepared by alkylation of the intermediate chromones **25** and **58**, respectively, with 2-bromoacetonitrile followed by sequential treatment with hydrazine and sodium azide to build the corresponding pyrazole and tetrazole rings, respectively (Scheme 6).

Biological evaluation of compounds **53**–**55** indicated that derivative **54** showed the best results, being the most potent LPA_2_ antagonist, with an *E*_max_ of 90% and an IC_50_ value of 1.9 μM (Figure S2), values that are superior to the ones showed by its analogue **13** (Table 5). None of these compounds showed any agonist activity at LPA_2_ (see Figure S1 for the result obtained for compound **54**, representative of the rest of the compounds).

**Table 5:**
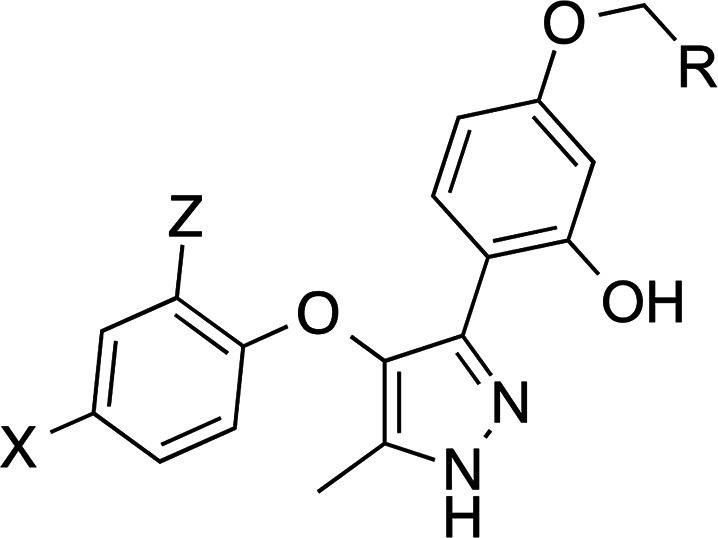

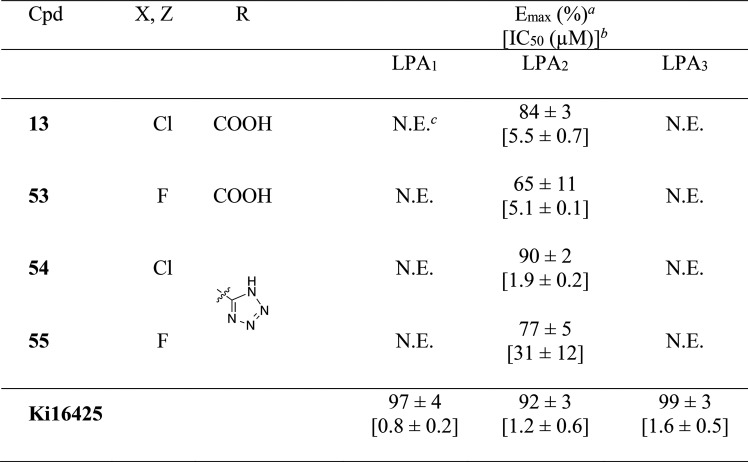
Antagonist Activities of Compounds **13**, **53**–**55**, and Ki16425 at LPA_1-3_

a *E*_max_ = maximum blockade effect of the activation induced by 10 μM of LPA (18:1, 1-oleoyl-*sn*-glycerol-3-phosphate) at a concentration of the compound under study of 10 μM.

b For *E*_max_ > 60%, IC_50_ values are expressed as mean ± s.e.m, from a minimum of two independent experiments, performed in triplicate.

c N.E., no effect was observed at the highest concentration of compound tested (10 μM).

**Figure 5:**
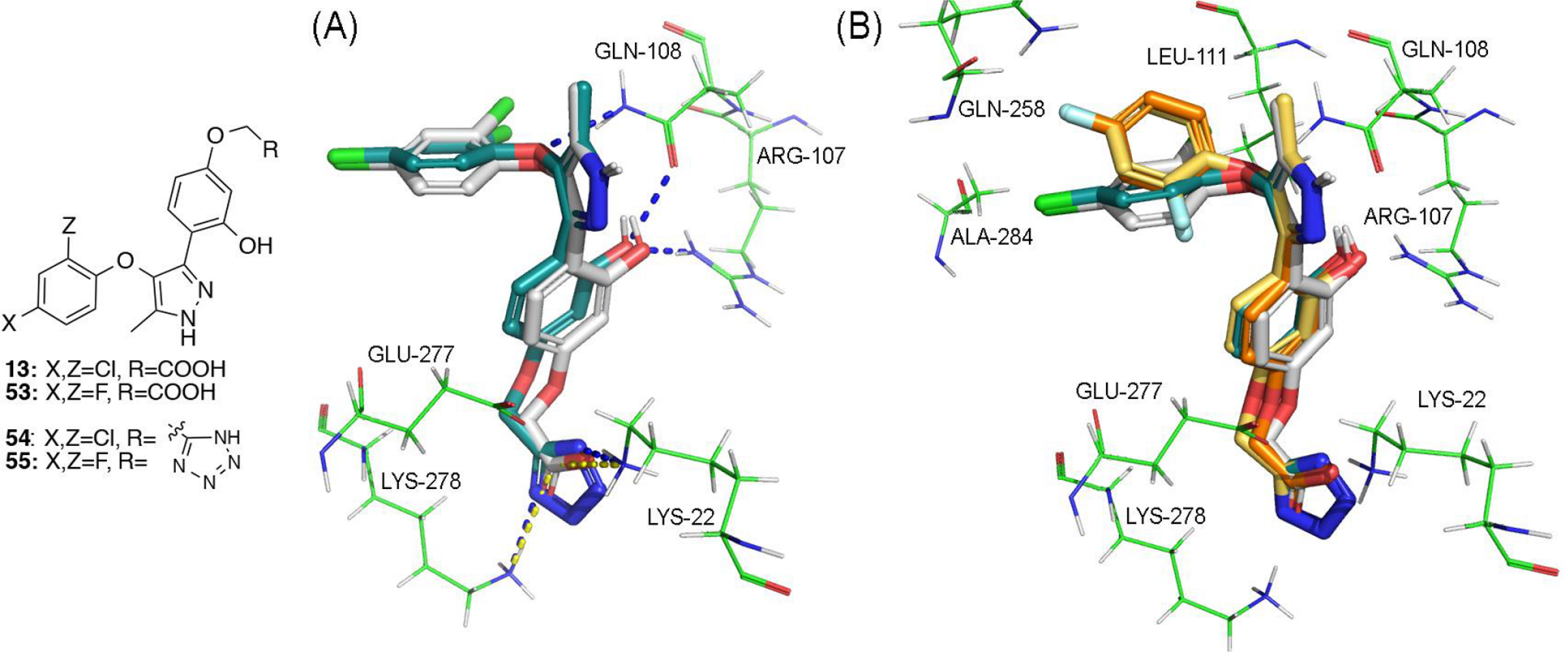
(A) LPA_1_ (PDB ID 4Z35)-derived homology model of LPA_2_ in complex with compounds **13** (in white) and **54** (in turquoise). The tetrazole moiety of compound **54** interacts in a similar manner to the carboxylic acid group of compound **13**. Thus, the carboxylic acid forms salt bridges with lysines 22 and 278 (in yellow) and the tetrazole ring establishes hydrogen bonds with these two residues (in blue). The rest key interactions are maintained in both compounds. (B) LPA_1_-derived homology model of LPA_2_ in complex with compounds **13** (in white), **53** (in orange), **54** (in turquoise), and **55** (in yellow). The dichlorophenoxy and difluorophenoxy moieties of compounds **13** and **53**–**55** lie in the same hydrophobic pocket. However, the substitution of chlorine for fluorine provokes a change in the orientation of the aromatic ring and prevents compounds **53** and **55** from reaching residues leucine 111 and alanine 284.

**Figure 6:**
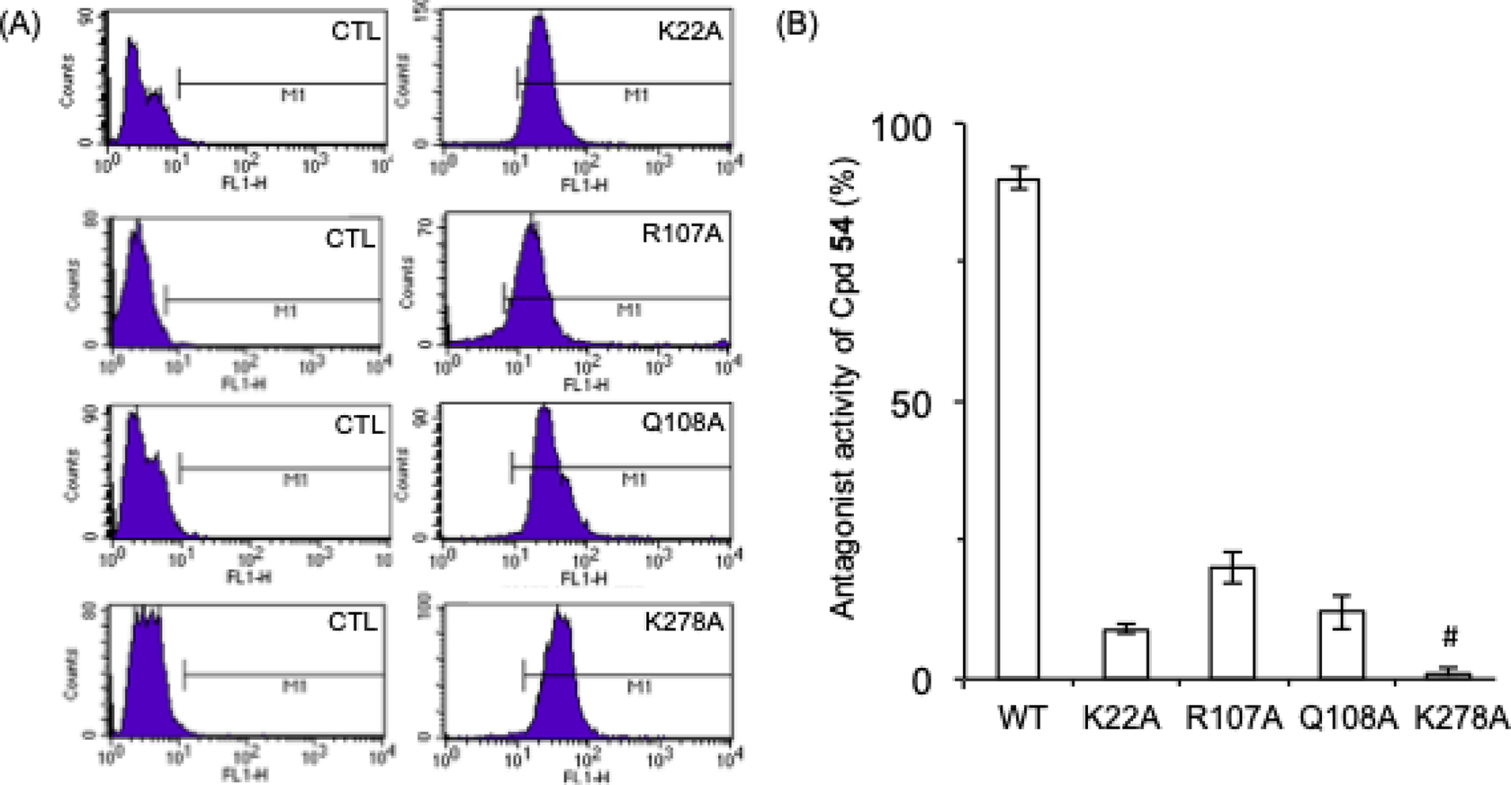
(A) Cell surface expression of each mutant LPA_2_ receptor was assessed by flow cytometry using an anti-HA antibody raised in mice followed by an antimouse antibody conjugated to Alexa 488 in McA-RH7777 cells transiently transfected with mock plasmid (CTL, control) or with plasmids containing the indicated LPA_2_ mutant receptor. (B) Capacity of compound **54** (10 μM) to block the activation induced by 10 μM of LPA (18:1, 1-oleoyl-*sn*-glycerol-3-phosphate) in the indicated mutant LPA_2_ receptor. Values are expressed as mean ± s.e.m, from two independent experiments performed in triplicate. Values obtained for the four point mutations have differences statistically significant (*p* < 0.01) with respect to the value obtained for the wild type (WT) receptor. ^#^Compound **54** behaved as an agonist of the LPA_2_ K278A mutant (able to induce 75 ± 5% activation at 10 μM, being the stimulation produced by 10 μM LPA normalized to 100%).

Docking studies of these compounds showed how the docking pose of compound **54** is very similar to that of compound **13** by replacing the carboxylate moiety with its tetrazole ring (Figure 5A). In fact, the tetrazole moiety of compound **54** perfectly reproduces the interactions of the carboxylic acid of compound **13**, substituting salt bridges for hydrogen bonds with lysines 22 and 278. With respect to the replacement of chlorine by fluorine in compounds **53** and **55**, the dichloro and difluorophenoxy moieties lie in the same hydrophobic pocket (Figure 5B). However, the substitution of chlorine by fluorine atoms provokes a change in the orientation of the aromatic ring and hinders the difluorophenoxy moiety from simultaneously reaching residues leucine 111 and alanine 284 as observed for compounds **13** and **54** through their two chlorine atoms (Figures 3 and 5B). To experimentally validate the proposed docking model, we carried out point mutation experiments to confirm the importance of the most relevant residues (lysines 22 and 278, arginine 107, and glutamine 108). Thus, we transfected McA-RH7777 cells with plasmids containing the corresponding N terminus HA-tagged LPA_2_ mutant (K22A, R107A, Q108A, or K278A). Transfection efficacy was assessed by flow cytometry using a primary antibody against HA and the appropriate fluorescent secondary antibody (Figure 6A) and the antagonist capacity of compound **54** was determined in cells transfected with each mutant. The obtained results indicate that replacement of any of the four amino acids (lysines 22 and 278, arginine 107 and glutamine 108) by alanine involved the lost of the antagonist activity of compound **54** (Figure 6B), thus confirming the importance of the proposed interactions. The data indicate that substitution of lysine 22, arginine 107, and glutamine 108 by alanine basically abolished the capacity of compound **54** to bind LPA_2_ receptor (since no significant agonist nor antagonist activity was observed in the mutant receptors). However, the exchange of lysine 278 by alanine completely switched the functional activity of the receptor since compound **54** behaved as an agonist in this mutant receptor. Overall, these data suggest that amino acids 22, 107, and 108 are important for binding, whereas lysine 278 seems to be involved in the functional activity of the receptor.

Noteworthy, compound **54** kept the receptor selectivity versus LPA_1_ and LPA_3_ (Table 5), so it emerged as an excellent candidate for in-depth pharmacological characterization.

### In-Depth Characterization of Compound **54**

First, we determined the membrane permeability using the PAMPA assay and the in vitro metabolic stability of the compound. The obtained results showed a good permeability value (*P* = 6.1 × 10^–6^ cm/s) and also increased stability in comparison with analog **13**, with *t*_1/2_ values of 50 ± 6 and 97 ± 15 min for MLMs and HLMs, respectively (Table 6).

**Table 6:** In Vitro Pharmacokinetic Profile of Compounds **13** and **54**

	stability (*t*_1/2_, min)^a^			
compd	MLMs	HLMs	*P* (cm/s)^b^	clog*P*^c^
**13**	16 ± 8	61 ± 10	0.1 × 10^–6^	4.14
**54**	50 ± 6	97 ± 15	6.1 × 10^–6^	3.29

a Data for stability in mouse and human liver microsomes (MLMs and HLMs, respectively) are expressed as the mean ± s.e.m. from five independent experiments performed in duplicate.

b *P* = permeability value; reference values consider *P* < 10^–7^ cm/s for low permeability compounds and *P* > 10^–5^ cm/s for highly permeable molecules.

c Values obtained with the ACDLabs *Percepta* software (version 6.0).

These in vitro values correlated with the results obtained in the in vivo pharmacokinetic (PK) study, which was carried out to determine the suitability of the compound to reach the CNS in therapeutically relevant doses. For this aim, compound **54** was administered intraperitoneally (i.p.) at a dose of 25 mg/kg. Then, at different postinjection times (between 0.5 and 4 h), plasma, brain, and spinal cord samples were taken and the levels of compound **54** were measured using high-performance liquid chromatography coupled to mass spectrometry (HPLC-MS). These experiments confirmed the presence of compound **54** at significant levels in spinal cord and brain, with the maximum levels reached at one hour postadministration. These data (Table 7) indicate that antagonist **54** can readily cross the blood brain barrier and it is therefore an excellent candidate to validate the role of LPA_2_ antagonism, at least as a proof of principle, in an in vivo model of SCI.

**Table 7:** In Vivo Levels of Compound **54** at Different Post-injection Times

	concentration of compound **54** (ng/mg tissue) after the indicated postinjection time (h)^a^			
sample	0.5	1	2	4
plasma	130 ± 50	41 ± 5	38 ± 1	ND
spinal cord	0.40 ± 0.06	3.3 ± 0.3	ND^b^	ND
brain	0.10 ± 0.02	28 ± 4	0.08 ± 0.01	ND

a Mice received a single injection of compound **54** (25 mg/kg, i.p.). Samples were taken at the indicated postinjection times and immediately frozen, and the levels of compound were then analyzed by HPLC-MS. Data are the means ± s.e.m. from three independent samples.

b ND, not detected.

In addition, the binding affinity of the compound for LPA_2_ was evaluated by means of a free solution assay-compensated interferometric reader (FSA-CIR) technique,^23−26^ showing a binding equilibrium constant (*K*_D_) value of 1.3 nM. As a positive control, LPA showed a *K*_D_ value of 6.7 nM to LPA_2_. The analogous assay carried out for LPA_4–6_ provided 10-fold selectivity versus LPA_6_ and >50-fold selectivity versus LPA_4_ and LPA_5_ (Figure S3), making compound **54** (UCM-14216) the most potent and selective LPA_2_ antagonist described so far.

### In Vivo Efficacy Study of Compound **54** in an SCI Mouse Model

**Figure 7:**
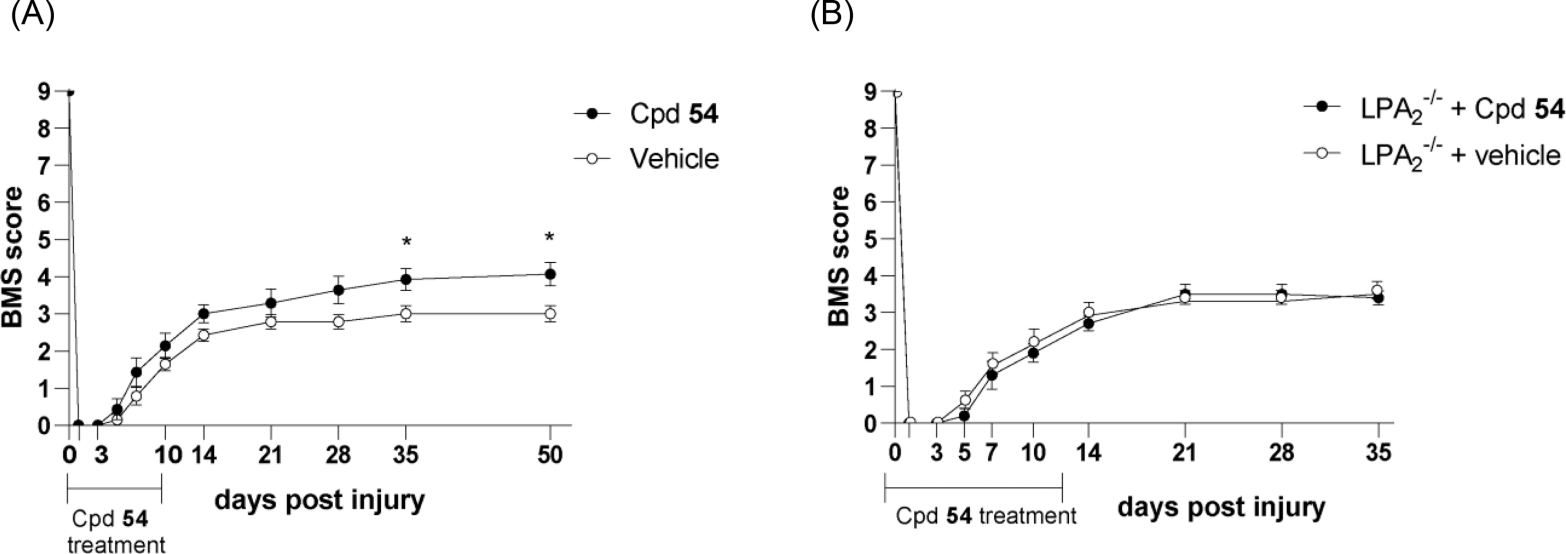
Compound **54** significantly improves locomotor recovery after SCI in LPA_2_ wild type mice but not in LPA_2_ deficient mice. Effect of i.p. injection of **54** (25 mg/kg) or vehicle on locomotor recovery in (A) C57bl/6 mice and (B) LPA_2_ deficient mice quantified using the 0 (absence of movement) to 9 (completely normal locomotor behavior) Basso Mouse Scale (BMS). Data are expressed as mean ± s.e.m. and correspond to seven animals per group in A and five animals per group in B. * *p* < 0.05 compared with vehicle-treated group (two-way repeated measures ANOVA with Bonferroni’s post hoc test for multiple comparisons).

Since LPA_2_ activation plays harmful actions after SCI, we finally assessed whether compound **54** protects against locomotor deficits in a spinal cord contusion injury model. It has been established that the LPA_2_ receptor is constitutively expressed at very low levels in spinal cord and its transcripts are up-regulated during the first days after injury, returning to basal levels by day 7.^13^ This suggests that LPA-LPA_2_ signaling in the injured spinal cord mainly occurs during the first week postinjury. Hence, we hypothesized that administration of compound **54** for 10 days could block LPA-LPA_2_ signaling in the injured spinal cord, and consequently, improve the outcome of SCI, as observed after genetic deletion of *Lpa*_*2*_.^13^ The in vivo PK study suggested that an i.p. dose of 25 mg/kg was enough to reach significant levels of the compound one hour after administration (3.3 ng/mg tissue are equivalent approximately to a concentration of 2 μM in the spinal cord considering the volume of the sections used in the study). It is conceivable that this concentration is even higher in the injured mice, as SCI results in increases permeability of the blood-spinal cord barrier.^27^ Then, this dose was selected as the minimal capable of potentially eliciting the sought biological effects and simultaneously avoiding side effects related with the use of higher concentrations. Accordingly, mice were treated daily with compound **54** (25 mg/kg, i.p.) starting at 1 h following lesion and subsequently for 10 consecutive days, and locomotor performance was assessed by using the Basso Mouse Scale (BMS). BMS is the gold standard test used for locomotor scoring after SCI in which two blinder observers score the mouse motor performance based on a nine-point scale.^28^ As shown in Figure 7A, mice treated with compound **54** displayed significant improvement in locomotor recovery after SCI. Bonferroni’s post hoc analysis revealed that motor skills were significantly enhanced in the injured mice that had been treated with compound **54** for 10 days at 25 mg/kg, from day 35 postinjury onward. At the end of the follow up (day 50 postinjury) mice treated with vehicle showed plantar placement of the hind paw but no weight-bearing stepping (BMS score 3.0 ± 0.2). In contrast, mice treated with compound **54** displayed occasional or frequent stepping (BMS of 4.1 ± 0.3). We do not discard that the therapeutic actions of the compound **54** could be enhanced with more frequent administration (i.e., twice a day), longer duration or greater dose of the compound.

Importantly, the observed locomotor improvement is largely mediated by the action of the compound at the LPA_2_ receptor, because administration of the same dose of compound to LPA_2_ null mice undergoing SCI did not induce any significant effect (Figure 7B). These results clearly validate the LPA_2_ receptor as a valuable therapeutic target for the treatment of SCI.

## Conclusions

In this work, we report the synthesis of the most potent and selective LPA_2_ antagonist identified to date, compound **54** (UCM-14216), with functional *E*_max_ and IC_50_ values of 90% and 1.9 μM, respectively, and a *K*_D_ value of 1.3 nM at LPA_2_ and functional selectivity against other LPA receptors (LPA_1,3–6_). In addition, compound **54** has a good pharmacokinetic profile both in vitro and in vivo, reaching pharmacologically relevant levels in the CNS, where the site of action is located. Furthermore, it shows efficacy in an acute in vivo mouse model of SCI being inactive in LPA_2_ knockout mice undergoing the same model, thus supporting the involvement of LPA_2_ in the secondary damage that follows SCI. SCI mainly affects to young and otherwise healthy adults, who suffer from a lack of efficacious treatments. Current treatments are generally palliative, limited to analgesic and anti-inflammatory drugs, underscoring high medical need for new pharmacological strategies that might be accessed by LPA_2_ antagonists to ameliorate SCI physiopathology and improve neurological outcomes. Further study of UCM-14216 and other related compounds could provide novel approaches to treat SCI and possibly other traumatic CNS injuries.

## Experimental Section

### Synthesis

Unless stated otherwise, starting materials, reagents, and solvents were purchased as high-grade commercial products from Sigma-Aldrich, Alfa Aesar, Acros, Fluka, Panreac or Scharlab, and were used without further purification. Dichloromethane (DCM) and tetrahydrofurane (THF) were dried using a Pure Solv Micro 100 Liter solvent purification system. Triethylamine and pyridine were dried over KOH and distilled prior to its use. All nonaqueous reactions were performed under an argon atmosphere in oven-dried glassware unless otherwise stated. MW irradiation reactions were carried out on a Biotage Initiatior 2.5 reactor, using Biotage vials sealed with an aluminum/Teflon crimp top, which can be exposed to a maximum of 250 °C and 20 bar internal pressure.

Analytical thin-layer chromatography (TLC) was run on Supelco silica gel plates (silica gel 60 F_254_) with detection by UV light (254 nm) and 5% ninhydrin solution in ethanol or 10% phosphomolybdic acid solution in ethanol. Products were purified by flash chromatography on glass columns using silica gel (60 Å pore size, 230–400 mesh particle size from Supelco) or using a Varian 971-FP system with cartridges of silica gel (Varian, 50 μm size particle).

All compounds were obtained as oils, except for those whose melting points (mp) are indicated, which were solids. Mp values were determined on a Stuart Scientific electrothermal apparatus. Infrared (IR) spectra were measured on a Bruker Tensor 27 instrument equipped with a Specac ATR accessory of 5200–650 cm^–1^ transmission range; frequencies (ν) are expressed in cm^–1^.

Nuclear magnetic resonance (NMR) spectra were recorded at rt on a Bruker Avance III 700 MHz (^1^H, 700 MHz; ^13^C, 175 MHz), Bruker Avance 500 MHz (^1^H, 500 MHz; ^13^C, 125 MHz) or Bruker DPX 300 MHz (^1^H, 300 MHz; ^13^C, 75 MHz) instrument at the Universidad Complutense de Madrid (UCM) NMR core facility. ^19^F-NMR spectra were recorded on a Bruker DPX 300 MHz. Chemical shifts (δ) are expressed in parts per million relative to the residual solvent peak for ^1^H and ^13^C nucleus (CDCl_3_: δ_H_ = 7.26, δ_C_ = 77.2; MeOH-*d*_4_: δ_H_ = 3.31, δ_C_ = 49.0; DMSO-*d*_6_: δ_H_ = 2.50, δ_C_ = 39.5), and coupling constants (*J*) are in hertz (Hz). The following abbreviations are used to describe peak patterns when appropriate: s (singlet), d (doublet), t (triplet), q (quadruplet), m (multiplet), br (broad), and app (apparent). 2D NMR experiments (COSY, HMQC, and HMBC) of representative compounds were carried out to assign protons and carbons of new structures; for those carbons displaying very broad signals in ^13^C NMR spectra, the corresponding chemical shifts were established by their correlation peaks in HSQC and HMBC spectra (Figure S4 shows the numbered structures used in the structural characterization by NMR of all final compounds). High-resolution mass spectrometry (HRMS) was carried out on a FTMS Bruker APEX Q IV spectrometer in electrospray ionization (ESI) or matrix-assisted laser desorption ionization (MALDI) mode at UCM’s mass spectrometry facilities.

For all final compounds, purity was determined by HPLC-MS and satisfactory chromatograms confirmed a purity of at least 95%. HPLC-MS analysis was performed using an Agilent 1200LC-MSD VL instrument. LC separation was achieved with an Eclipse XDB-C_18_ (5 μm, 4.6 mm × 150 mm) or a Zorbax SB-C_3_ column (5 μm, 2.1 mm × 50.0 mm) together with a guard column (5 μm, 4.6 mm × 12.5 mm). Mobile phase consisted of A (95:5 water/acetonitrile) and B (5:95 water/acetonitrile) with 0.1% formic acid as solvent modifier. Gradients are indicated in Table S1. MS analysis was performed with an ESI source. The capillary voltage was set to 3.0 kV and the fragmentor voltage was set at 72 eV. The drying gas temperature was 350 °C, the drying gas flow was 10 L/min, and the nebulizer pressure was 20 psi. Spectra were acquired in positive or negative ionization mode from 100 to 1000 *m*/*z* and in UV-mode at four different wavelengths (210, 230, 254, and 280 nm).

#### General Procedure 1: Friedel–Crafts Acylation

(a) Preparation of the aryloxyacetyl chloride: to a solution of the corresponding aryloxyacetic acid (1 equiv.) in anhydrous toluene (5.5 mL/mmol) was added thionyl chloride (2.8 mL/mmol) and the reaction mixture was refluxed for 16 h. After this time, the excess of thionyl chloride and toluene were evaporated under reduced pressure, affording the corresponding aryloxyacetyl chloride in quantitative yield. (b) Friedel–Crafts acylation: to a cooled (0 °C) stirred solution of the corresponding freshly prepared aryloxyacetyl chloride (1 equiv.) and resorcinol (1.1 equiv.) in anhydrous DCM (1.5 mL/mmol), boron trifluoride diethyl etherate (1.3 mL/mmol) was added. The reaction was stirred at 0 °C for 10 min and then at 90 °C until starting material was consumed (TLC, 4–5 h). The reaction vessel was then cooled in an ice bath and the mixture poured into an excess of ice water. The aqueous phase was extracted with DCM (×2), and the combined organic layers were washed with brine, dried over Na_2_SO_4_, filtered and concentrated under reduced pressure. The residue was purified by flash chromatography to yield the corresponding 2,4-dihydroxyphenylethanones **6**, **14**–**17**, and **56**.

#### General Procedure 2: Synthesis of Chromones by Kostanecki–Robinson Acylation

A mixture of the corresponding 2,4-dihydroxyphenylethanone (1 equiv.), freshly distilled acetic anhydride (0.6 mL/mmol), triethylamine (0.8 mL/mmol), and anhydrous sodium acetate (2.4 equiv.) was stirred at 140 °C until the reaction was completed (TLC, 2–3 h). Afterward, cold water was added and the mixture was extracted with DCM (×2). The combined organic phases were washed with brine, dried over Na_2_SO_4_, filtered, and concentrated under reduced pressure, affording the corresponding acetoxychromones **7**, **18**–**21**, and **57**, which were used without further purification.

#### General Procedure 3: Hydrolysis of Acetoxychromone Derivatives

To a solution of the appropriate acetoxychromone (1 equiv.) in the minimum amount of absolute ethanol was added conc. HCl (0.6 mL/mmol), and the reaction was refluxed for 2 h. After cooling to rt, the mixture was diluted with ethyl acetate and washed with a saturated aqueous solution of NaHCO_3_ and brine. The organic phase was dried over Na_2_SO_4_, filtered, and concentrated under reduced pressure, affording the corresponding hydroxychromones **8**, **22**–**25**, and **58**, which were used without further purification.

#### General Procedure 4: Alkylation of Hydroxychromone Derivatives

To a solution of the corresponding hydroxychromone (1 equiv.) in anhydrous acetone (15 mL/mmol) was added K_2_CO_3_ (2 equiv), and the reaction mixture was refluxed for 30 min. Then, a solution of the appropriate bromoderivative (1.1–4.3 equiv.) in anhydrous acetone (1 mL/mmol) was added and the mixture was refluxed until consumption of starting material (TLC, 3–5 h). Next, cold water was added and acetone was removed under reduced pressure. The aqueous residue was extracted with DCM (×2), and the combined organic phases were washed with brine, dried with Na_2_SO_4_, filtered, and concentrated under reduced pressure. The residue was purified by flash chromatography to afford the corresponding alkylated chromones **5**, **9**, **26**–**29**, **40**, **41**, **46**, **51**, **59**, **60**, and **61**.

#### General Procedure 5: Synthesis of Pyrazole Derivatives by Reaction with Hydrazine

A solution of the corresponding chromone or enaminone (1 equiv.) in absolute ethanol (5 mL/mmol) at 40 °C was treated with a solution of hydrazine monohydrate (65%, 0.18 mL/mmol) in absolute ethanol (1.3 mL/mmol), and the mixture was refluxed until the reaction was completed (TLC, 0.5–2 h). After cooling to rt, the mixture was concentrated under reduced pressure and the residue was dissolved with ethyl acetate and acidified with 1 M HCl until pH 6. The aqueous phase was extracted with ethyl acetate (×2), and the combined organic layers were washed with brine, dried over Na_2_SO_4_, filtered, and concentrated under reduced pressure. The residue was purified by flash chromatography to yield the corresponding pure pyrazoles **33**, **42**, **45**, **62**, and **63**. For those chromones or enaminones bearing an ester group, the resulting hydrazide derivative was taken to next step (General Procedure 6) without further purification.

#### General Procedure 6: Hydrolysis of Hydrazide Derivatives

To a solution of the corresponding hydrazide obtained according to General Procedure 5 (1 equiv.) in the minimum amount of 96% ethanol was added 2 M NaOH (0.8 mL/mmol), and the reaction was refluxed for 12 h. After cooling to rt, the mixture was diluted with ethyl acetate and acidified with 1 M HCl until pH 6. The aqueous phase was extracted with ethyl acetate (×2) and the combined organic layers were washed with brine, dried over Na_2_SO_4_, filtered, and concentrated under reduced pressure. The residue was purified by flash chromatography to yield target carboxylic acid derivatives **2**, **3**, **10**–**13**, **36**–**38**, **48**, and **53**.

#### General Procedure 7: Synthesis of Tetrazole Derivatives

To a solution of the corresponding nitrile (1 equiv.) in anhydrous DMF (15 mL/mmol), NH_4_Cl (1.5 equiv.), and NaN_3_ (1.5 equiv.) were added, and the reaction was refluxed overnight. Then, the mixture was filtered to remove salts, and the resulting solution was acidified until pH 3 with 1 M HCl and extracted with ethyl acetate (×2). The combined organic phases were washed with a 1:1 mixture of water/brine, dried with Na_2_SO_4_, filtered, and concentrated under reduced pressure. The residue was purified by flash chromatography to afford the corresponding tetrazoles **54** and **55**.

##### [3-Hydroxy-4-(1*H*-pyrazol-3-yl)phenoxy]acetic Acid (**2**)

Following general procedures 5 and 6, pyrazole **2** was obtained from chromone **5** (29 mg, 0.12 mmol) in 98% yield. Chromatography: DCM to DCM/methanol, 8:2. Mp: 92–94 °C. *R*_f_: 0.79 (DCM/methanol, 7:3). IR (ATR): ν 3404 (OH, NH); 1623 (C=O). ^1^H NMR (DMSO-*d*_6_, 700 MHz): δ 4.35 (s, 2H, CH_2_); 6.41–6.43 (m, 2H, H_2_, H_6_); 6.71 (br s, 1H, H_4′_); 7.56 (d, *J* = 8.7, 1H, H_5_); 7.80 (br s, 1H, H_5′_); 11.05 (br s, 1H, OH); 13.13 (br s, 1H, NH). ^13^C NMR (DMSO-*d*_6_, 175 MHz): δ 66.6 (CH_2_); 101.3 (C_4′_); 102.0 (C_2_); 106.3 (C_6_); 110.0 (C_4_); 127.3 (C_5_); 130.0 (C_5′_); 150.3 (C_3′_); 156.3 (C_3_); 159.1 (C_1_); 171.5 (C=O). HRMS (ESI, *m*/*z*): calcd for C_11_H_9_N_2_O_4_ [M-H]^−^: 233.0568; found: 233.0559. HPLC (method B, t_R_, min): 10.97.

##### [3-Hydroxy-4-(5-methyl-4-phenoxy-1*H*-pyrazol-3-yl)phenoxy]acetic Acid (**3**)

Following general procedures 5 and 6, pyrazole **3** was obtained from chromone **9** (10 mg, 0.03 mmol) in 99% yield. Chromatography: DCM to DCM/methanol, 7:3. Mp: >180 °C (decomp.). *R*_f_: 0.92 (DCM/methanol, 7:3). IR (ATR): ν 3440 (OH, NH); 1636 (C=O). ^1^H NMR (methanol-*d*_4_, 700 MHz): δ 2.11 (s, 3H, CH_3_); 4.38 (s, 2H, CH_2_); 6.32 (dd, *J* = 8.7, 2.2, 1H, H_6_); 6.46 (d, *J* = 2.2, 1H, H_2_); 6.88–6.91 (m, 2H, H_2″_, H_6″_); 6.99 (t, *J* = 7.4, 1H, H_4″_); 7.26–7.29 (m, 2H, H_3″_, H_5″_); 7.56 (d, *J* = 8.8, 1H, H_5_). ^13^C NMR (methanol-*d*_4_, 175 MHz): δ 8.8 (CH_3_); 67.9 (CH_2_); 103.3 (C_2_); 107.0 (C_6_); 110.6 (C_4_); 115.9 (C_2″_, C_6″_); 123.2 (C_4″_); 128.7 (C_5_); 130.8 (C_3″_, C_5″_); 133.6 (C_4′_, C_5′_); 159.7 (C_3_); 160.6 (C_1_, C_1″_); 177.2 (C=O); C_3′_ not observed. HRMS (ESI, *m*/*z*): calcd for C_18_H_15_N_2_O_5_ [M-H]^−^: 339.0986; found: 339.0975. HPLC (method B, t_R_, min): 21.34.

##### {4-[4-(3-Chlorophenoxy)-5-methyl-1*H*-pyrazol-3-yl]-3-hydroxyphenoxy}acetic Acid (**10**)

Following the general procedures 5 and 6, pyrazole **10** was obtained from chromone **26** (49 mg, 0.64 mmol) in 67% yield. Chromatography: ethyl acetate to ethyl acetate/methanol, 8:2. Mp: >240 °C (decomp.). *R*_f_: 0.18 (ethyl acetate/methanol, 8:2). IR (ATR): ν 3346 (OH, NH); 1585 (C=O). ^1^H NMR (methanol-*d*_4_, 700 MHz): δ 2.12 (s, 3H, CH_3_); 4.42 (s, 2H, CH_2_); 6.35 (dd, *J* = 8.8, 2.5, 1H, H_6_); 6.47 (d, *J* = 2.5, 1H, H_2_); 6.83 (ddd, *J* = 8.4, 2.3, 0.6, 1H, H_6″_); 6.90 (t, *J* = 2.2, 1H, H_2″_); 7.00 (dd, *J* = 7.9, 1.5, 1H, H_4″_); 7.25 (t, *J* = 8.2, 1H, H_5″_); 7.50 (d, *J* = 8.7, 1H, H_5_). ^13^C NMR (methanol-*d*_4_, 175 MHz): δ 8.7 (CH_3_); 67.5 (CH_2_); 103.5 (C_2_); 107.1 (C_6_); 110.5 (C_4_); 114.5 (C_6″_); 116.4 (C_2″_); 123.4 (C_4″_); 128.7 (C_5_); 132.0 (C_5″_); 133.2 (C_4′_/C_5′_), 135.3 (C_4′_/C_5′_); 136.3 (C_3″_); 140.7 (C_3′_); 158.1 (C_3_); 160.5, 160.6 (C_1_, C_1″_); 176.6 (C=O). HRMS (ESI, *m*/*z*): calcd for C_18_H_14_ClN_2_O_5_ [M-H]^−^: 373.0597; found: 373.0577. HPLC (method B, t_R_, min): 14.21.

##### {4-[4-(4-Chlorophenoxy)-5-methyl-1*H*-pyrazol-3-yl]-3-hydroxyphenoxy}acetic Acid (**11**)

Following general procedures 5 and 6, pyrazole **11** was obtained from chromone **27** (14 mg, 0.04 mmol) in 99% yield. Chromatography: DCM to DCM/methanol, 8:2. Mp: 121–123 °C. *R*_f_: 0.85 (DCM/methanol, 7:3). IR (ATR): ν 1627 (C=O). ^1^H NMR (methanol-*d*_4_, 700 MHz): δ 2.22 (s, 3H, CH_3_); 4.69 (s, 2H, CH_2_); 6.42 (dd, *J* = 8.9, 2.5, 1H, H_6_); 6.51 (d, *J* = 2.5, 1H, H_2_); 6.96 (d, *J* = 9.0, 2H, H_2″_, H_6″_); 7.30 (d, *J* = 9.0, 2H, H_3″_, H_5″_); 7.62 (d, *J* = 8.8, 1H, H_5_). ^13^C NMR (methanol-*d*_4_, 175 MHz): δ 8.5 (CH_3_); 65.9 (CH_2_); 103.3 (C_2_); 107.3 (C_6_); 108.3 (C_4_); 117.7 (C_2″_, C_6″_); 128.9 (C_4″_); 130.3 (C_5_); 130.9 (C_3″_, C_5″_); 134.1, 137.3 (C_4′_, C_5′_); 138.3 (C_3′_); 157.6 (C_1″_); 158.1 (C_3_); 161.6 (C_1_); 171.0 (C=O). HRMS (ESI, *m*/*z*): calcd for C_18_H_14_ClN_2_O_5_ [M-H]^−^: 373.0591; found: 373.0577. HPLC (method B, t_R_, min): 23.78.

##### {4-[4-(3,4-Dichlorophenoxy)-5-methyl-1*H*-pyrazol-3-yl]-3-hydroxyphenoxy}acetic Acid (**12**)

Following general procedures 5 and 6, pyrazole **12** was obtained from chromone **28** (38 mg, 0.09 mmol) in 92% yield. Chromatography: ethyl acetate to ethyl acetate/methanol, 8:2. Mp: >240 °C (decomp.). *R*_f_: 0.10 (ethyl acetate/methanol, 9:1). IR (ATR): ν 3406 (OH, NH); 1586 (C=O); 1258, 1174 (C–O–C). ^1^H NMR (methanol-*d*_4_, 700 MHz): δ 2.13 (s, 3H, CH_3_); 4.41 (s, 2H, CH_2_); 6.37 (dd, *J* = 8.7, 2.5, 1H, H_6_); 6.47 (d, *J* = 2.5, 1H, H_2_); 6.86 (dd, *J* = 9.0, 3.0, 1H, H_6″_); 7.06 (d, *J* = 3.0, 1H, H_2″_); 7.41 (d, *J* = 9.0, 1H, H_5″_); 7.46 (d, *J* = 8.7, 1H, H_5_). ^13^C NMR (methanol-*d*_4_, 175 MHz): δ 8.6 (CH_3_); 67.6 (CH_2_); 103.5 (C_2_); 107.2 (C_6_); 110.3 (C_4_); 116.2 (C_6″_); 118.1 (C_2″_); 126.4 (C_4″_); 128.7 (C_5_); 132.4 (C_5″_); 133.1 (C_4′_/C_5′_); 134.1 (C_4′_/C_5′,_ C_3″_); 158.9 (C_3_, C_1″_); 160.8 (C_1_); 176.9 (C=O); C_3′_not observed. HRMS (ESI, *m*/*z*): calcd for C_18_H_13_Cl_2_N_2_O_5_ [M-H]^−^: 407.0207; found: 407.0198. HPLC (method B, t_R_, min): 15.86.

##### {4-[4-(2,4-Dichlorophenoxy)-5-methyl-1*H*-pyrazol-3-yl]-3-hydroxyphenoxy}acetic Acid (**13**)

Following general procedures 5 and 6, pyrazole **13** was obtained from chromone **29** (45 mg, 0.11 mmol) in 99% yield. Chromatography: DCM to DCM/methanol, 8:2. Mp: >196 °C (decomp.). *R*_f_: 0.93 (DCM/methanol/acetic acid, 7:3:0.01). IR (ATR): ν 3413 (OH, NH); 1619 (C=O); 1182 (C–O–C). ^1^H NMR (methanol-*d*_4_, 300 MHz): δ 2.14 (s, 3H, CH_3_); 4.60 (s, 2H, CH_2_); 6.36 (dd, *J* = 8.7, 2.5, 1H, H_6_); 6.47 (d, *J* = 2.5, 1H, H_2_); 6.68 (d, *J* = 8.9, 1H, H_6″_); 7.14 (dd, *J* = 8.9, 2.5, 1H, H_5″_); 7.49–7.53 (m, 2H, H_5_, H_3″_). ^13^C NMR (methanol-*d*_4_, 125 MHz): δ 8.7 (CH_3_); 66.5 (CH_2_); 103.5 (C_2_); 107.1 (C_6_); 110.6 (C_4_); 116.8 (C_6″_); 124.1 (C_2″_); 128.3 (C_4″_); 128.6 (C_5_); 129.1 (C_5″_); 131.1 (C_3″_); 133.2, 134.3 (C_4′_, C_5′_); 139.7 (C_3′_); 153.9 (C_1″_); 158.1 (C_3_); 160.4 (C_1_); 173.8 (C=O). HRMS (ESI, *m*/*z*): calcd for C_18_H_13_Cl_2_N_2_O_5_ [M-H]^−^: 407.0207; found: 407.0192. HPLC (method B, t_R_, min): 14.90.

##### 4-(2,4-Dichlorophenoxy)-3-(4-carboxymethoxyphenyl)-5-methyl-1*H*-pyrazole (**30**)

A mixture of pyrazole **35** (10 mg, 0.02 mmol, 1 equiv.) and 1 M NaOH (30 μL, 0.03 mmol, 1.5 equiv.) in 1,4-dioxane (1 mL) was stirred at 60° for 2 h. After cooling to rt, the mixture was treated with 1 M HCl until acidic pH and extracted with ethyl acetate (2×). The combined organic phases were washed with brine, dried over Na_2_SO_4_, filtered, and concentrated under reduced pressure. The crude was purified by flash chromatography (ethyl acetate/methanol, 8:2 to ethyl acetate/methanol, 7:3) to yield pure compound **30** in 99% yield. Mp: 158–160 °C. *R*_f_: 0.13 (ethyl acetate/methanol, 7:3). IR (ATR): ν 3321 (NH/OH); 1649 (C=O); 1020 (C–O–C). ^1^H NMR (methanol-*d*_4_, 700 MHz): δ 2.11 (s, 3H, CH_3_); 4.63 (s, 2H, CH_2_); 6.66 (d, *J* = 8.9, 1H, H_6″_); 6.92 (d, *J* = 9.0, 2H, H_2_, H_6_); 7.12 (dd, *J* = 8.9, 2.5, 1H, H_5″_); 7.50 (d, *J* = 2.5, 1H, H_3″_); 7.62 (d, *J* = 9.0, 2H, H_3_, H_5_). ^13^C NMR (methanol-*d*_4_, 175 MHz): δ 9.2 (CH_3_); 66.1 (CH_2_); 115.9 (C_2_, C_6_); 116.7 (C_6″_); 124.1 (C_2″_); 124.4 (C_4_); 128.2 (C_3_, C_5_); 128.3 (C_4″_); 129.1 (C_5″_); 131.1 (C_3″_); 133.7, 137.5 (C_4′_, C_5′_); 139.0 (C_3′_); 154.1 (C_1″_); 159.5 (C_1_); 173.0 (C=O). HRMS (ESI, *m*/*z*): calcd for C_18_H_13_Cl_2_N_2_O_4_ [M-H]^−^: 391.0258; found: 391.0247. HPLC (method A, t_R_, min): 17.53.

##### 3-{4-[4-(2,4-Dichlorophenoxy)-5-methyl-1*H*-pyrazol-3-yl]-3-hydroxyphenoxy}propanoic Acid (**36**)

Following general procedures 5 and 6, pyrazole **36** was obtained from chromone **39** (13 mg, 0.03 mmol) in 97% yield. Chromatography: ethyl acetate/methanol, 8:2. *R*_f_: 0.83 (ethyl acetate/methanol, 8:2). IR (ATR): ν 2955, 2941 (NH, OH); 1653 (C=O); 1223 (C–O–C). ^1^H NMR (methanol-*d*_4_, 700 MHz): δ 2.20 (s, 3H, CH_3_); 2.78 (t, *J* = 6.5, 2H, CH_2_CO); 4.22 (t, *J* = 6.5, 2H, OCH_2_); 6.36 (dd, *J* = 8.8, 2.6, 1H, H_6_); 6.53 (d, *J* = 2.6, 1H, H_2_); 6.59 (d, *J* = 8.9, 1H, H_6″_); 7.03 (dd, *J* = 8.9, 2.6, 1H, H_5″_); 7.45 (d, *J* = 2.5, 1H, H_3″_); 7.56 (d, *J* = 8.7, 1H, H_5_). ^13^C NMR (methanol-*d*_4_, 175 MHz): δ 8.6 (CH_3_); 34.5 (CH_2_CO); 63.4 (OCH_2_); 102.6 (C_2_); 106.8 (C_6_); 109.0 (C_4_); 115.7 (C_6″_); 123.6 (C_2″_); 127.6 (C_4″_); 127.7 (C_5_); 128.0 (C_5″_); 130.5 (C_3″_); 133.0, 135.0 (C_4′_, C_5′_); 140.0 (C_3′_); 152.4 (C_1″_); 158.0 (C_3_); 160.0 (C_1_); 174.0 (C=O). HRMS (ESI, *m*/*z*): calcd for C_19_H_15_Cl_2_N_2_O_5_ [M-H]^−^: 421.0364; found: 421.0370. HPLC (method B, t_R_, min): 23.38.

##### 4-{4-[4-(2,4-Dichlorophenoxy)-5-methyl-1*H*-pyrazol-3-yl]-3-hydroxyphenoxy}butanoic Acid (**37**)

Following general procedures 5 and 6, pyrazole **37** was obtained from chromone **40** (12 mg, 0.03 mmol) in 99% yield. Chromatography: ethyl acetate to ethyl acetate/methanol, 8:2. *R*_f_: 0.56 (ethyl acetate/methanol, 1:1). IR (ATR): ν 3431 (NH/OH); 1669 (C=O); 1263 (C–O–C). ^1^H NMR (methanol-*d*_4_, 700 MHz): δ 2.00–2.04 (m, 2H, CH_2_); 2.13 (s, 3H, CH_3_); 2.43 (t, *J* = 7.4, 2H, CH_2_CO); 3.96 (t, *J* = 6.3, 2H, OCH_2_); 6.35 (dd, *J* = 8.7, 2.6, 1H, H_6_); 6.45 (d, *J* = 2.5, 1H, H_2_); 6.69 (d, *J* = 8.9, 1H, H_6″_); 7.14 (dd, *J* = 8.9, 2.5, 1H, H_5″_); 7.48 (d, *J* = 8.8, 1H, H_5_); 7.52 (d, *J* = 2.5, 1H, H_3″_). ^13^C NMR (methanol-*d*_4_, 175 MHz): δ 7.9 (CH_3_); 26.1 (CH_2_); 31.7 (CH_2_CO); 68.1 (OCH_2_); 103.2 (C_2_); 107.1 (C_6_); 109.1 (C_4_); 116.8 (C_6″_); 124.1 (C_2″_); 128.3 (C_5_, C_4″_); 129.1 (C_5″_); 131.1 (C_3″_); 133.1, 134.2 (C_4′_, C_5′_); 154.0 (C_1″_); 158.2 (C_3_); 161.3 (C_1_); 178.1 (C=O); C_3′_ not observed. MS (ESI, *m*/*z*): 435.0, 437.1, 439.0 [M-H]^−^. HPLC (method B, t_R_, min): 23.33.

##### 5-{4-[4-(2,4-Dichlorophenoxy)-5-methyl-1*H*-pyrazol-3-yl]-3-hydroxyphenoxy}pentanoic Acid (**38**)

Following general procedures 5 and 6, pyrazole **38** was obtained from chromone **41** (140 mg, 0.30 mmol) in 86% yield. Chromatography: hexane/ethyl acetate 9:1 to hexane/ethyl acetate 1:1. R_f_: 0.64 (ethyl acetate/methanol, 8:2). IR (ATR): ν 2923, 2852 (NH, OH); 1703 (C=O); 1242, 1191 (C–O–C). ^1^H NMR (methanol-*d*_4_, 700 MHz): δ 1.74–1.78 (m, 4H, 2CH_2_); 2.13 (s, 3H, CH_3_); 2.35 (t, *J* = 6.7, 2H, COCH_2_); 3.94 (t, *J* = 5.5, 2H, OCH_2_); 6.34 (dd, *J* = 8.7, 2.6, 1H, H_6_); 6.44 (d, *J* = 2.5, 1H, H_2_); 6.68 (d, *J* = 8.9, 1H, H_6″_); 7.14 (dd, *J* = 8.9, 2.5, 1H, H_5″_); 7.48 (d, *J* = 8.8, 1H, H_5_); 7.52 (d, *J* = 2.5, 1H, H_3″_). ^13^C NMR (methanol-*d*_4_, 175 MHz): δ 8.3 (CH_3_); 22.8, 29.8 (2CH_2_); 34.7 (COCH_2_); 68.5 (OCH_2_); 103.2 (C_2_); 107.1 (C_6_); 109.8 (C_4_); 116.8 (C_6″_); 124.1 (C_2″_); 128.3 (C_5_); 128.6 (C_4″_); 129.1 (C_5″_); 131.1 (C_3″_); 133.2 (C_5′_); 135.5 (C_4′_); 140.4 (C_3′_); 154.0 (C_1″_); 156.0 (C_3_); 161.4 (C_1_); 177.5 (C=O). HRMS (ESI, *m*/*z*): calcd for C_21_H_19_Cl_2_N_2_O_5_ [M-H]^−^: 449.0749; found: 449.0741. HPLC (method B, t_R_, min): 18.34.

##### 4-[4-(2,4-Dichlorophenoxy)-5-methyl-1*H*-pyrazol-3-yl]benzene-1,3-diol (**42**)

Following general procedure 5, pyrazole **42** was obtained from chromone **25** (82 mg, 0.24 mmol) in 65% yield. Mp: 206–208 °C. *R*_f_: 0.23 (ethyl acetate/methanol, 6:4). IR (ATR): ν 3293 (OH, NH); 1250 (C–O–C). ^1^H NMR (CDCl_3_, 700 MHz): δ 2.12 (s, 3H, CH_3_); 6.21 (dd, *J* = 8.5, 2.1, 1H, H_6_); 6.34 (d, *J* = 2.0, 1H, H_2_); 6.66 (d, *J* = 8.9, 1H, H_6″_); 7.11 (dd, *J* = 8.9, 2.4, 1H, H_5″_); 7.40 (d, *J* = 8.6, 1H, H_5_); 7.50 (d, *J* = 2.4, 1H, H_3″_). ^13^C NMR (CDCl_3_, 175 MHz): δ 8.7 (CH_3_); 103.9 (C_2_); 108.0 (C_6_); 108.9 (C_4_); 116.8 (C_6″_); 124.1 (C_2″_); 128.3 (C_4″_); 128.6 (C_5_); 129.1 (C_5″_); 130.9 (C_4′_/C_5′_); 131.1 (C_3″_); 133.0 (C_4′_/C_5′_); 153.9 (C_3′_); 158.1 (C_1″_); 159.6 (C_1_, C_3_). HRMS (ESI, *m*/*z*): calcd for C_16_H_13_Cl_2_N_2_O_3_ [M + H]^+^: 351.0298; found: 351.0303. HPLC (method B, t_R_, min): 21.80.

##### 2-[4-(2,4-Dichlorophenoxy)-5-methyl-1*H*-pyrazol-3-yl]-5-methoxyphenol (**43**)

A mixture of pyrazole **42** (25 mg, 0.07 mmol, 1 equiv.), potassium carbonate (21 mg, 0.15 mmol, 2.1 equiv.) and iodomethane (10 μL, 0.13 mmol, 1.8 equiv.) in acetone (0.84 mL) was stirred at 65 °C for 6 h. After cooling to rt, the solvent was evaporated under reduced pressure and the residue was dissolved in ethyl acetate and washed with water and brine. The organic phase was dried over Na_2_SO_4_, filtered, and concentrated under reduced pressure. The crude was purified by flash chromatography (hexane to hexane/ethyl acetate, 7:3) to yield pure compound **43** in 20% yield. Mp: 220–222 °C. *R*_f_: 0.50 (ethyl acetate/methanol, 6:4). IR (ATR): ν 3352 (OH, NH); 1252 (C–O–C). ^1^H NMR (methanol-*d*_*4*_, 700 MHz): δ 2.15 (s, 3H, C_5′_CH_3_); 3.85 (s, 3H, OCH_3_); 6.18 (dd, *J* = 8.6, 2.5, 1H, H_4_); 6.31 (d, *J* = 2.4, 1H, H_6_); 6.68 (d, = 8.9, 1H, H_6″_); 7.14 (dd, *J* = 8.9, 2.5, 1H, H_5″_); 7.37 (d, *J* = 8.6, 1H, H_3_); 7.53 (d, *J* = 2.5, 1H, H_3″_). ^13^C NMR (methanol-*d*_4_, 175 MHz): δ 8.1 (C_5′_CH_3_); 52.1 (OCH_3_); 104.0 (C_6_); 107.9 (C_4_); 109.1 (C_2_); 116.8 (C_6″_); 124.1 (C_2″_); 128.35 (C_3_); 128.4 (C_4″_); 129.1 (C_5″_); 131.1 (C_3″_); 132.8, 133.3 (C_4′_, C_5′_); 141.6 (C_3′_); 153.9 (C_1″_); 158.4, 159.5 (C_1_, C_5_). HRMS (ESI, *m*/*z*): calcd for C_17_H_15_Cl_2_N_2_O_3_ [M + H]^+^: 365.0454; found: 365.0458. HPLC (method B, t_R_, min): 21.90.

##### Methyl {4-[4-(2,4-dichlorophenoxy)-5-methyl-1*H*-pyrazol-3-yl]-3-hydroxyphenoxy} Acetate (**44**)

To a solution of pyrazole **13** (293 mg, 0.94 mmol) in methanol (3.70 mL) was added conc. H_2_SO_4_ (31 μL), and the reaction was refluxed overnight. The mixture was diluted with ethyl acetate, washed with water and brine, dried over Na_2_SO_4_, filtered, and concentrated under reduced pressure. The crude was purified by flash chromatography (hexane/ethyl acetate, 9:1 to hexane/ethyl acetate, 7:3) to yield pure compound **44** in 85% yield. Mp: >210 °C (decomp.). *R*_f_: 0.98 (DCM/methanol/acetic acid, 8:2:0.1). IR (ATR): ν 3297 (OH, NH); 1634 (C=O); 1016 (C–O–C). ^1^H NMR (methanol-*d*_4,_ 500 MHz): δ 2.21 (s, 3H, C_5′_CH_3_); 3.77 (s, 3H, OCH_3_); 4.69 (s, 2H, CH_2_); 6.41 (dd, *J* = 8.7, 2.6, 1H, H_6_); 6.49 (d, *J* = 2.5, 1H, H_2_); 6.75 (d, *J* = 8.9, 1H, H_6″_); 7.17 (dd, *J* = 8.9, 2.5, 1H, H_5″_); 7.55 (d, *J* = 8.8, 1H, H_5_); 7.57 (d, *J* = 2.5, 1H, H_3″_). ^13^C NMR (methanol-*d*_4,_ 125 MHz): δ 8.5 (C_5′_CH_3_); 52.6 (OCH_3_); 65.9 (CH_2_); 103.4 (C_2_); 107.2 (C_6_); 108.8 (C_4_); 116.9 (C_6″_); 124.3 (C_2″_); 129.0 (C_4″_); 129.3 (C_5_); 129.7 (C_5″_); 131.3 (C_3″_); 133.6, 137.5 (C_4′,_ C_5′_); 142.2 (C_3′_); 153.3 (C_1″_); 158.2 (C_3_); 161.3 (C_1_); 171.0 (C=O). HRMS (ESI, *m*/*z*): calcd for C_19_H_15_Cl_2_N_2_O_5_ [M-H]^−^: 421.0363; found: 421.0343. HPLC (method B, t_R_, min): 26.27.

##### 2-{4-[4-(2,4-Dichlorophenoxy)-5-methyl-1*H*-pyrazol-3-yl]-3-hydroxyphenoxy}acetamide (**45**)

Following the general procedure 5, pyrazole **45** was obtained from chromone **46** (24 mg, 0.06 mmol) in 80% yield. *R*_f_: 0.50 (ethyl acetate/methanol, 9:1). IR (ATR): ν 3413 (OH, NH); 1619 (C=O); 1182 (C–O–C). ^1^H NMR (methanol-*d*_4_, 700 MHz): δ 2.14 (s, 3H, CH_3_); 4.44 (s, 2H, CH_2_); 6.42 (d, *J* = 8.6, 1H, H_6_); 6.53 (d, *J* = 2.5, 1H, H_2_); 6.68 (d, *J* = 8.9, 1H, H_6″_); 7.14 (dd, *J* = 8.9, 2.4, 1H, H_5″_); 7.52 (d, *J* = 8.7, 1H, H_5_); 7.52 (d, *J* = 2.4, 1H, H_3″_). ^13^C NMR (methanol-*d*_4,_ 175 MHz): δ 7.9 (CH_3_); 67.8 (CH_2_); 103.6 (C_2_); 107.1 (C_6_); 110.9 (C_4_); 116.7 (C_6″_); 124.1 (C_2″_); 128.4 (C_4″_, C_5_); 129.1 (C_5″_); 131.1 (C_3″_); 133.2, 136.3 (C_5′_, C_4′_); 142.5 (C_3′_); 153.9 (C_1″_); 158.3 (C_3_); 159.8 (C_1_); 173.9 (C=O). HRMS (ESI, *m*/*z*): calcd for C_18_H_14_Cl_2_N_3_O_4_ [M-H]^−^: 406.0440; found: 406.0356. HPLC (method B, t_R_, min): 15.39.

##### {4-[4-(2,4-Dichlorophenoxy)-1,5-dimethyl-1*H*-pyrazol-3-yl]-3-hydroxyphenoxy}acetic Acid (**47**)

To a solution of pyrazole **13** (66 mg, 0.16 mmol, 1 equiv.) in anhydrous THF (0.70 mL) was added sodium hydride (60% dispersion in mineral oil, 8 mg, 0.32 mmol, 2 equiv.) at 0 °C and the mixture was stirred for 3 h, allowing it to reach rt during this time. Next, iodomethane (20 μL, 0.16 mmol, 1 equiv.) was added and the reaction was stirred at rt for 16 h. After this time, 1 M HCl was added until neutral pH and the organic solvent was evaporated. The residue was extracted with ethyl acetate (3×). The combined organic phases were washed with brine, dried over Na_2_SO_4_, filtered, and concentrated under reduced pressure to obtain pure compound **47** in 31% yield. Chromatography: hexane/ethyl acetate, 4:6 to ethyl acetate. R_f_: 0.16 (ethyl acetate/methanol/acetic acid, 9:1:0.01). IR (ATR): ν 2929 (OH); 1629 (C=O); 1261 (C–O–C). ^1^H NMR (methanol-*d*_4_, 700 MHz): δ 2.16 (s, 3H, C_5′_CH_3_); 3.86 (s, 3H, NCH_3_); 4.49 (s, 2H, CH_2_); 6.34 (dd, *J* = 8.8, 2.5, 1H, H_6_); 6.46 (d, *J* = 2.5, 1H, H_2_); 6.67 (d, *J* = 8.9, 1H, H_6″_); 7.13 (dd, *J* = 8.9, 2.5, 1H, H_5″_); 7.46 (d, *J* = 2.5, 1H, H_5_); 7.53 (d, *J* = 8.7, 1H, H_3″_). ^13^C NMR (methanol-*d*_4_, 175 MHz): δ 8.2 (C_5′_CH_3_); 37.2 (NCH_3_); 67.2 (CH_2_); 103.5 (C_2_); 107.2 (C_6_); 110.5 (C_4_); 116.7 (C_6″_); 124.0 (C_2″_); 128.2 (C_4″_); 128.5 (C_5″_); 129.2 (C_5_); 131.2 (C_3″_); 133.0, 133.4 (C_5′_, C_4′_); 141.2 (C_3′_); 153.8 (C_1″_); 158.3 (C_3_); 160.6 (C_1_); 172.9 (C=O). HRMS (MALDI, *m*/*z*): calcd for C_19_H_16_Cl_2_N_2_O_5_ [M]^+^: 422.0436; found: 422.0433. HPLC (method B, t_R_, min): 16.60.

##### {4-[4-(2,4-Dichlorophenoxy)-1*H*-pyrazol-3-yl]-3-hydroxyphenoxy}acetic Acid (**48**)

Following general procedures 5 and 6, pyrazole **48** was obtained from chromone **51** (27 mg, 0.07 mmol) in 58% yield. Chromatography: ethyl acetate/methanol, 9:1 to ethyl acetate/methanol, 8:2. *R*_f_: 0.10 (ethyl acetate/methanol, 7:3). IR (ATR): ν 3187 (OH, NH); 1627 (C=O); 1226 (C–O–C). ^1^H NMR (methanol-*d*_4,_, 500 MHz): δ 4.42 (s, 2H, CH_2_); 6.39 (dd, *J* = 8.8, 2.5, 1H, H_6_); 6.49 (d, *J* = 2.5, 1H, H_2_); 6.84 (d, *J* = 8.9, 1H, H_6″_); 7.16 (dd, *J* = 8.9, 2.5, 1H, H_5″_); 7.50 (d, *J* = 2.5, 1H, H_3″_); 7.57 (d, *J* = 8.7, 1H, H_5_); 7.61 (s, 1H, H_5′_). ^13^C NMR (methanol-*d*_4_, 125 MHz): δ 67.8 (CH_2_); 103.5 (C_2_); 107.2 (C_6_); 110.1 (C_4_); 117.8 (C_6″_); 124.7 (C_2″_); 128.7 (C_4″_); 128.9 (C_5_, C_5′_); 129.1 (C_5″_); 131.1 (C_3″_); 136.6 (C_4′_); 154.4 (C_1″_); 157.9 (C_3_); 160.8 (C_1_); 176.5 (C=O); C_3′_ not observed. HRMS (ESI, *m*/*z*): calcd for C_17_H_11_Cl_2_N_2_O_5_ [M-H]^−^: 393.0050; found: 393.0017. HPLC (method B, t_R_, min): 21.11.

##### {4-[4-(2,4-Dichlorophenoxy)-3-methyl-1,2-oxazol-3-yl]-3-hydroxyphenoxy}acetic Acid (**49**)

To a solution of isoxazole **52** (7 mg, 0.02 mmol, 1 equiv.) in the minimum amount of 1,4-dioxane was added 1 M NaOH (0.20 mL, 0.20 mmol, 10 equiv.), and the reaction was heated at 60 °C overnight. After cooling to rt, the solvent was removed under reduced pressure. The residue was dissolved with ethyl acetate and washed with a 1 M HCl solution and brine, dried over Na_2_SO_4_, filtered, and concentrated under reduced pressure, affording the corresponding isoxazole **49** in 91% yield. Mp: >210 °C (decomp.). *R*_f_: 0.15 (ethyl acetate/methanol/acetic acid, 7:3:0.01). IR (ATR): ν 2919 (OH); 1729 (C=O); 1175 (C–O–C). ^1^H NMR (methanol-*d*_*4*_, 500 MHz): δ 2.13 (s, 3H, CH_3_); 4.63 (s, 2H, OCH_2_); 6.41 (d, *J* = 2.4, 1H, H_2_); 6.51 (dd, *J* = 8.7, 2.5, 1H, H_6_); 6.87 (d, *J* = 8.9, 1H, H_6″_); 7.17 (dd, *J* = 8.9, 2.5, 1H, H_5″_); 7.38 (d, *J* = 8.7, 1H, H_5_); 7.47 (dd, *J* = 2.5, 1H, H_3″_). ^13^C NMR (methanol-*d*_4_, 125 MHz): δ 9.2 (CH_3_); 65.9 (OCH_2_); 103.2 (C_2_); 107.3 (C_6_); 108.5 (C_4_); 117.6 (C_6″_); 124.4 (C_2″_); 128.7 (C_4″_); 128.8 (C_5″_); 131.0 (C_5_); 131.1 (C_3″_); 132.2 (C_4′_); 153.4 (C_1″_); 156.7 (C_5′_); 158.1, 158.9 (C_3_, C_3′_); 162.5 (C_1_); 172.4 (C=O). HRMS (MALDI, *m*/*z*): calcd for C_18_H_13_Cl_2_NO_6_ [M]^+^: 409.0120; found: 409.0127. HPLC (method B, t_R_, min): 11.48.

##### {4-[4-(2,4-Difluorophenoxy)-5-methyl-1*H*-pyrazol-3-yl]-3-hydroxyphenoxy}acetic Acid (**53**)

Following general procedures 5 and 6, compound **53** was obtained from chromone **59** (65 mg, 0.17 mmol) in 61% yield. Chromatography: ethyl acetate to ethyl acetate/methanol 8:2. Mp: >210 °C (decomp.). *R*_f_: 0.15 (ethyl acetate/methanol 8:2). IR (ATR): ν 3400 (OH); 1620 (C=O); 1246, 1190 (C–O–C). ^1^H NMR (methanol-*d*_4_, 700 MHz): δ 2.14 (s, 3H, CH_3_); 4.43 (s, 2H, CH_2_); 6.37 (dd, *J* = 9.0, 2.5, 1H, H_6_); 6.47 (d, *J* = 2.5, 1H, H_2_); 6.71 (td, *J* = 9.2, 5.3, 1H, H_6″_); 6.74–6.77 (m, 1H, H_5″_); 7.09 (ddd, *J* = 11.5, 9.0, 3.0, 1H, H_3″_); 7.53 (d, *J* = 8.5, 1H, H_5_). ^13^C NMR (methanol-*d*_4_, 175 MHz): δ 8.6 (CH_3_); 67.5 (CH_2_); 103.4 (C_2_); 106.0 (dd, *J* = 27.5, 22.0, C_3″_); 107.1 (C_6_); 110.5 (C_4_); 111.8 (dd, *J* = 23.0, 3.5, C_5″_); 117.2 (d, *J* = 9.5, C_6″_); 128.6 (C_5_); 133.5, 1135.7 (C_4′_, C_5′_); 140.5 (C_3′_); 143.9 (dd, *J* = 10.5, 3.0, C_1″_); 153.0 (dd, *J* = 248.5, 12.0, C_2″_); 158.1 (C_3_); 158.5 (dd, *J* = 241.5, 10.0, C_4″_); 160.7 (C_1_); 176.3 (C=O). HRMS (ESI, *m*/*z*): calcd for C_18_H_13_F_2_N_2_O_5_ [M-H]^−^: 375.0798; found: 375.0792. HPLC (method B, t_R_, min): 13.33.

##### 2-[4-(2,4-Dichlorophenoxy)-5-methyl-*1H*-pyrazol-3-yl]-5-[*1H*-tetrazol-5-yl)methoxy] Phenol (**54**)

Following general procedure 7, compound **54** was obtained from pyrazole **62** (262 mg, 0.70 mmol) in 72% yield. Chromatography: ethyl acetate/methanol, 9:1 to ethyl acetate/methanol, 6:4. Mp: 180–182 °C. *R*_f_: 0.19 (ethyl acetate/methanol, 8:2). IR (ATR): ν 2921 (NH, OH); 1238 (C–O–C). ^1^H NMR (methanol-*d*_4_, 300 MHz): δ 2.14 (s, 3H, CH_3_); 5.40 (s, 2H, CH_2_); 6.49 (dd, *J* = 8.7, 2.6, 1H, H_4_); 6.59 (d, *J* = 2.6, 1H, H_6_); 6.68 (d, *J* = 8.9, 1H, H_6″_); 7.14 (dd, *J* = 8.9, 2.5, 1H, H_5″_); 7.52–7.56 (m, 2H, H_3_, H_3″_). ^13^C NMR (methanol-*d*_4*,*_ 75 MHz): δ 8.5 (CH_3_); 60.8 (CH_2_); 103.7 (C_6_); 107.0 (C_4_); 111.4 (C_2_); 116.8 (C_6″_); 124.1 (C_2″_); 128.4 (C_4″_); 128.8 (C_5″_); 129.1 (C_3_); 131.1 (C_3″_); 133.3, 134.9 (C_4′_, C_5′_); 140.2 (C_3′_); 153.9 (C_1″_); 156.9 (C_tetrazole_); 158.3 (C_1_); 159.8 (C_5_). HRMS (MALDI, *m*/*z*): calcd for C_18_H_15_Cl_2_N_6_O_3_ [M + H]^+^: 433.0504; found: 433.0506. HPLC (method C, t_R_, min): 10.38.

##### 2-[4-(2,4-Difluorophenoxy)-5-methyl-*1H*-pyrazol-3-yl]-5-[*1H*-tetrazol-5-yl)methoxy] Phenol (**55**)

Following general procedure 7, compound **55** was obtained from pyrazole **63** (344 mg, 0.96 mmol) in 52% yield. Chromatography: ethyl acetate/methanol, 9:1 to ethyl acetate/methanol, 6:4. Mp: >210 °C (decomp.). *R*_f_: 0.47 (hexane/ethyl acetate, 4:6). IR (ATR): ν 2931 (OH, NH); 1252, 1190 (C–O–C). ^1^H NMR (methanol-*d*_*4*_, 300 MHz): δ 2.15 (s, 3H, CH_3_); 5.40 (s, 2H, OCH_2_); 6.51 (dd, *J* = 8.7, 2.6, 1H, H_4_); 6.59 (d, *J* = 2.6, 1H, H_6_); 6.72–6.77 (m, 2H, H_5″_, H_6″_); 7.10 (td, *J* = 9.5, 8.3, 2.5, 1H, H_3″_); 7.60 (d, *J* = 8.6, 1H, H_3_). ^13^C NMR (methanol-*d*_4*,*_ 75 MHz): δ 8.5 (CH_3_); 60.8 (OCH_2_); 103.7 (C_6_); 106.0 (dd, *J* = 27.5, 22.0, C_3″_); 107.0 (C_4_); 110.4 (C_2_); 111.7 (dd, *J* = 26.4, 3.3, C_5″_); 117.2 (dd, *J* = 9.5, 1.9, C_6″_); 128.8 (C_3_); 133.7, 134.6 (C_4′_, C_5′_); 141.2 (C_3′_); 143.8 (dd, *J* = 11.1, 3.2, C_1″_); 153.1 (dd, *J* = 248.7, 12.2, C_2″_); 155.3 (C_tetrazole_); 158.3 (C_1_); 158.6 (dd, *J* = 241.8, 10.2, C_4″_); 159.7 (C_5_). ^19^F-NMR (methanol-*d*_4_, 282 MHz): −132.5, −120.5. HRMS (ESI, *m*/*z*): calcd for C_18_H_15_F_2_N_6_O_3_ [M + H]^+^: 401.1095; found: 401.1105. HPLC (method C, t_R_, min): 9.20.

### Evaluation of Receptor Activation by Ca^2+^ Mobilization Assay

Cells stably expressing the corresponding LPA_1–3_ receptor were grown as described previously.^18^ Changes in intracellular calcium levels were measured by using the fluorescent calcium sensitive dye Fluo-4 NW (Invitrogen). RH7777 cells or B103 cells were plated on poly-d-lysine or collagen coated, respectively, black-wall clear-bottom 96-well plates (Corning) at a density of 50 000 cells/well and cultured overnight. The culture medium was then replaced with 100 μL of Fluo-4 NW dye loading solution containing 2.5 μM of probenecid and incubated for 30 min at 37 °C followed by an additional 30 min at rt. Then, 20 μL of the test compound from a 6× stock solution in assay buffer were added and fluorescence was measured during 120 s after which 10 μM of LPA (18:1, 1-oleoyl-*sn*-glycerol-3-phosphate) was added and wells were monitored for additional 120 s. Fluorescence changes were registered in a FluoStar Optima instrument (BMG Labtech) at 525 nm using an excitation wavelength of 494 nm. Ca^2+^ transient increase was quantified by calculating the difference between maximum (stimulation with LPA 10 μM) and baseline values for each well, and antagonist activity was quantified by determining the percentage of the signal suppression caused by the compound under study with respect to the Ca^2+^ increase induced by LPA (which was considered 100%). As positive controls, 10 μM LPA and 10 μM ionomycin were included in every experiment. At this concentration, LPA induced a response about 30–33% of the one shown by ionomycin, which is in agreement with previously described results.^29^ The data presented are from two to four independent experiments carried out in triplicate or quadruplicate. Dose–response curves were generated and IC_50_ values calculated by nonlinear regression analysis using *Prism* software version 5 (GraphPad Software Inc., San Diego, CA, USA).

The agonist activity at LPA_2_ receptors was determined at 10 μM concentration for all final compounds as previously described.^18^

### Binding Affinity at LPA_4–6_

A free solution assay,^23,24^ where the lysophosphatidic acid receptor (LPA_2, 4–6_) containing nanovesicles (of 110–130 nm size as measured by dynamic light scattering) and compound under study are freely moving into solution was prepared to determine the equilibrium binding constants (*K*_D_) in a native environment of the binding partners (ligand/compound–receptor). The assay was analyzed using a benchtop Compensated Interferometric Reader (CIR) that measured the light refractive index (ΔRI) change from binding-induced conformational and/or hydration changes produced by real time binding events in a sample (receptor containing nanovesicles plus compound) and compared to a nonbinding reference (RI matched buffer plus compound).^30^ Finally, the interferometric signal from vector nanovesicles binding (nonspecific) to compound was subtracted from the LPA_2,4–6_ containing nanovesicles binding (total) to compound, to determine the specific binding interactions of the compounds to LPA_2,4–6_. The concentration-dependent change in RI (ΔRI) signal from the compounds to LPA_2,4–6_ or vector was fitted using the single site total vs nonspecific binding isotherm using GraphPad *Prism*. Specific *K*_D_ values were determined by fitting the total minus nonspecific signal to a single site binding isotherm.

The detailed free solution assay method was described previously.^25,26^ All the compounds were dissolved in 100% DMSO, aliquoted, and frozen at −80 °C for 1 week. The compound dilution series was freshly prepared in 0.5% DMSO/PBS (pH 7.4) to keep the maximum compound in solution. In the final assay, total protein concentration was maintained at 25 μg/mL with 0, 0.08, 0.4, 2, 10, 50, and 250 nM concentrations of the compound in a final buffer composition of 0.25% DMSO/PBS. Receptor-compound mixture was incubated at rt for about an hour on a shaker and then filled in a dropix sample well tray in the format of reference then sample and finally introduced to CIR using an automated Mitos Dropix (Dolomite Microfluidics, UK) sample introducer. The detailed description of the CIR was mentioned elsewhere.^31,32^ It is a benchtop RI reader that combined a compensated interferometer with a Mitos Dropix (an automated droplet generator) and a syringe pump. The compensated interferometer, which consisted of a diode laser, one or two mirrors, one glass capillary, and a CCD camera, measures the RI change from a solution undergoing conformational and/or hydration alteration compared to a reference (with no such binding events). ΔRI is measured by capturing the translational shifts in backscattered light interference fringes produced from the interaction between an expanded beam profile of the laser and a capillary filled with droplets of sample-reference solutions. The positional shift of the backscattered fringes, which is equivalent to molecular interaction, was quantified using fast Fourier transform of selected bright fringes captured in a CCD camera. The data acquisition and analysis were performed using a LabVIEW interface designed at the laboratory.

### In Silico Experiments

Docking calculations were performed using *Autodock4*(33) [using: ga_num_evals (depending on the number of rotatable bonds) = 6 310 000 (for compounds **3**, **13**, **53**, **54**, **55**) and 25 000 000 (for compound **38**), ga_run = 100 and all the other parameters set to their default values]. The LPA_2_ receptor model was generated using SwissModel^34^ and the crystal structure with PDB ID 4Z35(19) as a template. The generated model was prepared for docking using pdb 2pqr(35,36) with the propka^37,38^ protonation option at a pH of 7.4 and the peoepb force field.^39^ All the analyzed compounds were modeled using *RDKIT* (Open-source cheminformatics) and its protonation state adjusted at pH 7.4 by the ChemAxon cxcalc module (command line version of ChemAxon’s *Calculator Plugins*, v16.10.24.0, 2016). Binding mode pictures were created using *PyMOL* v2.5.0.

### Mutagenesis Experiments

Amino terminal hemagglutinin (HA)-tagged LPA_2_ point mutants K22A, R107A, Q108A, and K278A containing pcDNA3.1 plasmids were provided by GenScript. For expression of the different constructs, McA-RH7777 (CRL-1601, ATCC) cells were selected, as they have been previously used for point mutation experiments of LPA receptors.^40^ Cells were grown in Dulbecco’s modified Eagle’s medium supplemented with 10% (v/v) fetal bovine serum and 1% (v/v) penicillin-streptomycin and kept at 37 °C and 5% CO_2_. Cells were transiently transfected with the different plasmids using lipofectamine and following the manufacturer’s procedure. Successful transfection was confirmed by flow cytometry analysis. For these experiments, 1 × 10^5^ cells were resuspended in 50 μL of PBS with 2 mM EDTA and 0.5% BSA. Anti-HA antibody (Santa Cruz, sc-7392; 1 μg per 1 × 10^6^ cells) was added and cells were incubated for 30 min at rt with shaking. Cells were centrifuged, washed with buffer, and incubated with antimouse Alexa Fluor 488 (Invitrogen, 1:5000) for 30 min at rt with shaking and protected from light. Cells were centrifuged, washed with buffer, resuspended in 0.3 mL of buffer and analyzed by flow cytometry in a FACScalibur instrument (Becton Dickinson) at the UCM’s microscopy and flow cytometry unit. After confirming the transfection by flow cytometry, calcium mobilization experiments were carried out as previously described.

### Permeability and Microsomal Stability

These studies were carried out as previously described with minor modifications.^41,42^ The assessment of the membrane permeability of synthesized compounds and propranolol and metoprolol as reference compounds was performed in a commercially available 96-well Corning Gentest precoated PAMPA plate system (Cultek S.L.U., Spain). Prior to use, the precoated PAMPA plate system was warmed to rt for 30 min and 300 μL of 200 μM solution of tested compound in 2% DMSO in PBS (pH 7.4) were added into wells in the receiver (donor) plate. Then, 200 μL of PBS were added into wells in the filter (acceptor) plate. The filter plate was placed on the receiver plate by slowly lowering the precoated PAMPA plate until it sits on the receiver plate. The assembly was incubated at rt for 5 h, and then buffer samples were collected carefully from each plate. The final concentrations of compound in both donor and acceptor wells were analyzed by HPLC-MS and quantification was estimated by using the peak area integration normalized with an internal standard. Permeability value of the compounds was calculated using the following formula: *P* (cm/s) = {−ln[1 – *C*_A_(*t*)/*C*_eq_]}/[*A*(1/*V*_D_ + 1/*V*_A_)*t*], where *A* = filter area (0.3 cm^2^), *V*_D_ = donor well volume (0.3 mL), *V*_A_ = acceptor well volume (0.2 mL), *t* = incubation time (s), *C*_A_(*t*) = compound concentration (μM) in the acceptor well at time *t*, *C*_D_(*t*) = compound concentration (μM) in donor well at time *t*, and *C*_eq_ = [*C*_D_(*t*)*V*_D_ + *C*_A_(*t*)*V*_A_]/(*V*_D_ + *V*_A_). Assays were performed in duplicate, and the compound was tested in two different plates on different days.

For measuring the stability in mouse and human liver microsomes, compounds were incubated at 37 °C at a final concentration of 1 or 5 μM in PBS, respectively, together with a solution of nicotinamide adenine dinucleotide phosphate (NADPH) in PBS (final concentration of 2 mM) and a solution of MgCl_2_ in PBS (final concentration of 5 mM). Reactions were initiated by the addition of a suspension of mouse liver microsomes (MLMs) (male CD-1 mice pooled, Sigma-Aldrich) or human liver microsomes (HLMs) (male human pooled, Sigma-Aldrich), respectively, at a final protein concentration of 1 mg/mL. The solutions were vortexed and incubated at 37 °C. Aliquots of 100 μL were quenched at time zero and at seven points ranging to 2 h (MLM) or 4 h (HLM) by pouring into 100 μL of ice-cold acetonitrile. Quenched samples were centrifuged at 10 000*g* for 10 min, and the supernatants were filtered through a polytetrafluoroethylene (PTFE) membrane syringe filter (pore size of 0.2 μm, 13 mm in diameter, GE Healthcare Life Sciences). The relative disappearance of the compound under study over the course of the incubation was monitored by HPLC-MS using SIM mode. Concentrations were quantified by measuring the area under the peak ([M + H]^+^) normalized with an internal standard and converted to the percentage of compound remaining, using the time zero peak area value as 100%. The natural logarithm of the remaining percentage versus time data for each compound was fit to a linear regression, and the slope was used to calculate the degradation half-life (*t*_1/2_).

### Determination of the In Vivo Levels of Compound

Compound **54** was administered intraperitoneally (25 mg/kg) in adult female 12–16 weeks old C57Bl/6J mice. At 1, 2, and 4 h after drug administration (*n* = 3 for each time and sample), mice were sacrificed and their brains, spinal cords, and blood were obtained. The brain and spinal cord were immediately frozen and kept at −80 °C until analysis. Blood was allowed to clot at rt for 30 min and centrifuged at 4 °C for 10 min at 16 000*g*. Serum was transferred to a clean polypropylene tube and stored at −80 °C until analysis. For analysis, a volume of cold acetonitrile was added to the serum. The sample was incubated in an ice bath for 10 min and centrifuged at 4 °C for 10 min at 16 000*g*. The resulting organic layer was filtered through a PTFE filter (0.2 μm, 13 mm diameter, Fisher Scientific) and 20 μL of the sample analyzed by LC-MS/MS at the UCM’s Mass Spectrometry CAI. Separation was performed using a Phenomenex Gemini 5 μm C18 110A 150 × 2 mm column (run time 8 min; flow 0.5 mL/min; gradient: 0.5 min 10% Phase B, 2 min 60% Phase B, 4.5–6 min 100% Phase B, 7–8 min 10% Phase B; Phase A: water with formic acid 0.1%; Phase B: acetonitrile). The entire LC eluent was directly introduced to an electrospray ionization (ESI) source operating in the positive ion mode for LC MS/MS analysis on a Shimadzu LCMS8030 triple quadrupole mass spectrometer coupled to UHPLC with an oven temperature of 31.5 °C. The mass spectrometer ion optics were set in the multiple reaction monitoring mode and the transition selected for quantification was 432.90 > 215.10 (CE: −30 V).

### Spinal Cord Injury In Vivo Model

All Surgical Procedures Were Approved by the Universitat Autònoma De Barcelona Animal Care Committee (CEEAH 4273) and followed the guidelines of the European Commission on Animal Care (EU Directive 2010/63/EU). Adult female C57Bl/6J mice (10–12 weeks old) and LPA_2_ null mice were anesthetized by intramuscular injection with a mixture of ketamine and xylazine (90:10 mg/kg). A laminectomy was performed at the 11th thoracic vertebrae and the exposed spinal cord was contused using the Infinite Horizon Impactor device (Precision Scientific Instrumentation) using a force of 60 kdynes. Only mice showing a spinal cord tissue displacement ranging between 450 and 550 μm were selected. One hour after injury, compound **54** was injected intraperitoneally (25 mg/kg) which was then repeated daily for 10 consecutive days.

## Supporting Information Available

Representative agonist curves for compounds **1**, **13**, and **54** (Figure S1); dose–activity curves for compounds **13**, **44**, and **53**–**55** (Figure S2); representative plots of FSA-CIR signal vs ligand concentration for the determination of affinity constants of compound **54** (Figure S3); numbered structures used in the structural characterization by NMR of all final compounds (Figure S4); HPLC gradients employed in HPLC-MS analysis of all tested compounds (Table S1); detailed synthetic procedures and characterization data of all intermediates; NMR spectra and HPLC trace analysis of final compounds (PDF)
PDB files of the structure of the LPA_2_ receptor and select compounds from this study (ZIP)
Molecular formula strings (CSV)

## Dedication

This paper is dedicated to Prof. Joan Bosch on the occasion of his 75th birthday.

## Abbreviations Used

BMS: Basso Mouse Scale
CNS: central nervous system
*E*_max_: maximal receptor activation
FSA-CIR: free solution assay-compensated interferometric reader
GPCR: G protein-coupled receptor
HLM: human liver microsome
IC_50_: concentration of compound that inhibit the 50% of the activation induced by LPA
i.p.: intraperitoneally
LPA: lysophosphatidic acid
MLM: mouse liver microsome
MW: microwave
N.E.: no effect
P: permeability
PK: pharmacokinetic
SCI: spinal cord injury
s.e.m.: standard error of the mean

## References

[ref1] Ahuja C. S.; Wilson J. R.; Nori S.; Kotter M. R. N.; Druschel C.; Curt A.; Fehlings M. G. Traumatic spinal cord injury. Nat. Rev. Dis. Primers 2017, 3, 17018 10.1038/nrdp.2017.18. 28447605

[ref2] Hutson T. H.; Di Giovanni S. The translational landscape in spinal cord injury: focus on neuroplasticity and regeneration. Nat. Rev. Neurol. 2019, 15, 732–745. 10.1038/s41582-019-0280-3. 31728042

[ref3] Fehlings M. G.; Chen Y.; Aarabi B.; Ahmad F.; Anderson K. D.; Dumont T.; Fourney D. R.; Harrop J. S.; Kim K. D.; Kwon B. K.; Lingam H. K.; Rizzo M.; Shih L. C.; Tsai E. C.; Vaccaro A.; McKerracher L. A randomized controlled trial of local delivery of a Rho inhibitor (VX-210) in patients with acute traumatic cervical spinal cord injury. J. Neurotrauma 2021, 38, 2065–2072. 10.1089/neu.2020.7096. 33559524 PMC8309435

[ref4] Liu Z.; Yang Y.; He L.; Pang M.; Luo C.; Liu B.; Rong L. High-dose methylprednisolone for acute traumatic spinal cord injury: A meta-analysis. Neurology 2019, 93, e841–e850. 10.1212/WNL.0000000000007998. 31358617

[ref5] López-Vales R.; David S. Bioactive lipids in inflammation after central nervous system injury. Adv. Exp. Med. Biol. 2019, 1127, 181–194. 10.1007/978-3-030-11488-6_12. 31140179

[ref6] David S.; López-Vales R. Bioactive lipid mediators in the initiation and resolution of inflammation after spinal cord injury. Neuroscience 2021, 466, 273–297. 10.1016/j.neuroscience.2021.04.026. 33951502

[ref7] Yung Y. C.; Stoddard N. C.; Mirendil H.; Chun J. Lysophosphatidic acid signaling in the nervous system. Neuron 2015, 85, 669–682. 10.1016/j.neuron.2015.01.009. 25695267 PMC4400838

[ref8] Santos-Nogueira E.; López-Serrano C.; Hernández J.; Lago N.; Astudillo A. M.; Balsinde J.; Estivill-Torrús G.; de Fonseca F. R.; Chun J.; López-Vales R. Activation of lysophosphatidic acid receptor type 1 contributes to pathophysiology of spinal cord injury. J. Neurosci. 2015, 35, 10224–10235. 10.1523/JNEUROSCI.4703-14.2015. 26180199 PMC4502263

[ref9] Choi J. W.; Herr D. R.; Noguchi K.; Yung Y. C.; Lee C. W.; Mutoh T.; Lin M. E.; Teo S. T.; Park K. E.; Mosley A. N.; Chun J. LPA receptors: subtypes and biological actions. Annu. Rev. Pharmacol. Toxicol. 2010, 50, 157–186. 10.1146/annurev.pharmtox.010909.105753. 20055701

[ref10] Kihara Y.; Maceyka M.; Spiegel S.; Chun J. Lysophospholipid receptor nomenclature review: IUPHAR Review 8. Br. J. Pharmacol. 2014, 171, 3575–3594. 10.1111/bph.12678. 24602016 PMC4128058

[ref11] González-Gil I.; Zian D.; Vázquez-Villa H.; Ortega-Gutiérrez S.; López-Rodríguez M. L. The status of the lysophosphatidic acid receptor type 1 (LPA1R). MedChemComm. 2015, 6, 13–26. 10.1039/C4MD00333K.

[ref12] Suardíaz M.; Galan-Arriero I.; Avila-Martin G.; Estivill-Torrús G.; de Fonseca F. R.; Chun J.; Gómez-Soriano J.; Bravo-Esteban E.; Taylor J. Spinal cord compression injury in lysophosphatidic acid 1 receptor-null mice promotes maladaptive pronociceptive descending control. Eur. J. Pain 2016, 20, 176–185. 10.1002/ejp.695. 25820316

[ref13] López-Serrano C.; Santos-Nogueira E.; Francos-Quijorna I.; Coll-Miró M.; Chun J.; López-Vales R. Lysophosphatidic acid receptor type 2 activation contributes to secondary damage after spinal cord injury in mice. Brain Behav. Immun. 2019, 76, 258–267. 10.1016/j.bbi.2018.12.007. 30550929 PMC6348147

[ref14] Beck H. P.; Kohn T.; Rubenstein S.; Hedberg C.; Schwandner R.; Hasslinger K.; Dai K.; Li C.; Liang L.; Wesche H.; Frank B.; An S.; Wickramasinghe D.; Jaen J.; Medina J.; Hungate R.; Shen W. Discovery of potent LPA2 (EDG4) antagonists as potential anticancer agents. Bioorg. Med. Chem. Lett. 2008, 18, 1037–1041. 10.1016/j.bmcl.2007.12.024. 18178086

[ref15] Fells J. I.; Tsukahara R.; Fujiwara Y.; Liu J.; Perygin D. H.; Osborne D. A.; Tigyi G.; Parrill A. L. Identification of non-lipid LPA3 antagonists by virtual screening. Bioorg. Med. Chem. 2008, 16, 6207–6217. 10.1016/j.bmc.2008.04.035. 18467108 PMC2483252

[ref16] Ohta H.; Sato K.; Murata N.; Damirin A.; Malchinkhuu E.; Kon J.; Kimura T.; Tobo M.; Yamazaki Y.; Watanabe T.; Yagi M.; Sato M.; Suzuki R.; Murooka H.; Sakai T.; Nishitoba T.; Im D. S.; Nochi H.; Tamoto K.; Tomura H.; Okajima F. Ki16425, a subtype-selective antagonist for EDG-family lysophosphatidic acid receptors. Mol. Pharmacol. 2003, 64, 994–1005. 10.1124/mol.64.4.994. 14500756

[ref17] Ma L.; Matsumoto M.; Xie W.; Inoue M.; Ueda H. Evidence for lysophosphatidic acid 1 receptor signaling in the early phase of neuropathic pain mechanisms in experiments using Ki-16425, a lysophosphatidic acid 1 receptor antagonist. J. Neurochem. 2009, 109, 603–610. 10.1111/j.1471-4159.2009.05987.x. 19222705

[ref18] González-Gil I.; Zian D.; Vázquez-Villa H.; Hernández-Torres G.; Martínez R. F.; Khiar-Fernández N.; Rivera R.; Kihara Y.; Devesa I.; Mathivanan S.; Del Valle C. R.; Zambrana-Infantes E.; Puigdomenech M.; Cincilla G.; Sanchez-Martinez M.; Rodríguez de Fonseca F.; Ferrer-Montiel A. V.; Chun J.; López-Vales R.; López-Rodríguez M. L.; Ortega-Gutiérrez S. A novel agonist of the type 1 lysophosphatidic acid receptor (LPA(1)), UCM-05194, shows efficacy in neuropathic pain amelioration. J. Med. Chem. 2020, 63, 2372–2390. 10.1021/acs.jmedchem.9b01287. 31790581 PMC7344333

[ref19] Chrencik J. E.; Roth C. B.; Terakado M.; Kurata H.; Omi R.; Kihara Y.; Warshaviak D.; Nakade S.; Asmar-Rovira G.; Mileni M.; Mizuno H.; Griffith M. T.; Rodgers C.; Han G. W.; Velasquez J.; Chun J.; Stevens R. C.; Hanson M. A. Crystal structure of antagonist bound human lysophosphatidic acid receptor 1. Cell 2015, 161, 1633–1643. 10.1016/j.cell.2015.06.002. 26091040 PMC4476059

[ref20] Meanwell N. A. Fluorine and fluorinated motifs in the design and application of bioisosteres for drug design. J. Med. Chem. 2018, 61, 5822–5880. 10.1021/acs.jmedchem.7b01788. 29400967

[ref21] Shinada N. K.; de Brevern A. G.; Schmidtke P. Halogens in protein-ligand binding mechanism: A structural perspective. J. Med. Chem. 2019, 62, 9341–9356. 10.1021/acs.jmedchem.8b01453. 31117513

[ref22] Lassalas P.; Gay B.; Lasfargeas C.; James M. J.; Tran V.; Vijayendran K. G.; Brunden K. R.; Kozlowski M. C.; Thomas C. J.; Smith A. B. 3rd; Huryn D. M.; Ballatore C. Structure property relationships of carboxylic acid isosteres. J. Med. Chem. 2016, 59, 3183–3203. 10.1021/acs.jmedchem.5b01963. 26967507 PMC4833640

[ref23] Baksh M. M.; Kussrow A. K.; Mileni M.; Finn M. G.; Bornhop D. J. Label-free quantification of membrane-ligand interactions using backscattering interferometry. Nat. Biotechnol. 2011, 29, 357–360. 10.1038/nbt.1790. 21399645 PMC3246389

[ref24] Bornhop D. J.; Latham J. C.; Kussrow A.; Markov D. A.; Jones R. D.; Sørensen H. S. Free-solution, label-free molecular interactions studied by back-scattering interferometry. Science 2007, 317, 1732–1736. 10.1126/science.1146559. 17885132

[ref25] Mizuno H.; Kihara Y.; Kussrow A.; Chen A.; Ray M.; Rivera R.; Bornhop D. J.; Chun J. Lysophospholipid G protein-coupled receptor binding parameters as determined by backscattering interferometry. J. Lipid Res. 2019, 60, 212–217. 10.1194/jlr.D089938. 30463988 PMC6314248

[ref26] Ray M.; Nagai K.; Kihara Y.; Kussrow A.; Kammer M. N.; Frantz A.; Bornhop D. J.; Chun J. Unlabeled lysophosphatidic acid receptor binding in free solution as determined by a compensated interferometric reader. J. Lipid Res. 2020, 61, 1244–1251. 10.1194/jlr.D120000880. 32513900 PMC7397748

[ref27] Whetstone W. D.; Hsu J. Y.; Eisenberg M.; Werb Z.; Noble-Haeusslein L. J. Blood-spinal cord barrier after spinal cord injury: relation to revascularization and wound healing. J. Neurosci. Res. 2003, 74, 227–239. 10.1002/jnr.10759. 14515352 PMC2837839

[ref28] Basso D. M.; Fisher L. C.; Anderson A. J.; Jakeman L. B.; McTigue D. M.; Popovich P. G. Basso Mouse Scale for locomotion detects differences in recovery after spinal cord injury in five common mouse strains. J. Neurotrauma 2006, 23, 635–659. 10.1089/neu.2006.23.635. 16689667

[ref29] Hopper D. W.; Ragan S. P.; Hooks S. B.; Lynch K. R.; Macdonald T. L. Structure-activity relationships of lysophosphatidic acid: conformationally restricted backbone mimetics. J. Med. Chem. 1999, 42, 963–970. 10.1021/jm970809v. 10090779

[ref30] Bornhop D. J.; Kammer M. N.; Kussrow A.; Flowers R. A. 2nd; Meiler J. Origin and prediction of free-solution interaction studies performed label-free. Proc. Natl. Acad. Sci. U.S.A. 2016, 113, E1595–E1604. 10.1073/pnas.1515706113. 26960999 PMC4812771

[ref31] Kammer M. N.; Kussrow A. K.; Bornhop D. J. Longitudinal pixel averaging for improved compensation in backscattering interferometry. Opt. Lett. 2018, 43, 482–485. 10.1364/OL.43.000482. 29400820

[ref32] Kammer M. N.; Kussrow A. K.; Olmsted I. R.; Bornhop D. J. A highly compensated interferometer for biochemical analysis. ACS Sens. 2018, 3, 1546–1552. 10.1021/acssensors.8b00361. 29984991

[ref33] Morris G. M.; Huey R.; Lindstrom W.; Sanner M. F.; Belew R. K.; Goodsell D. S.; Olson A. J. AutoDock4 and AutoDockTools4: Automated docking with selective receptor flexibility. J. Comput. Chem. 2009, 30, 2785–2791. 10.1002/jcc.21256. 19399780 PMC2760638

[ref34] Biasini M.; Bienert S.; Waterhouse A.; Arnold K.; Studer G.; Schmidt T.; Kiefer F.; Cassarino T. G.; Bertoni M.; Bordoli L.; Schwede T. SWISS-MODEL: modelling protein tertiary and quaternary structure using evolutionary information. Nucleic Acids Res. 2014, 42, W252–W258. 10.1093/nar/gku340. 24782522 PMC4086089

[ref35] Dolinsky T. J.; Czodrowski P.; Li H.; Nielsen J. E.; Jensen J. H.; Klebe G.; Baker N. A. PDB2PQR: expanding and upgrading automated preparation of biomolecular structures for molecular simulations. Nucleic Acids Res. 2007, 35, W522–525. 10.1093/nar/gkm276. 17488841 PMC1933214

[ref36] Dolinsky T. J.; Nielsen J. E.; McCammon J. A.; Baker N. A. PDB2PQR: an automated pipeline for the setup of Poisson-Boltzmann electrostatics calculations. Nucleic Acids Res. 2004, 32, W665–667. 10.1093/nar/gkh381. 15215472 PMC441519

[ref37] Li H.; Robertson A. D.; Jensen J. H. Very fast empirical prediction and rationalization of protein pKa values. Proteins 2005, 61, 704–721. 10.1002/prot.20660. 16231289

[ref38] Søndergaard C. R.; Olsson M. H.; Rostkowski M.; Jensen J. H. Improved treatment of ligands and coupling effects in empirical calculation and rationalization of pKa values. J. Chem. Theory Comput. 2011, 7, 2284–2295. 10.1021/ct200133y. 26606496

[ref39] Czodrowski P.; Dramburg I.; Sotriffer C. A.; Klebe G. Development, validation, and application of adapted PEOE charges to estimate pKa values of functional groups in protein-ligand complexes. Proteins 2006, 65, 424–437. 10.1002/prot.21110. 16927370

[ref40] Valentine W. J.; Fells J. I.; Perygin D. H.; Mujahid S.; Yokoyama K.; Fujiwara Y.; Tsukahara R.; Van Brocklyn J. R.; Parrill A. L.; Tigyi G. Subtype-specific residues involved in ligand activation of the endothelial differentiation gene family lysophosphatidic acid receptors. J. Biol. Chem. 2008, 283, 12175–12187. 10.1074/jbc.M708847200. 18316373 PMC3774115

[ref41] Marín-Ramos N. I.; Balabasquer M.; Ortega-Nogales F. J.; Torrecillas I. R.; Gil-Ordóñez A.; Marcos-Ramiro B.; Aguilar-Garrido P.; Cushman I.; Romero A.; Medrano F. J.; Gajate C.; Mollinedo F.; Philips M. R.; Campillo M.; Gallardo M.; Martín-Fontecha M.; López-Rodríguez M. L.; Ortega-Gutiérrez S. A potent isoprenylcysteine carboxylmethyltransferase (ICMT) inhibitor improves survival in Ras-driven acute myeloid leukemia. J. Med. Chem. 2019, 62, 6035–6046. 10.1021/acs.jmedchem.9b00145. 31181882

[ref42] Marcos-Ramiro B.; Gil-Ordóñez A.; Marín-Ramos N. I.; Ortega-Nogales F. J.; Balabasquer M.; Gonzalo P.; Khiar-Fernández N.; Rolas L.; Barkaway A.; Nourshargh S.; Andrés V.; Martín-Fontecha M.; López-Rodríguez M. L.; Ortega-Gutiérrez S. Isoprenylcysteine carboxylmethyltransferase-based therapy for Hutchinson-Gilford progeria syndrome. ACS Cent. Sci. 2021, 7, 1300–1310. 10.1021/acscentsci.0c01698. 34471675 PMC8393201

