# Supplementary material for: Novel Antagonist of the Type 2 Lysophosphatidic Acid Receptor (LPA2), UCM-14216, Ameliorates Spinal Cord Injury in Mice
Source: J Med Chem. 2022 Aug 10;65(16):10956–74. doi: 10.1021/acs.jmedchem.2c00046 (PMC9421655; doi:10.1021/acs.jmedchem.2c00046)

Supporting Information

A novel antagonist of the type 2 lysophosphatidic acid receptor (LPA_2), UCM-14216, ameliorates spinal cord injury in mice

Nora Khiar-Fernández,[1,‡] Debora Zian,[1,‡] Henar Vázquez-Villa,[1] R. Fernando Martínez,[1] Andrea Escobar-Peña,[1] Román Foronda-Sainz,[1] Manisha Ray,[2] Maria Puigdomenech-Poch,[3] Giovanni Cincilla,[4] Melchor Sánchez-Martínez,[4,5] Yasuyuki Kihara,[2] Jerold Chun,[2] Rubèn López-Vales,[3] María L. López-Rodríguez[1,*] and Silvia Ortega-Gutiérrez[1,*]

[1]Departamento de Química Orgánica I, Facultad de Ciencias Químicas, Universidad Complutense de Madrid, E-28040 Madrid, Spain
[2]Translational Neuroscience Initiative, Sanford Burnham Prebys Medical Discovery Institute, 10901 North Torrey Pines Road, La Jolla, CA 92037, USA
[3]Departament de Biologia Cel·lular, Fisiologia i Immunologia, Institut de Neurociències, Centro de Investigación Biomédica en Red sobre Enfermedades Neurodegenerativas (CIBERNED), Universitat Autònoma de Barcelona, E-08193 Bellaterra, Barcelona, Spain
[4]Molomics, Barcelona Science Park, Baldiri i Reixac 4-8, E-08028 Barcelona, Spain
[5]Burua Scientific, E-08810 Sant Pere de Ribes, Spain

Correspondence: siortega@ucm.es, mluzlr@ucm.es

Table of Contents

1. Supporting Figures **S2**

2. Chemistry **S7**

2.1. HPLC gradients employed in HPLC-MS analysis of all tested compounds S7

2.2. Numbered chemical structures for NMR assignation of final compounds S8

2.3. Synthesis and characterization data of intermediate compounds S9

3. NMR spectra and HPLC trace analysis of final compounds **S37**

1. Supporting Figures

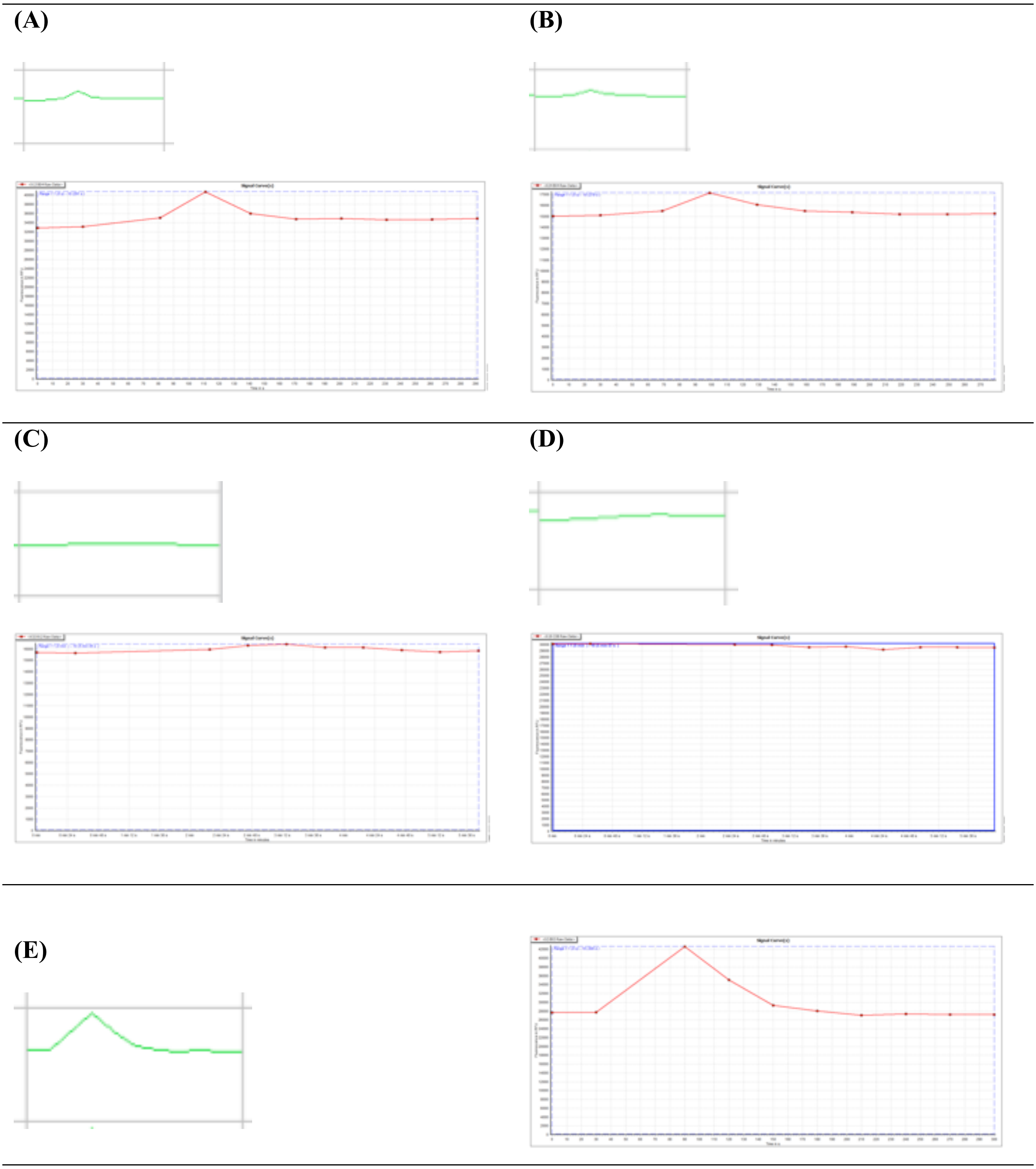

Figure S1. Representative snapshots showing the lack of significant agonist activity of selected compounds. (A) **1**; (B) **13**; (C) **54**; and (D) Ki16425. (E) Graphs show the agonist activity induced by 10 µM of LPA (18:1, 1-oleoyl-*sn*-glycerol-3-phosphate) for the sake of comparison. B103 cells stably expressing LPA_2 receptor were plated on collagen-coated black-wall clear-bottom 96-well plates (Corning) at a density of 50000 cells/well and cultured overnight. The culture medium was then replaced with Fluo-4 NW dye loading solution containing 2.5 µM of probenecid and incubated

for 30 minutes at 37ºC followed by an additional 30 minutes at rt. Fluorescence changes were registered in a FluoStar Optima instrument (BMG Labtech) at 525 nm using an excitation wavelength of 494 nm. Each well was monitored for 240 s. 20 μL of the test compound from a 6x stock solution in assay buffer were added after 120 s of starting the measurement. Ca^{2+} transient increase was quantified by calculating the difference between maximum and baseline values for each well. As positive controls, 10 μM LPA and 10 μM ionomycin were included in every experiment. Graphs shown are representative of two to three independent experiments.

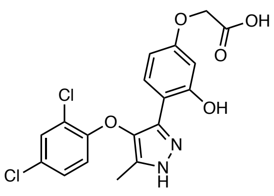

13

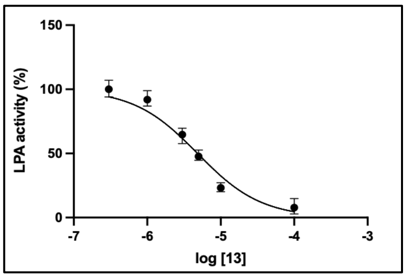

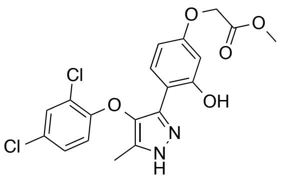

44

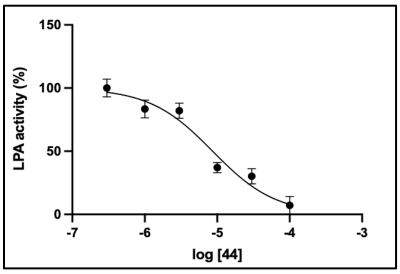

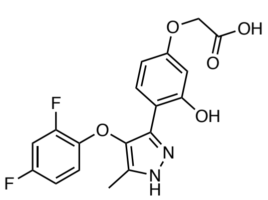

53

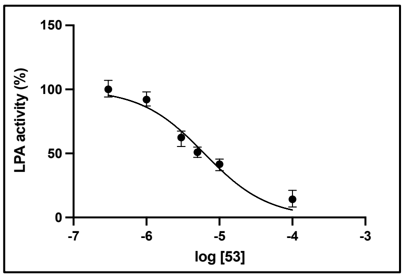

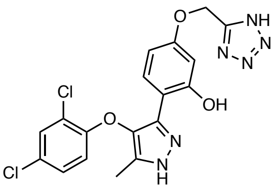

54

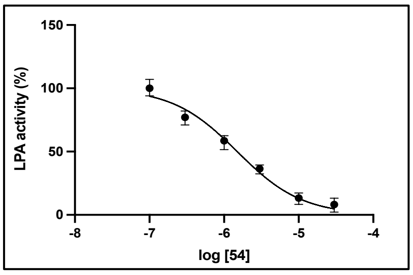

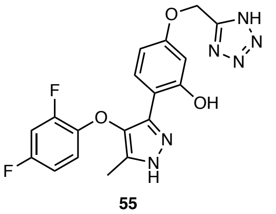

55

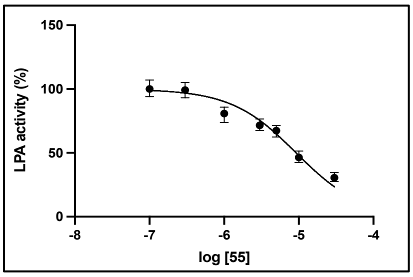

Figure S2. Dose response curves for compounds **13**, **44**, **53**, **54**, and **55**. B103 cells stably expressing LPA_2 receptor were incubated with different concentrations of the compound under study and subsequently stimulated with LPA (10 μM). 100% LPA activity represents the maximal Ca^{2+}

increase induced by 10 μM LPA in the absence of compound. Curves were generated with PRISM software version 5 (GraphPad Software Inc, San Diego, CA, USA) using nonlinear regression analysis. Each data point corresponds to the mean±SEM of two to four independent experiments carried out in triplicate or quadruplicate.

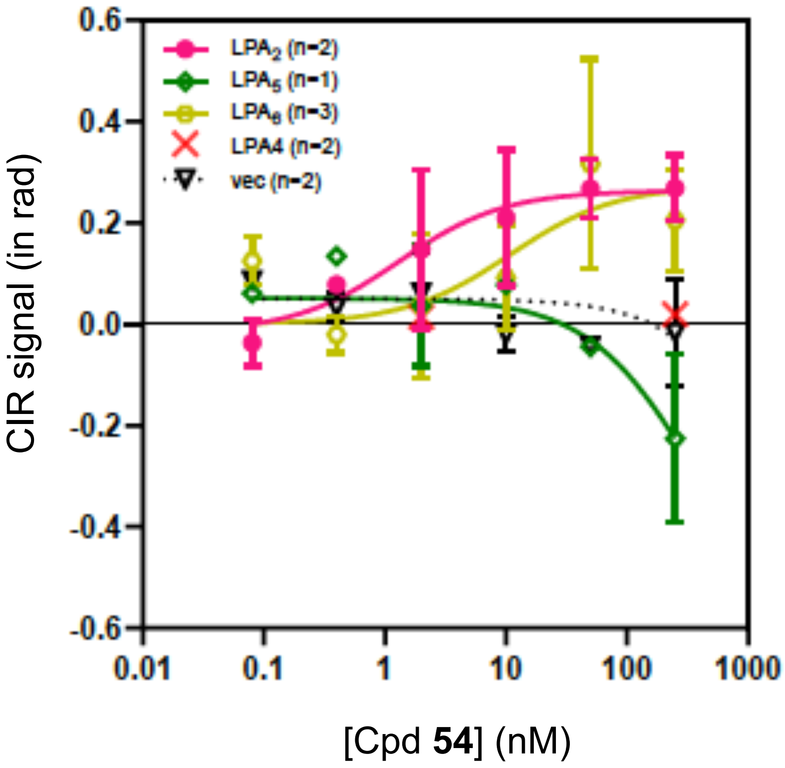

Figure S3. Specific binding signals for compound **54** to $LPA_{2,4\text{-}6}$ receptors. Representative plots of CIR signal versus ligand concentration for the determination of affinity constant. Each data point represents the average of at least four independent measurements. Membranes from cells transfected with the empty plasmid -without any receptor (VEC)- were used as negative control.

2. Chemistry

2.1. HPLC gradients employed in HPLC-MS analysis of all tested compounds

Table S1: HPLC gradients.

**Method A**		**Method B**		**Method C**	
Column SB-C3 Water/acetonitrile		Column XDB-C18 Water/acetonitrile		Column XDB-C18 Water/acetonitrile	
**t (min)**	**% B**	**t (min)**	**% B**	**t (min)**	**% B**
0	0	0	0	0	0
2	0	2	0	0.5	0
8	80	8	50	2	60
20	100	20	100	4.5	100
25	100	25	100	6	100
30	0	30	0	7	0

2.2 Numbered chemical structures for NMR assignation of final compounds

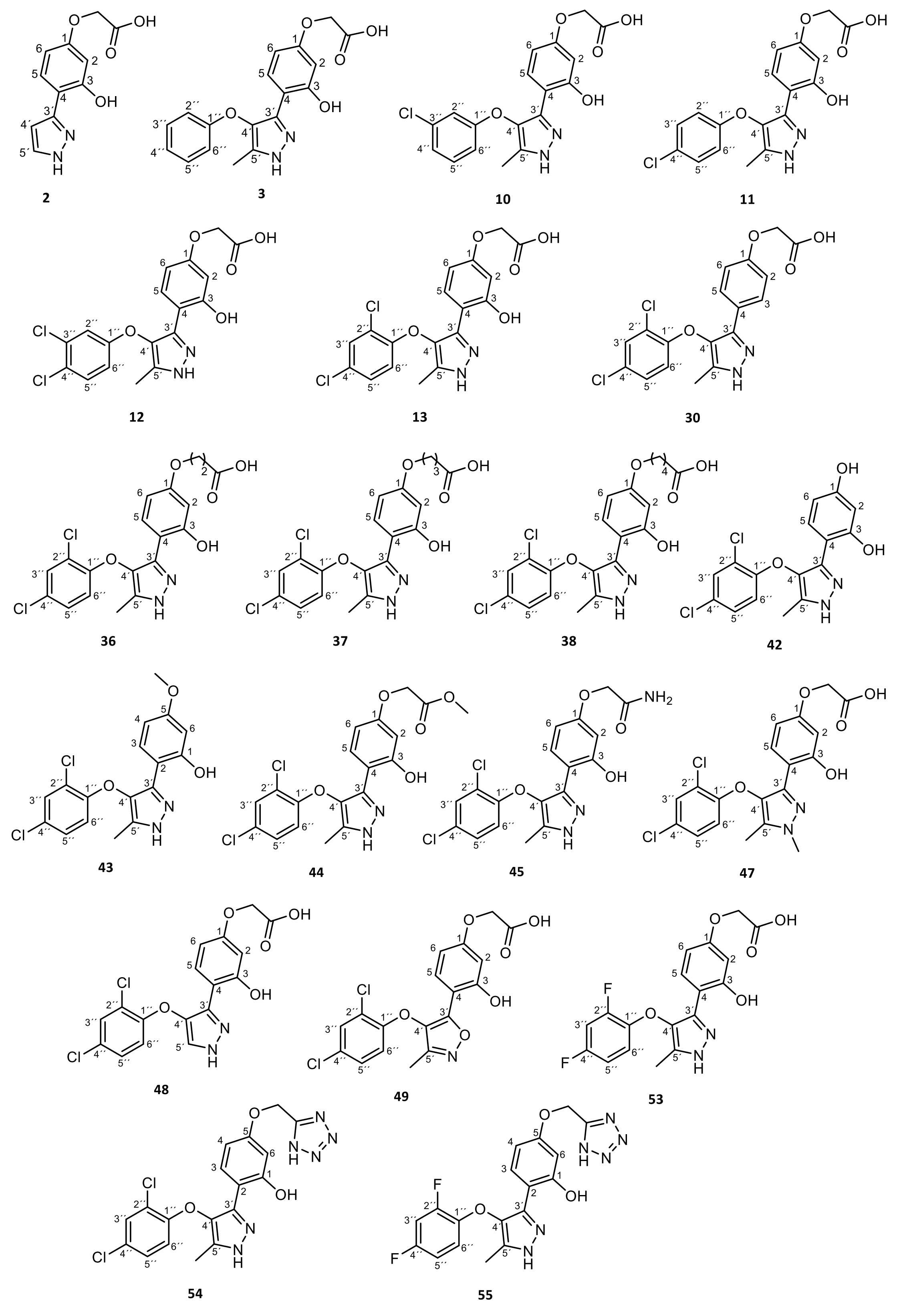

Figure S4: Structures of final compounds **2, 3, 10-13, 30, 36-38, 42-45, 47-49, 53-55.**

2.3. Synthesis and characterization data of intermediate compounds

2.3.1. Synthesis of intermediates 4-9 (Scheme 1)

7-Hydroxy-4*H*-chromen-4-one (4). A solution of 1-(2,4-dihydroxyphenyl)ethanone (500 mg, 3.29 mmol, 1 equiv) in triethyl orthoformate (2.9 mL, 17.4 mmol, 5.3 equiv) was treated with 70% perchloric acid (0.3 mL, 3.83 mmol, 1.2 equiv). The resulting warm and thick dark solution was stirred until it cooled to rt (about 1 h). Anhydrous diethyl ether (12.5 mL) was added to precipitate out the brown-colored intermediate of the oxonium perchlorate salt. The salt was taken in water and hydrolyzed by heating to reflux for 5 min. The reaction mixture was then allowed to cool at rt and was stirred for 12 h. A dark brown solid precipitated out, which was filtered, dried under vacuum, and purified by flash chromatography (hexane/ethyl acetate, 1:1 to ethyl acetate) to yield pure hydroxychromone **4** in 47% yield.

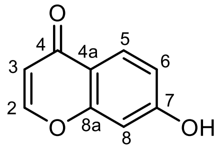

4

R_f: 0.56 (hexane/ethyl acetate, 2:8). IR (ATR): ν 3526 (OH); 1630 (C=O). ^{1}H-NMR (acetone-d_6, 300 MHz): δ 7.27 (d, J = 5.6, 1H, H_3); 7.34 (d, J = 2.2, 1H, H_8); 7.40 (dd, J = 9.0, 2.3, 1H, H_6); 8.33 (d, J = 9.0, 1H, H_5); 8.98 (d, J = 5.6, 1H, H_2). ^{13}C-NMR (acetone-d_6, 75 MHz): δ 103.6 (C_8); 108.5 (C_3); 120.2 (C_6); 120.3 (C_{4a}); 128.3 (C_5); 154.5 (C_2); 161.6 (C_{8a}); 164.4 (C_7); 167.7 (C_4). MS (ESI, *m/z*): 161.1 [M-H]$^-$. HPLC (method B, t_R, min): 11.51.

Methyl [(4-oxo-4*H*-chromen-7-yl)oxy]acetate (5). Following the general procedure 4, compound **5** was obtained from hydroxychromone **4** (147 mg, 0.91 mmol) and methyl 2-bromoacetate (0.35 mL, 3.19 mmol, 3.5 equiv) in 24% yield. Chromatography: hexane to hexane/ethyl acetate, 7:3.

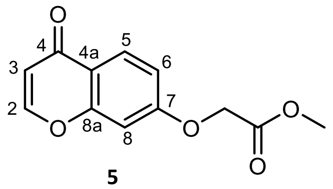

5

<u>mp</u>: 146-148 ºC. <u>R_f</u>: 0.21 (hexane/ethyl acetate, 7:3). <u>IR (ATR)</u>: ν 1756 (C=O); 1648 (C=O); 1214 (C-O-C). <u>^{1}H-NMR ($CDCl_3$, 300 MHz)</u>: δ 3.83 (s, 3H, CH_3); 4.73 (s, 2H, CH_2); 6.28 (d, *J* = 6.0, 1H, H_3); 6.82 (d, *J* = 2.4, 1H, H_8); 7.00 (dd, *J* = 8.9, 2.4, 1H, H_6); 7.77 (d, *J* = 6.0, 1H, H_2); 8.13 (d, *J* = 8.9, 1H, H_5). <u>^{13}C-NMR ($CDCl_3$, 75 MHz)</u>: δ 52.7 (CH_3); 65.4 (CH_2); 101.7 (C_8); 113.2 (C_3); 114.5 (C_6); 119.7 (C_{4a}); 127.8 (C_5); 155.1 (C_2); 158.1 (C_{8a}); 162.1 (C_7); 168.4 (C=O); 177.0 (C_4). <u>MS (ESI, *m/z*)</u>: 235.1 $[M+H]^+$. <u>HPLC (method B, t_R, min)</u>: 12.69.

1-(2,4-Dihydroxyphenyl)-2-phenoxyethanone (6). Following the general procedure 1, arylethanone **6** was obtained from phenoxyacetic acid (500 mg, 3.29 mmol) in 19% yield. Chromatography: hexane/DCM, 3:7 to DCM.

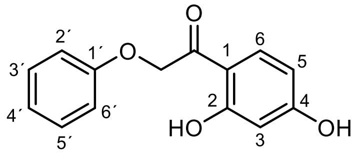

6

<u>mp</u>: 191-193 ºC. <u>R_f</u>: 0.44 (hexane/ethyl acetate, 7:3). <u>IR (ATR)</u>: ν 3316 (OH); 1628 (C=O); 1231 (C-O-C). <u>^{1}H-NMR (methanol-d_4, 300 MHz)</u>: δ 5.31 (s, 2H, CH_2); 6.31 (d, *J* = 2.2, 1H, H_3); 6.41 (dd, *J* = 8.8, 2.2, 1H, H_5); 6.93-7.00 (m, 3H, $3H_{Ar}$); 7.25-7.30 (m, 2H, $2H_{Ar}$); 7.80 (d, *J* = 8.9, 1H, H_6). <u>^{13}C-NMR (methanol-d_4, 75 MHz)</u>: δ 70.7 (CH_2); 103.8 (C_3); 109.6 (C_5); 112.4 (C_1); 115.8 ($2CH_{Ar}$); 122.4 (CH_{Ar}); 130.5 ($2CH_{Ar}$); 132.9 (C_6); 159.6 ($C_{1'}$); 166.3, 166.9 (C_2, C_4); 199.3 (C=O). <u>MS (ESI, *m/z*)</u>: 243.1 $[M-H]^-$. <u>HPLC (method B, t_R, min)</u>: 23.38.

2-Methyl-4-oxo-3-phenoxy-4*H*-chromen-7-yl acetate (7). Following the general procedure 2, chromone **7** was obtained from arylethanone **6** (35 mg, 0.14 mmol) in 99% yield.

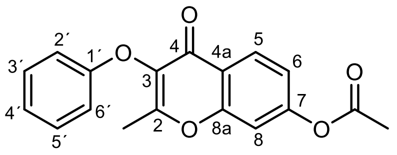

7

R_f: 0.58 (hexane/ethyl acetate, 7:3). IR (ATR): ν 1768, 1618 (C=O); 1195 (C-O-C). ^{1}H-NMR (methanol-d_4, 700 MHz): δ 2.35 (s, 3H, $COCH_3$); 2.44 (s, 3H, C_2CH_3); 6.94-6.96 (m, 2H, $H_{2'}$, $H_{6'}$); 7.02 (t, J = 7.4, 0.9, 1H, $H_{4'}$); 7.26-7.30 (m, 3H, H_6, $H_{3'}$, $H_{5'}$); 7.49 (d, J = 2.1, 1H, H_8); 8.16 (d, J = 8.7, 1H, H_5). ^{13}C-NMR (methanol-d_4, 175 MHz): δ 15.8 ($C_2\underline{C}H_3$); 20.9 ($CO\underline{C}H_3$); 112.4 (C_8); 115.8 ($C_{2'}$, $C_{6'}$); 121.1 (C_6); 122.8 (C_{4a}); 123.5 ($C_{4'}$); 127.8 (C_5); 130.7 ($C_{3'}$, $C_{5'}$); 137.6 (C_3); 156.6 (C_7); 157.6 (C_{8a}); 158.6 ($C_{1'}$); 163.9 (C_2); 170.1 (C=O); 174.2 (C_4). MS (ESI, *m/z*): 311.1 $[M+H]^+$. HPLC (method B, t_R, min): 25.30.

7-Hydroxy-2-methyl-3-phenoxy-4*H*-chromen-4-one (8). Following the general procedure 3, hydroxychromone **8** was obtained from acetoxychromone **7** (46 mg, 0.15 mmol) in 99% yield.

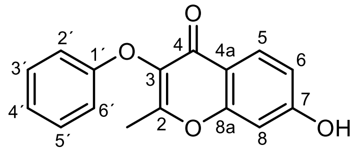

8

mp: 250-252 ºC. R_f: 0.29 (hexane/ethyl acetate, 7:3). IR (ATR): ν 3229 (OH); 1594 (C=O); 1249 (C-O-C). ^{1}H-NMR (methanol-d_4, 500 MHz): δ 2.39 (s, 3H, CH_3); 6.89-6.95 (m, 4H, H_6, H_8, $H_{2'}$, $H_{6'}$); 7.01 (t, J = 7.4, 1H, $H_{4'}$); 7.26-7.30 (m, 2H, $H_{3'}$, $H_{5'}$); 7.97 (d, J = 8.8, 1H, H_5). ^{13}CNMR (methanol-d_4, 125 MHz): δ 15.7 (CH_3); 103.3 (C_8); 115.8 ($C_{2'}$, $C_{6'}$); 116.4 (C_6); 117.8 (C_{4a}); 123.3 ($C_{4'}$); 128.1 (C_5); 130.7 ($C_{3'}$, $C_{5'}$); 137.0 (C_3); 158.7 (C_{8a}); 159.1 ($C_{1'}$); 162.6 (C_2); 164.7 (C_7); 174.5 (C_4). MS (ESI, *m/z*): 267.1 $[M-H]^-$. HPLC (method B, t_R, min): 23.82.

Methyl [(2-methyl-4-oxo-3-phenoxy-4*H*-chromen-7-yl)oxy] acetate (9). Following the general procedure 4, compound **9** was obtained from hydroxychromone **8** (33 mg, 0.12 mmol) and methyl 2-

bromoacetate (0.05 mL, 0.43 mmol, 3.5 equiv) in 60% yield. Chromatography: hexane to hexane/ethyl acetate, 7:3.

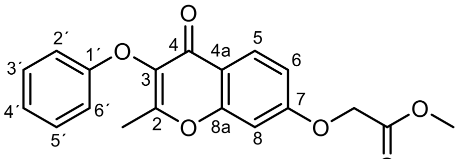

9

mp: 169-171 ºC. R_f: 0.38 (hexane/ethyl acetate, 7:3). IR (ATR): ν 1760, 1650 (C=O); 1234 (C-O-C). ^{1}H-NMR (methanol-d_4, 700 MHz): δ 2.42 (s, 3H, C_2CH_3); 3.82 (s, 3H, OCH_3); 4.92 (s, 2H, CH_2); 6.94 (d, *J* = 8.6, 2H, $H_{2'}$, $H_{6'}$); 7.02 (t, *J* = 7.4, 1H, $H_{4'}$); 7.12-7.14 (m, 2H, H_6, H_8); 7.27-7.30 (m, 2H, $H_{3'}$, $H_{5'}$); 8.06 (d, *J* = 9.2, 1H, H_5). ^{13}C-NMR (methanol-d_4, 175 MHz): δ 15.7 ($C_2\underline{C}H_3$); 52.8 (OCH_3); 66.3 (CH_2); 102.7 (C_8); 115.8 ($C_{2'}$, $C_{6'}$); 116.1 (C_6); 119.4 (C_{4a}); 123.4 ($C_{4'}$); 128.0 (C_5); 130.7 ($C_{3'}$, $C_{5'}$); 137.3 (C_3); 158.7 ($C_{1'}$); 158.8 (C_{8a}); 163.1 (C_2); 164.2 (C_7); 170.3 (C=O); 174.3 (C_4). MS (ESI, *m/z*): 341.1 $[M+H]^+$. HPLC (method B, t_R, min): 24.50.

2.3.2. Synthesis of intermediates 14-29 (Scheme 1)

2.3.2.1. Synthesis of arylethanones 14-17

2-(3-Chlorophenoxy)-1-(2,4-dihydroxyphenyl)ethanone (14). Following the general procedure 1, arylethanone **14** was obtained from 3-chlorophenoxyacetic acid (1.1 g, 5.36 mmol) in 22% yield. Chromatography: hexane/DCM, 9:1 to DCM.

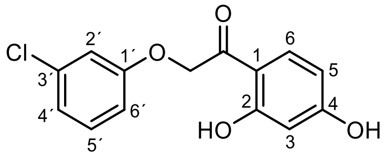

14

mp: 168-170 ºC. R_f: 0.51 (hexane/DCM, 6:4). IR (ATR): ν 3181 (OH); 1583 (C=O); 1258, 1259 (C-O-C). ^{1}H-NMR (methanol-d_4, 300 MHz): δ 5.36 (s, 2H, CH_2); 6.31 (d, *J* = 2.5, 1H, H_3); 6.41 (dd, *J* = 9.0, 2.5, 1H, H_5); 6.90 (ddd, *J* = 9.0, 2.5, 1.0, 1H, $H_{6'}$); 6.97 (ddd, *J* = 8.0, 2.0, 1.0, 1H, $H_{4'}$); 7.01 (t,

J = 2.0, 1H, $H_{2'}$); 7.25 (t, J = 8.0, 1H, $H_{5'}$); 7.78 (d, J = 9.0, 1H, H_6). ^{13}C-NMR (methanol-d_4, 75 MHz): δ 70.8 (CH_2); 103.8 (C_3); 109.7 (C_5); 112.3 (C_1); 114.2 ($C_{6'}$); 116.3 ($C_{2'}$); 122.4 ($C_{4'}$); 131.6 ($C_{5'}$); 132.8 (C_6); 135.9 ($C_{3'}$); 160.6 ($C_{1'}$); 166.2 (C_4); 167.1 (C_2); 198.5 (C=O). MS (ESI, *m/z*): 279.0 [M(^{35}Cl)+H]$^+$, 281.0 [M(^{37}Cl)+H]$^+$. HPLC (method B, t_R, min): 20.58.

2-(4-Chlorophenoxy)-1-(2,4-dihydroxyphenyl)ethanone (15). Following the general procedure 1, arylethanone **15** was obtained from 4-chlorophenoxyacetic acid (500 mg, 2.68 mmol) in 20% yield. Chromatography: hexane to hexane/ethyl acetate, 7:3.

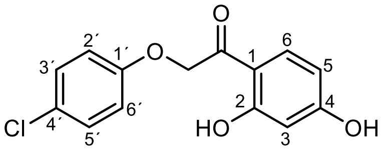

15

mp: 181-183 ºC. R_f: 0.71 (hexane/ethyl acetate, 7:3). IR (ATR): ν 3321 (OH); 1629 (C=O); 1233 (C-O-C). ^{1}H-NMR (methanol-d_4, 300 MHz): δ 5.33 (s, 2H, CH_2); 6.31 (d, J = 2.3, 1H, H_3); 6.41 (dd, J = 8.9, 2.4, 1H, H_5); 6.93-6.97 (m, 2H, $H_{2'}$, $H_{6'}$); 7.23-7.28 (m, 2H, $H_{3'}$, $H_{5'}$); 7.78 (d, J = 8.9, 1H, H_6). ^{13}C-NMR (methanol-d_4, 75 MHz): δ 70.9 (CH_2); 103.8 (C_3); 109.6 (C_5); 112.4 (C_1); 117.3 ($C_{2'}$, $C_{6'}$); 127.2 ($C_{4'}$); 130.3 ($C_{3'}$, $C_{5'}$); 132.8 (C_6); 158.5 ($C_{1'}$); 166.2 (C_4); 167.0 (C_2); 198.7 (C=O). MS (ESI, *m/z*): 277.0 [M(^{35}Cl)-H]$^-$, 279.0 [M(^{37}Cl)-H]$^-$. HPLC (method B, t_R, min): 27.20.

2-(3,4-Dichlorophenoxy)-1-(2,4-dihydroxyphenyl)ethanone (16). Following the general procedure 1, arylethanone **16** was obtained from 3,4-dichlorophenoxyacetic acid (1.1 g, 4.52 mmol) in 25% yield. Chromatography: hexane/DCM, 9:1 to DCM.

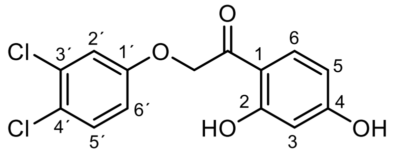

16

mp: 217-218 ºC. R_f: 0.63 (hexane/ethyl acetate, 6:4). IR (ATR): ν 3260 (OH); 1631 (C=O); 1224 (C-O-C). ^{1}H-NMR (methanol-d_4, 300 MHz): δ 5.39 (s, 2H, CH_2); 6.31 (d, J = 2.5, 1H, H_3); 6.42 (dd, J =

9.0, 2.5, 1H, H_5); 6.92 (dd, J = 9.0, 3.0, 1H, $H_{6'}$); 7.16 (d, J = 3.0, 1H, $H_{2'}$); 7.40 (d, J = 9.0, 1H, $H_{5'}$); 7.77 (d, J = 9.0, 1H, H_6). ^{13}C-NMR (methanol-d_4, 75 MHz): δ 71.0 (CH_2); 103.8 (C_3); 109.6 (C_5); 112.4 (C_1); 116.0 ($C_{6'}$); 118.0 ($C_{2'}$); 125.3 ($C_{4'}$); 131.9 ($C_{5'}$); 132.8 (C_6); 133.7 ($C_{3'}$); 159.1 ($C_{1'}$); 166.2 (C_4); 167.0 (C_2); 198.1 (C=O). MS (ESI, *m/z*): 313.1, 315.1, 317.1 $[M+H]^+$. HPLC (method B, t_R, min): 22.37.

2-(2,4-Dichlorophenoxy)-1-(2,4-dihydroxyphenyl)ethanone (17). Following the general procedure 1, arylethanone **17** was obtained from 2,4-dichlorophenoxyacetic acid (508 mg, 2.30 mmol) in 21% yield. Chromatography: hexane to DCM.

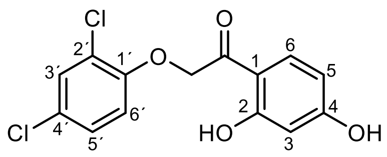

17

mp: 190-192 ºC. R_f: 0.41 (hexane/ethyl acetate, 7:3). IR (ATR): ν 3352 (OH); 1628 (C=O); 1233 (C-O-C). ^{1}H-NMR (methanol-d_4, 500 MHz): δ 5.45 (s, 2H, CH_2); 6.31 (d, J = 2.3, 1H, H_3); 6.41 (dd, J = 8.9, 2.3, 1H, H_5); 6.95 (d, J = 8.9, 1H, $H_{6'}$); 7.22 (dd, J = 8.8, 2.6, 1H, $H_{5'}$); 7.43 (d, J = 2.5, 1H, $H_{3'}$); 7.79 (d, J = 8.8, 1H, H_6). ^{13}C-NMR (methanol-d_4, 125 MHz): δ 71.8 (CH_2); 103.8 (C_3); 109.6 (C_5); 112.4 (C_1); 116.1 ($C_{6'}$); 124.7, 127.3 ($C_{2'}$, $C_{4'}$); 128.8 ($C_{5'}$); 130.8 ($C_{3'}$); 132.9 (C_6); 154.4 ($C_{1'}$); 166.2, 167.0 (C_2, C_4); 198.0 (C=O). MS (ESI, *m/z*): 311.0, 313.0, 315.0 $[M-H]^-$. HPLC (method B, t_R, min): 26.82.

2.3.2.2. Synthesis of acetoxychromones 18-21

3-(3-Chlorophenoxy)-2-methyl-4-oxo-4*H*-chromen-7-yl acetate (18). Following the general procedure 2, chromone **18** was obtained from arylethanone **14** (90 mg, 0.32 mmol) in quantitative yield.

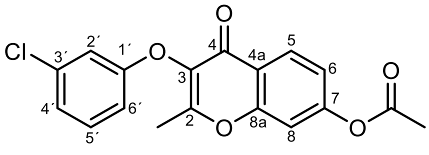

18

<u>mp</u>: 161-163 ºC. <u>R_f</u>: 0.46 (hexane/ethyl acetate, 7:3). <u>IR (ATR)</u>: ν 1768, 1654 (C=O); 1236, 1189 (C-O-C). <u>^{1}H-NMR ($CDCl_3$, 300 MHz)</u>: δ 2.36 (s, 3H, $COCH_3$); 2.42 (s, 3H, C_2CH_3); 6.85 (dd, *J* = 8.3, 2.4, 1H, $H_{6'}$); 6.90-6.94 (m, 1H, $H_{2'}$); 7.01 (d, *J* = 8.0, 1H, $H_{4'}$); 7.15 (dd, *J* = 8.8, 2.1, 1H, H_6); 7.19 (t, *J* = 8.2, 1H, $H_{5'}$); 7.32 (d, *J* = 2.0, 1H, H_8); 8.23 (d, *J* = 8.5, 1H, H_5). <u>^{13}C-NMR ($CDCl_3$, 75 MHz)</u>: δ 15.9 ($C_2\underline{C}H_3$); 21.3 ($CO\underline{C}H_3$); 111.1 (C_8); 113.5 ($C_{6'}$); 115.6 ($C_{2'}$); 119.5 (C_6); 122.1 (C_{4a}); 122.9 ($C_{4'}$); 127.7 (C_5); 130.6 ($C_{5'}$); 135.2 ($C_{3'}$); 136.5 (C_3); 154.6, 156.0 (C_7, C_{8a}); 157.8 ($C_{1'}$); 161.1 (C_2); 168.7 (C=O); 171.8 (C_4). <u>MS (ESI, *m/z*)</u>: 345.1 $[M(^{35}Cl)+H]^+$, 347.0 $[M(^{37}Cl)+H]^+$. <u>HPLC (method B, t_R, min)</u>: 27.87.

3-(4-Chlorophenoxy)-2-methyl-4-oxo-4*H*-chromen-7-yl acetate (19). Following the general procedure 2, chromone **19** was obtained from arylethanone **15** (60 mg, 0.22 mmol) in quantitative yield.

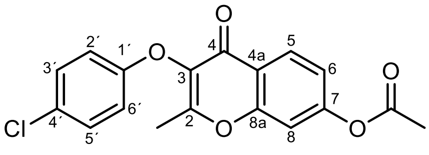

19

<u>mp</u>: 134-136 ºC. <u>R_f</u>: 0.81 (hexane/ethyl acetate, 7:3). <u>IR (ATR)</u>: ν 1769, 1617 (C=O); 1237 (C-O-C). <u>^{1}H-NMR (methanol-d_4, 500 MHz)</u>: δ 2.34 (s, 3H, $COCH_3$); 2.45 (s, 3H, C_2CH_3); 6.94-6.97 (m, 2H, $H_{2'}$, $H_{6'}$); 7.26-7.29 (m, 3H, $H_{3'}$, $H_{5'}$, H_6); 7.49 (d, *J* = 2.1, 1H, H_8); 8.15 (d, *J* = 8.7, 1H, H_5). <u>^{13}C-NMR (methanol-d_4, 125 MHz)</u>: δ 15.8 ($C_2\underline{C}H_3$); 20.9 ($CO\underline{C}H_3$); 112.5 (C_8); 117.5 ($C_{2'}$, $C_{6'}$); 121.1 (C_6); 122.8 (C_{4a}); 127.8 (C_5); 128.4 ($C_{4'}$); 130.6 ($C_{3'}$, $C_{5'}$); 137.5 (C_3); 156.6 (C_7); 157.3 (C_{8a}); 157.6 ($C_{1'}$); 163.9 (C_2); 170.1 (C=O); 173.9 (C_4). <u>MS (ESI, *m/z*)</u>: 345.0 $[M(^{35}Cl)+H]^+$, 347.1 $[M(^{37}Cl)+H]^+$. <u>HPLC (method B, t_R, min)</u>: 28.23.

3-(3,4-Dichlorophenoxy)-2-methyl-4-oxo-4*H*-chromen-7-yl acetate (20). Following the general procedure 2, chromone **20** was obtained from arylethanone **16** (90 mg, 0.29 mmol) in 83% yield.

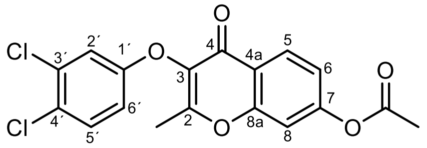

20

mp: 148-150 ºC. R_f: 0.43 (hexane/ethyl acetate, 7:3). IR (ATR): ν 1768, 1618 (C=O); 1188 (C-O-C). ^{1}H-NMR ($CDCl_3$, 300 MHz): δ 2.37 (s, 3H, $COCH_3$); 2.44 (s, 3H, C_2CH_3); 6.83 (dd, *J* = 9.0, 3.0, 1H, $H_{6'}$); 7.03 (d, *J* = 3.0, 1H, $H_{2'}$); 7.16 (dd, *J* = 8.7, 2.0, 1H, H_6); 7.33 (d, *J* = 2.1, 1H, H_8); 7.34 (d, *J* = 9.0, 1H, $H_{5'}$); 8.22 (d, *J* = 8.7, 1H, H_5). ^{13}C-NMR ($CDCl_3$, 75 MHz): δ 15.9 ($C_2\underline{C}H_3$); 21.3 ($CO\underline{C}H_3$); 111.1 (C_8); 115.0 ($C_{6'}$); 117.2 ($C_{2'}$); 119.7 (C_6); 122.1 (C_{4a}); 126.1 ($C_{4'}$); 127.7 (C_5); 131.1 ($C_{5'}$); 133.3 ($C_{3'}$); 136.5 (C_3); 154.7 (C_7); 156.0 (C_{8a}); 156.1 ($C_{1'}$); 161.1 (C_2); 168.7 (C=O); 171.6 (C_4). MS (ESI, *m/z*): 378.8, 381.0, 383.0 $[M+H]^+$. HPLC (method B, t_R, min): 11.56.

3-(2,4-Dichlorophenoxy)-2-methyl-4-oxo-4*H*-chromen-7-yl acetate (21). Following the general procedure 2, chromone **21** was obtained from arylethanone **17** (53 mg, 0.17 mmol) in quantitative yield.

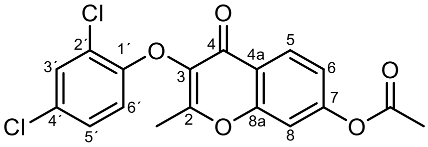

21

mp: 158-160 ºC. R_f: 0.49 (hexane/ethyl acetate, 7:3). IR (ATR): ν 1764, 1651 (C=O); 1206 (C-O-C). ^{1}H-NMR ($CDCl_3$, 300 MHz): δ 2.37 (s, 3H, $COCH_3$); 2.47 (s, 3H, C_2CH_3); 6.65 (d, *J* = 8.8, 1H, $H_{6'}$); 7.07 (dd, *J* = 8.8, 2.5, 1H, $H_{5'}$); 7.15 (dd, *J* = 8.7, 2.1, 1H, H_6); 7.32 (d, *J* = 2.1, 1H, H_8); 7.44 (d, *J* = 2.5, 1H, $H_{3'}$); 8.21 (d, *J* = 8.7, 1H, H_5). ^{13}C-NMR ($CDCl_3$, 75 MHz): δ 15.9 (C_2CH_3); 21.3 ($COCH_3$); 111.1 (C_8); 115.6 ($C_{6'}$); 119.6 (C_6); 122.1 (C_{4a}); 123.7 ($C_{2'}$); 127.6 (C_5); 127.7 ($C_{5'}$); 127.9 ($C_{4'}$);

130.5 ($C_{3'}$); 137.0 (C_3); 151.6 ($C_{1'}$); 154.7 (C_7); 156.0 (C_{8a}); 161.0 (C_2); 168.7 (C=O); 171.3 (C_4). MS (ESI, *m/z*): 379.0, 381.0, 383.0 $[M+H]^+$. HPLC (method B, t_R, min): 27.32.

2.3.2.3. Synthesis of hydroxychromones 22-25

3-(3-Chlorophenoxy)-7-hydroxy-2-methyl-4*H*-chromen-4-one (22). Following the general procedure 3, hydroxychromone **22** was obtained from acetoxychromone **18** (60 mg, 0.17 mmol) in quantitative yield.

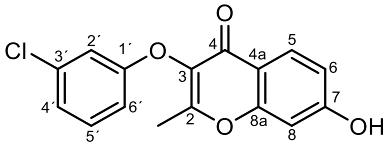

22

mp: 267-269 ºC. R_f: 0.13 (hexane/ethyl acetate, 7:3). IR (ATR): ν 3190 (OH); 1627 (C=O); 1247, 1183 (C-O-C). ^{1}H-NMR (methanol-*d*$_4$, 700 MHz): δ 2.41 (s, 3H, CH_3); 6.89 (ddd, J = 8.5, 2.5, 0.9, 1H, $H_{6'}$); 6.90 (d, J = 2.0, 1H, H_8); 6.95 (dd, J = 8.5, 2.5, 1H, H_6); 6.97 (t, J = 2.5, 1H, $H_{2'}$); 7.04 (dd, J = 8.0, 1.5, 1H, $H_{4'}$); 7.26 (t, J = 8.0, 1H, $H_{5'}$); 7.97 (d, J = 8.5, 1H, H_5). ^{13}C-NMR (methanol-*d*$_4$, 175 MHz): δ 15.6 (CH_3); 103.4 (C_8); 114.4 ($C_{6'}$); 116.5 ($C_{2'}$, C_6); 117.7 (C_{4a}); 123.5 ($C_{4'}$); 128.1 (C_5); 131.8 ($C_{5'}$); 136.1 ($C_{3'}$); 136.7 (C_3); 159.2 (C_{8a}); 159.5 ($C_{1'}$); 162.7 (C_2); 165.0 (C_7); 174.0 (C_4). MS (ESI, *m/z*): 301.0 $[M(^{35}Cl)\text{-}H]^-$, 303.0 $[M(^{37}Cl)\text{-}H]^-$. HPLC (method B, t_R, min): 20.59.

3-(4-Chlorophenoxy)-7-hydroxy-2-methyl-4*H*-chromen-4-one (23). Following the general procedure 3, hydroxychromone **23** was obtained from acetoxychromone **19** (61 mg, 0.18 mmol) in quantitative yield.

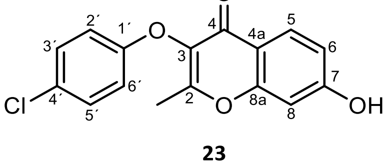

23

mp: >260 ºC (decomp.). R_f: 0.43 (hexane/ethyl acetate, 7:3). IR (ATR): ν 3393 (OH); 1651 (C=O). ^{1}H-NMR (methanol-d_4, 500 MHz): δ 2.33 (s, 3H, CH_3); 6.52 (d, J = 2.2, 1H, H_8); 6.69 (dd, J = 8.9, 2.2, 1H, H_6); 6.90-6.93 (m, 2H, $H_{2'}$, $H_{6'}$); 7.24-7.27 (m, 2H, $H_{3'}$, $H_{5'}$); 7.77 (d, J = 8.9, 1H, H_5). ^{13}C-NMR (methanol-d_4, 125 MHz): δ 15.5 (CH_3); 104.3 (C_8); 116.0 (C_{4a}); 117.4 ($C_{2'}$, $C_{6'}$); 121.5 (C_6); 126.8 (C_5); 127.9 ($C_{4'}$); 130.4 ($C_{3'}$, $C_{5'}$); 136.2 (C_3); 157.8 (C_{8a}); 160.7 ($C_{1'}$); 160.8 (C_2); 161.5 (C_7); 174.2 (C_4). MS (ESI, *m/z*): 301.0 [M(^{35}Cl)-H]$^-$, 303.0 [M(^{37}Cl)-H]$^-$. HPLC (method B, t_R, min): 26.85.

3-(3,4-Dichlorophenoxy)-7-hydroxy-2-methyl-4*H*-chromen-4-one (24). Following the general procedure 3, hydroxychromone **24** was obtained from acetoxychromone **20** (60 mg, 0.16 mmol) in 75% yield.

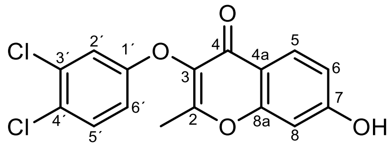

24

mp: 267-269 ºC. R_f: 0.45 (hexane/ethyl acetate,7:3). IR (ATR): ν 3317 (OH); 1643 (C=O); 1247 (C-O-C). ^{1}H-NMR (methanol-d_4, 700 MHz): δ 2.42 (s, 3H, CH_3); 6.90 (d, J = 1.5, 1H, H_8); 6.91 (dd, J = 8.5, 2.6, 1H, $H_{6'}$); 6.95 (dd, J = 8.8, 2.2, 1H, H_6); 7.14 (d, J = 2.9, 1H, $H_{2'}$); 7.42 (d, J = 8.9, 1H, $H_{5'}$); 7.97 (d, J = 8.8, 1H, H_5). ^{13}C-NMR (methanol-d_4, 175 MHz): δ 15.6 (CH_3); 103.4 (C_8); 116.2 ($C_{6'}$); 116.5 (C_6); 117.7 (C_{4a}); 118.3 ($C_{2'}$); 126.5 ($C_{4'}$); 128.1 (C_5); 132.1 ($C_{5'}$); 133.9 ($C_{3'}$); 136.7 (C_3); 158.0 ($C_{1'}$); 159.2 (C_{8a}); 162.8 (C_2); 165.1 (C_7); 173.8 (C_4). MS (ESI, *m/z*): 335.0, 337.0, 339.0 [M-H]$^-$. HPLC (method B, t_R, min): 16.96.

3-(2,4-Dichlorophenoxy)-7-hydroxy-2-methyl-4*H*-chromen-4-one (25). Following the general procedure 3, hydroxychromone **25** was obtained from acetoxychromone **21** (115 mg, 0.30 mmol) in quantitative yield.

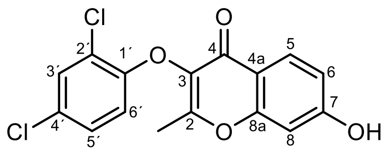

25

mp: 190-192 ºC. R_f: 0.29 (hexane/ethyl acetate, 7:3). IR (ATR): ν 3100 (OH); 1595 (C=O); 1268 (C-O-C). ^{1}H-NMR (methanol-d_4, 700 MHz): δ 2.42 (s, 3H, CH_3); 6.79 (d, J = 8.8, 1H, $H_{6'}$); 6.90 (d, J = 2.2, 1H, H_8); 6.94 (dd, J = 8.8, 2.2, 1H, H_6); 7.16 (dd, J = 8.8, 2.5, 1H, $H_{5'}$); 7.51 (d, J = 2.5, 1H, $H_{3'}$); 7.96 (d, J = 8.8, 1H, H_5). ^{13}C-NMR (methanol-d_4,175 MHz): δ 15.6 (CH_3); 103.4 (C_8); 116.5 (C_6); 116.7 ($C_{6'}$); 117.7 (C_{4a}); 124.2 ($C_{2'}$); 128.1 (C_5); 128.4 ($C_{4'}$); 128.9 ($C_{5'}$); 131.1 ($C_{3'}$); 136.9 (C_3); 153.2 ($C_{1'}$); 159.2 (C_{8a}); 162.6 (C_2); 165.1 (C_7); 173.5 (C_4). MS (ESI, *m/z*): 334.9, 336.9, 338.9 $[M-H]^-$. HPLC (method B, t_R, min): 25.95.

2.3.2.4. Synthesis of chromones 26-29

Methyl {[3-(3-chlorophenoxy)-2-methyl-4-oxo-4*H*-chromen-7-yl]oxy} acetate (26). Following the general procedure 4, compound **26** was obtained from hydroxychromone **22** (50 mg, 0.17 mmol) and methyl 2-bromoacetate (30 µL, 0.33 mmol, 1.9 equiv) in 97% yield.

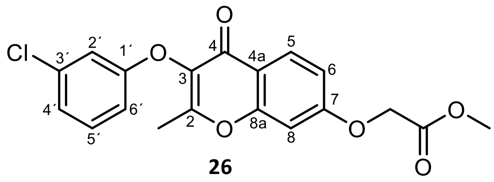

26

mp: 152-154 ºC. R_f: 0.28 (hexane/ethyl acetate, 8:2). IR (ATR): ν 1759, 1647 (C=O); 1226 (C-O-C). ^{1}H-NMR ($CDCl_3$, 300 MHz): δ 2.40 (s, 3H, C_2CH_3); 3.84 (s, 3H, OCH_3); 4.75 (s, 2H, OCH_2); 6.83 (ddd, J = 8.3, 2.5, 0.9, 1H, $H_{6'}$); 6.86 (d, J = 2.4, 1H, H_8); 6.92 (t, J = 2.2, 1H, $H_{2'}$); 6.98-7.02 (m, 1H, $H_{4'}$); 7.02 (dd, J = 8.1, 2.4, 1H, H_6); 7.20 (d, J = 8.1, 1H, $H_{5'}$); 8.14 (d, J = 8.9, 1H, H_5). ^{13}C-NMR ($CDCl_3$, 75 MHz): δ 15.8 ($C_2\underline{C}H_3$); 52.7 (OCH_3); 65.5 (CH_2); 101.5 (C_8); 113.6 ($C_{6'}$); 114.5 ($C_{2'}$); 115.5 (C_6); 119.0 (C_{4a}); 122.8 ($C_{4'}$); 128.0 (C_5); 130.5 ($C_{5'}$); 135.2 ($C_{3'}$); 136.3 (C_3); 157.1 (C_{8a});

158.0 ($C_{1'}$); 160.3 (C_2); 162.1 (C_7); 168.5 (C=O); 171.8 (C_4). MS (ESI, *m/z*): 375.0 $[M(^{35}Cl)+H]^+$, 377.0 $[M(^{37}Cl)+H]^+$. HPLC (method B, t_R, min): 20.71.

Methyl {[3-(4-chlorophenoxy)-2-methyl-4-oxo-4*H*-chromen-7-yl]oxy}acetate (27). Following the general procedure 4, compound **27** was obtained from hydroxychromone **23** (82 mg, 0.27 mmol) and methyl 2-bromoacetate (90 µL, 0.95 mmol, 3.5 equiv) in 50% yield. Chromatography: hexane to hexane/ethyl acetate, 1:1.

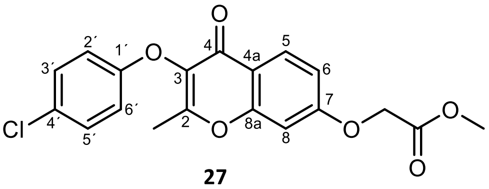

27

mp: 147-149 ºC. R_f: 0.48 (hexane/ethyl acetate, 7:3). IR (ATR): ν 1761, 1649 (C=O); 1237 (C-O-C). ^{1}H-NMR (methanol-d_4, 500 MHz): δ 2.42 (s, 3H, C_2CH_3); 3.81 (s, 3H, OCH_3); 4.92 (s, 2H, CH_2); 6.92-6.95 (m, 2H, $H_{2'}$, $H_{6'}$); 7.12-7.14 (m, 2H, H_6, H_8); 7.26-7.29 (m, 2H, $H_{3'}$, $H_{5'}$); 8.06 (d, J = 9.3, 1H, H_5). ^{13}C-NMR (methanol-d_4, 125 MHz): δ 15.7 ($C_2\underline{C}H_3$); 52.8 (OCH_3); 66.3 (CH_2); 102.7 (C_8); 116.1 (C_6); 117.5 ($C_{2'}$, $C_{6'}$); 119.4 (C_{4a}); 128.0 (C_5); 128.3 ($C_{4'}$); 130.5 ($C_{3'}$, $C_{5'}$); 137.3 (C_3); 157.4 ($C_{1'}$); 158.8 (C_{8a}); 163.2 (C_2); 164.3 (C_7); 170.3 (C=O); 174.0 (C_4). MS (ESI, *m/z*): 375.1 $[M(^{35}Cl)+H]^+$, 377.0 $[M(^{37}Cl)+H]^+$. HPLC (method B, t_R, min): 27.19.

Methyl {[3-(3,4-dichlorophenoxy)-2-methyl-4-oxo-4*H*-chromen-7-yl]oxy} acetate (28). Following the general procedure 4, compound **28** was obtained from hydroxychromone **24** (32 mg, 0.09 mmol) and methyl 2-bromoacetate (20 µL, 0.19 mmol, 2.1 equiv) in 98% yield.

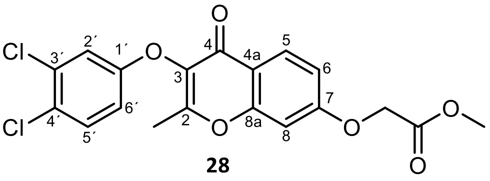

28

mp: 170-172 ºC. R_f: 0.22 (hexane/ethyl acetate, 8:2). IR (ATR): ν 1758, 1646 (C=O); 1223 (C-O-C).

^{1}H-NMR (CDCl$_3$, 300 MHz): δ 2.41 (s, 3H, C_2CH_3); 3.84 (s, 3H, OCH_3); 4.75 (s, 2H, CH_2); 6.82 (d, J = 9.0, 3.0, 1H, $H_{6'}$); 6.86 (d, J = 2.5, 1H, H_8); 7.02 (d, J = 3.0, 1H, $H_{2'}$); 7.02 (dd, J = 8.9, 2.5, 1H, H_6); 7.32 (d, J = 9.0, 1H, $H_{5'}$); 8.13 (d, J = 8.9, 1H, H_5). ^{13}C-NMR (CDCl$_3$, 75 MHz): δ 15.8 ($C_2\underline{C}H_3$); 52.7 (OCH_3); 65.4 (CH_2); 101.5 (C_8); 114.6 ($C_{6'}$); 115.0 (C_6); 117.2 ($C_{2'}$); 118.9 (C_{4a}); 126.0 ($C_{4'}$); 128.0 (C_5); 131.0 ($C_{5'}$); 133.3 ($C_{3'}$); 136.3 (C_3); 156.3 ($C_{1'}$); 157.1 (C_{8a}); 160.3 (C_2); 162.2 (C_7); 168.4 (C=O); 171.6 (C_4). MS (ESI, *m/z*): 409.0, 411.0, 413.0 $[M+H]^+$. HPLC (method B, t_R, min): 22.16.

Methyl {[3-(2,4-dichlorophenoxy)-2-methyl-4-oxo-4*H*-chromen-7-yl]oxy} acetate (29). Following the general procedure 4, compound **29** was obtained from hydroxychromone **25** (81 mg, 0.24 mmol) and methyl 2-bromoacetate (90 µL, 0.84 mmol, 3.5 equiv) in 51% yield. Chromatography: hexane to hexane/ethyl acetate, 7:3.

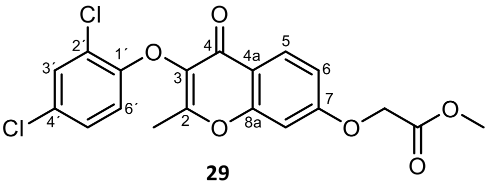

29

mp: 176-178 ºC. R_f: 0.33 (hexane/ethyl acetate, 7:3). IR (ATR): ν 1760, 1640 (C=O); 1256 (C-O-C). ^{1}H-NMR (CDCl$_3$, 500 MHz): δ 2.44 (s, 3H, C_2CH_3); 3.85 (s, 3H, OCH_3); 4.75 (s, 2H, CH_2); 6.65 (d, J = 8.8, 1H, $H_{6'}$); 6.86 (d, J = 2.4, 1H, H_8); 7.02 (dd, J = 8.9, 2.4, 1H, H_6); 7.06 (dd, J = 8.8, 2.5, 1H, $H_{5'}$); 7.43 (d, J = 2.5, 1H, $H_{3'}$); 8.13 (d, J = 8.9, 1H, H_5). ^{13}C-NMR (CDCl$_3$, 125 MHz): δ 15.8 ($C_2\underline{C}H_3$); 52.7 (OCH_3); 65.5 (CH_2); 101.5 (C_8); 114.6 (C_6); 115.6 ($C_{6'}$); 118.9 (C_{4a}); 123.6 ($C_{2'}$); 127.7 ($C_{5'}$); 127.7 ($C_{4'}$); 128.0 (C_5); 130.5 ($C_{3'}$); 136.8 (C_3); 151.8 ($C_{1'}$); 157.1 (C_{8a}); 160.2 (C_2); 162.2 (C_7); 168.4 (C=O); 171.4 (C_4). MS (ESI, *m/z*): 409.0, 411.0, 413.0 $[M+H]^+$. HPLC (method B, t_R, min): 26.08.

2.3.3. Synthesis of intermediates 31-35 (Scheme 2)

2-(2,4-Dichlorophenoxy)-1-(4-methoxyphenyl)ethanone (31). To a solution of 2,4-dichlorophenol (1.00 g, 6.13 mmol, 1 equiv) and 1,8-diazabiciclo[5.4.0]undeca-7-eno (DBU) (1.10 mL, 7.36 mmol, 1.2 equiv) in anhydrous *N,N*-dimethylformamide (DMF) (16 mL), 2-bromo-1-(4-methoxyphenyl)ethanone (1.69 g, 7.36 mmol, 1.2 equiv) was added and the mixture was heated at 140 °C for 30 min under MW irradiation. After cooling to rt, the reaction mixture was diluted with ethyl acetate and washed with water and brine. The organic layer was dried over Na_2SO_4, filtered, and concentrated under reduced pressure. The residue was purified by flash chromatography (hexane/ethyl acetate, 9:1 to hexane/ethyl acetate, 1:1) to yield pure compound **31** in 80% yield.

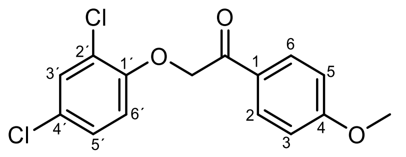

31

mp: 110-112 ºC. R_f: 0.40 (hexane/ethyl acetate, 5:1). IR (ATR): ν 1691 (C=O); 1231 (C-O-C). ^{1}H-NMR ($CDCl_3$, 300 MHz): δ 3.88 (s, 3H, CH_3); 5.27 (s, 2H, CH_2); 6.77 (d, J = 8.8, 1H, $H_{6'}$); 6.96 (d, J = 9.0, 2H, H_3, H_5); 7.12 (dd, J = 8.8, 2.5, 1H, $H_{5'}$); 7.37 (d, J = 2.5, 1H, $H_{3'}$); 7.99 (d, J = 9.0, 2H, H_2, H_6). ^{13}C-NMR ($CDCl_3$, 75 MHz): δ 55.7 (CH_3); 72.0 (CH_2); 114.2 (C_3, C_5); 115.0 ($C_{6'}$); 124.2, 126.9, 127.4 (C_1, $C_{2'}$, $C_{4'}$); 127.7 ($C_{5'}$); 130.4 ($C_{3'}$); 130.8 (C_2, C_6); 152.8 ($C_{1'}$); 164.4 (C_4); 192.3 (C=O). MS (ESI, *m/z*): 311.1, 313.0, 315.1 $[M+H]^+$. HPLC (method A, t_R, min): 17.81.

2-(2,4-Dichlorophenoxy)-3-(dimethylamino)-1-(4-methoxyphenyl)but-2-en-1-one (32). A solution of arylethanone **31** (263 mg, 0.85 mmol, 1 equiv) in 1,1-dimethoxy-*N,N*-dimethylethanamine (0.5 mL, 3.57 mmol, 4.2 equiv) was stirred at 90 °C for 5 h. After cooling to rt, the reaction mixture was concentrated under reduced pressure and the crude was purified by flash chromatography (hexane/ethyl acetate, 1:1 to ethyl acetate) to afford enaminone **32** in 52% yield.

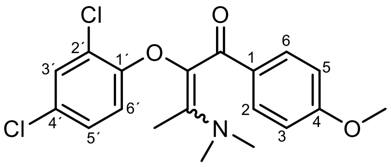

32

Rf: 0.30 (ethyl acetate). IR (ATR): ν 1741 (C=O). ^{1}H-NMR ($CDCl_3$, 300 MHz): δ Mixture of isomers A:B (1:1): 2.04 (s, 3H, CH_{3A}); 2.40 (s, 3H, CH_{3B}); 2.99 (s, 6H, $N(CH_3)_{2B}$); 3.02 (s, 6H, $N(CH_3)_{2A}$); 3.78 (s, 3H, OCH_3); 6.74-6.77 (m, 3H, H_3, H_5, $H_{6'B}$); 6.80 (d, J = 9.0, 1H, $H_{6'A}$); 6.99 (dd, J = 8.9, 2.5, 1H, $H_{5'A}$); 7.00 (dd, J = 8.9, 2.5, 1H, $H_{5'B}$); 7.19 (app t, J = 2.2, 1H, $H_{3'}$); 7.73 (d, J = 8.9, 2H, H_{2B}, H_{6B}); 7.77 (d, J = 8.9, 2H, H_{2A}, H_{6A}).

4-(2,4-Dichlorophenoxy)-5-methyl-3-(4-methoxyphenyl)-1*H*-pyrazole (33). Following the general procedure 5, pyrazole **33** was obtained from enaminone **32** (154 mg, 0.41 mmol) in 44% yield. Chromatography: hexane/ethyl acetate, 4:1 to hexane/ethyl acetate, 1:1.

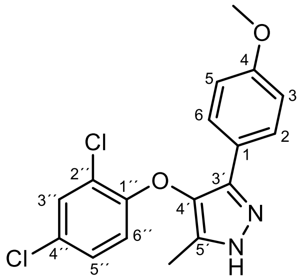

33

Rf: 0.16 (hexane/ethyl acetate, 1:1). IR (ATR): ν 3133 (NH); 1251 (C-O-C). ^{1}H-NMR ($CDCl_3$, 500 MHz): δ 2.13 (s, 3H, $C_{5'}CH_3$); 3.79 (s, 3H, OCH_3); 6.60 (d, J = 8.9, 1H, $H_{6''}$); 6.85-6.87 (m, 2H, H_3, H_5); 7.00 (dd, J = 8.9, 2.5, 1H, $H_{5''}$); 7.42 (d, J = 2.5, 1H, $H_{3''}$); 7.60-7.63 (m, 2H, H_2, H_6). ^{13}C-NMR ($CDCl_3$, 125 MHz): δ 9.7 ($C_{5'}\underline{C}H_3$); 55.4 (OCH_3); 114.5 (C_3, C_5); 115.6 ($C_{6''}$); 122.1 (C_1); 123.3 ($C_{2''}$); 127.26 (C_2, C_6); 127.32 ($C_{4''}$); 127.9 ($C_{5''}$); 130.4 ($C_{3''}$); 132.9, 137.0 ($C_{4'}$, $C_{5'}$); 138.3 ($C_{3'}$); 152.7 ($C_{1''}$); 159.9 (C_4). HRMS (ESI, *m/z*): calculated for $C_{17}H_{13}Cl_2N_2O_2$ $[M-H]^-$: 347.0360, found: 347.0357. HPLC (method A, t_R, min): 20.54.

4-[4-(2,4-Dichlorophenoxy)-5-methyl-1*H*-pyrazol-3-yl]phenol (34). To a solution of pyrazole **33** (49 mg, 0.14 mmol, 1 equiv) in anhydrous DCM (3.0 mL) at -78°C, BBr_3 (105 mg, 0.42 mmol, 3 equiv) was added and the mixture was stirred at -78°C for 1 h. After this time, the reaction was warmed up to rt and stirred at this temperature for 20 h. Then, the mixture was washed with water and brine, dried over Na_2SO_4, filtered and concentrated under reduced pressure. The crude was purified by flash chromatography (hexane/ethyl acetate, 1:1 to 3:7) to yield pure compound **34** in 70% yield.

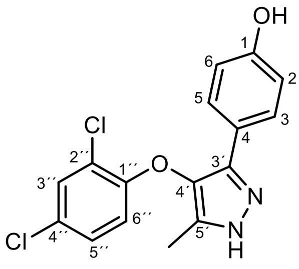

34

R_f

R_f: 0.54 (hexane/ethyl acetate, 1:3). IR (ATR): ν 3267 (OH, NH); 1251 (C-O-C). ^{1}H-NMR ($CDCl_3$, 500 MHz): δ 2.14 (s, 3H, CH_3); 6.61 (d, J = 8.9, 1H, $H_{6''}$); 6.80 (d, J = 8.7, 2H, H_2, H_6); 7.02 (dd, J = 8.8, 2.5, 1H, $H_{5''}$); 7.42 (d, J = 2.4, 1H, $H_{3''}$); 7.54 (d, J = 8.6, 2H, H_3, H_5). ^{13}C-NMR ($CDCl_3$, 125 MHz): δ 9.7 (CH_3); 115.6 ($C_{6''}$); 116.0 (C_2, C_6); 122.2 (C_4); 123.3 ($C_{4''}$); 127.4 ($C_{2''}$); 127.5 (C_3, C_5); 127.9 ($C_{5''}$); 130.4 ($C_{3''}$); 133.0, 136.9 ($C_{4'}$, $C_{5'}$); 138.6 ($C_{3'}$); 152.7 ($C_{1''}$); 156.1 (C_1). HRMS (ESI, *m/z*): calculated for $C_{16}H_{11}Cl_2N_2O_2$ [M-H]$^-$: 333.0203; found: 333.0198. HPLC (method A, t_R, min): 17.81.

Methyl {4-[4-(2,4-dichlorophenoxy)-5-methyl-1*H*-pyrazol-3-yl]phenoxy}acetate (35). To a solution of pyrazole **34** (31 mg, 0.09 mmol, 1 equiv) in anhydrous DMF (2.0 mL), K_2CO_3 (26 mg, 0.19 mmol, 2 equiv) and methyl 2-bromoacetate (9 µL, 0.09 mmol, 1 equiv) were added at -20°C. After stirring 4 h at this temperature, the reaction was warmed up to rt and stirred overnight. Then, another portion of methyl 2-bromoacetate (2 µL, 0.02 mmol, 0.2 equiv) was added and the mixture was stirred for 4 h. The reaction was diluted with ethyl acetate, washed with water and brine, dried

over Na_2SO_4, filtered and concentrated under reduced pressure. The crude was purified by flash chromatography (toluene/acetone, 10:1 to toluene/acetone, 3:1) to yield pure compound **35** in 32% yield.

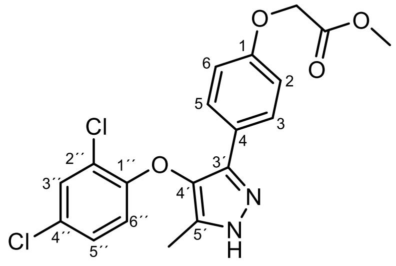

35

Rf: 0.49 (toluene/acetone, 1:1). IR (ATR): ν 3133 (NH); 1761 (C=O); 1250 (C-O-C). ^{1}H-NMR ($CDCl_3$, 500 MHz): δ 2.14 (s, 3H, $C_{5'}CH_3$); 3.79 (s, 3H, OCH_3); 4.62 (s, 2H, CH_2); 6.61 (d, *J* = 8.9, 1H, $H_{6''}$); 6.88 (d, *J* = 9.0, 2H, H_2, H_6); 7.02 (dd, *J* = 8.9, 2.5, 1H, $H_{5''}$); 7.42 (d, *J* = 2.5, 1H, $H_{3''}$); 7.63 (d, *J* = 9.0, 2H, H_3, H_5). ^{13}C-NMR ($CDCl_3$, 125 MHz): δ 9.7 ($C_{5'}CH_3$); 52.5 (OCH_3); 65.4 (CH_2); 115.2 (C_2, C_6); 115.6 ($C_{6''}$); 123.3 ($C_{2''}$); 123.5 (C_4); 127.3 (C_3, C_5); 127.4 ($C_{4''}$); 127.9 ($C_{5''}$); 130.4 ($C_{3''}$); 133.1, 136.5 ($C_{4'}$, $C_{5'}$); 138.6 ($C_{3'}$); 152.7 ($C_{1''}$); 158.0 (C_1); 169.3 (C=O). HRMS (ESI, *m/z*): calculated for $C_{19}H_{15}Cl_2N_2O_4$ $[M-H]^-$: 405.0414, found: 405.0410. HPLC (method A, t_R, min): 12.86.

2.3.4. Synthesis of intermediates 39-41 (Scheme 3)

Methyl 3-((3-(2,4-dichlorophenoxy)-2-methyl-4-oxo-4*H*-chromen-7-yl)oxy) propanoate (39). A mixture of hydroxychromone **25** (130 mg, 0.38 mmol, 1 equiv), *N,N*-dimethylpyridin-4-amine (14 mg, 0.12 mmol, 0.3 equiv) and methyl acrylate (0.70 mL, 7.71 mmol, 20 equiv) was stirred under MW irradiation (150 °C, 2.5 h). After this time, the mixture was dissolved with ethyl acetate and washed with brine, dried over Na_2SO_4, filtered and concentrated under reduced pressure. The crude was purified by flash chromatography (hexane to hexane/ethyl acetate, 8:2) to yield pure compound **39** in 10% yield.

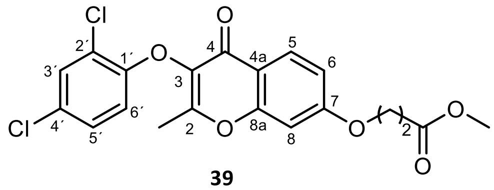

39

mp: 138-140 ºC. R_f: 0.42 (hexane/ethyl acetate, 7:3). IR (ATR): ν 1734, 1637 (C=O); 1254 (C-O-C). ^{1}H-NMR ($CDCl_3$, 300 MHz): δ 2.44 (s, 3H, C_2CH_3); 2.88 (t, *J* = 6.3, 2H, CH_2CO); 3.74 (s, 3H, OCH_3); 4.36 (t, *J* = 6.3, 2H, OCH_2); 6.66 (d, *J* = 8.8, 1H, $H_{6'}$); 6.90 (d, *J* = 2.3, 1H, H_8); 6.98 (dd, *J* = 8.9, 2.3, 1H, H_6); 7.07 (dd, *J* = 8.8, 2.5, 1H, $H_{5'}$); 7.43 (d, *J* = 2.5, 1H, $H_{3'}$); 8.10 (d, *J* = 8.9, 1H, H_5). ^{13}C-NMR ($CDCl_3$, 75 MHz): δ 15.8 ($C_2\underline{C}H_3$); 34.2 (CH_2CO); 52.2 (OCH_3); 64.2 (OCH_2); 101.0 (C_8); 115.0 (C_6); 115.6 ($C_{6'}$); 118.3 (C_{4a}); 123.6 ($C_{2'}$); 127.6 (C_5); 127.7 ($C_{5'}$, $C_{4'}$); 130.4 ($C_{3'}$); 136.7 (C_3); 151.8 ($C_{1'}$); 157.3 (C_{8a}); 160.1 (C_2); 163.2 (C_7); 171.1 (C_4, C=O). MS (ESI, *m/z*): 422.6, 424.6, 426.6 $[M+H]^+$. HPLC (method B, t_R, min): 15.27.

Methyl 4-{[3-(2,4-dichlorophenoxy)-2-methyl-4-oxo-4*H*-chromen-7-yl]oxy} butanoate (40). Following the general procedure 4, compound **40** was obtained from hydroxychromone **25** (40 mg, 0.12 mmol) and methyl 4-bromobutanoate (0.02 mL, 0.13 mmol, 1.1 equiv) in 80% yield. Chromatography: hexane/ethyl acetate, 9:1 to hexane/ethyl acetate, 8:2.

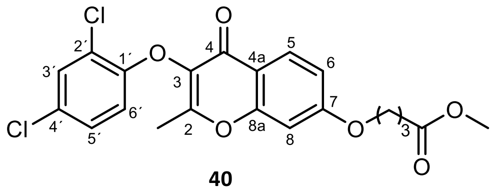

40

mp: 142-144 ºC. R_f: 0.72 (hexane/ethyl acetate, 1:1). IR (ATR): ν 1736, 1646 (C=O); 1251 (C-O-C). ^{1}H-NMR ($CDCl_3$, 300 MHz): δ 2.13-2.22 (m, 2H, CH_2); 2.43 (s, 3H, C_2CH_3); 2.56 (t, *J* = 7.2, 2H, CH_2CO); 3.71 (s, 3H, OCH_3); 4.12 (t, *J* = 6.1, 2H, OCH_2); 6.66 (d, *J* =8.8, 1H, $H_{6'}$); 6.86 (d, *J* = 2.3, 1H, H_8); 6.95 (dd, *J* = 8.9, 2.3, 1H, H_6); 7.06 (dd, *J* = 8.8, 2.5, 1H, $H_{5'}$); 7.42 (d, *J* = 2.5, 1H, $H_{3'}$); 8.03 (d, *J* = 8.9, 1H, H_5). ^{13}C-NMR ($CDCl_3$, 75 MHz): δ 15.8 ($C_2\underline{C}H_3$); 24.5 (CH_2); 30.5 (CH_2CO); 52.0 (OCH_3); 67.7 (OCH_2); 100.8 (C_8); 115.0 (C_6); 115.6 ($C_{6'}$); 118.1 (C_{4a}); 123.6 ($C_{2'}$); 127.5 (C_5);

127.6 ($C_{5'}$); 127.7 ($C_{4'}$); 130.4 ($C_{3'}$); 136.7 (C_3); 151.8 ($C_{1'}$); 157.3 (C_{8a}); 160.0 (C_2); 163.4 (C_7); 171.5 (C_4); 173.5 (C=O). MS (ESI, *m/z*): 436.9, 439.0, 441.0 $[M+H]^+$. HPLC (method B, t_R, min): 18.58.

Methyl 5-((3-(2,4-dichlorophenoxy)-2-methyl-4-oxo-4*H*-chromen-7-yl)oxy) pentanoate (41). Following the general procedure 4, compound **41** was obtained from hydroxychromone **25** (144 mg, 0.43 mmol) and 5-bromopentanoate (0.21 mL, 1.49 mmol, 3.5 equiv) in 82% yield. Chromatography: hexane/ethyl acetate, 9:1 to hexane/ethyl acetate, 6:4.

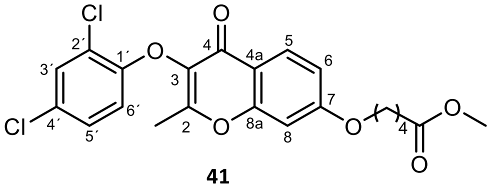

41

mp: 148-151 ºC. R_f: 0.52 (hexane/ethyl acetate, 1:1). IR (ATR): ν 1736, 1643 (C=O); 1251 (C-O-C). ^{1}H-NMR ($CDCl_3$, 300 MHz): δ 1.83-1.92 (m, 4H, $C\underline{H_2}C\underline{H_2}CH_2CO$); 2.40-2.45 (m, 2H, CH_2CO); 2.43 (s, 3H, C_2CH_3); 3.69 (s, 3H, OCH_3); 4.07 (t, *J* = 5.7, 2H, OCH_2); 6.66 (d, *J* = 8.8, 1H, $H_{6'}$); 6.85 (d, *J* = 2.3, 1H, H_8); 6.96 (dd, *J* = 8.9, 2.3, 1H, H_6); 7.06 (dd, *J* = 8.8, 2.5, 1H, $H_{5'}$); 7.43 (d, *J* = 2.5, 1H, $H_{3'}$); 8.08 (d, *J* = 8.9, 1H, H_5). ^{13}C-NMR ($CDCl_3$, 75 MHz): δ 15.8 ($C_2\underline{C}H_3$); 21.7, 28.5 ($2CH_2$); 33.7 ($\underline{C}H_2CO$); 51.8 (OCH_3); 68.3 (OCH_2); 100.8 (C_8); 115.0 (C_6); 115.6 ($C_{6'}$); 118.0 (C_{4a}); 123.6 ($C_{2'}$); 127.5 (C_5); 127.6 ($C_{4'}$); 127.7 ($C_{5'}$); 130.4 ($C_{3'}$); 136.7 (C_3); 151.9 ($C_{1'}$); 157.3 (C_{8a}); 160.0 (C_2); 163.6 (C_7); 171.5 (C_4); 173.8 (C=O). MS (ESI, *m/z*): 451.0, 453.0, 455.0 $[M+H]^+$. HPLC (method B, t_R, min): 18.44.

2.3.5. Synthesis of intermediate 46 (Scheme 4)

2-{[3-(2,4-Dichlorophenoxy)-2-methyl-4-oxo-4*H*-chromen-7-yl]oxy}acetamide (46). Following the general procedure 4, chromone **46** was obtained from hydroxychromone **25** (144 mg, 0.43 mmol)

and 2-bromoacetamide (0.21 mL, 1.49 mmol, 3.5 equiv) in 67% yield. Chromatography: hexane/ethyl acetate, 6:4 to ethyl acetate.

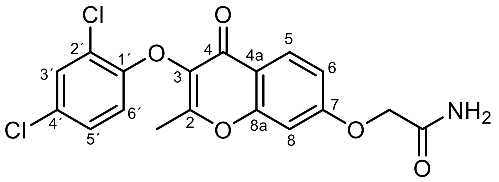

46

mp: 244-246 ºC. R_f: 0.75 (ethyl acetate/methanol, 9:1). IR (ATR): ν 3384, 3196 (NH); 1687, 1620 (C=O); 1255 (C-O-C). ^{1}H-NMR (methanol-d_4, 700 MHz): δ 2.45 (s, 3H, CH_3); 4.69 (s, 2H, CH_2); 6.81 (d, J = 8.8, 1H, $H_{6'}$); 7.17 (dd, J = 8.8, 2.5, 1H, H_6); 7.18 (d, J = 2.4, 1H, H_8); 7.20 (dd, J = 8.8, 2.4, 1H, $H_{5'}$); 7.52 (d, J = 2.5, 1H, $H_{3'}$); 8.07 (d, J = 8.8, 1H, H_5). ^{13}C-NMR (methanol-d_4,175 MHz): δ 15.6 (CH_3); 68.2 (CH_2); 102.8 (C_8); 116.4 (C_6); 116.7 ($C_{6'}$); 119.4 (C_{4a}); 124.3 ($C_{2'}$); 128.0 (C_5); 128.5 ($C_{4'}$); 128.9 ($C_{5'}$); 131.1 ($C_{3'}$); 137.3 (C_3); 153.1 ($C_{1'}$); 158.8 (C_{8a}); 163.1 (C_2); 164.0 (C_7); 172.7 (C=O); 173.3 (C_4). MS (ESI, *m/z*): 394.0, 396.0, 398.0 $[M+H]^+$. HPLC (method B, t_R, min): 20.54.

2.3.6. Synthesis of intermediates 50-52 (Scheme 5)

3-(2,4-Dichlorophenoxy)-7-hydroxy-4H-chromen-4-one (50). A mixture of chromone **17** (40 mg, 0.13 mmol, 1 equiv), methanesulfonyl chloride (20 µL, 0.23 mmol, 1.8 equiv) and boron trifluoride diethyl etherate complex (30 µL, 0.19 mmol, 1.5 equiv) in anhydrous DMF was stirred at 100 °C for 1.5 h. After cooling to rt, the solvent was evaporated and the residue was dissolved in ethyl acetate and washed with brine. The organic phase was dried over Na_2SO_4, filtered and concentrated under reduced pressure. The crude was purified by flash chromatography (hexane/ethyl acetate, 8:2 to hexane/ethyl acetate, 4:6) to yield pure compound **50** in 94% yield.

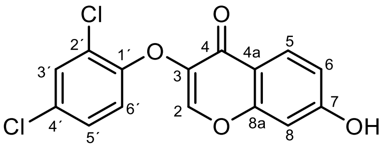

50

R_f: 0.60 (hexane/ethyl acetate 4:6). IR (ATR): ν 3091 (OH); 1594 (C=O); 1263 (C-O-C). ^{1}H-NMR (methanol-d_4, 300 MHz): δ 6.91 (d, *J* = 8.8, 1H, $H_{6'}$); 6.91 (d, *J* = 2.2, 1H, H_8); 6.97 (dd, *J* = 8.8, 2.2, 1H, H_6); 7.19 (dd, *J* = 8.9, 2.5, 1H, $H_{5'}$); 7.50 (d, *J* = 2.5, 1H, $H_{3'}$); 8.01 (d, *J* = 8.8, 1H, H_5); 8.36 (s, 1H, H_2). ^{13}C-NMR (methanol-d_4, 75 MHz): δ 103.6 (C_8); 116.8 (C_6); 118.0 ($C_{6'}$); 118.3 (C_{4a}); 124.8 ($C_{2'}$); 128.2 (C_5); 128.9 ($C_{5'}$); 129.0 ($C_{4'}$); 131.1 ($C_{3'}$); 140.8 (C_3); 150.8 (C_2); 153.6 ($C_{1'}$); 159.8 (C_{8a}); 165.1 (C_7); 173.6 (C_4). MS (ESI, *m/z*): 321.0, 323.0, 324.9 $[M+H]^+$. HPLC (method B, t_R, min): 28.80.

Ethyl {[3-(2,4-dichlorophenoxy)-4-oxo-4*H*-chromen-7-yl]oxy}acetate (51). Following the general procedure 4, derivative **51** was obtained from hydroxychromone **50** (35 mg, 0.11 mmol) and ethyl 2-bromoacetate (24 μL, 0.22 mmol, 2 equiv) in 91% yield.

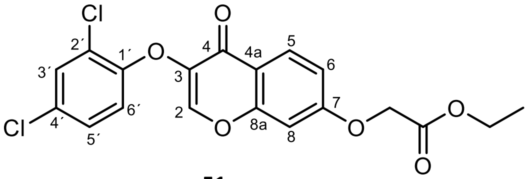

51

R_f: 0.50 (hexane/ethyl acetate 8:2). IR (ATR): ν 1752, 1653 (C=O); 1196 (C-O-C). ^{1}H-NMR ($CDCl_3$, 300 MHz): δ 1.32 (t, *J* = 7.1, 3H, CH_3); 4.30 (q, *J* = 7.1, 2H, C$\underline{H_2}$$CH_3$); 4.73 (s, 2H, CH_2); 6.82 (d, *J* = 8.8, 1H, $H_{6'}$); 6.88 (d, *J* = 2.4, 1H, H_8); 7.05 (dd, *J* = 9.0, 2.4, 1H, H_6); 7.11 (dd, *J* = 8.8, 2.5, 1H, $H_{5'}$); 7.44 (d, *J* = 2.5, 1H, $H_{3'}$); 8.03 (s, 1H, H_2); 8.17 (d, *J* = 9.0, 1H, H_5). ^{13}C-NMR ($CDCl_3$, 75 MHz): δ 14.3 (CH_3); 62.0 ($\underline{C}H_2CH_3$); 65.6 (CH_2), 101.7 (C_8); 115.0 (C_6); 117.4 ($C_{6'}$); 119.4 (C_{4a}); 124.4 ($C_{2'}$); 127.8 ($C_{5'}$); 128.0 (C_5); 128.6 ($C_{4'}$); 130.5 ($C_{3'}$); 140.6 (C_3); 147.9 (C_2); 151.8 ($C_{1'}$); 157.6 (C_{8a}); 162.5 (C_7); 167.8 (C=O); 171.6 (C_4). MS (ESI, *m/z*): 408.9, 410.9, 412.9 $[M+H]^+$. HPLC (method B, t_R, min): 17.52.

Ethyl {4-[4-(2,4-dichlorophenoxy)-3-methyl-1,2-oxazol-3-yl]-3-hydroxyphenoxy} acetate (52). To a solution of chromone **29** (210 mg, 0.51 mmol, 1 equiv) in absolute EtOH (5.1 mL), hydroxylamine hydrochloride (570 mg, 8.16 mmol, 16 equiv) and pyridine (1.0 mL, 12.75 mmol, 25 equiv) were added and the mixture was refluxed 12 h. After this time, the reaction was quenched with water, neutralized with a solution of 1 M HCl and the organic solvent was removed under vacuum. The resulting aqueous residue was extracted with ethyl acetate (3x), and the combined organic phases were washed with brine, dried over Na_2SO_4, filtered and concentrated under reduced pressure. Next, the resulting crude was dissolved in absolute ethanol (4.3 mL), *p*-toluenesulfonic acid monohydrate (82 mg, 1.02 mmol, 2 equiv) was added and the reaction was refluxed for 5 h. After this time, the solvent was evaporated under vacuum and the residue was neutralized with 1 M $NaHCO_3$. The mixture was extracted with ethyl acetate (3x) and the combined organic phases were washed with brine, dried over Na_2SO_4, filtered, and concentrated under reduced pressure. The crude was purified by flash chromatography (hexane/ethyl acetate, 8:2 to hexane/ethyl acetate, 1:1) to yield pure compound **52** in a 40% yield.

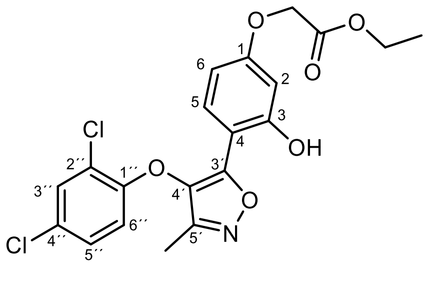

52

mp: 186-188 ºC. R_f: 0.51 (hexane/ethyl acetate, 6:4). IR (ATR): ν 3317 (OH); 1735 (C=O); 1297 (C-O-C). ^{1}H-NMR ($CDCl_3$, 300 MHz): δ 1.29 (t, J = 7.1, 3H, $CH_2C\underline{H_3}$); 2.16 (s, 3H, $C_{5'}CH_3$); 4.28 (q, J = 7.1, 2H, $C\underline{H_2}CH_3$); 4.61 (s, 2H, OCH_2); 6.46-6.58 (m, 2H, H_6, H_2); 6.68 (d, J = 8.8, 1H, $H_{6''}$); 7.11 (dd, J = 8.8, 2.5, 1H, $H_{5''}$); 7.48 (d, J = 2.4, 1H, $H_{3''}$); 7.56 (dd, J = 8.6, 2.5, 1H, H_5). ^{13}C-NMR ($CDCl_3$, 75 MHz): δ 9.2 ($C_{5'}CH_3$); 14.3 ($CH_2\underline{C}H_3$); 61.7 ($\underline{C}H_2CH_3$); 65.3 (OCH_2); 103.5 (C_2); 108.6 (C_6); 108.9 (C_4); 115.8 ($C_{6''}$); 123.8 ($C_{2''}$); 128.2 ($C_{5''}$); 128.3 (C_5); 129.1 ($C_{4''}$); 130.0 ($C_{4'}$); 130.9

($C_{3''}$); 151.1 ($C_{1''}$); 155.7 (C_3/$C_{3'}$); 156.1 ($C_{5'}$); 157.6 (C_3/$C_{3'}$); 161.1 (C_1); 168.4 (C=O). MS (ESI, *m/z*): 438.1, 440.1, 442.1 $[M+H]^+$. HPLC (method B, t_R, min): 17.98

2.3.7. Synthesis of intermediates 56-59 (Scheme 6)

2-(2,4-Difluorophenoxy)-1-(2,4-dihydroxyphenyl)ethanone (56). Following the general procedure 1, compound **56** was obtained from 2,4-difluorophenoxyacetic acid (1.1 g, 5.32 mmol) in 10% yield. Chromatography: hexane/DCM 9:1 to DCM.

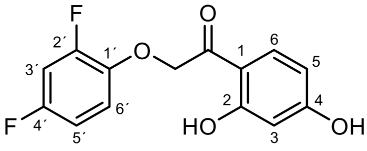

56

mp: 151-153 ºC. R_f: 0.59 (DCM/ethyl acetate, 9:1). IR (ATR): ν 3348 (OH); 1631 (C=O); 1248, 1208 (C-O-C). ^{1}H-NMR (methanol-d_4, 700 MHz): δ 5.38 (s, 2H, CH_2); 6.30 (d, *J* = 2.5, 1H, H_3); 6.40 (dd, *J* = 9.0, 2.5, 1H, H_5); 6.82-6.84 (m, 1H, $H_{5'}$); 6.99 (ddd, *J* = 11.5, 8.5, 3.0, 1H, $H_{3'}$); 7.04 (td, *J* = 9.5, 5.0, 1H, $H_{6'}$); 7.75 (d, *J* = 9.0, 1H, H_6). ^{13}C-NMR (methanol-d_4,175 MHz): δ 72.4 (CH_2); 103.8 (C_3); 105.6 (dd, *J* = 27.0, 22.2, $C_{3'}$); 109.6 (C_5); 111.4 (dd, *J* = 23.0, 4.0, $C_{5'}$); 112.4 (C_1); 117.7 (dd, *J* = 10.0, 2.0, $C_{6'}$); 132.8 (C_6); 144.4 (dd, *J* = 10.5, 3.5, $C_{1'}$); 153.8 (dd, *J* = 248.0, 12.0, $C_{2'}$); 158.3 (dd, *J* = 240.5, 10.5, $C_{4'}$); 166.2 (C_4); 166.9 (C_2); 198.4 (C=O). ^{19}F-NMR (methanol-d_4, 282 MHz): -128.7, -118.5. MS (ESI, *m/z*): 281.0 $[M+H]^+$. HPLC (method B, t_R, min): 15.23.

3-(2,4-Difluorophenoxy)-2-methyl-4-oxo-4*H*-benzopyran-7-yl acetate (57). Following the general procedure 2, chromone **57** was obtained from 2,4-dihydroxyphenylethanone **56** (109 mg, 0.39 mmol) in 71% yield. Chromatography: hexane/ethyl acetate, 9:1 to hexane/ethyl acetate, 7:3.

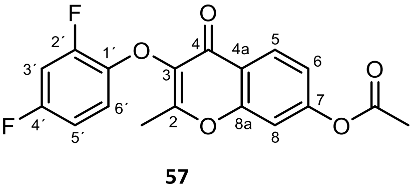

57

mp: 111-113 ºC. R_f: 0.28 (hexane/ethyl acetate, 7:3). IR (ATR): ν 1766, 1655 (C=O); 1196 (C-O-C). ^{1}H-NMR ($CDCl_3$, 700 MHz): δ 2.36 (s, 3H, C_2CH_3); 2.50 (s, 3H, $COCH_3$); 6.70-6.73 (m, 1H, $H_{5'}$); 6.85 (td, J = 9.0, 5.5, 1H, $H_{6'}$); 6.91 (ddd, J = 11.0, 8.5, 3.0, 1H, $H_{3'}$); 7.14 (dd, J = 8.5, 2.0, 1H, H_6); 7.31 (d, J = 2.0, 1H, H_8); 8.19 (d, J = 8.5, 1H, H_5). ^{13}C-NMR ($CDCl_3$,175 MHz): δ 15.8 ($CO\underline{C}H_3$); 21.3 ($C_2\underline{C}H_3$); 105.3 (dd, J = 27.0, 22.0, $C_{3'}$); 110.7 (dd, J = 23.0, 4.0, $C_{5'}$); 111.1 (C_8); 117.2 (d, J = 9.6, 1.6, $C_{6'}$); 119.5 (C_6); 122.1 (C_{4a}); 127.5 (C_5); 137.4 (C_3); 141.5 (dd, J = 10.5, 4.0, $C_{1'}$); 152.2 (dd, J = 250.0, 12.0, $C_{2'}$); 154.6 (C_7); 156.0 (C_{8a}); 157.7 (dd, J = 243.5, 10.0, $C_{4'}$); 160.7 (C_2); 168.7 (C=O); 171.6 (C_4). ^{19}F-NMR ($CDCl_3$, 282 MHz): -129.1, -117.7. MS (ESI, *m/z*): 347.0 $[M+H]^+$. HPLC (method B, t_R, min): 17.74.

3-(2,4-Difluorophenoxy)-7-hydroxy-2-methyl-4*H*-chromen-4-one (58). Following the general procedure 3, compound **58** was obtained from acetoxychromone **57** (75 mg, 0.22 mmol) in 99% yield.

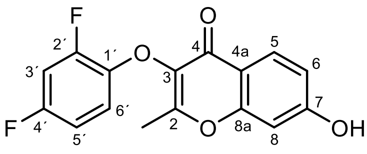

58

mp: 167-169 ºC. R_f: 0.15 (hexane/ethyl acetate, 7:3). IR (ATR): ν 3318 (OH); 1638 (C=O); 1263 (C-O-C). ^{1}H-NMR (methanol-d_4, 700 MHz): δ 2.45 (s, 3H, CH_3); 6.78-6.81 (m, 1H, $H_{5'}$); 6.87-6.90 (m, 1H, $H_{6'}$); 6.89 (dd, J = 2.3, 1H, H_8); 6.93 (dd, J = 9.0, 2.0, 1H, H_6); 7.07 (ddd, J = 11.5, 8.5, 3.0, 1H, $H_{3'}$); 7.95 (d, J = 9.0, 1H, H_5). ^{13}C-NMR (methanol-d_4,175 MHz): δ 15.6 (CH_3); 103.4 (C_8); 105.9 (dd, J = 27.5, 22.0, $C_{3'}$); 111.6 (dd, J = 23.0, 4.0, $C_{5'}$); 116.4 (C_6); 117.5 (d, J = 9.5, 1.6, $C_{6'}$); 117.8 (C_{4a}); 128.0 (C_5); 137.2 (C_3); 143.1 (dd, J = 11.0, 4.0, $C_{1'}$); 153.2 (dd, J = 249.5, 13.0, $C_{2'}$); 158.8 (dd, J = 241.7, 10.5, $C_{4'}$); 159.1 (C_{8a}); 162.4 (C_2); 164.9 (C_7); 173.8 (C_4). MS (ESI, *m/z*): 303.1 $[M-H]^-$. HPLC (method B, t_R, min): 14.92.

Methyl {[3-(2,4-difluorophenoxy)-2-methyl-4-oxo-4*H*-benzopyran-7-yl]oxy} acetate (59). Following the general procedure 4, compound **59** was obtained from hydroxychromone **58** (60 mg, 0.20 mmol) and methyl 2-bromoacetate (0.04 mL, 0.39 mmol, 3.2 equiv) in 99% yield.

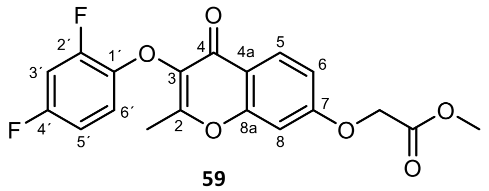

59

mp: 270-272 ºC. R_f: 0.19 (hexane/ethyl acetate, 7:3). IR (ATR): ν 1758 (C=O); 1648 (C=O); 1214 (C-O-C). ^{1}H-NMR ($CDCl_3$, 700 MHz): δ 2.47 (s, 3H, C_2CH_3); 3.84 (s, 3H, OCH_3); 4.75 (s, 2H, CH_2); 6.71 (dddd, *J* = 9.0, 8.0, 3.0, 1.5, 1H, $H_{5'}$); 6.82-6.86 (m, 1H, $H_{6'}$); 6.84 (d, *J* = 2.3, 1H, H_8); 6.90 (ddd, *J* = 11.0, 8.5, 3.0, 1H, $H_{3'}$); 7.01 (dd, *J* = 9.0, 2.5, 1H, H_6); 8.11 (d, *J* = 9.0, 1H, H_5). ^{13}C-NMR ($CDCl_3$,175 MHz): δ 15.7 ($C_2\underline{C}H_3$); 52.7 (OCH_3); 65.4 (CH_2); 101.4 (C_8); 105.3 (dd, *J* = 27.1, 22.0, $C_{3'}$); 110.7 (dd, *J* = 23.0, 3.5, $C_{5'}$); 114.5 (C_6); 117.2 (d, *J* = 9.0, 1.8, $C_{6'}$); 119.0 (C_{4a}); 127.9 (C_5); 137.3 (C_3); 141.6 (dd, *J* = 11.0, 3.5, $C_{1'}$); 152.2 (dd, *J* = 250.0, 12.5, $C_{2'}$); 157.0 (C_{8a}); 157.7 (dd, *J* = 243.5, 10.5, $C_{4'}$); 160.0 (C_2); 162.1 (C_7); 168.4 (C=O); 171.7 (C_4). ^{19}F-NMR ($CDCl_3$, 282 MHz): -129.2, -117.9. MS (ESI, *m/z*): 377.1 $[M+H]^+$. HPLC (method B, t_R, min): 16.79.

2.3.8. Synthesis of intermediates 60-63 (Scheme 6)

2-{[3-(2,4-Dichlorophenoxy)-2-methyl-4-oxo-4*H*-chromen-7-yl]oxy}acetonitrile (60). Following the general procedure 4, compound **60** was obtained from hydroxychromone **25** (327 mg, 0.97 mmol) and bromoacetonitrile (0.16 mL, 2.33 mmol, 2.4 equiv) in 77% yield. Chromatography: hexane/ethyl acetate, 9:1 to hexane/ethyl acetate, 1:1.

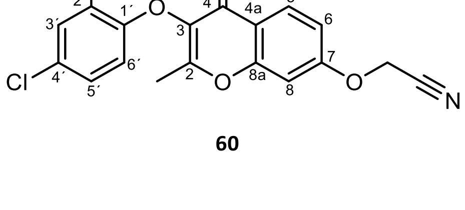

60

mp: 134-136 ºC. R_f: 0.33 (hexane/ethyl acetate, 7:3). IR (ATR): ν 2250 (CN), 1637 (C=O), 1240 (C-O-C). ^{1}H-NMR ($CDCl_3$, 300 MHz): δ 2.47 (s, 3H, CH_3); 4.90 (s, 2H, OCH_2); 6.66 (d, *J* = 8.8, 1H, $H_{6'}$); 7.02 (d, *J* = 2.4, 1H, H_8); 7.02-7.14 (m, 2H, H_6, $H_{5'}$); 7.44 (d, *J* = 2.5, 1H, $H_{3'}$); 8.18 (d, *J* = 8.8, 1H, H_5). ^{13}C-NMR ($CDCl_3$, 75 MHz): δ 15.8 (CH_3); 53.6 (OCH_2); 101.9 (C_8); 114.2 (CN); 114.5 (C_6); 115.6 ($C_{6'}$); 119.8 (C_{4a}); 123.6 ($C_{2'}$); 127.7 ($C_{5'}$); 127.8 ($C_{4'}$); 128.4 (C_5); 130.5 ($C_{3'}$); 137.0 (C_3); 151.7 ($C_{1'}$); 156.9 (C_{8a}); 160.5, 160.6 (C_2, C_7); 171.2 (C_4). MS (ESI, *m/z*): 375.9, 377.9, 379.9 $[M+H]^+$. HPLC (method B, t_R, min): 17.33.

{[3-(2,4-Difluorophenoxy)-2-methyl-4-oxo-4H-benzopyran-7yl]oxy}acetonitrile (61). Following the general procedure 4, compound **61** was obtained from hydroxychromone **58** (327 mg, 0.97 mmol) and bromoacetonitrile (0.16 mL, 2.33 mmol, 2.4 equiv) in 93% yield. Chromatography: hexane to hexane/ethyl acetate, 8:2.

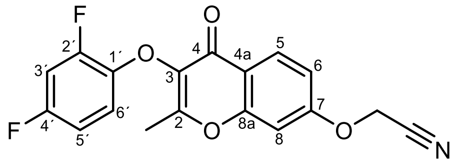

61

mp: 120-122 ºC. R_f: 0.62 (hexane/ethyl acetate, 6:4). IR (ATR): ν 2250 (CN); 1642 (C=O); 1210, 1160 (C-O-C). ^{1}H-NMR ($CDCl_3$, 300 MHz): δ 2.50 (s, 3H, CH_3); 4.89 (s, 2H, OCH_2); 6.72 (dddd, *J* = 9.2, 7.7, 2.9, 1.7, 1H, $H_{5'}$); 6.82-6.94 (m, 2H, $H_{6'}$, $H_{3'}$); 7.00 (d, *J* = 2.5, 1H, H_8); 7.04 (dd, *J* = 8.8, 2.5, 1H, H_6); 8.16 (d, *J* = 8.8, 1H, H_5). ^{13}C-NMR ($CDCl_3$, 75 MHz): δ 15.6 (CH_3); 53.5 (OCH_2); 101.8 (C_8); 105.2 (dd, *J* = 26.9, 21.9, $C_{3'}$); 110.6 (dd, *J* = 22.9, 4.0, $C_{5'}$); 114.1 (CN); 114.3 (C_6); 117.2 (dd, *J* = 9.6, 2.2, $C_{6'}$); 119.8 (C_{4a}); 128.2 (C_5); 137.3 (C_3); 141.4 (dd, *J* = 10.8, 3.8, $C_{1'}$); 152.1 (dd, *J* = 250.0, 12.1, $C_{2'}$); 156.7 (C_{8a}); 157.6 (dd, *J* = 243.6, 10.3, $C_{4'}$); 160.2 (C_2); 160.3 (C_7); 171.4 (C_4). ^{19}F-NMR (methanol-*d*$_4$, 282 MHz): -129.1, -117.7. MS (ESI, *m/z*): 344.0 $[M+H]^+$. HPLC (method B, t_R, min): 16.04.

{4-[4-(2,4-Dichlorophenoxy)-5-methyl-1*H*-pyrazol-3-yl]-3-hydroxyphenoxy}acetonitrile (62). Following the general procedure 5, compound **62** was obtained from chromone **60** (272 mg, 0.72 mmol) in 93% yield.

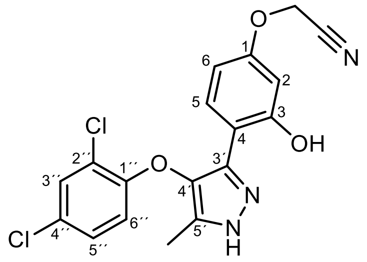

62

mp: >190 ºC (decomp.). R_f: 0.13 (hexane/ethyl acetate, 6:4). IR (ATR): ν 3324 (NH); 1200 (C-O-C). ^{1}H-NMR ($CDCl_3$, 300 MHz): δ 2.21 (s, 3H, CH_3); 4.73 (s, 2H, CH_2); 6.44 (dd, *J* = 8.8, 2.7, 1H, H_6); 6.60 (d, *J* = 2.7, 1H, H_2); 6.61 (d, *J* = 8.8, 1H, $H_{6''}$); 7.05 (dd, *J* = 8.9, 2.5, 1H, $H_{5''}$); 7.47 (d, *J* = 2.5, 1H, $H_{3''}$); 7.65 (d, *J* = 8.7, 1H, H_5). MS (ESI, *m/z*): 389.9, 391.9, 393.9 $[M+H]^+$. HPLC (method B, t_R, min): 17.58.

{4-[4-(2,4-Difluorophenoxy)-5-methyl-1*H*-pyrazol-3-yl]-3-hydroxyphenoxy}acetonitrile (63). Following the general procedure 5, compound **63** was obtained from chromone **61** (53 mg, 0.14 mmol) in 21% yield. Chromatography: hexane to hexane/ethyl acetate, 7:3.

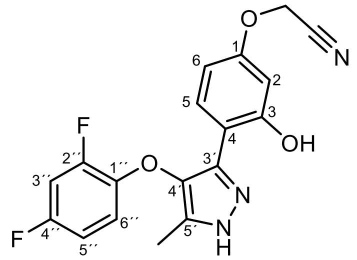

63

mp: 170-173 ºC. R_f: 0.55 (hexane/ethyl acetate, 6:4). IR (ATR): ν 3351, 3326 (NH, OH); 2250 (CN); 1247 (C-O-C). ^{1}H-NMR (methanol-d_4, 300 MHz): δ 2.14 (s, 3H, CH_3); 4.88 (s, 2H, OCH_2); 6.45 (dd, *J* = 8.8, 2.7, 1H, H_6); 6.56 (d, *J* = 2.7, 1H, H_2); 6.70-6.74 (m, 2H, $H_{5''}$, $H_{6''}$); 7.01-7.04 (m, 1H, $H_{3''}$); 7.63 (d, *J* = 8.7, 1H, H_5). ^{13}C-NMR (methanol-d_4, 75 MHz): δ 8.7 (CH_3); 54.1 (OCH_2); 103.7 (C_2);

105.6 (dd, J = 27.3, 22.0, $C_{3''}$); 106.8 (C_6); 111.6 (dd, J = 23.1, 4.1, $C_{5''}$); 112.2 (C_4); 116.6 (CN); 117.1 (dd, J = 9.6, 1.6, $C_{6''}$); 128.7 (C_5); 133.6, 136.6 ($C_{4'}$, $C_{5'}$); 140.4 ($C_{3'}$); 143.5 (dd, J = 10.7, 3.5, $C_{1''}$); 152.9 (dd, J = 249.1, 12.3, $C_{2''}$); 158.4 (dd, J = 242.3, 10.1, $C_{4''}$); 158.1 (C_3); 158.5 (C_1). MS (ESI, *m/z*): 357.8 $[M+H]^+$. HPLC (method B, t_R, min): 17.85.

3. NMR Spectra and HPLC Trace Analysis of Final Compounds

^{1}H NMR spectrum for **2** (DMSO-*d6*, 700 MHz):

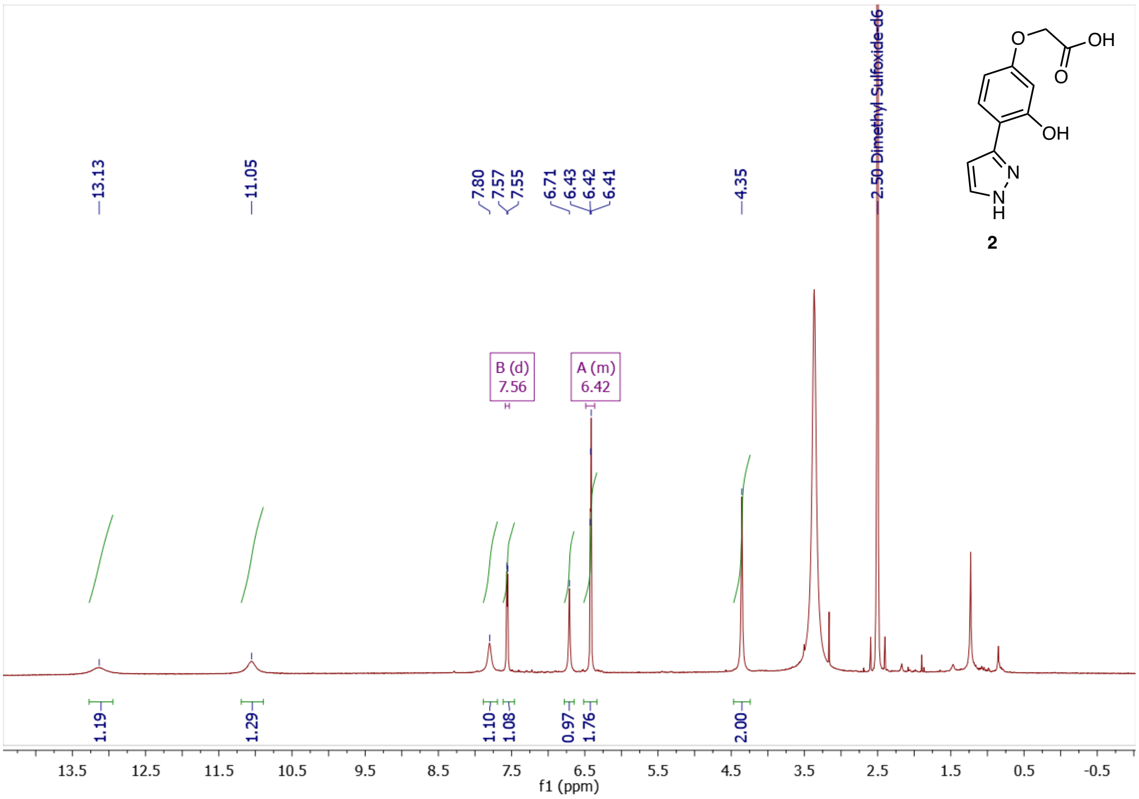

^{13}C NMR spectrum for **2** (DMSO-*d6*, 175 MHz):

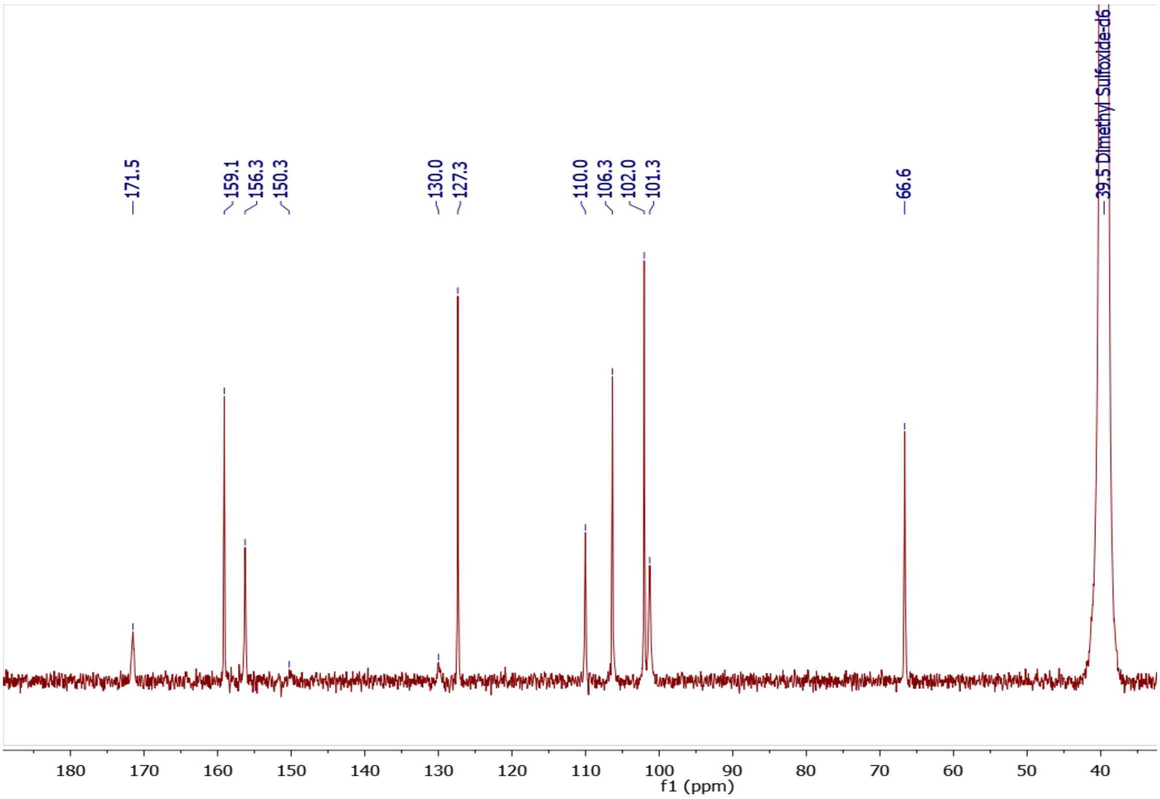

HPLC traces of compound **2**:

(ESI negative mode)

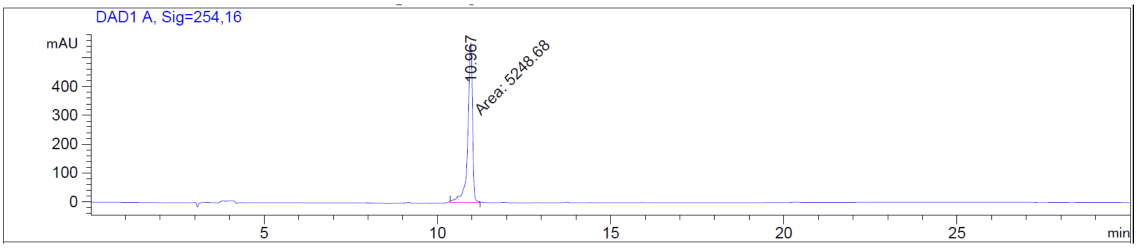

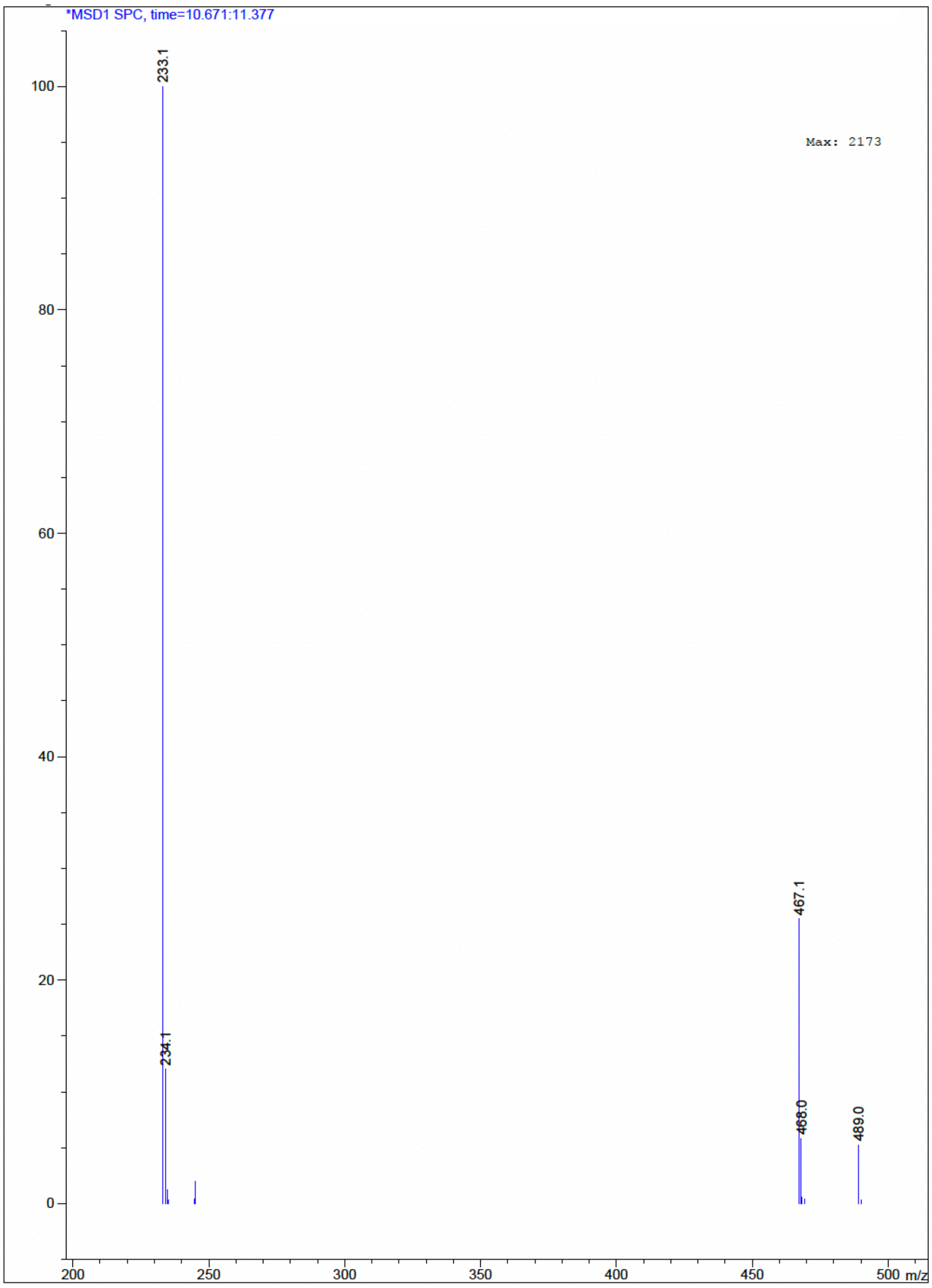

^{1}H NMR spectrum for **3** (CD_3OD, 300 MHz):

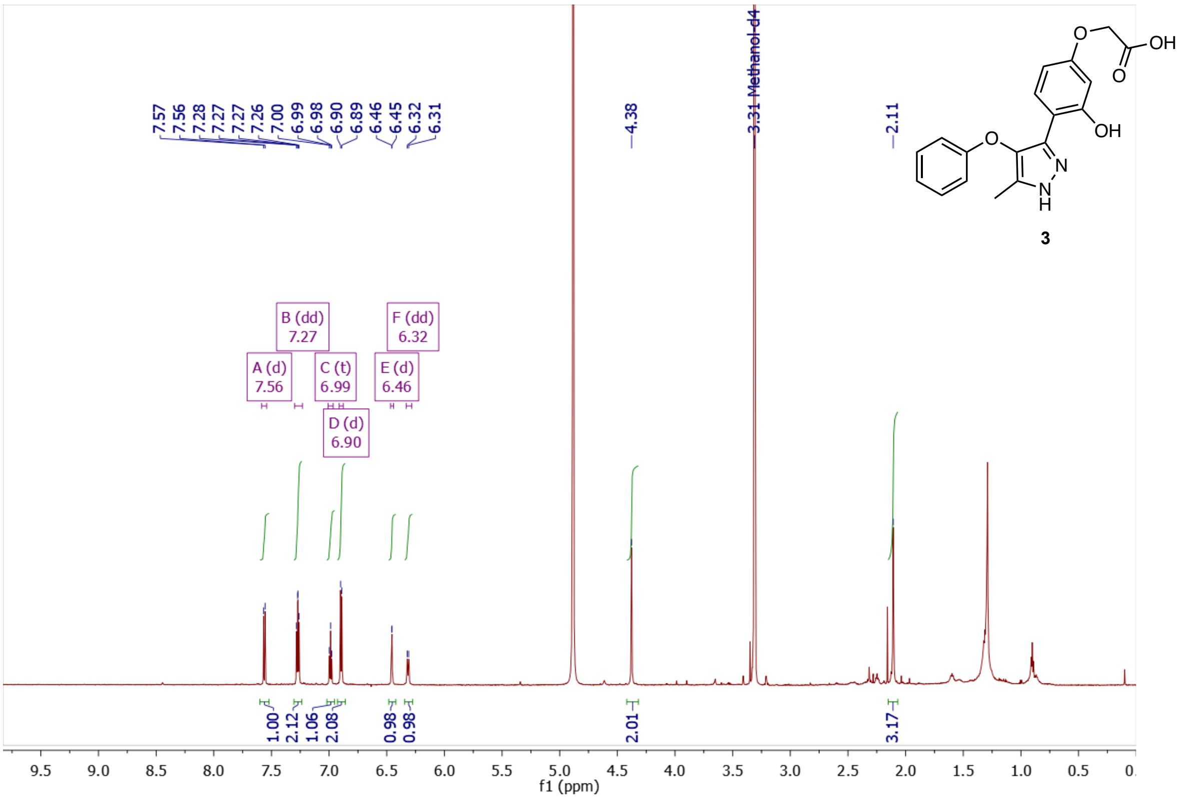

HPLC traces of compound **3**:

(ESI negative mode)

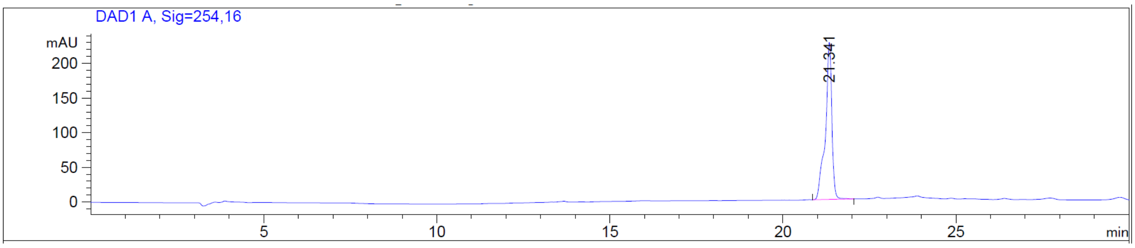

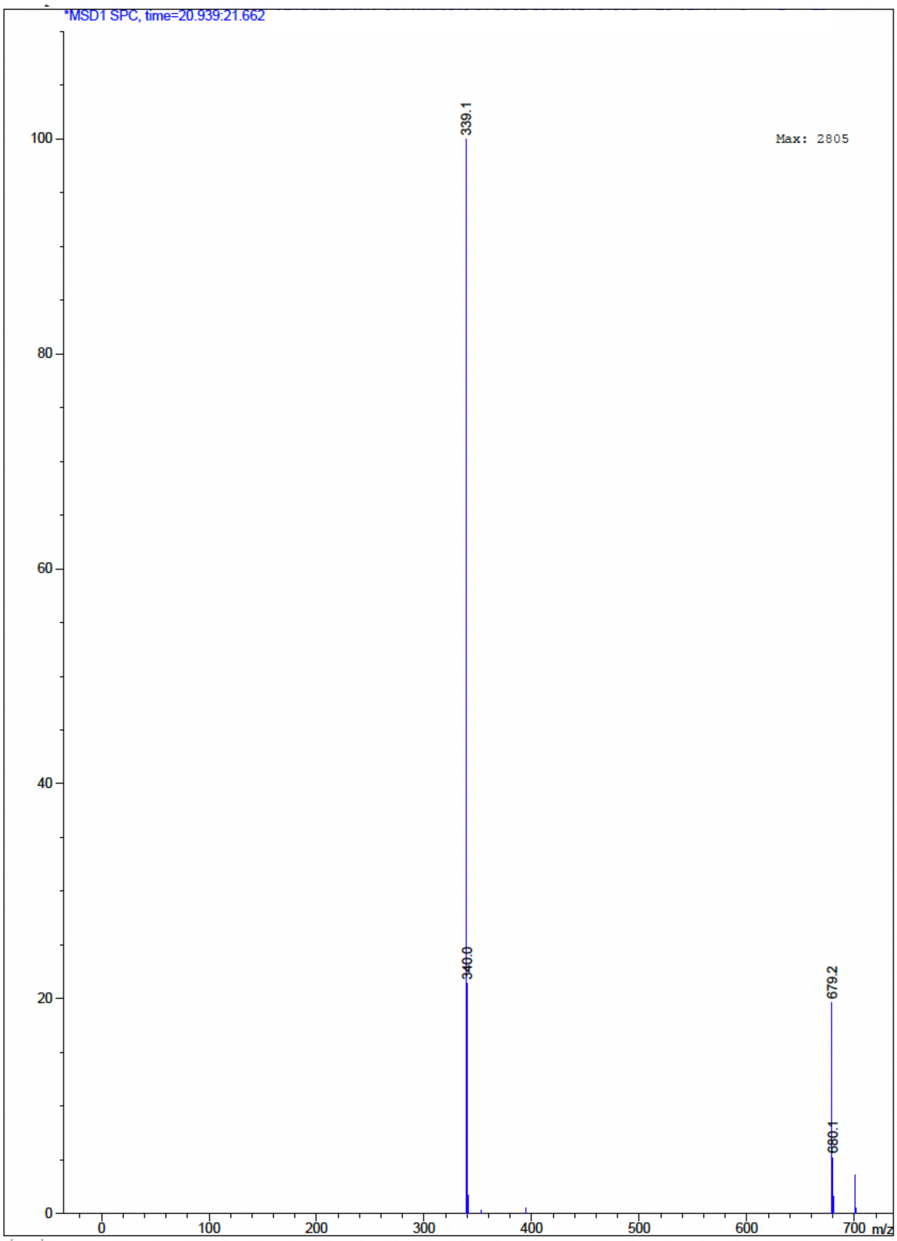

^{1}H NMR spectrum for **10** (CD_3OD, 700 MHz):

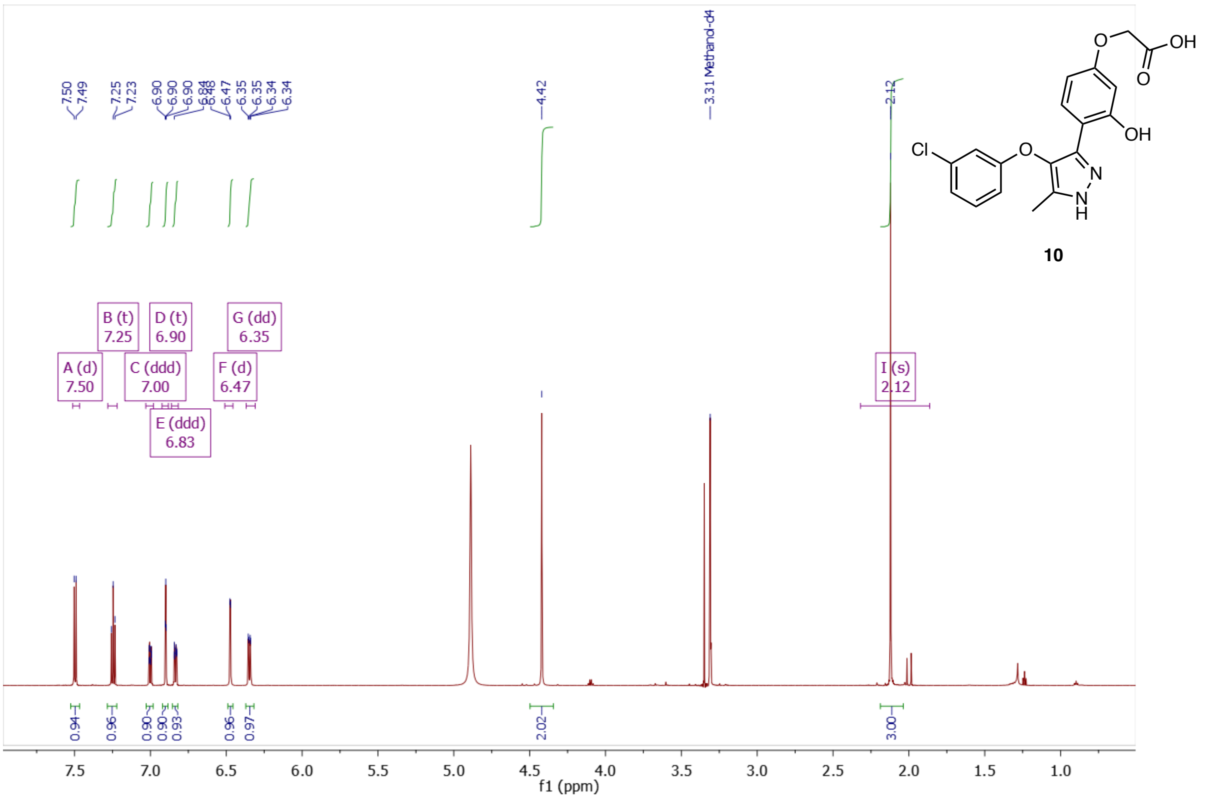

^{13}C NMR spectrum for **10** (CD_3OD, 175 MHz):

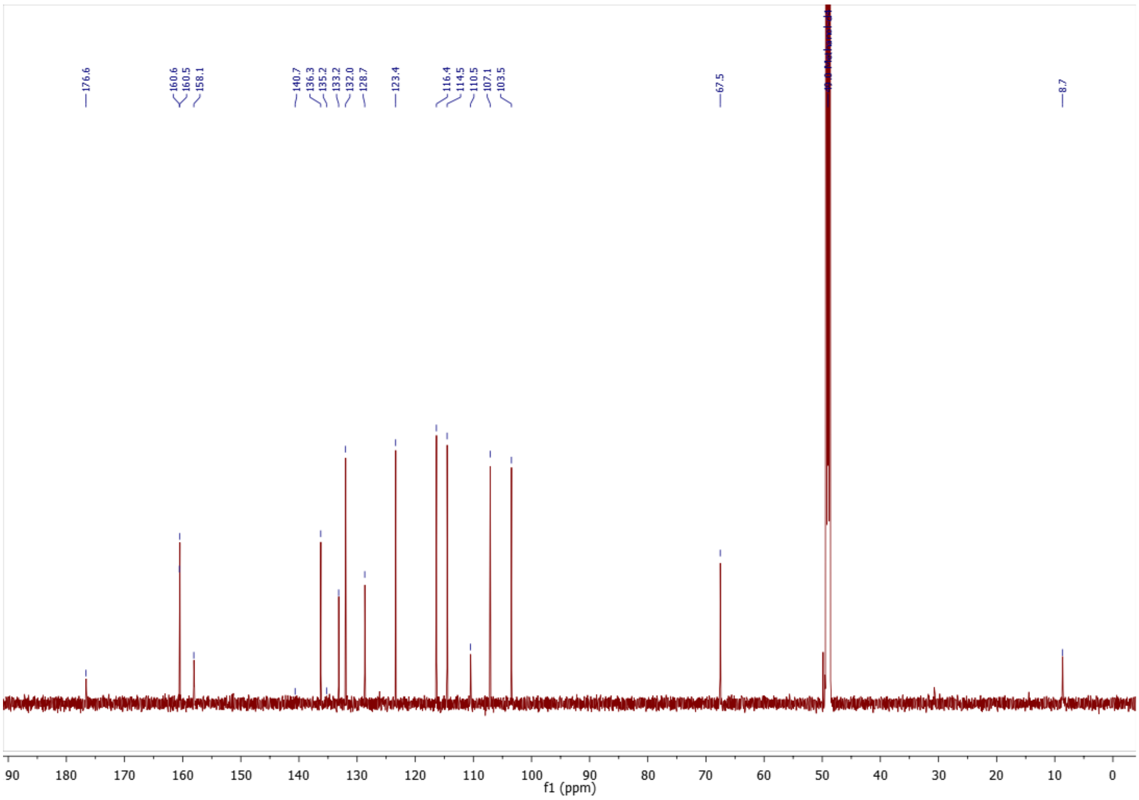

HPLC traces of compound **10**:

(ESI positive mode)

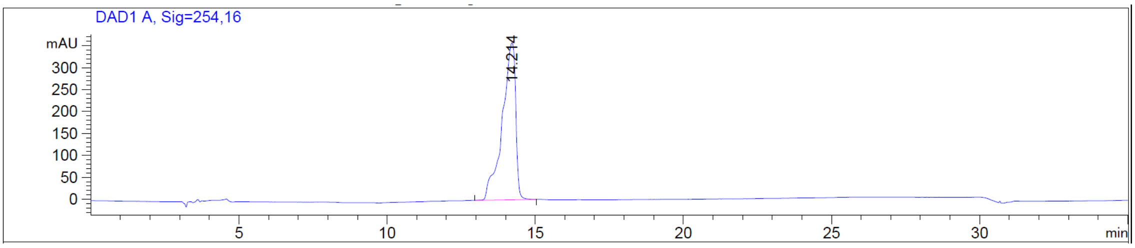

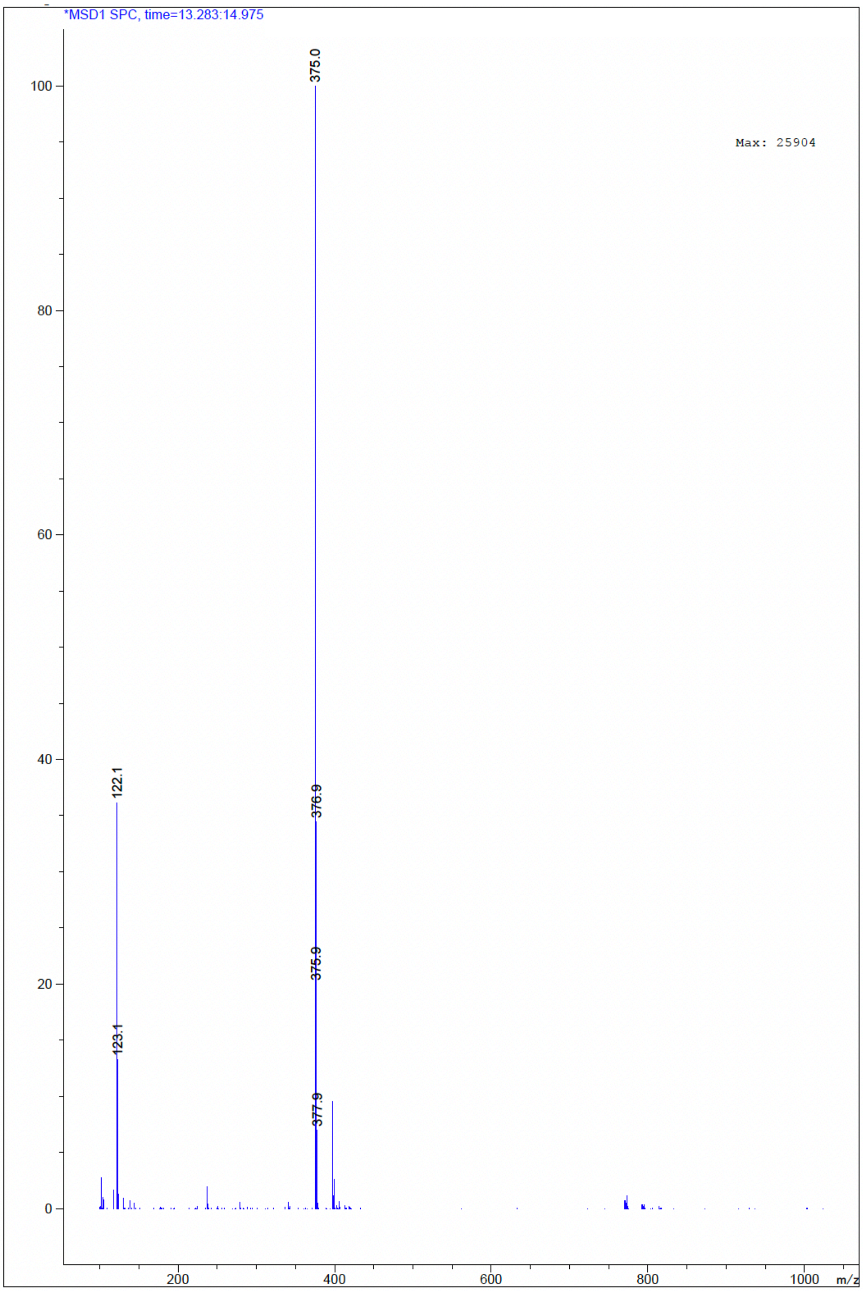

^{1}H NMR spectrum for **11** (CD_3OD, 700 MHz):

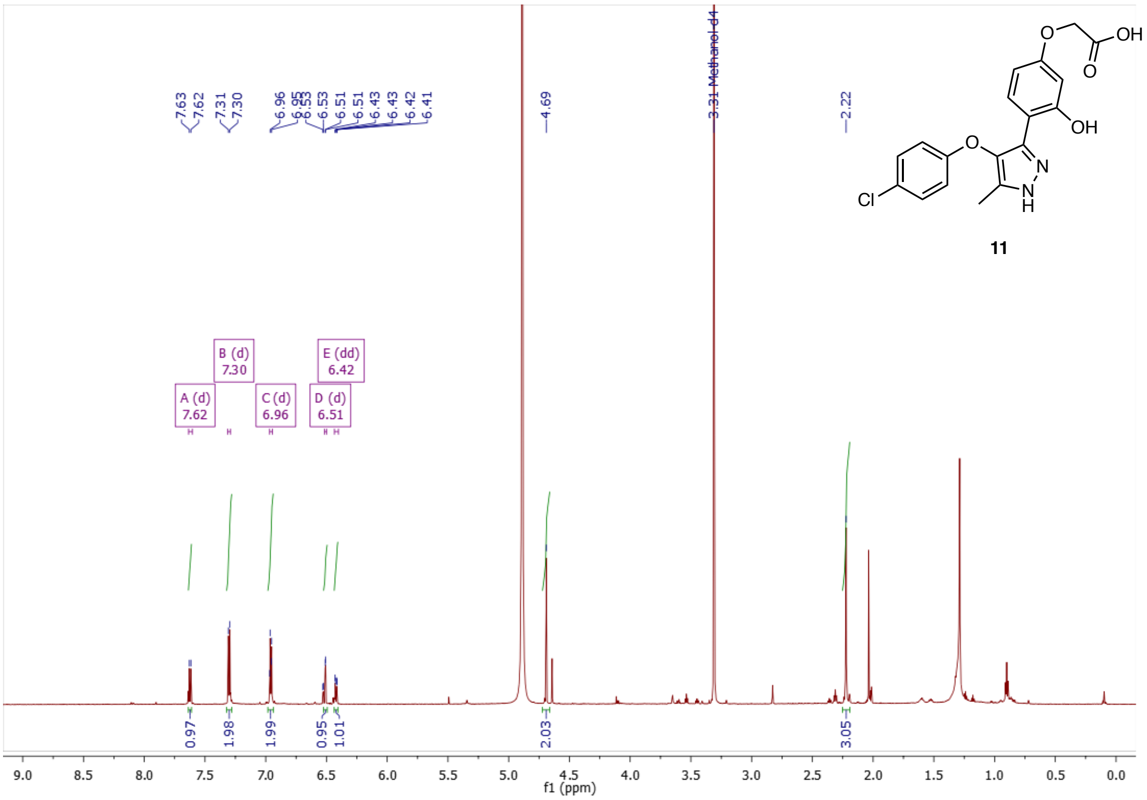

HPLC traces of compound **11**:

(ESI negative mode)

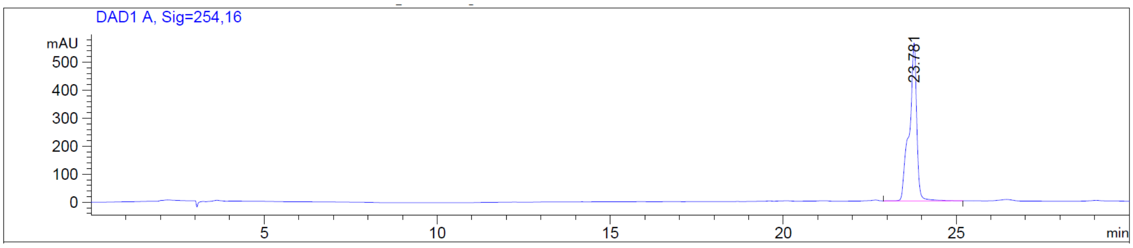

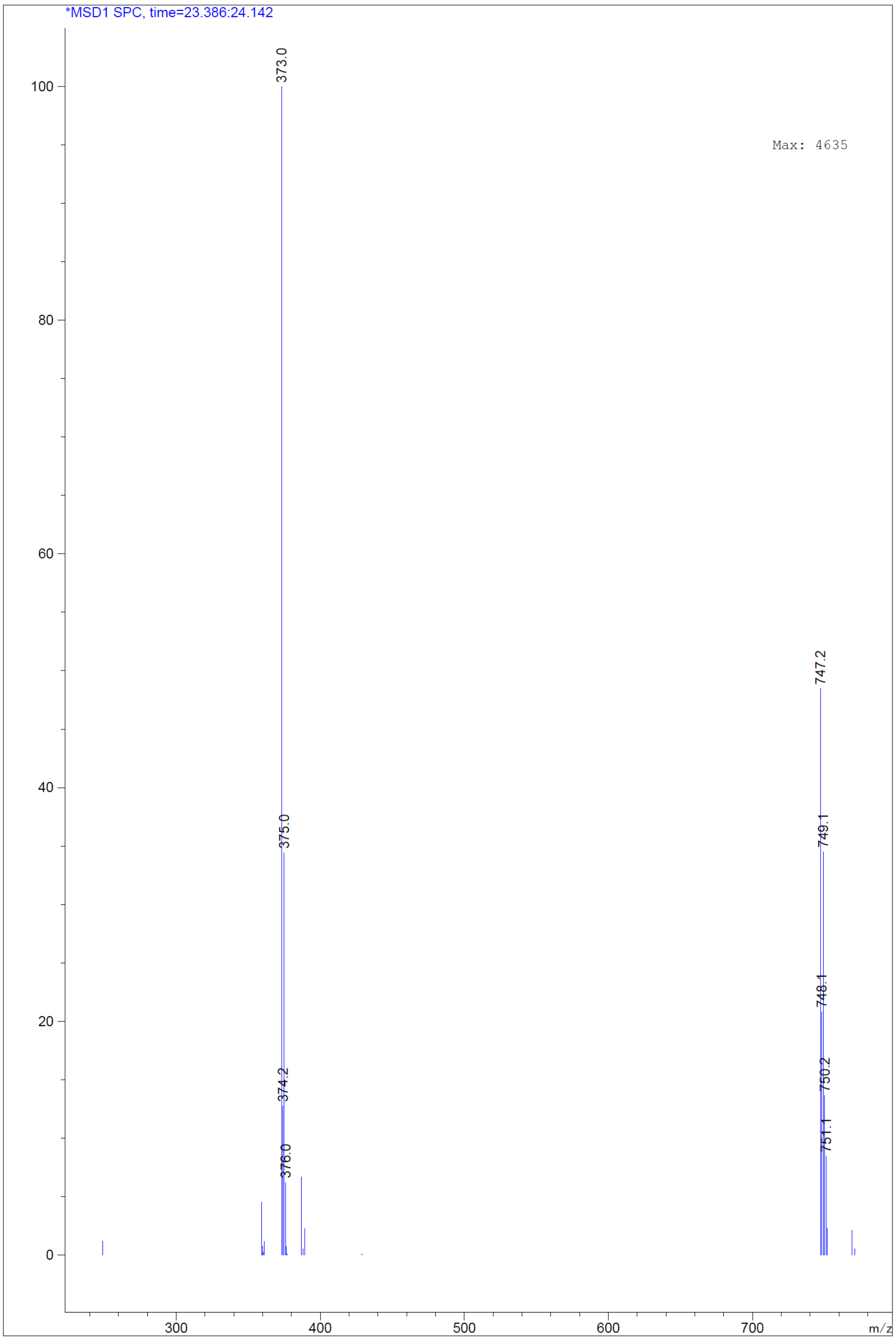

^{1}H NMR spectrum for **12** (CD_3OD, 700 MHz):

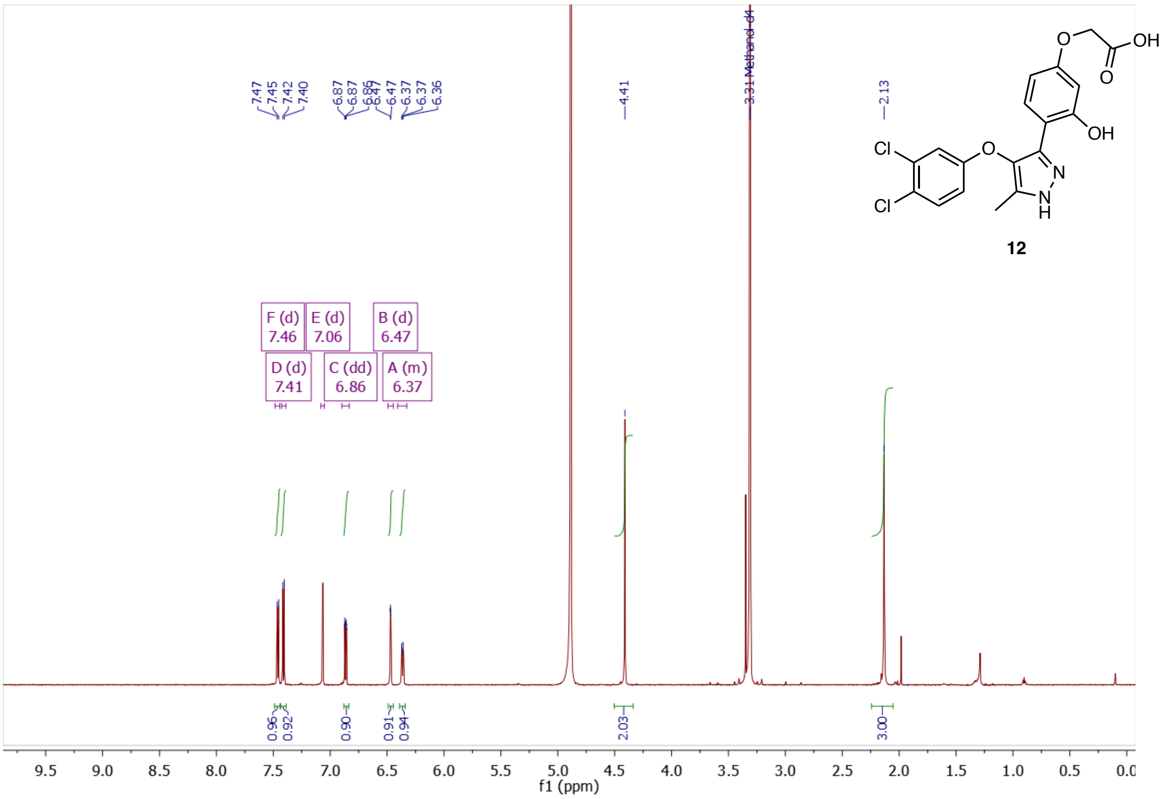

^{13}C NMR spectrum for **12** (CD_3OD, 175 MHz):

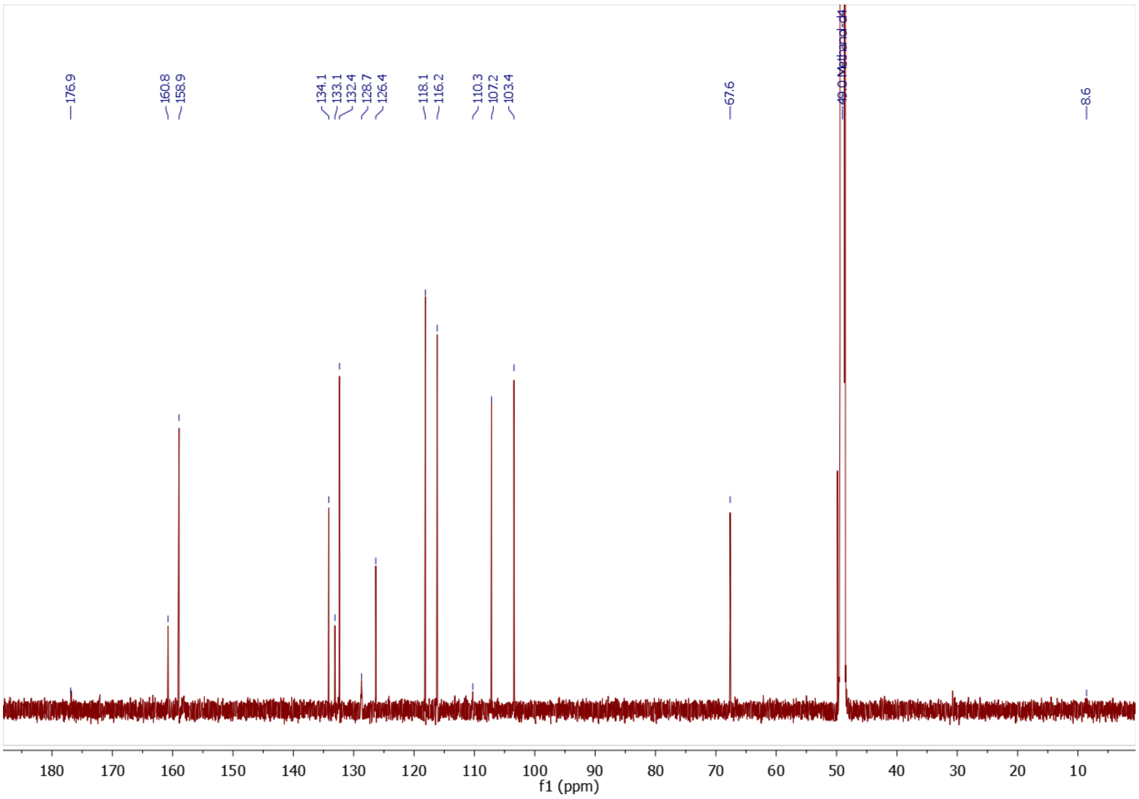

HPLC traces of compound **12**:

(ESI positive mode)

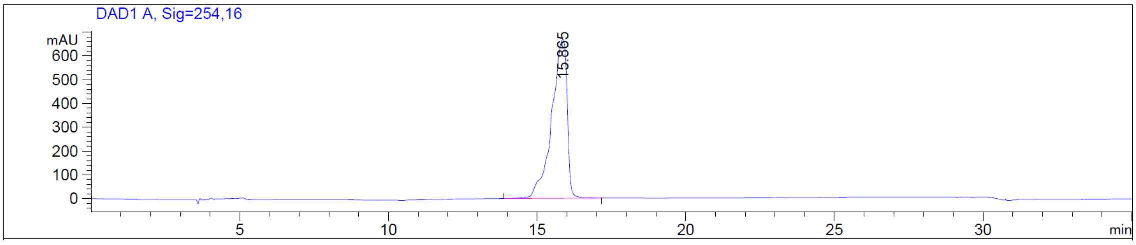

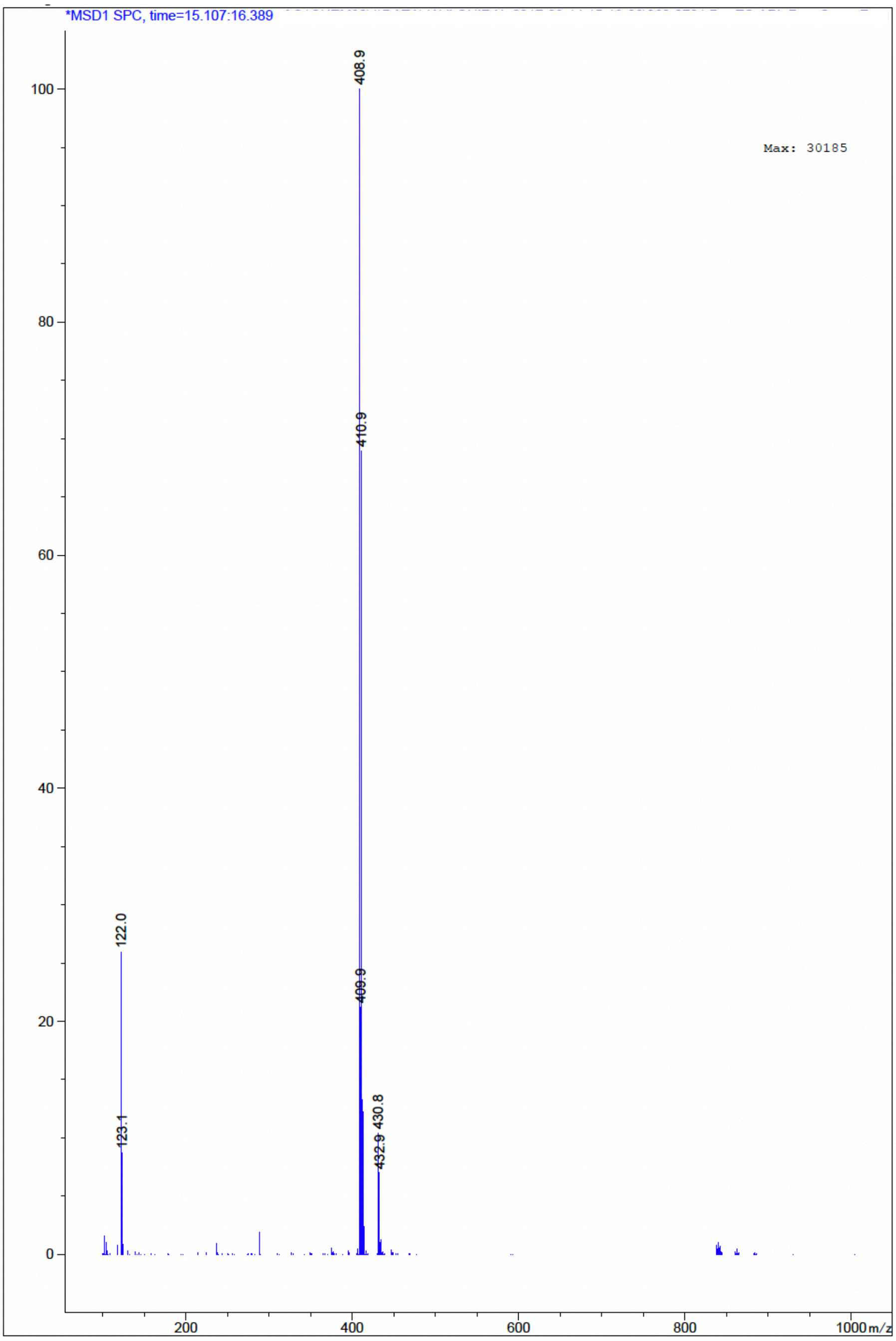

^{1}H NMR spectrum for **13** (CD_3OD, 700 MHz):

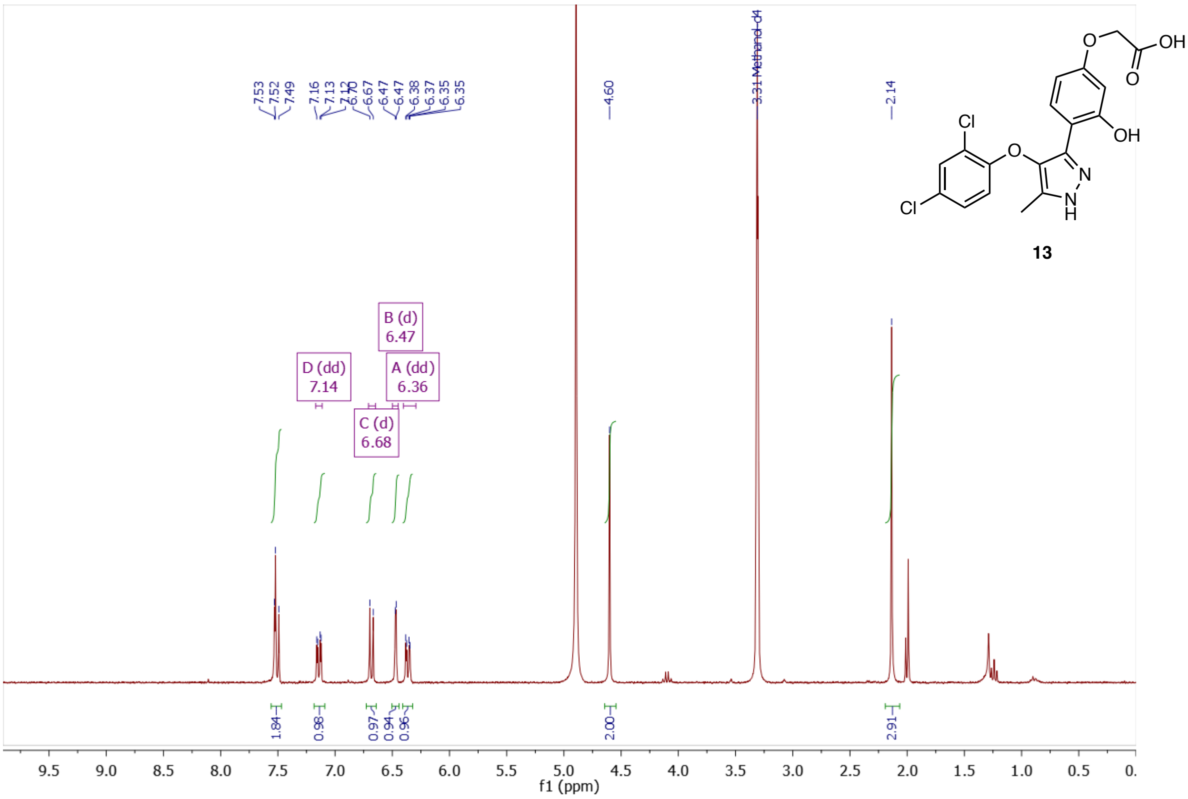

^{13}C NMR spectrum for **13** (CD_3OD, 125 MHz):

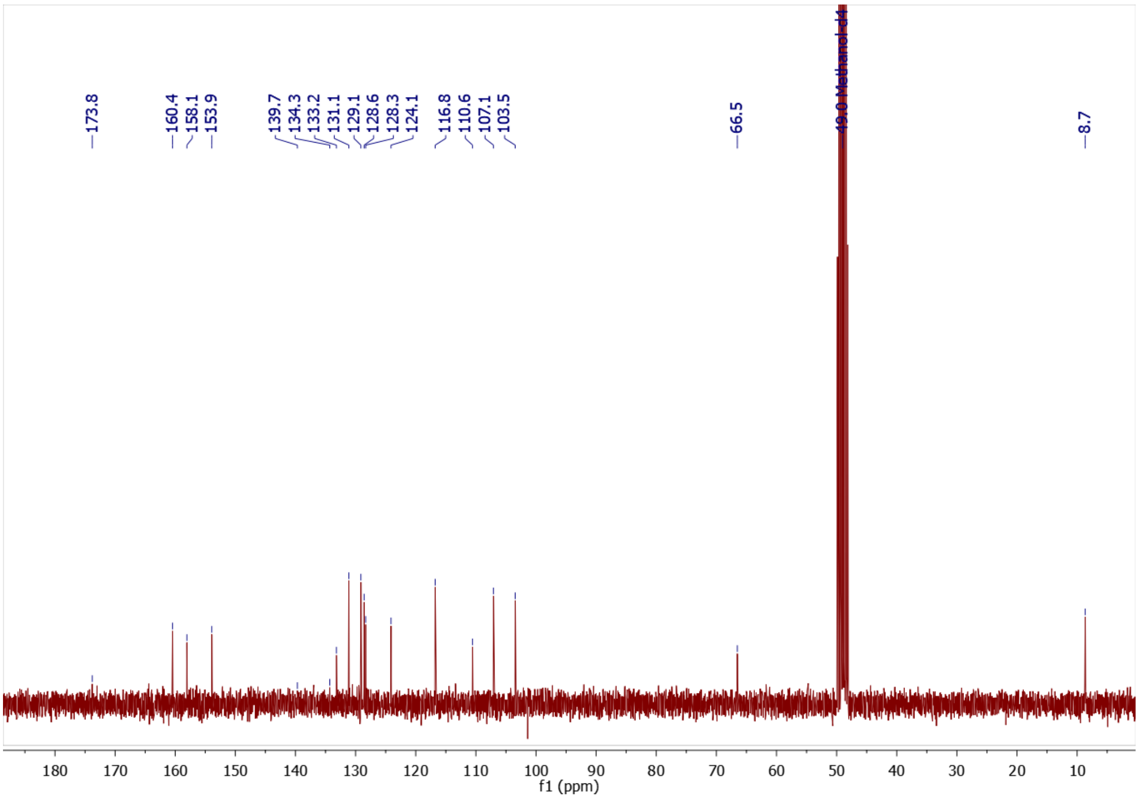

HPLC traces of compound **13**:

(ESI negative mode)

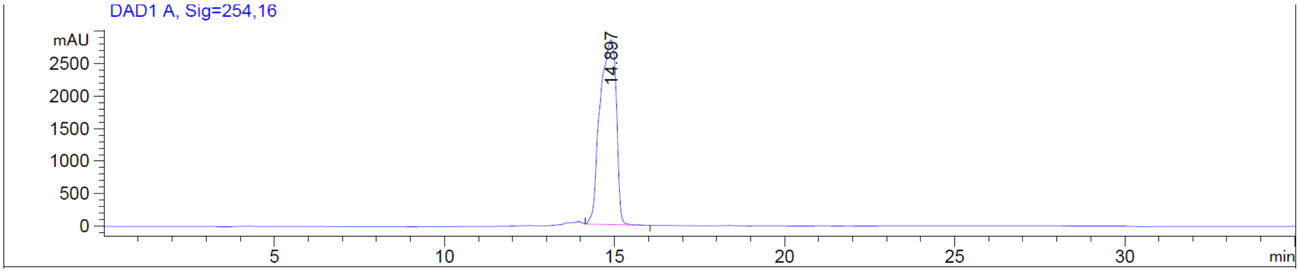

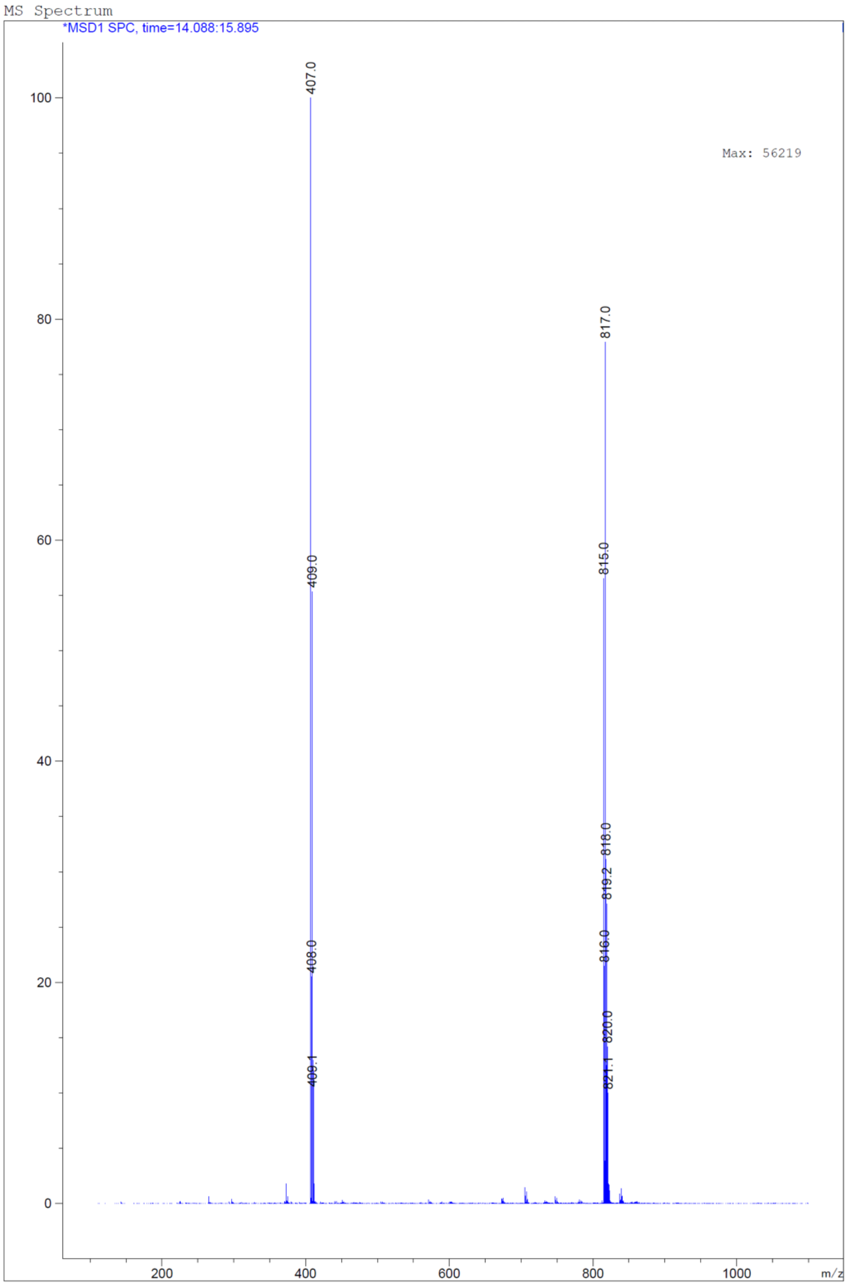

^{1}H NMR spectrum for **30** (CD_3OD, 700 MHz):

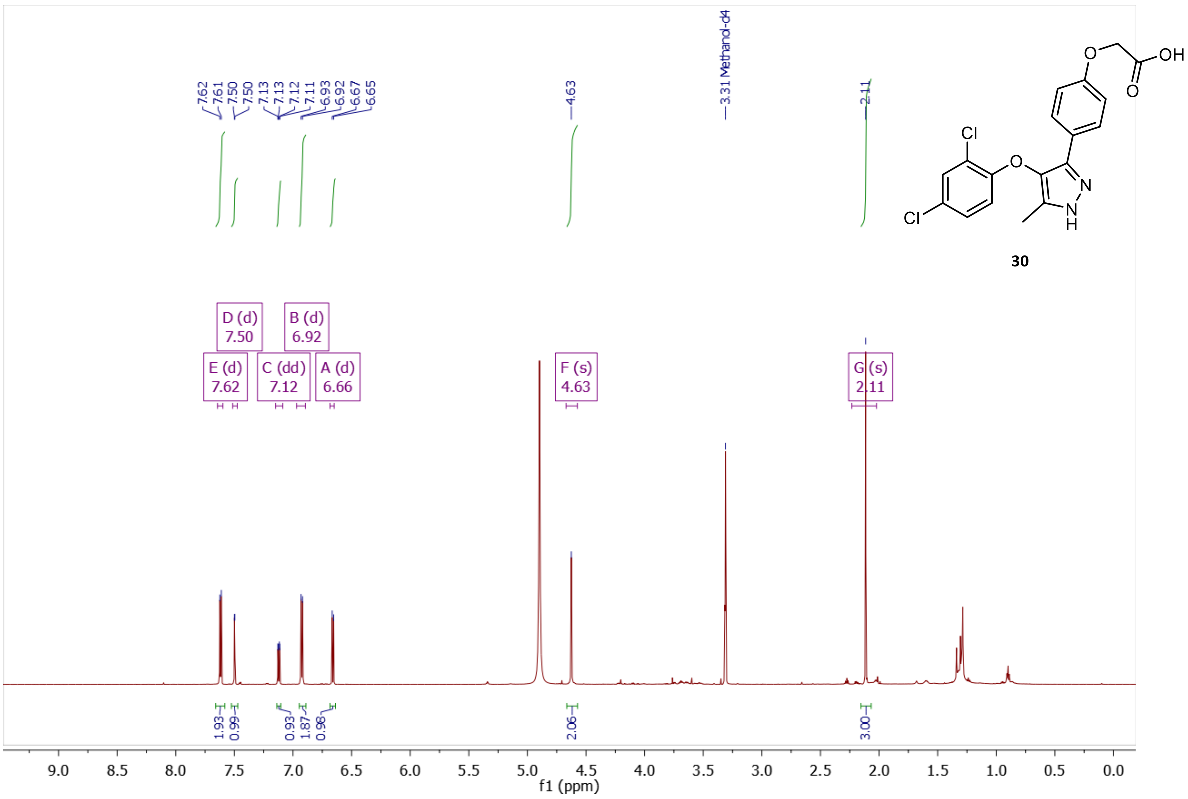

HPLC traces of compound **30**:

(ESI negative mode)

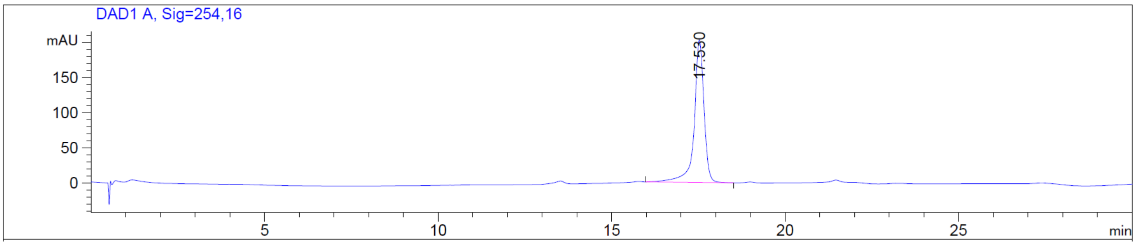

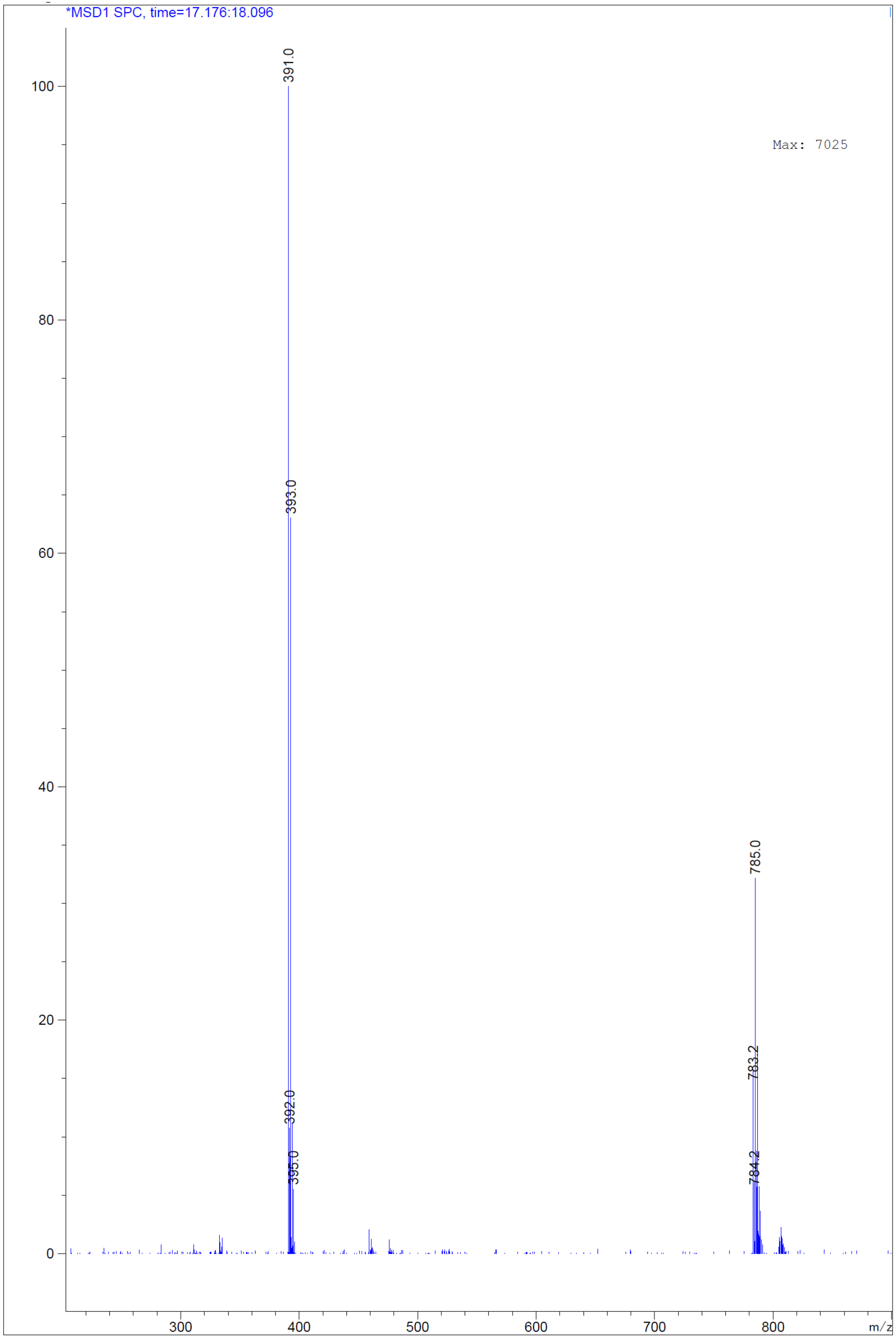

^{1}H NMR spectrum for **37** (CD_3OD, 700 MHz):

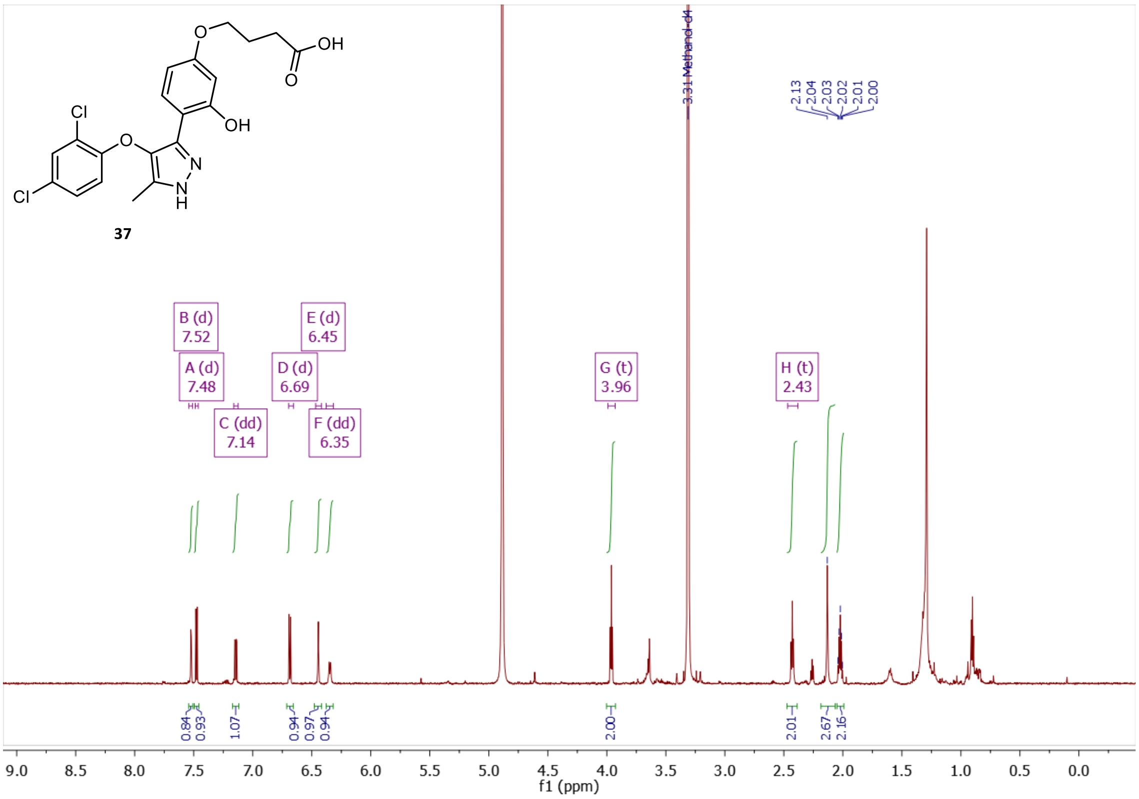

HPLC traces of compound **37**:

(ESI negative mode)

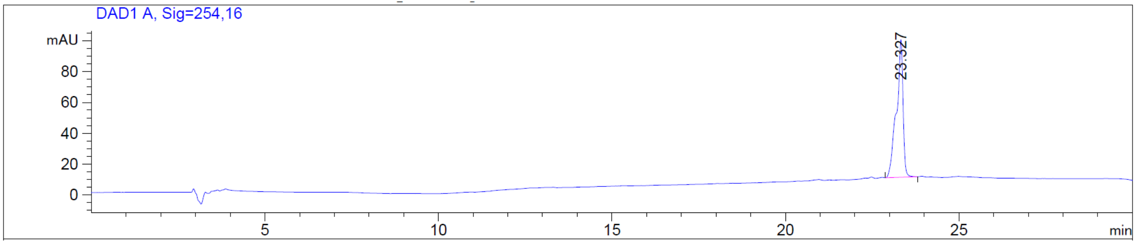

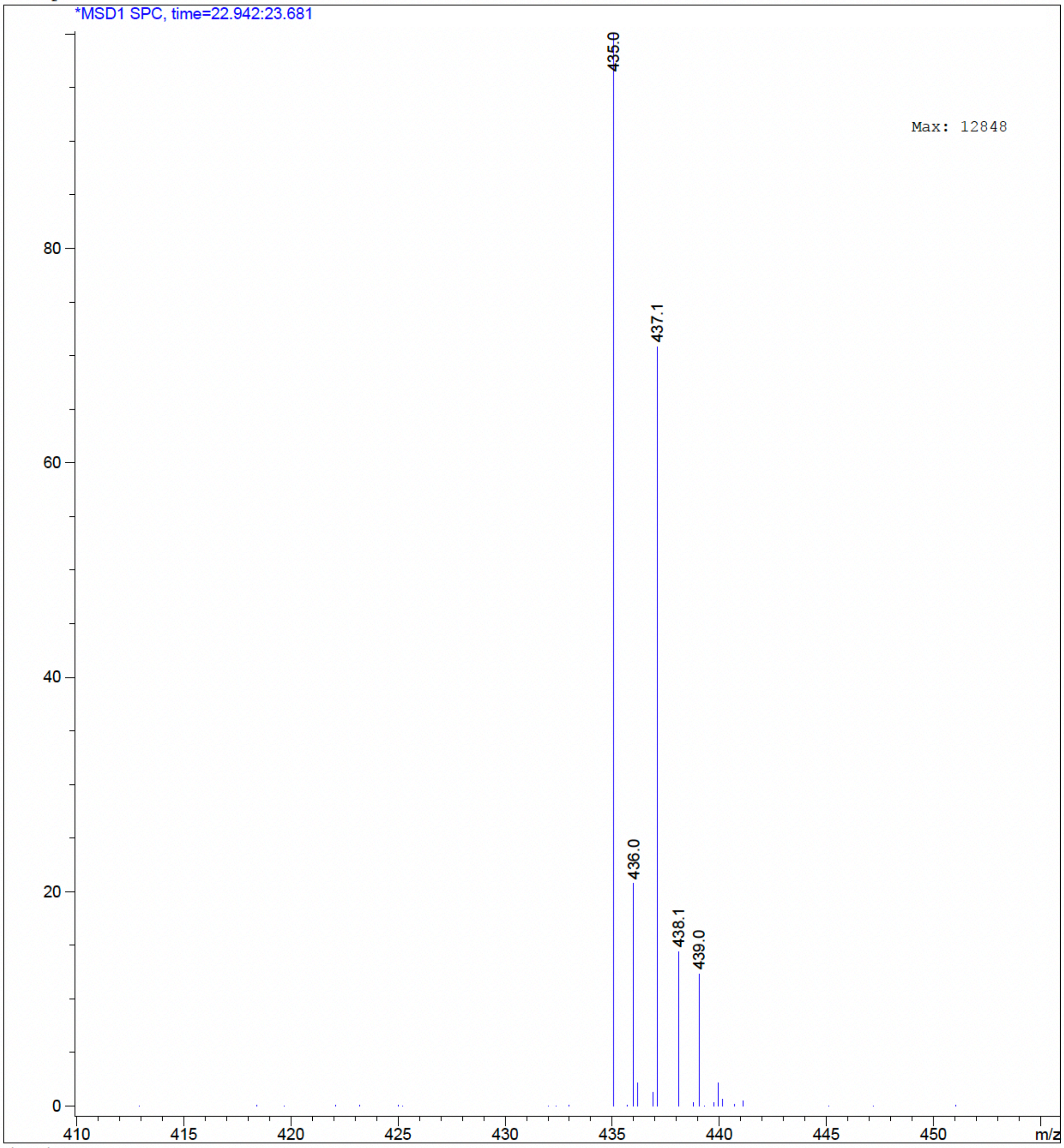

^{1}H NMR spectrum for **38** (CD_3OD, 700 MHz):

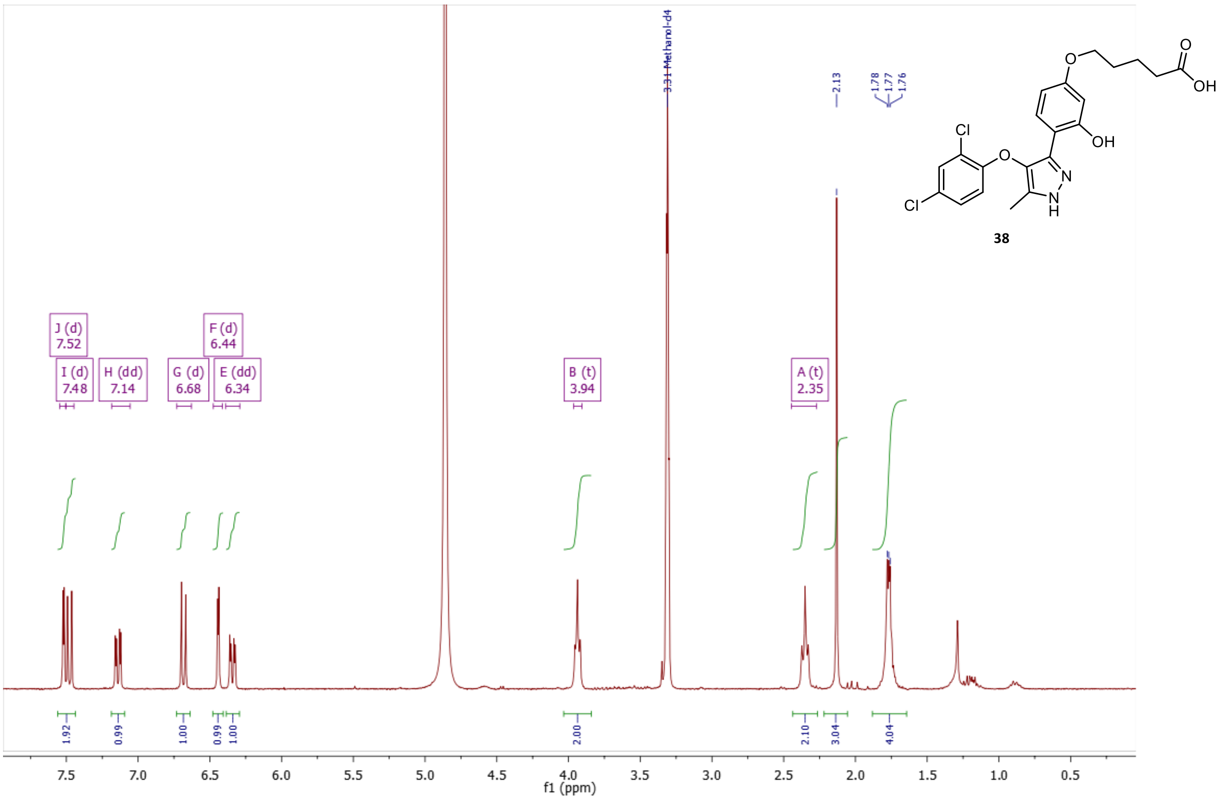

^{13}C NMR spectrum for **38** (CD_3OD, 175 MHz):

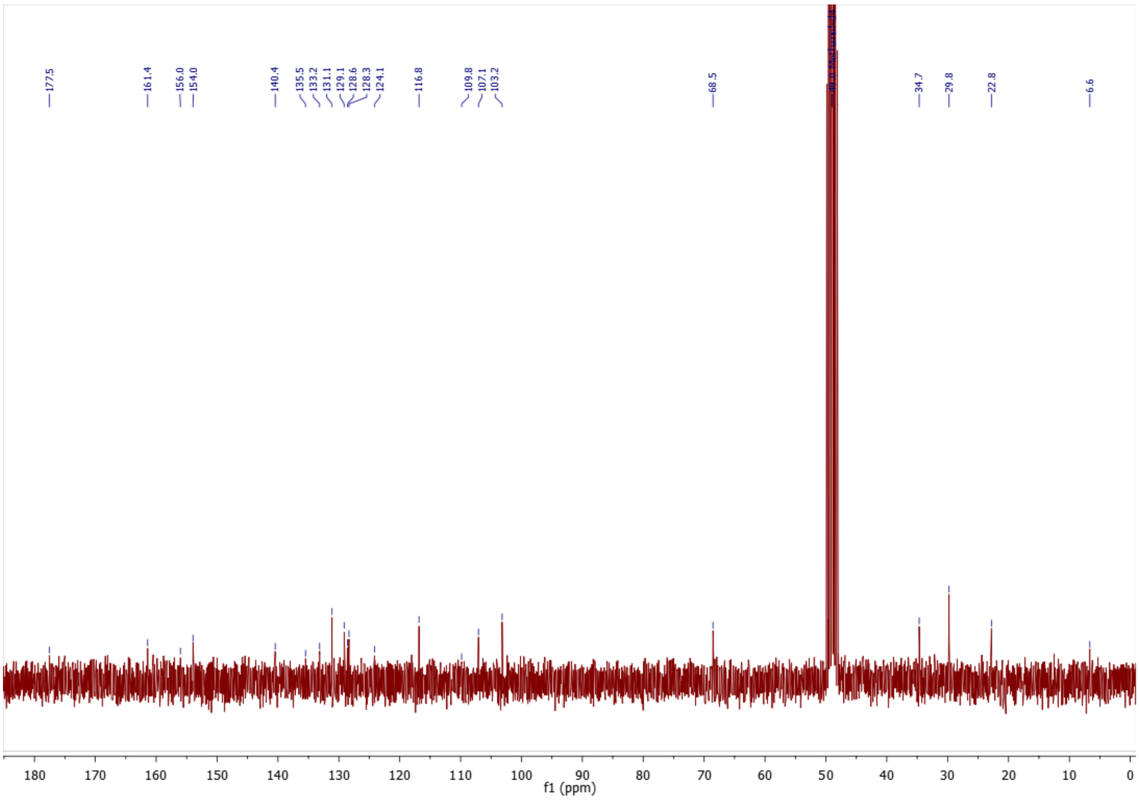

MS ESI positive mode

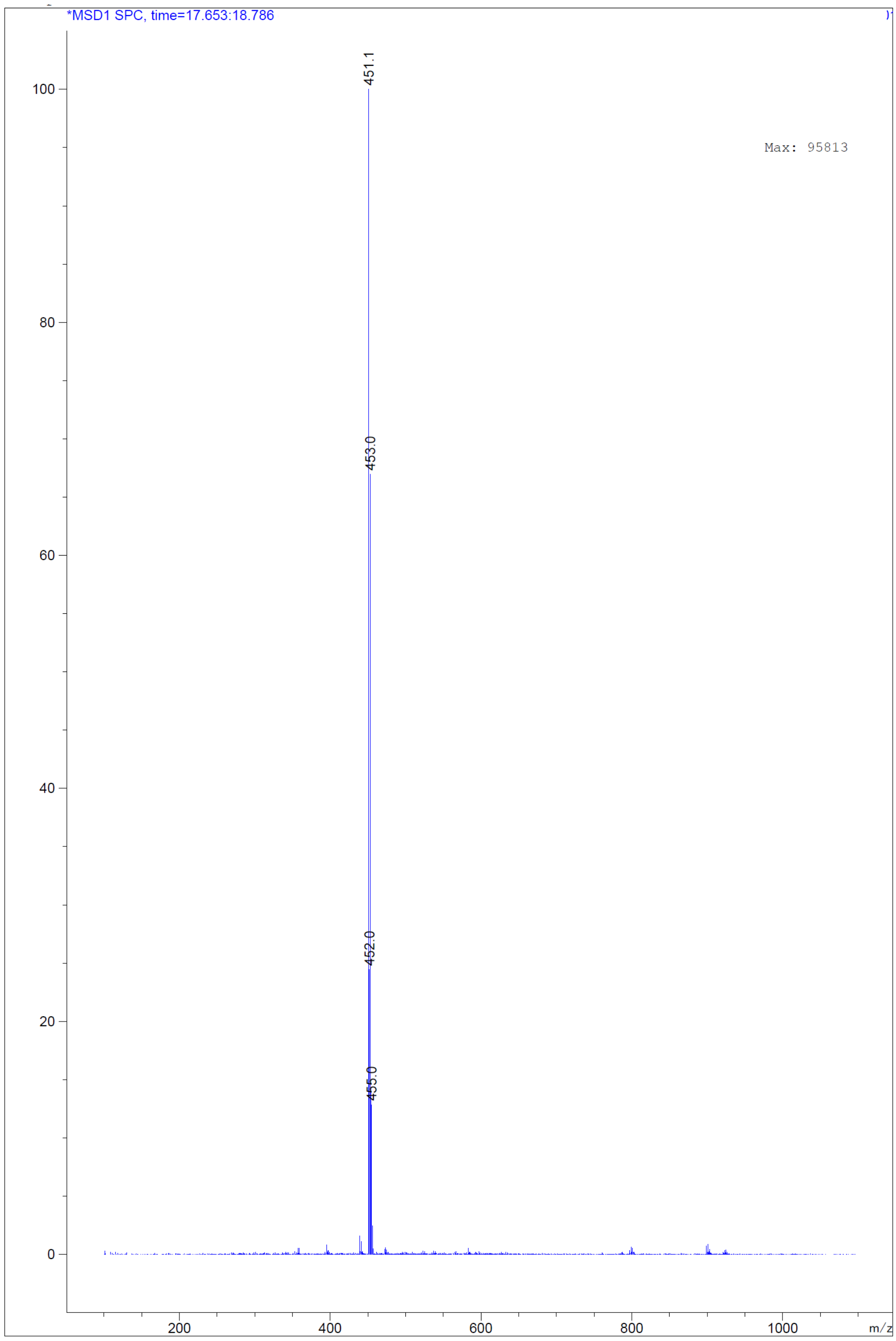

^{1}H NMR spectrum for **42** (CD_3OD, 300 MHz):

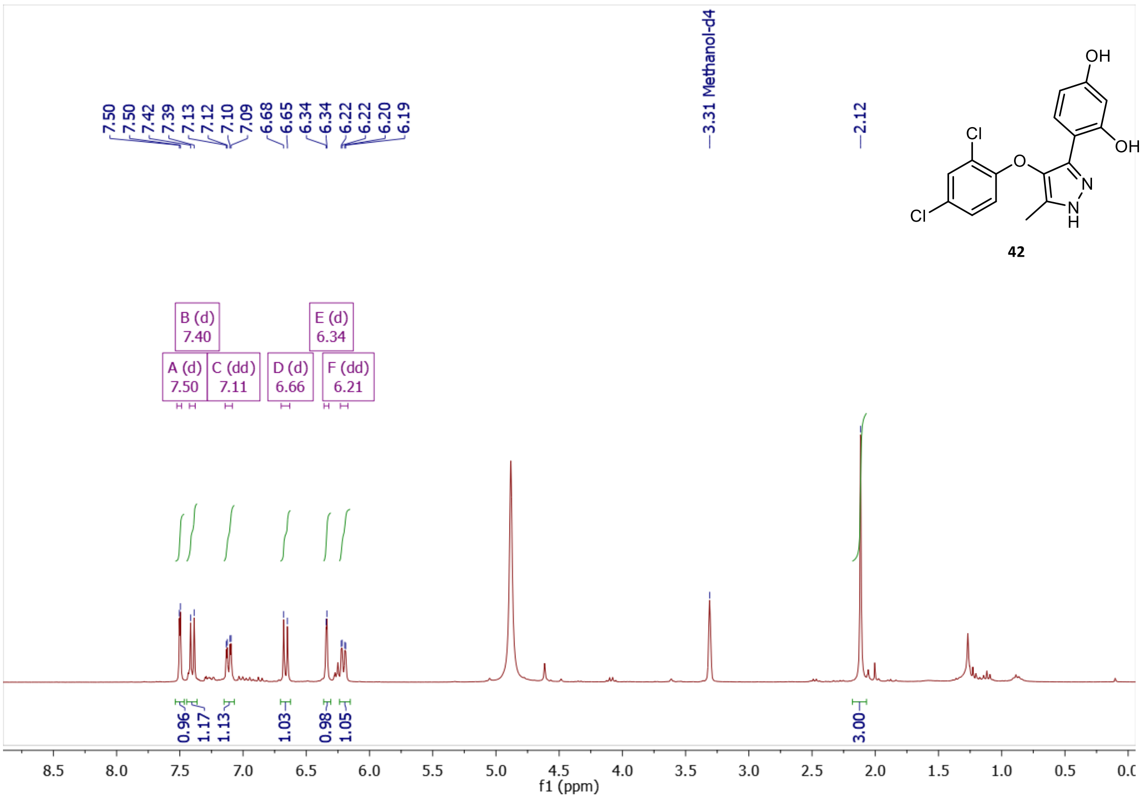

^{13}C NMR spectrum for **42** (CD_3OD, 75 MHz):

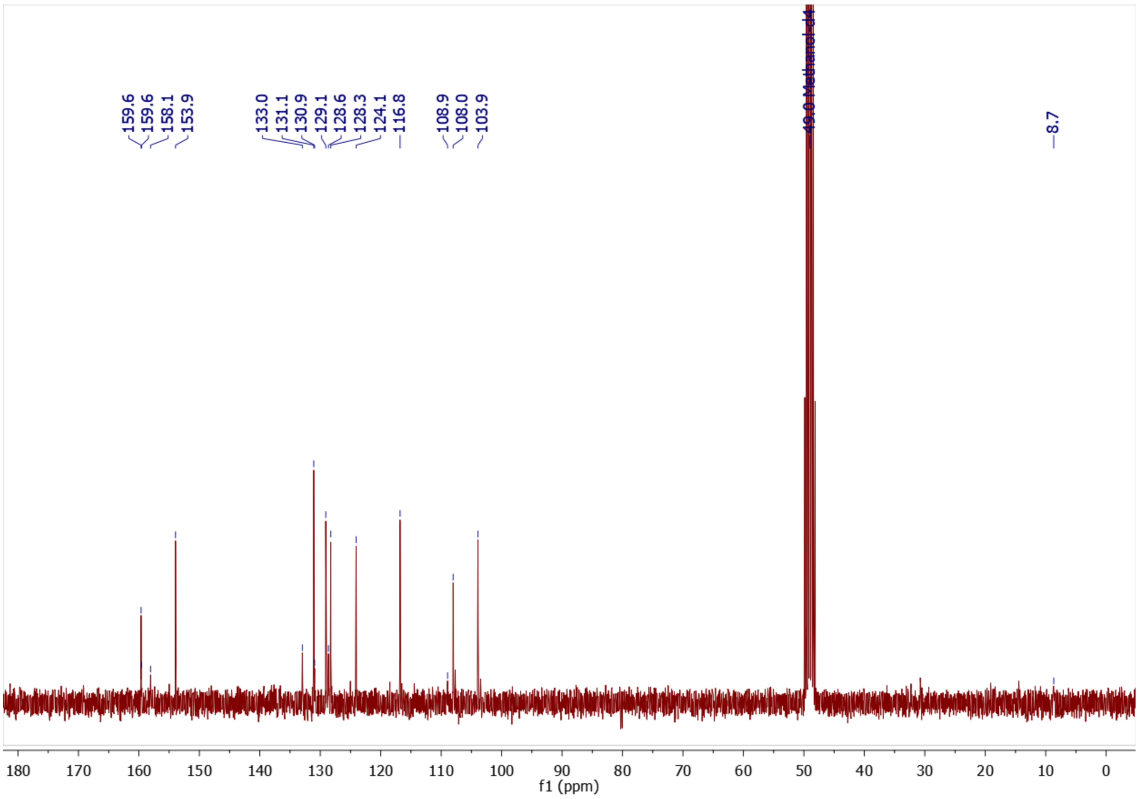

HPLC traces of compound **42**:

(ESI negative mode)

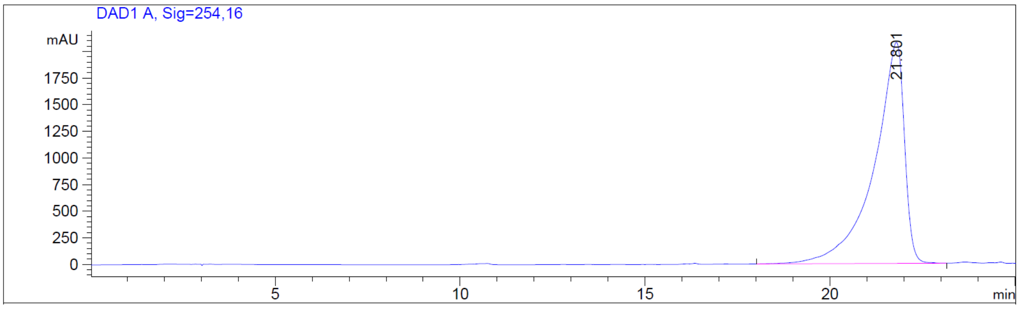

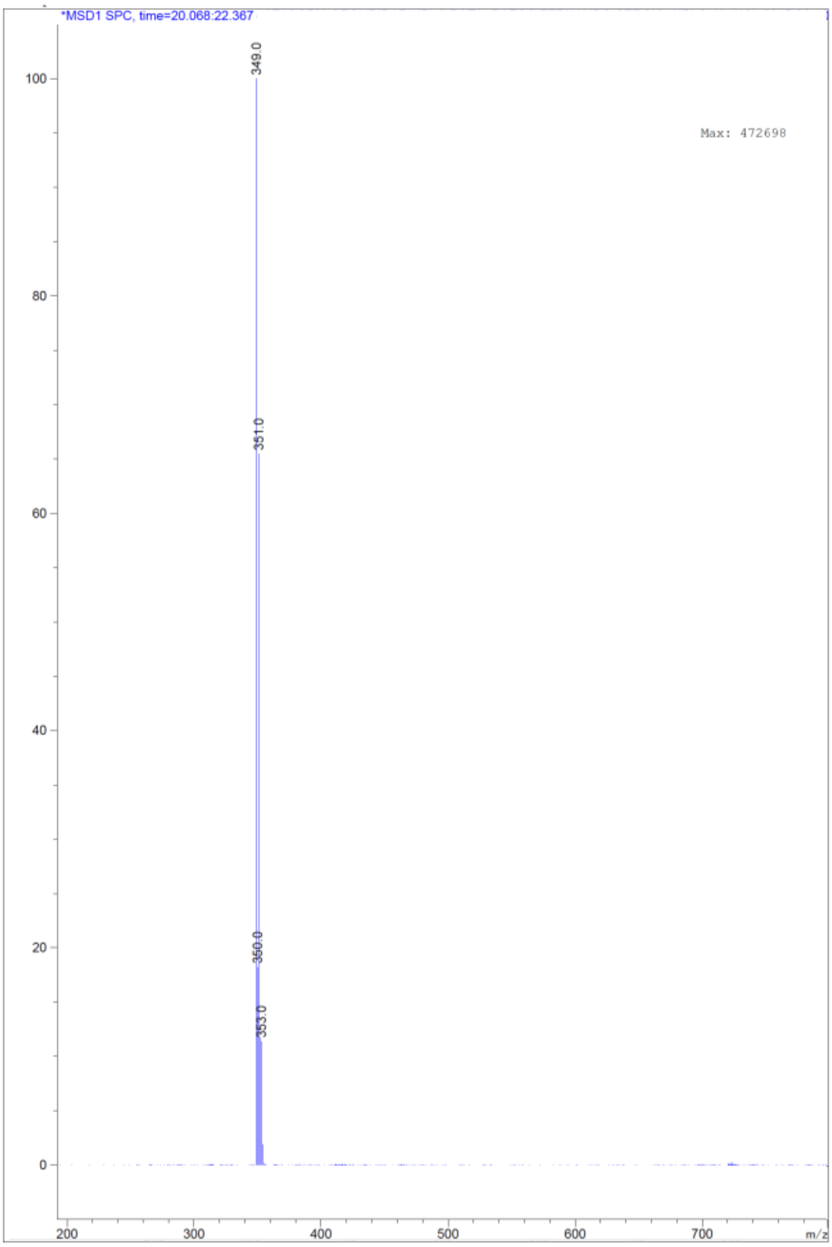

^{1}H NMR spectrum for **43** (CD_3OD, 700 MHz):

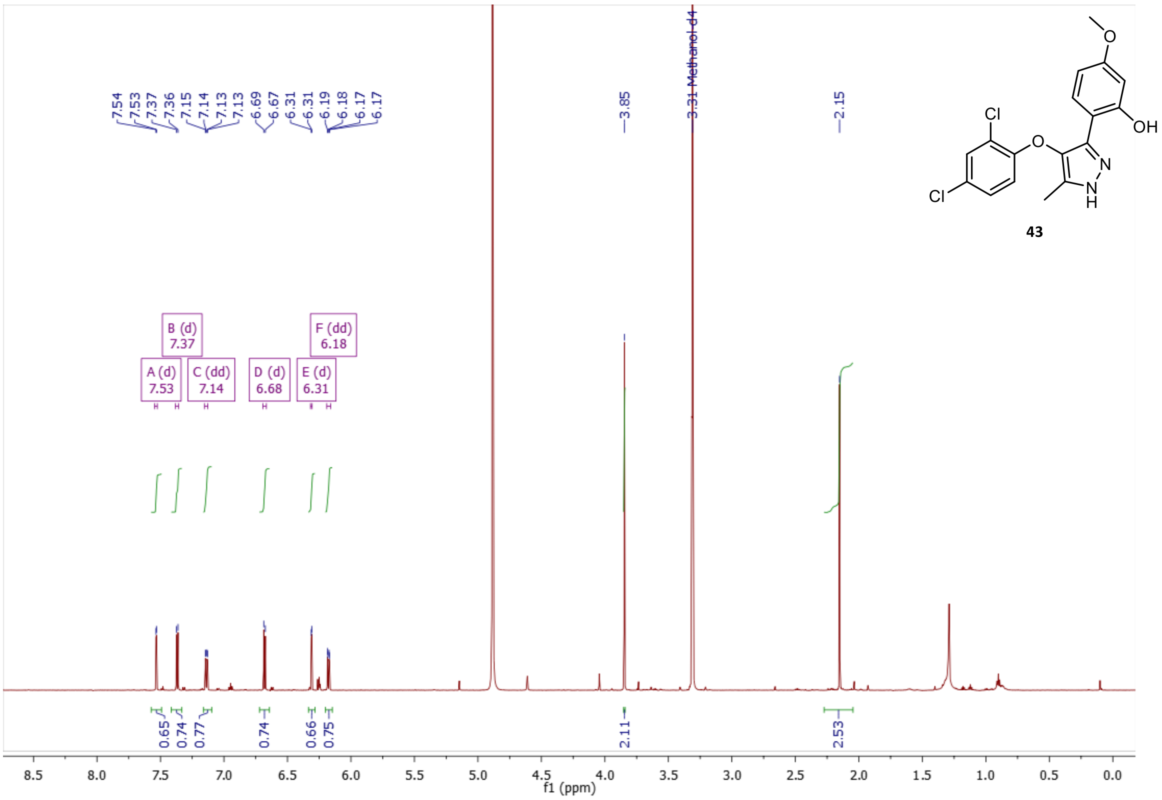

HPLC traces of compound **43**:

(ESI negative mode)

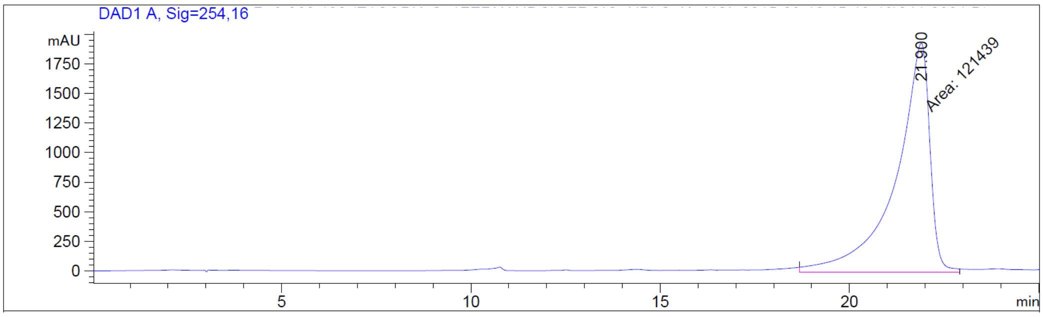

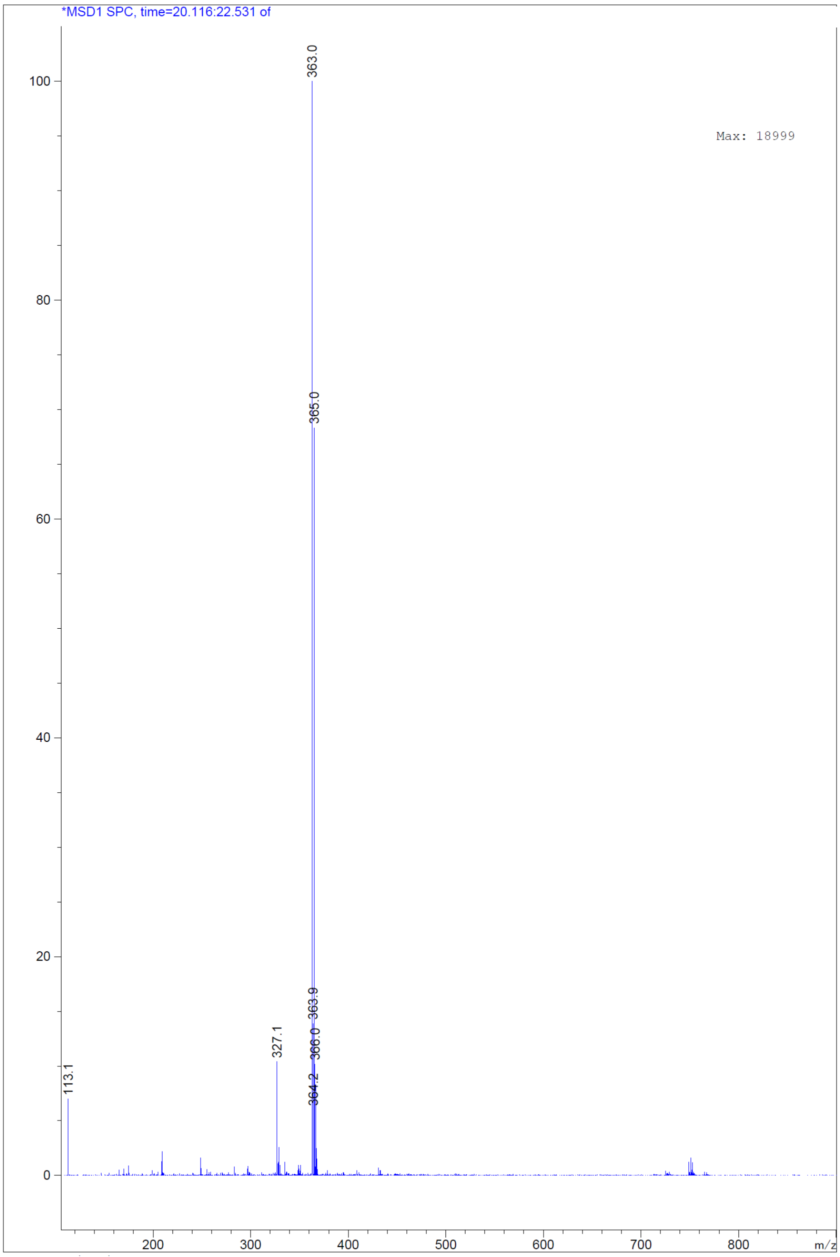

^{1}H NMR spectrum for **44** (CD_3OD, 700 MHz):

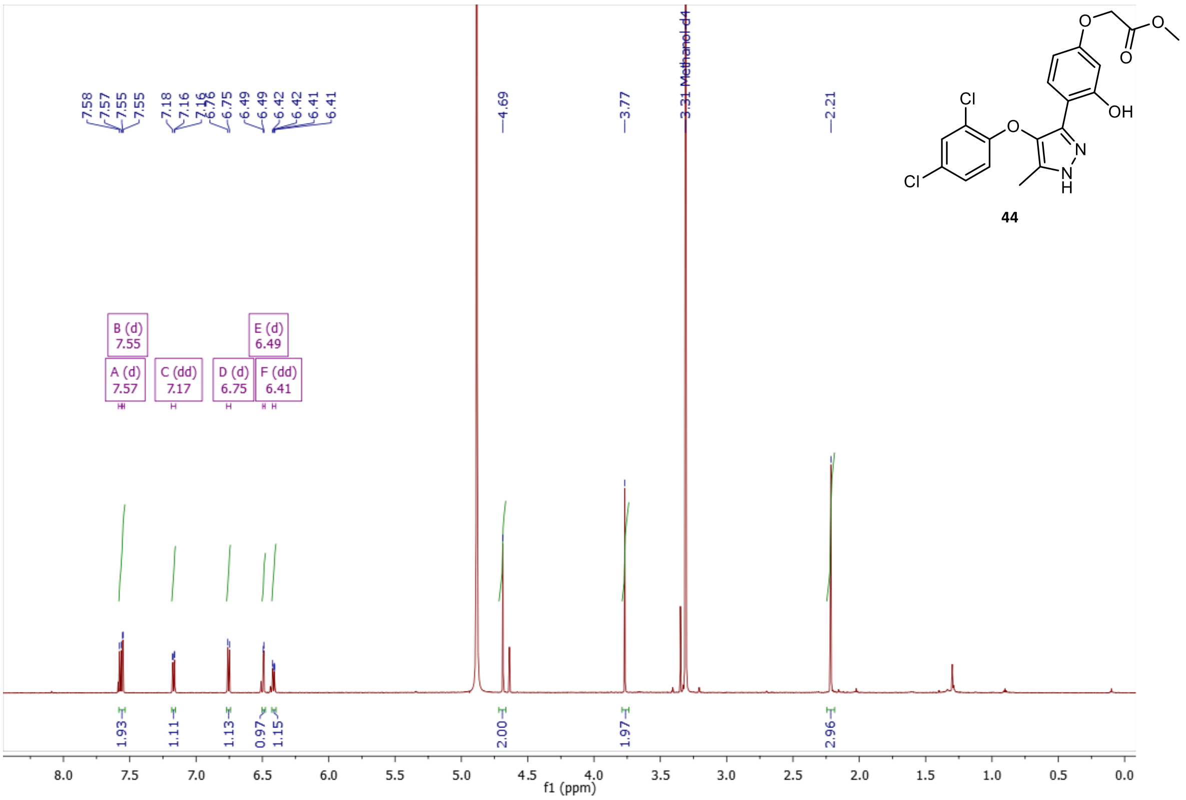

^{13}C NMR spectrum for **44** (CD_3OD, 175 MHz):

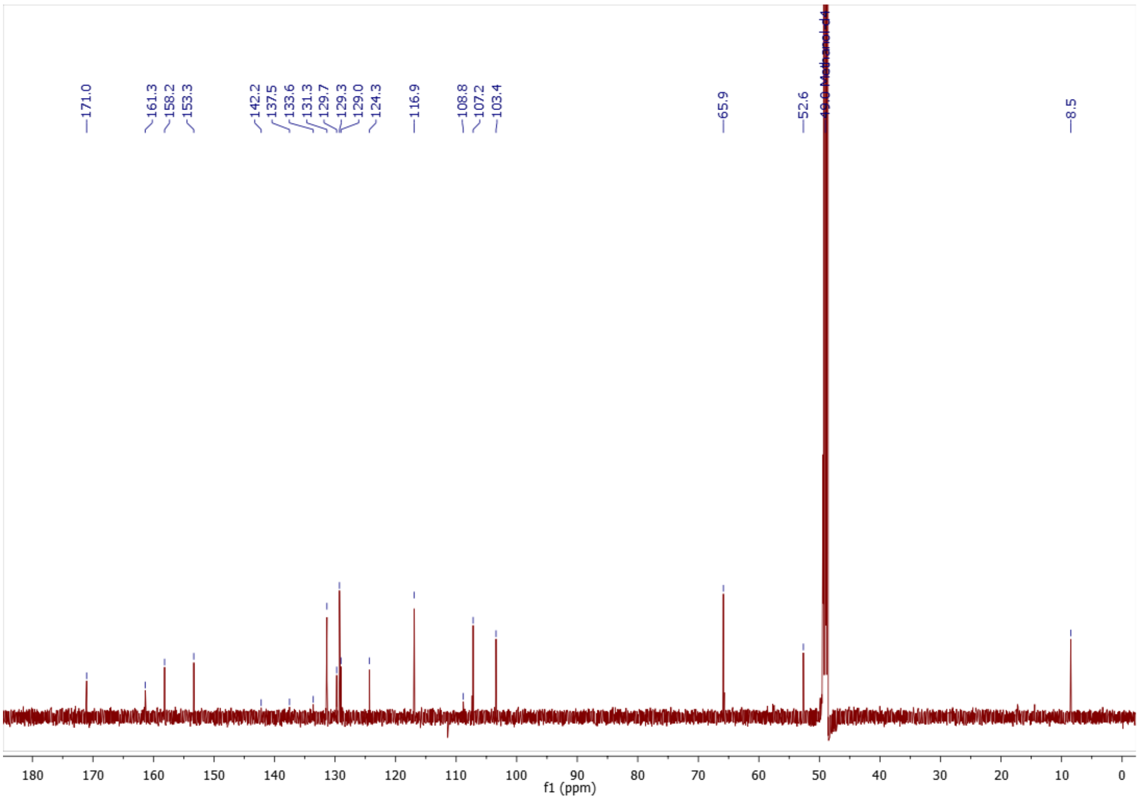

HPLC traces of compound **44**:

(ESI positive mode)

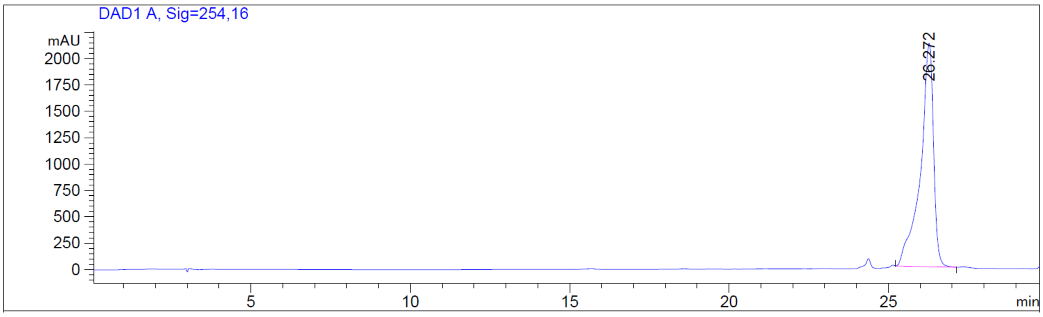

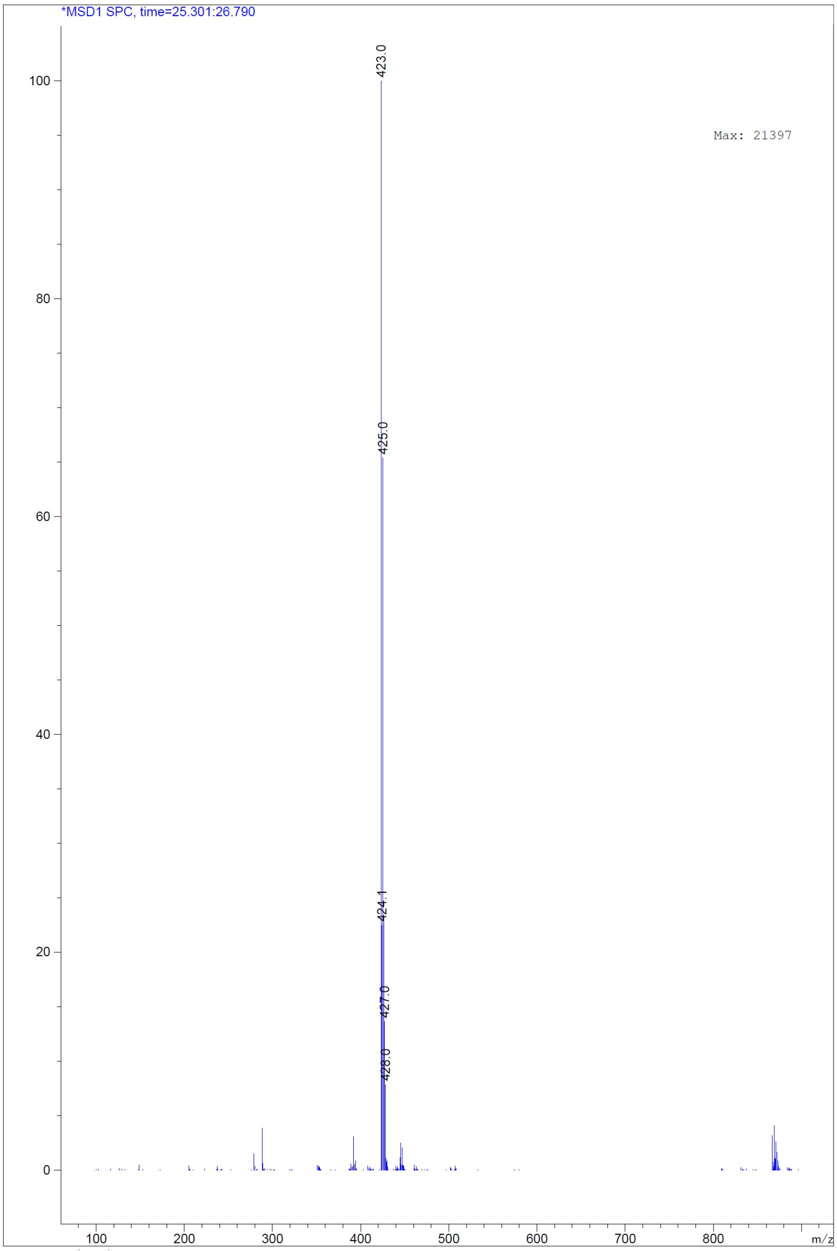

^{1}H NMR spectrum for **45** (CD_3OD, 700 MHz):

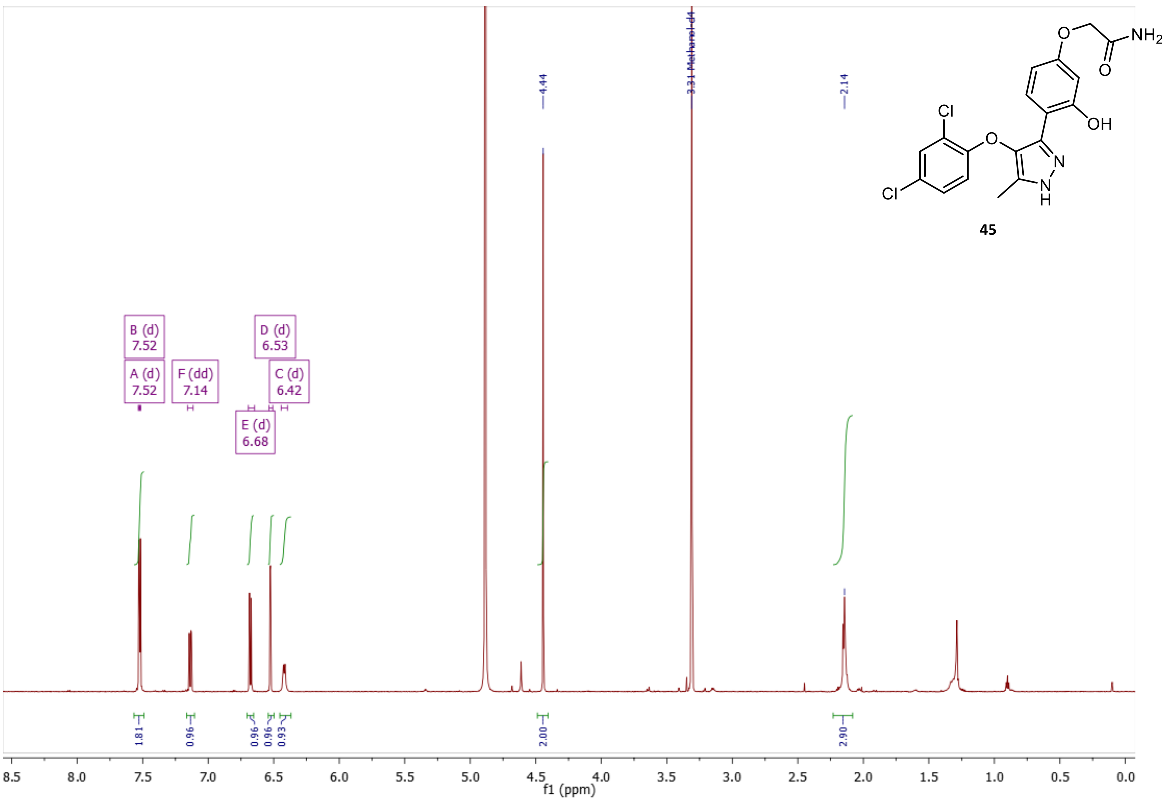

^{13}C NMR spectrum for **45** (CD_3OD, 175 MHz):

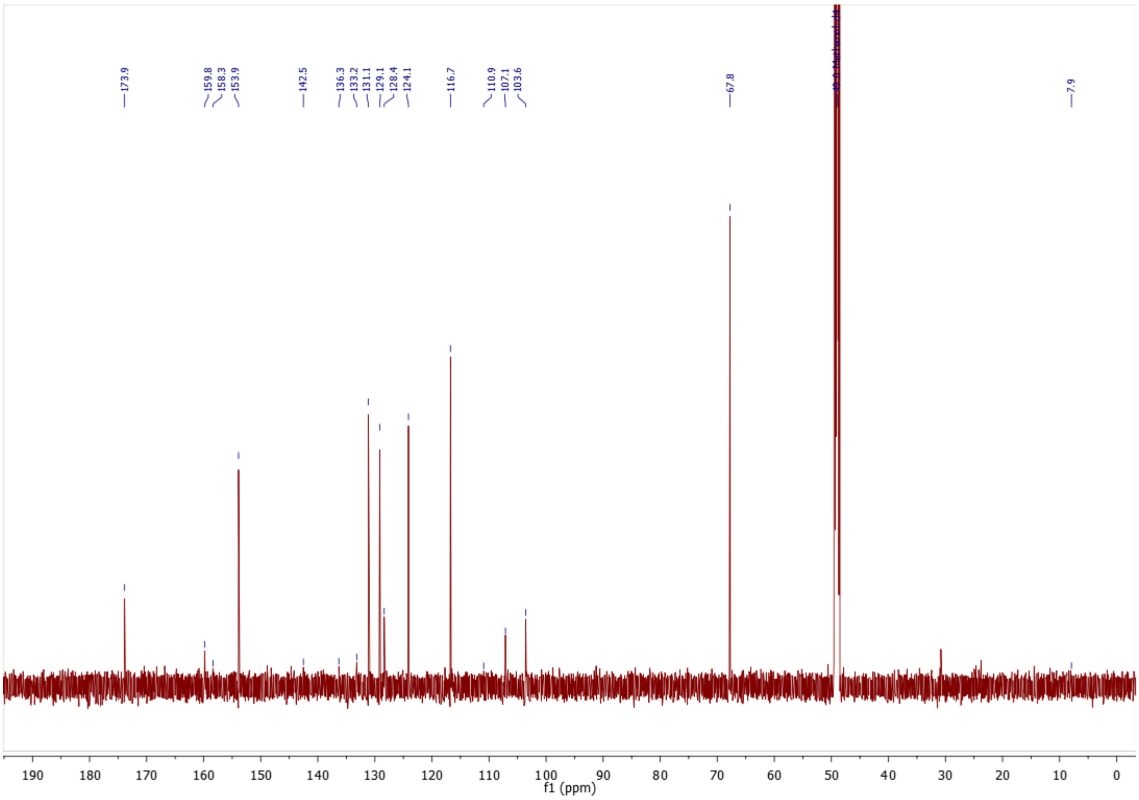

HPLC traces of compound **45**:

(ESI positive mode)

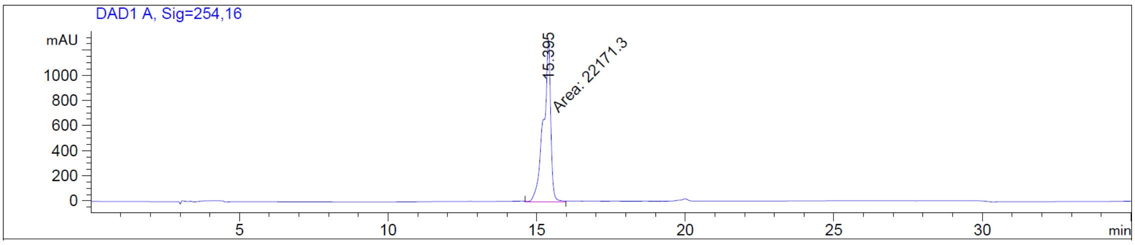

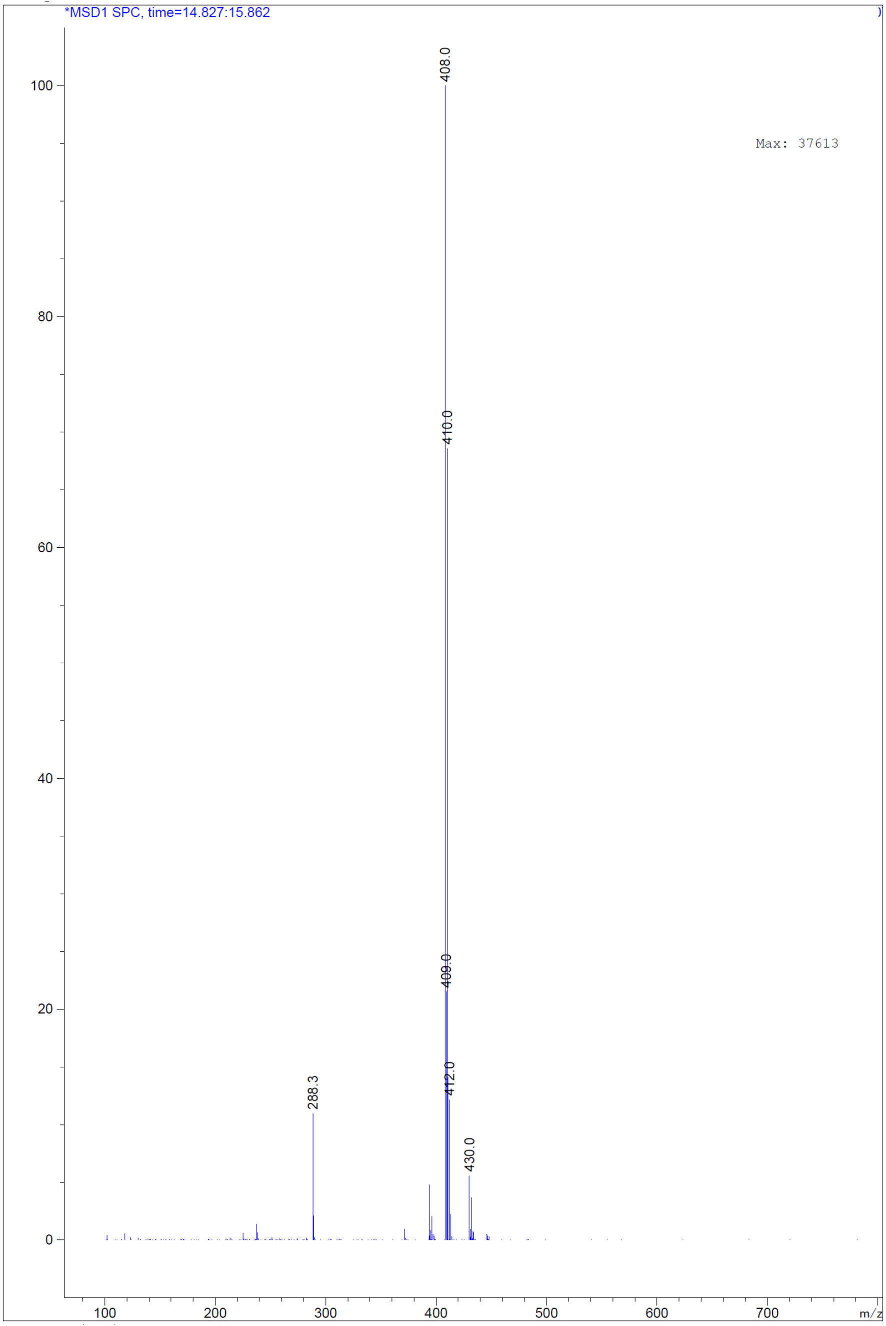

^{1}H NMR spectrum for **47** (CD_3OD, 700 MHz):

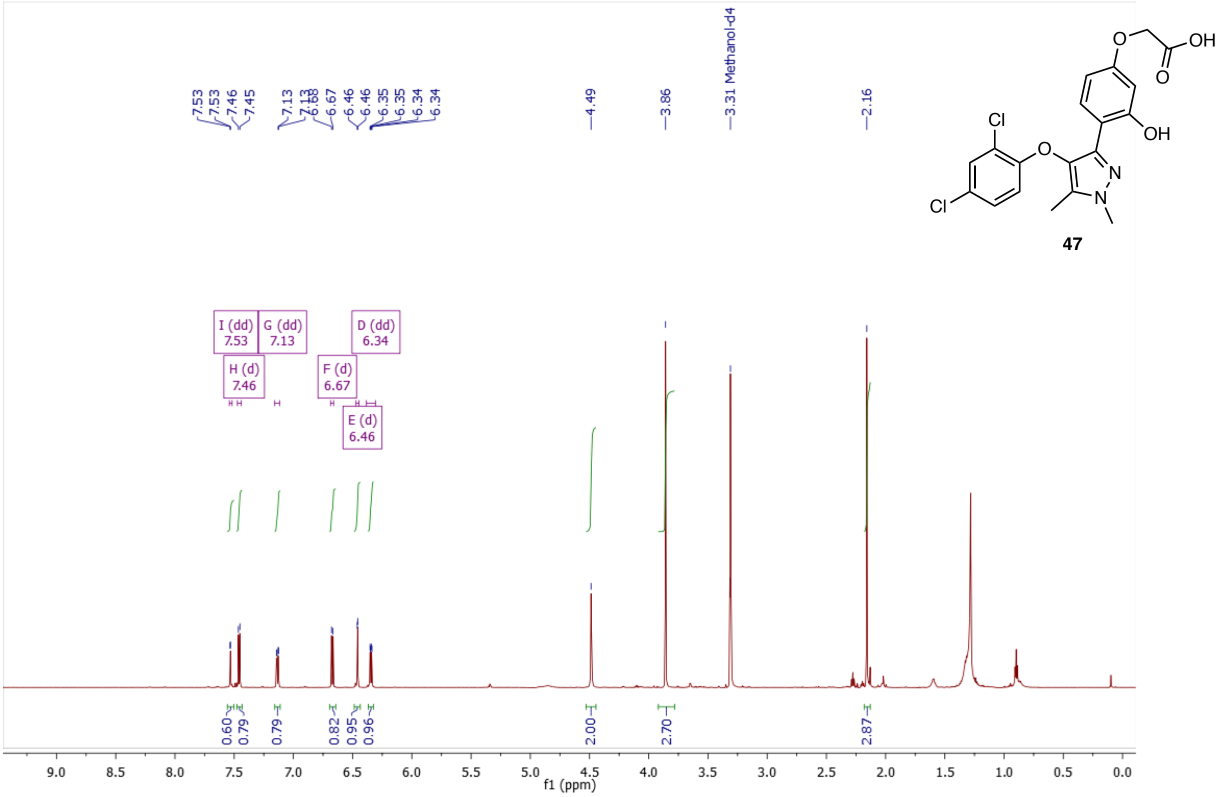

HPLC traces of compound **47**:

(ESI positive mode)

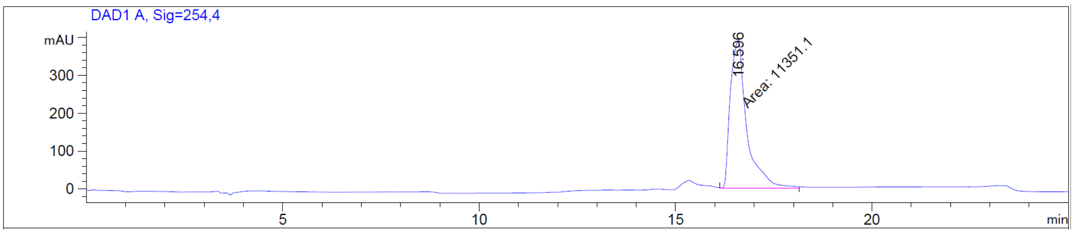

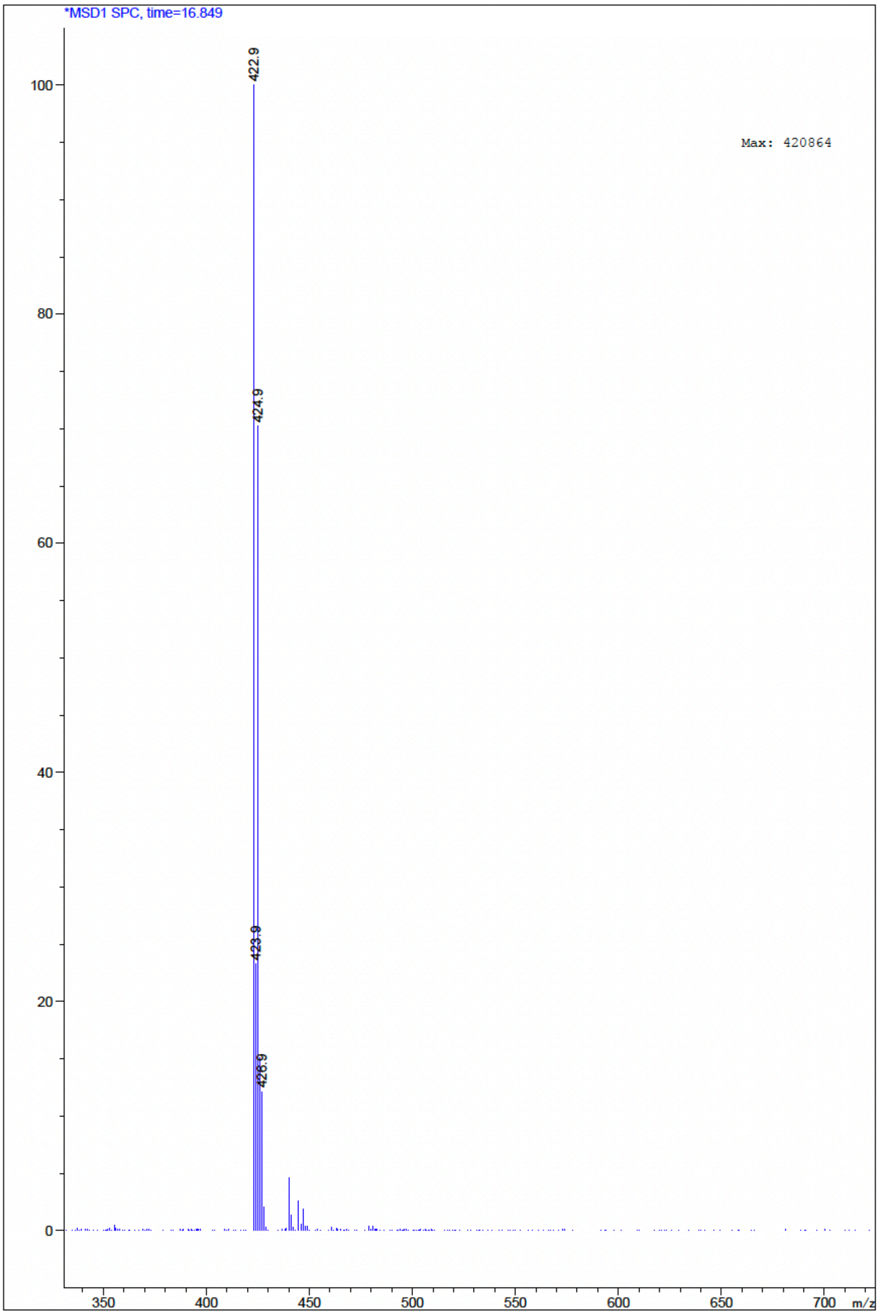

^{1}H NMR spectrum for **48** (CD_3OD, 700 MHz):

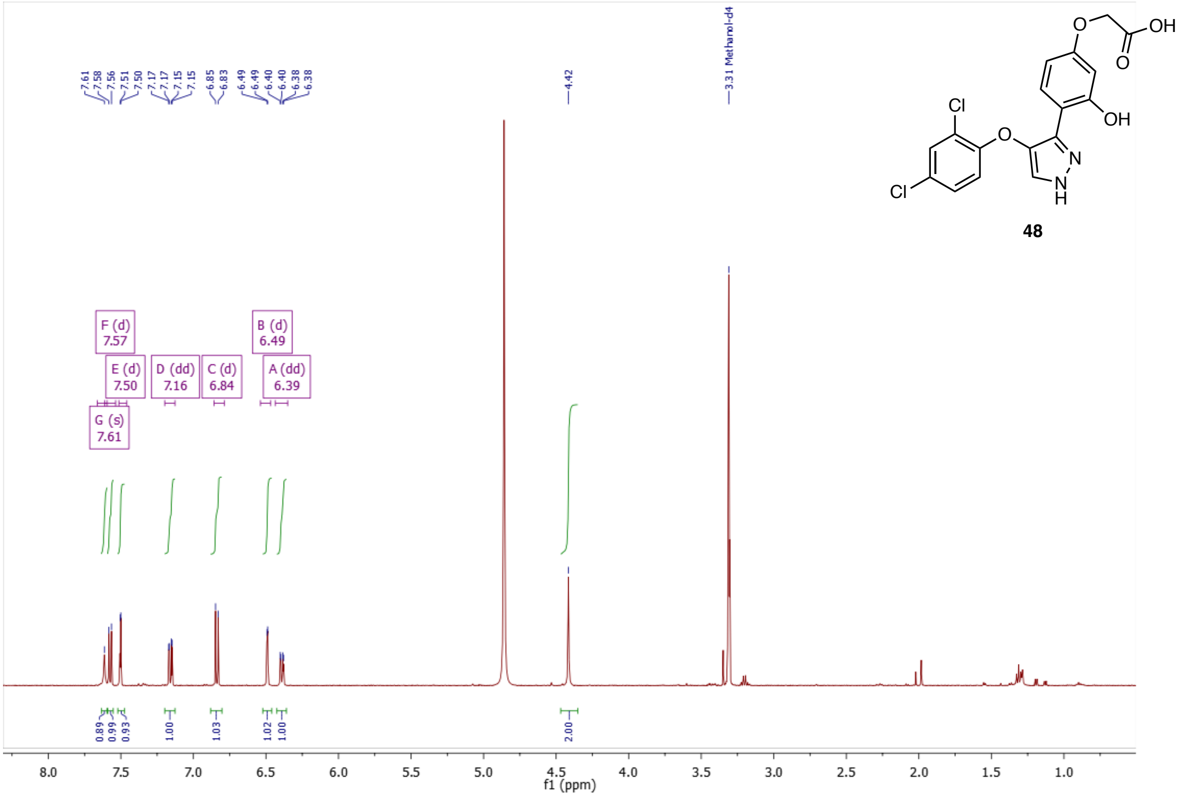

^{13}C NMR spectrum for **48** (CD_3OD, 175 MHz):

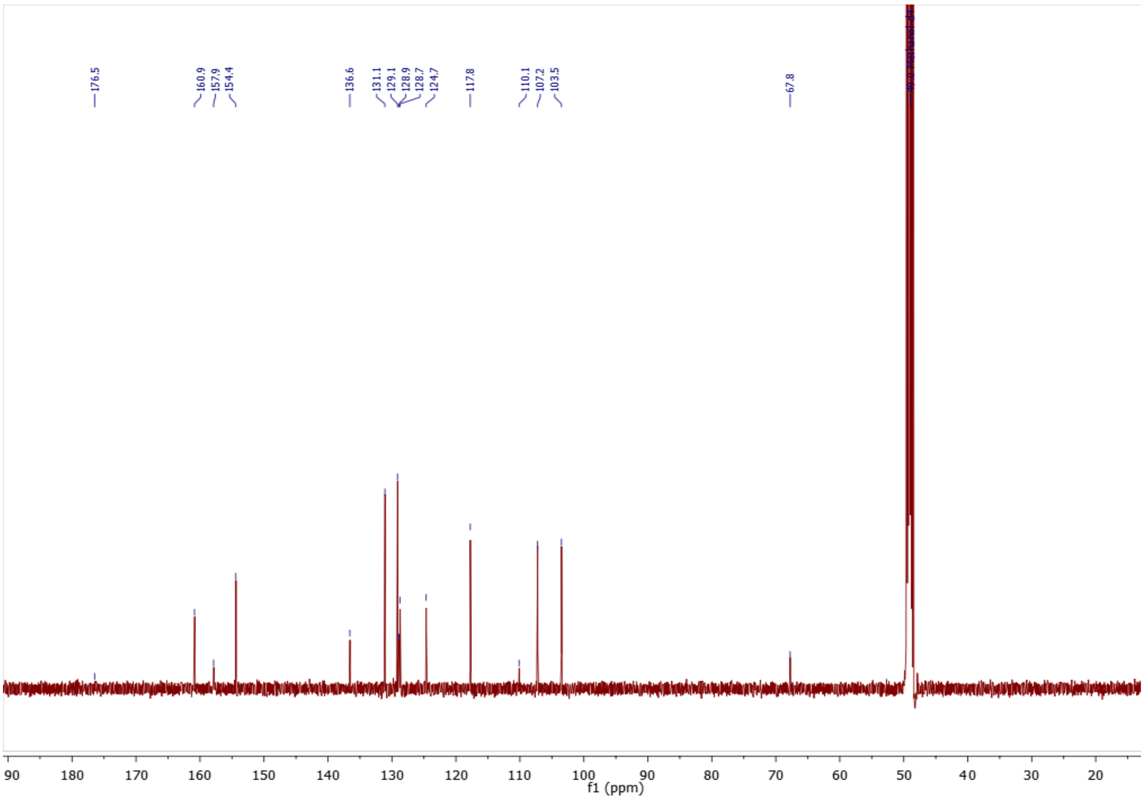

HPLC traces of compound **48**:

(ESI positive mode)

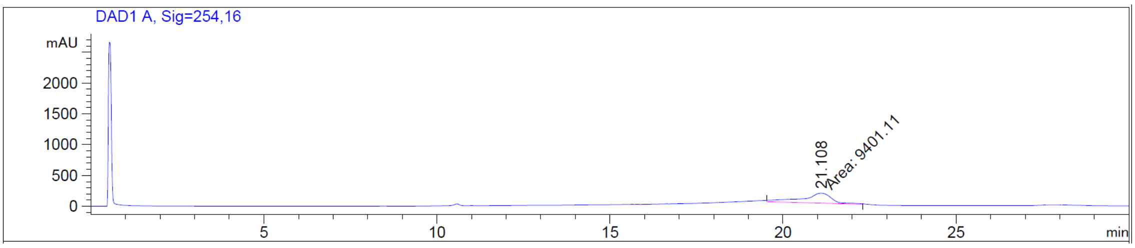

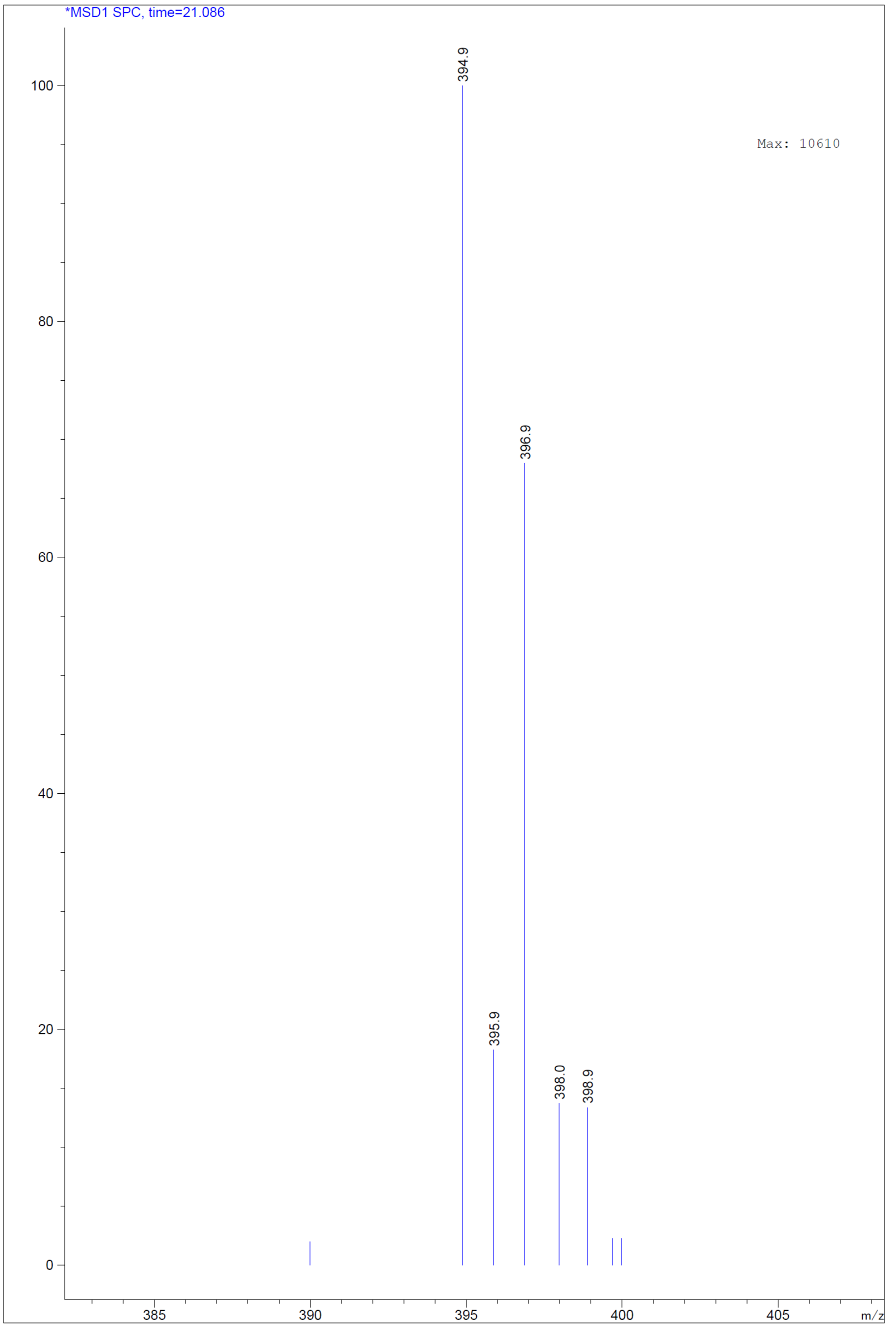

^{1}H NMR spectrum for **49** (CD_3OD, 700 MHz):

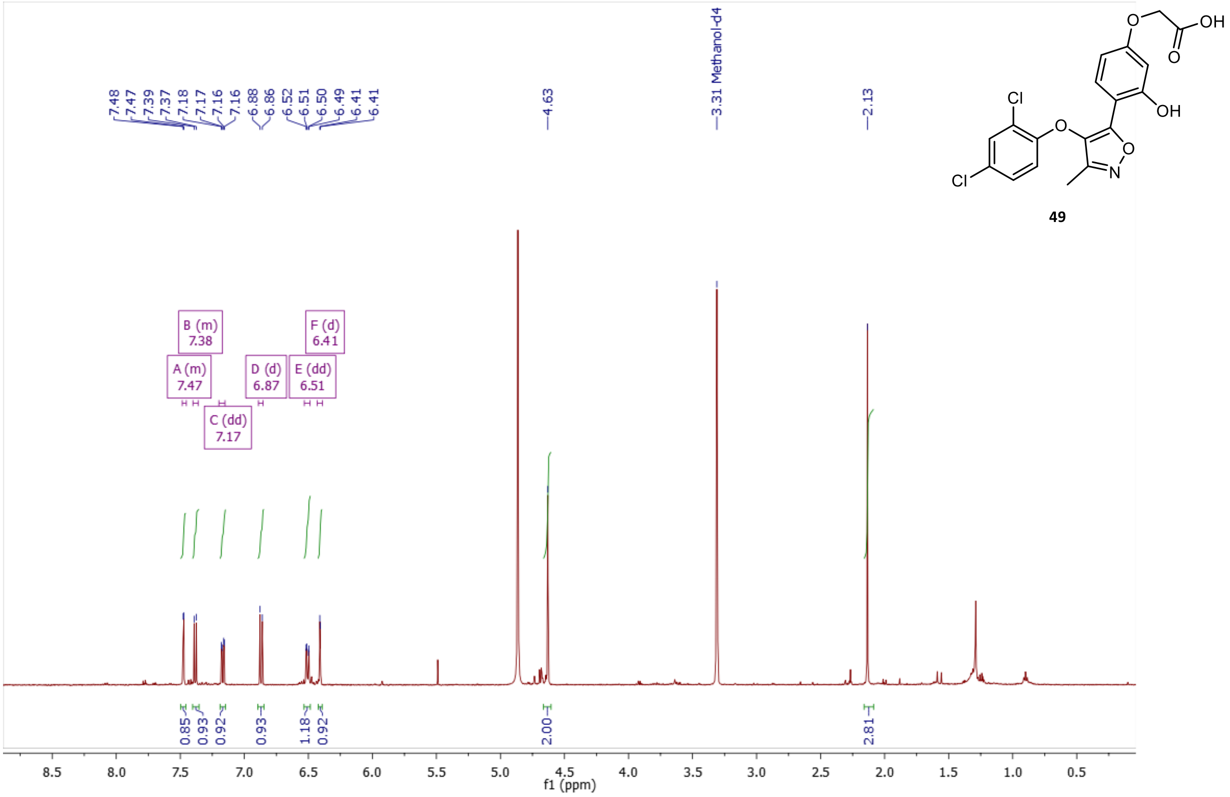

^{13}C NMR spectrum for **49** (CD_3OD, 175 MHz):

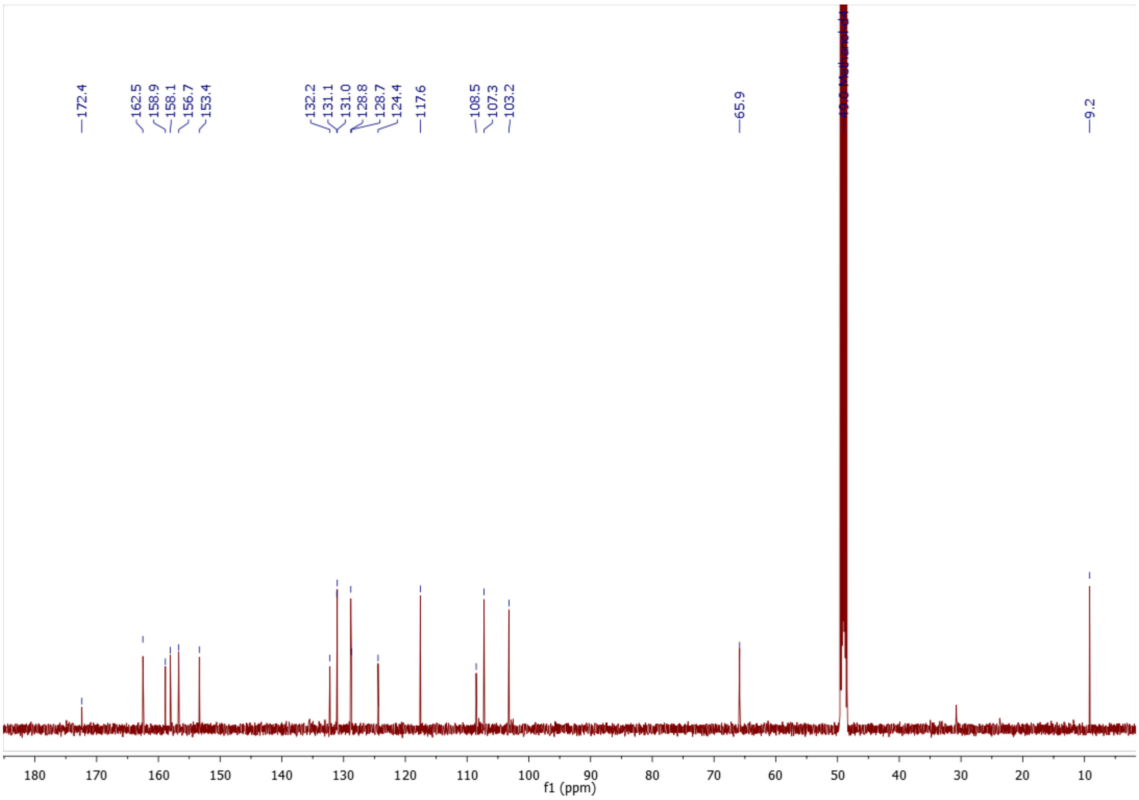

HPLC traces of compound **49**:

(ESI negative mode)

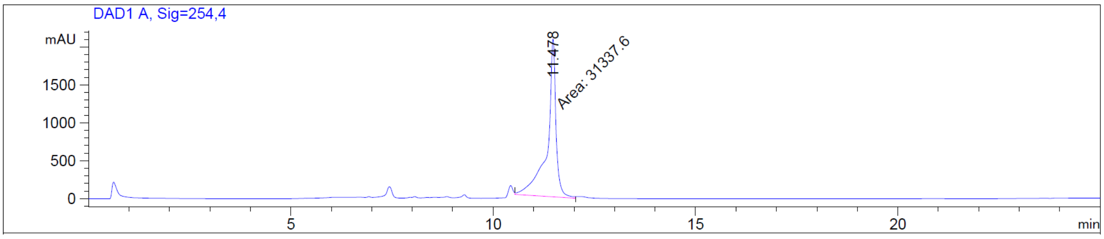

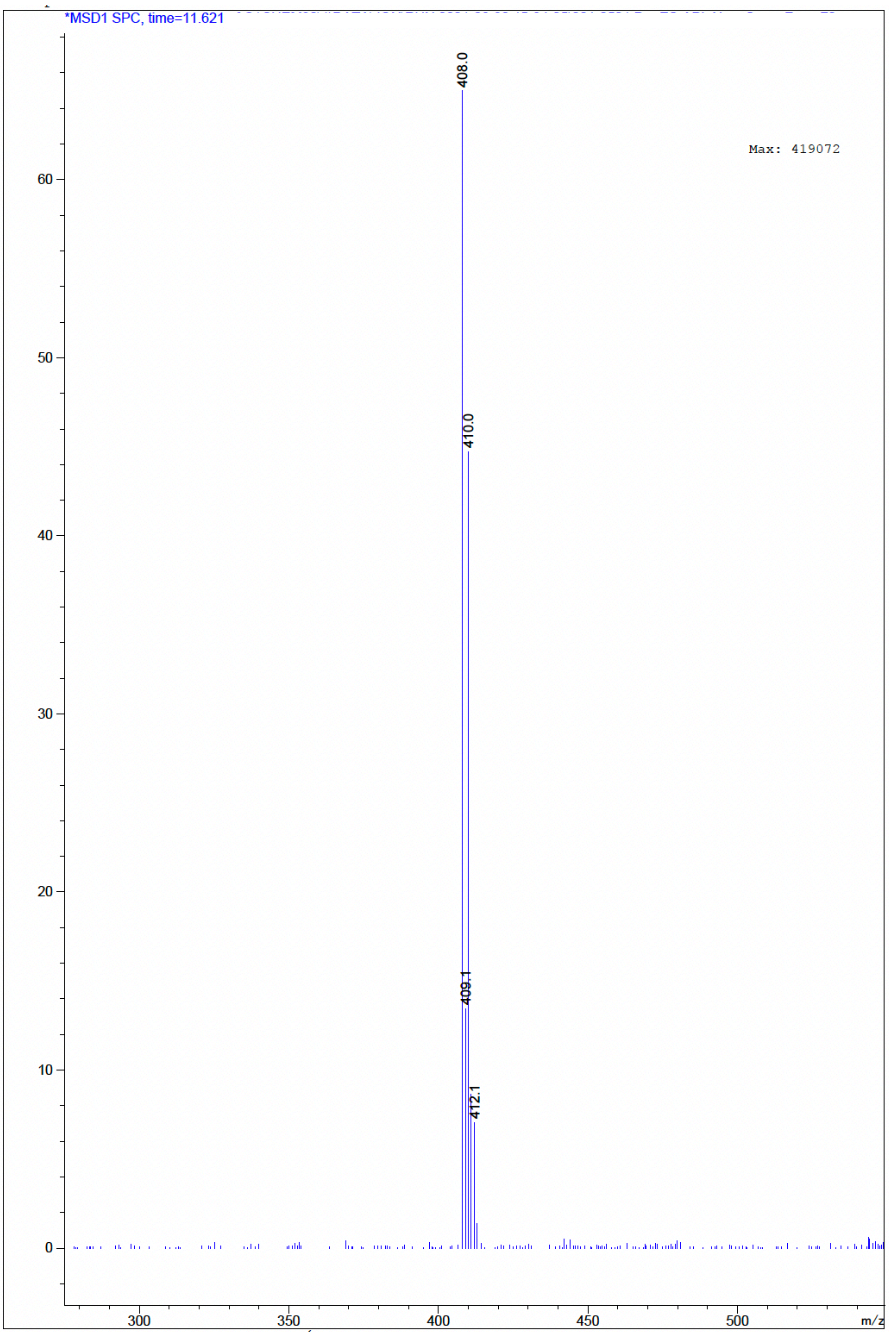

^{1}H NMR spectrum for **53** (CD_3OD, 700 MHz):

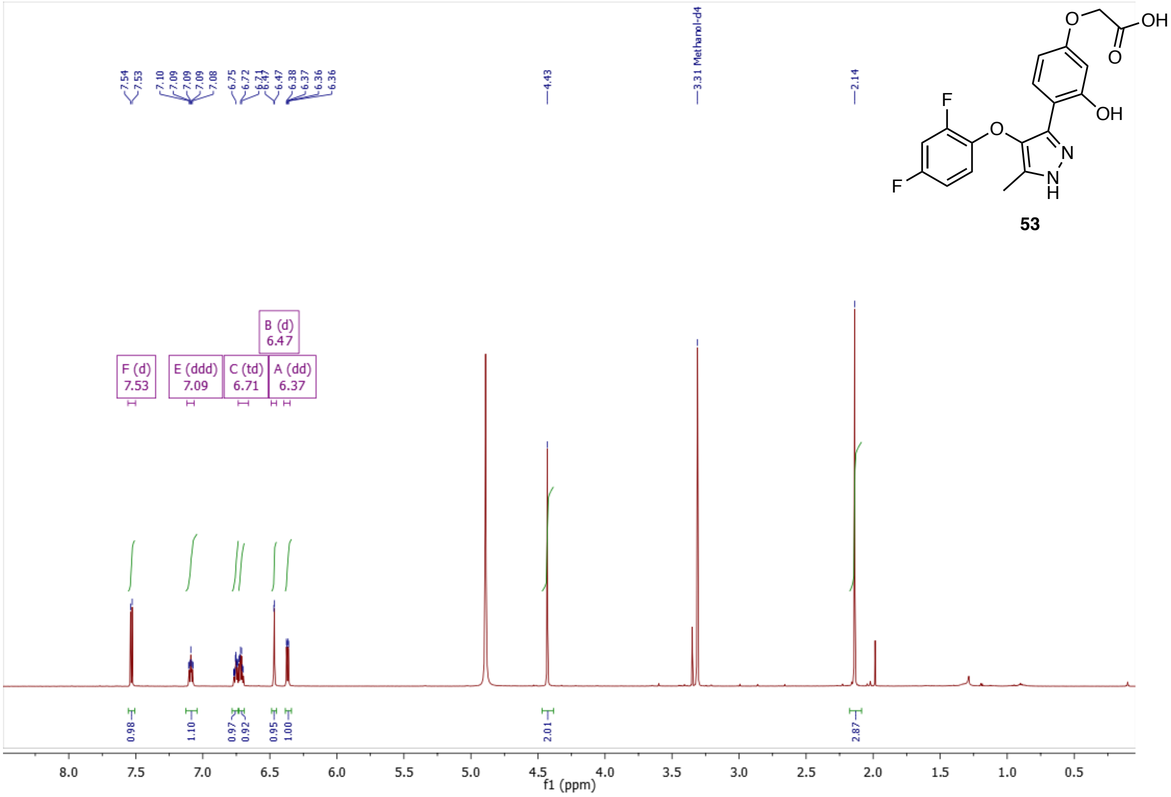

^{13}C NMR spectrum for **53** (CD_3OD, 175 MHz):

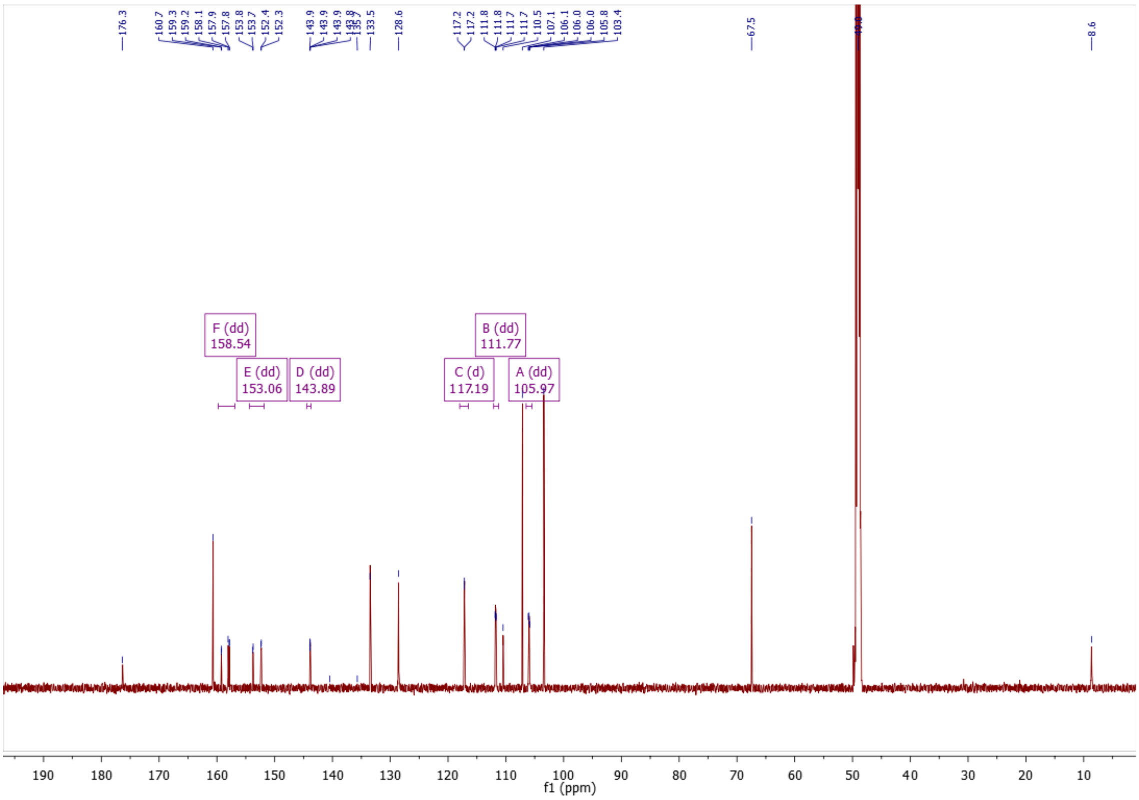

HPLC traces of compound **53**:

(ESI positive mode)

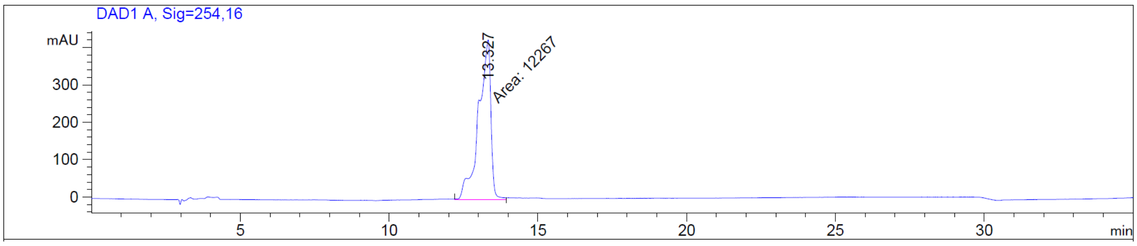

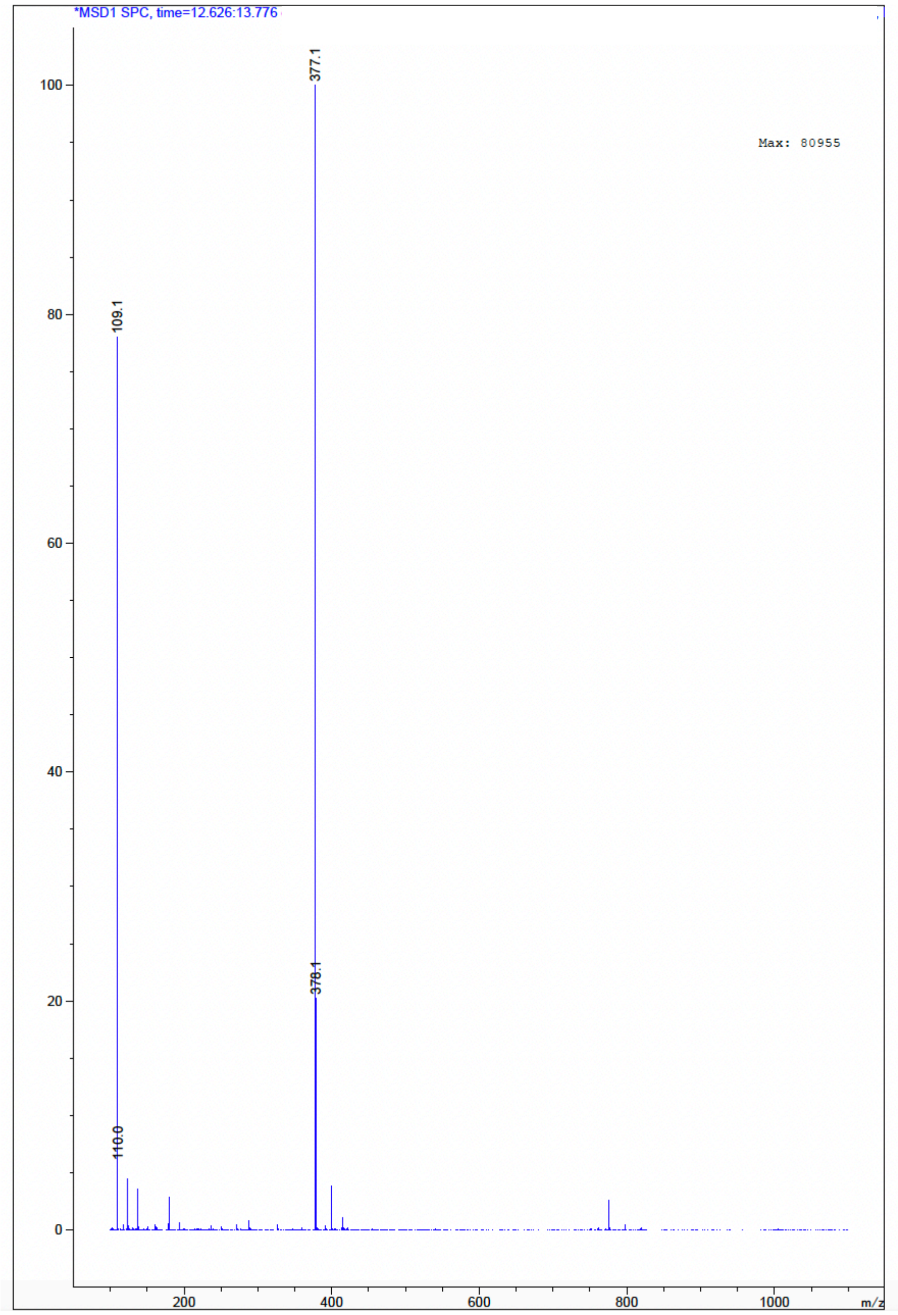

^{1}H NMR spectrum for **54** (CD_3OD, 300 MHz):

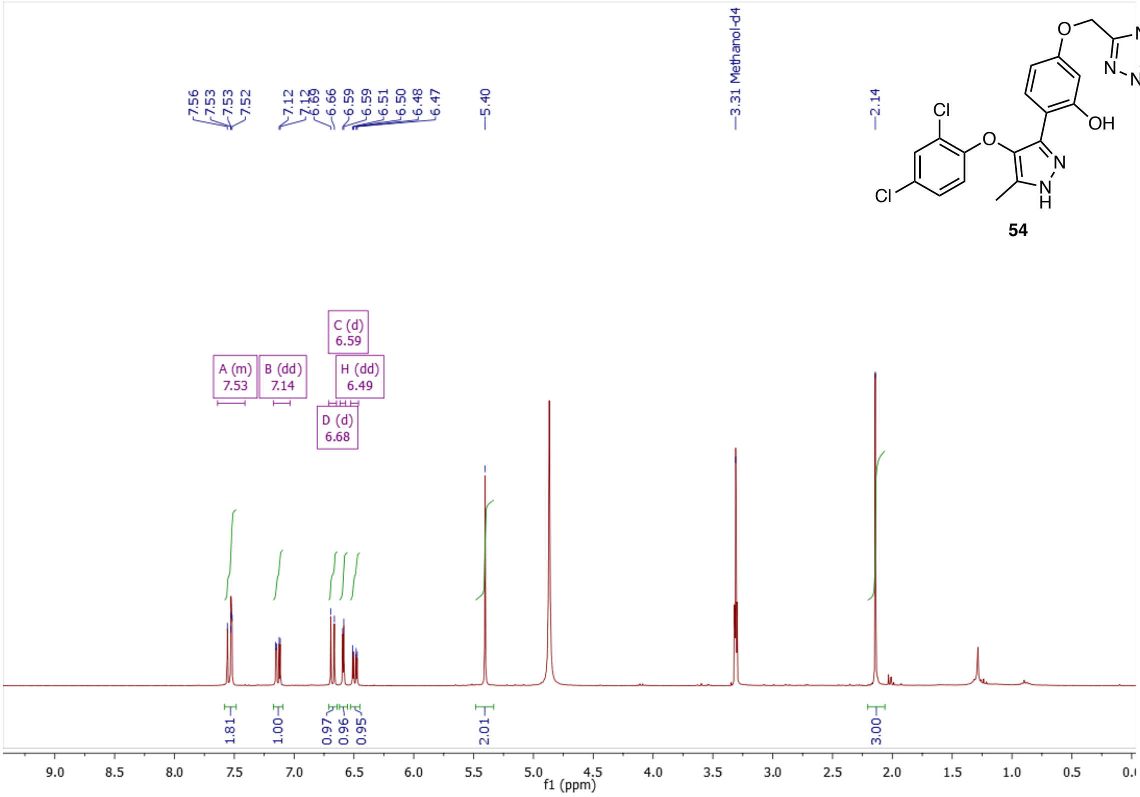

^{13}C NMR spectrum for **54** (CD_3OD, 75 MHz):

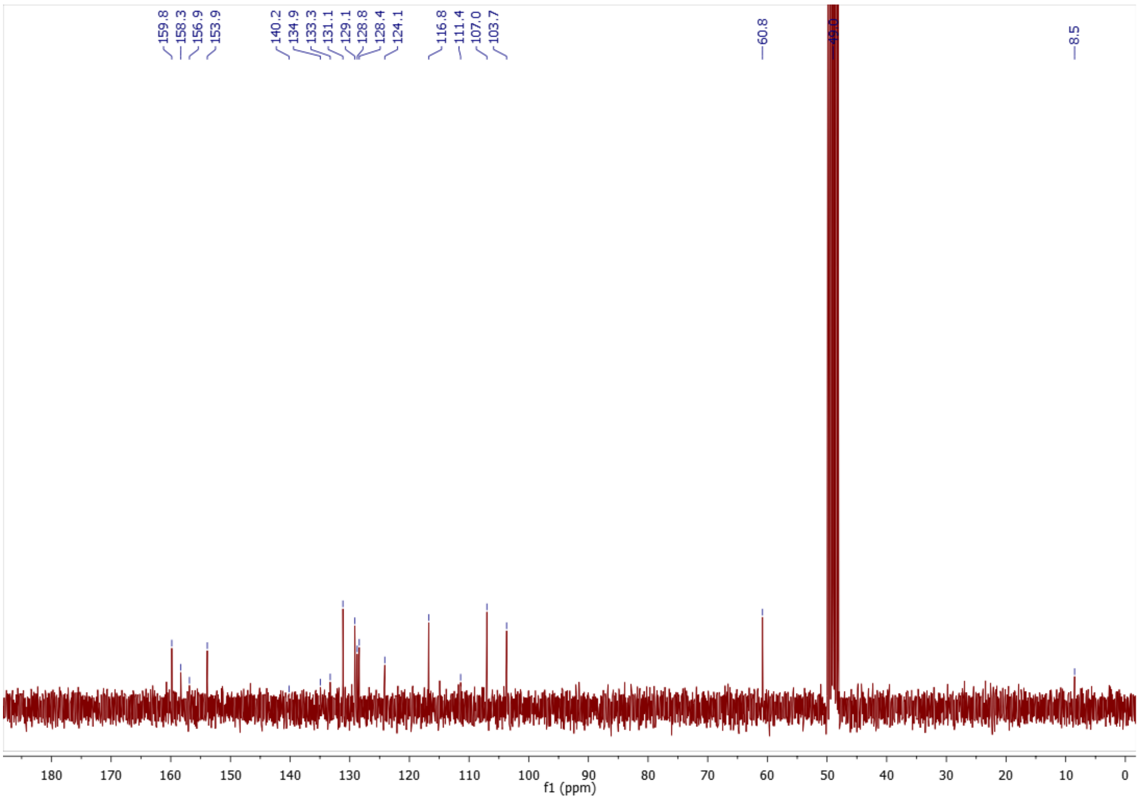

HPLC traces of compound **54**:

(ESI positive mode)

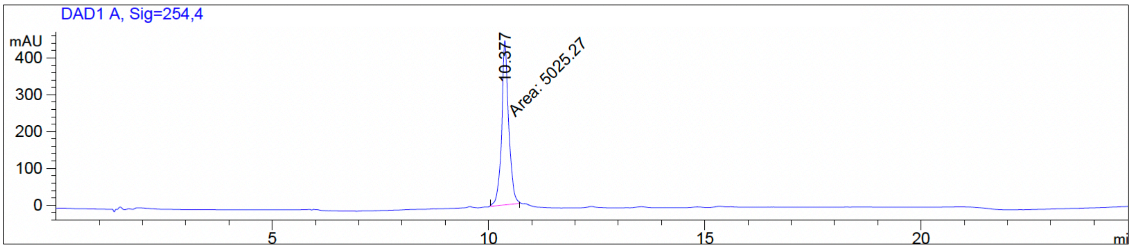

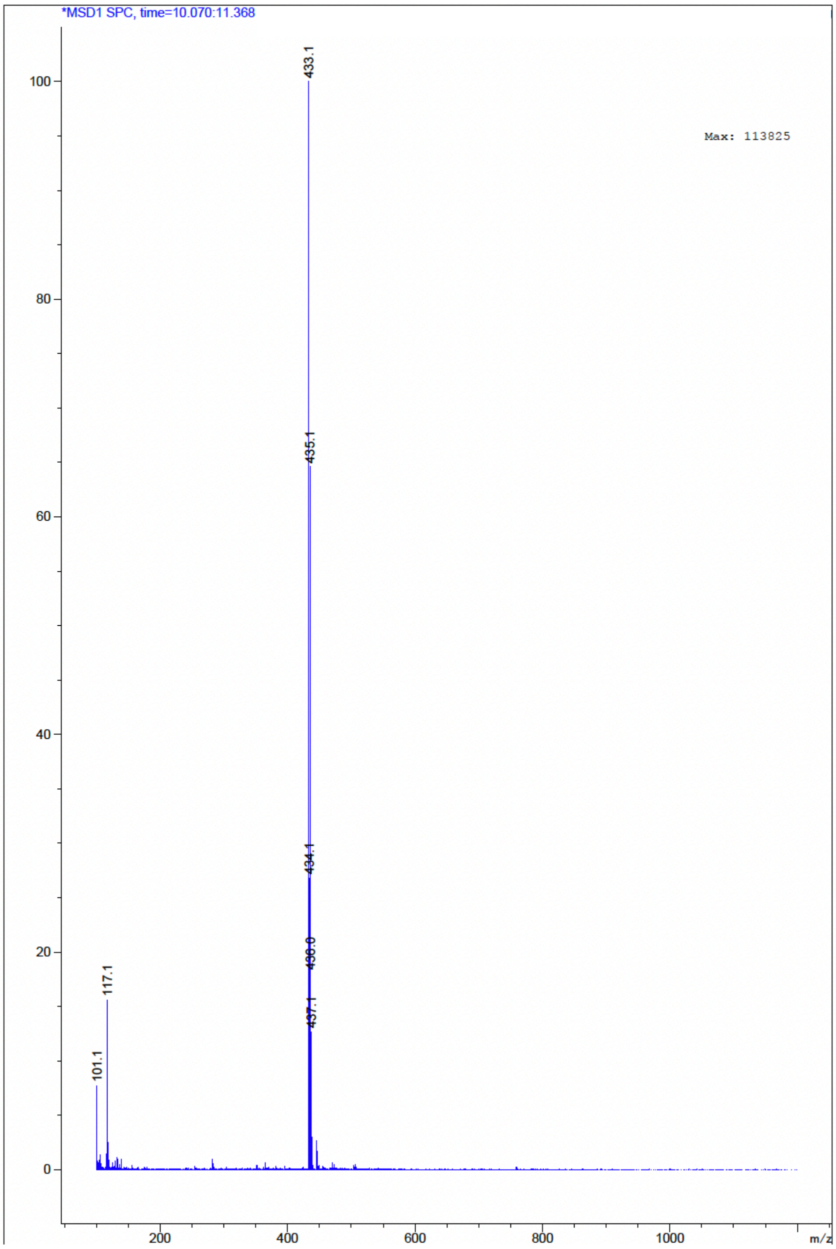

^{1}H NMR spectrum for **55** (CD_3OD, 700 MHz):

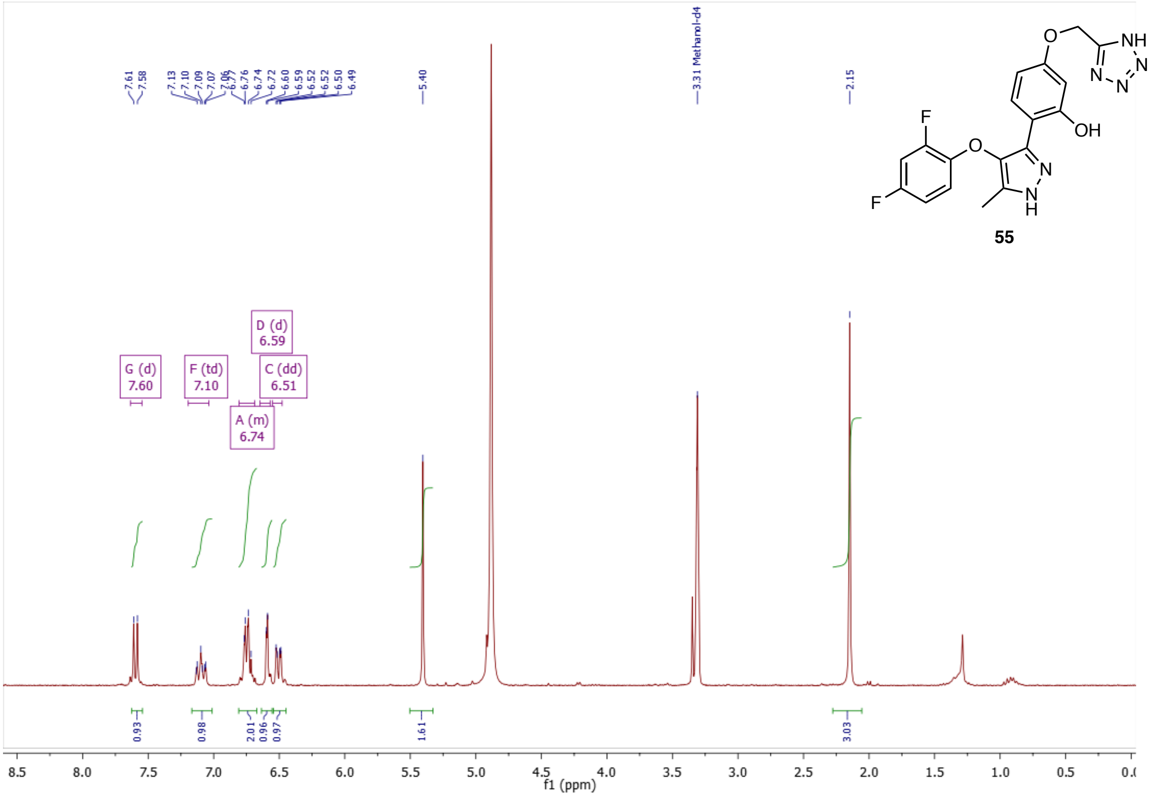

HPLC traces of compound **55**:

(ESI positive mode)

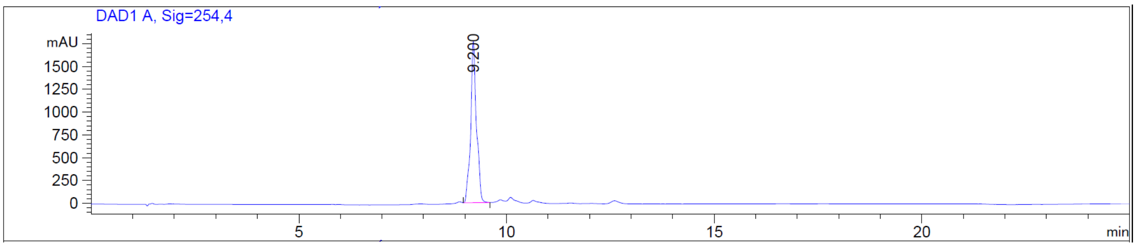

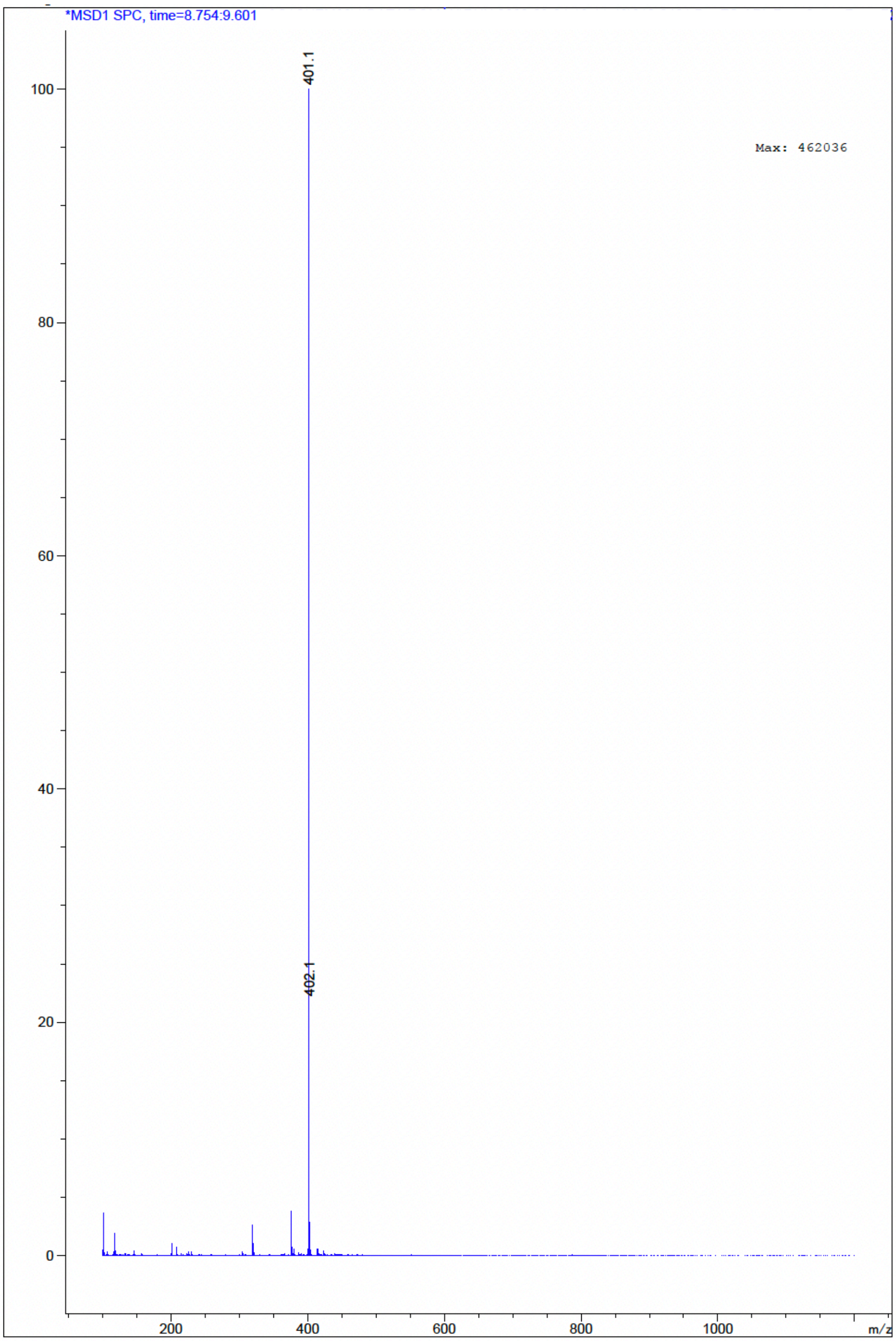

Supplement: Supplementary file 1 — jm2c00046_si_001.pdf [file jm2c00046_si_001.pdf]
